# Chemical Profile Analysis and Comparison of Two Versions of the Classic TCM Formula Danggui Buxue Tang by HPLC-DAD-ESI-IT-TOF-MS^n^

**DOI:** 10.3390/molecules19055650

**Published:** 2014-04-30

**Authors:** Ya-Zhou Zhang, Feng Xu, Tao Yi, Jian-Ye Zhang, Jun Xu, Yi-Na Tang, Xi-Chen He, Jing Liu, Hu-Biao Chen

**Affiliations:** 1 School of Chinese Medicine, Hong Kong Baptist University, 7 Baptist University Road, Kowloon, Hong Kong, China; 2 Guizhou College of Technology, No.1 Caiguan Road, Guiyang 550003, China; 3 State Key Laboratory of Natural and Biomimetic Drugs, School of Pharmaceutical Sciences, Peking University, 38 Xueyuan Road, Beijing 100191, China

**Keywords:** Radix Hedysari, Radix Astragali, Danggui Buxue Tang, HPLC-DAD-ESI-IT-TOF-MS^n^, isoflavonoid, astragaloside

## Abstract

Danggui Buxue Tang (DBT) is a Traditional Chinese Medicine (TCM) formula primarily used to treat symptoms associated with menopause in women. Usually, DBT is composed of one portion of Radix Angelicae Sinensis (RAS) and five portions of Radix Astragali (RA). Clinically, Radix Hedysari (RH) is sometimes used by TCM physicians to replace RA in DBT. In order to verity whether the chemical constituents of the DBT1 (RA:RAS = 5:1, w/w) and DBT2 (RH:RAS = 5:1, w/w) share similarities the chemical profiles of the two DBTs crude extracts and urine samples were analyzed and compared with the aid of HPLC-DAD-ESI-IT-TOF-MS^n^, which determines the total ion chromatogram (TIC) and multi-stage mass spectra (MS^n^). Then, the DBT1 and DBT2 were identified and compared on the basis of the TIC and the MS^n^. In the first experiment (with crude extracts), 69 compounds (**C1**–**C69**) were identified from the DBT1; 46 compounds (**c1**–**c46**) were identified from the DBT2. In the second experiment(with urine samples), 44 compounds (**M1**–**M44**) were identified from the urine samples of rats that had been administered DBT1, and 34 compounds (**m1**–**m34**) were identified from the urine samples of rats that had been administered DBT2. Identification and comparison of the chemical compositions were carried out between the DBT1 and DBT2 of the crude extracts and urine samples respectively. Our results showed that the two crude extracts of the DBTs have quite different chemical profiles. The reasons for their differences were that the special astragalosides in DBT1 and the isoflavonoid glycosides formed the malonic acid esters undergo single esterification and acetyl esters undergo acetylation in DBT1. In contrast, the urine from DBT1-treated rats strongly resembled that of DBT2-treated rats. These metabolites originate mainly from formononetin, calycosin and their related glycosides, and they were formed mainly by the metabolic process of reduction, deglycosylation, demethylation, hydrogenation and sulfation. The HPLC-DAD-ESI-IT-TOF-MS^n^ method was successfully applied for the rapid chemical profiles evaluation of two DBTs and their related urine samples.

## 1. Introduction

Danggui Buxue Tang (DBT) is a Traditional Chinese Medicine (TCM) formula primarily used to treat symptoms associated with menopause in women. It is believed to invigorate ‘Qi’ (vital energy) and nourish the ‘Blood’ (body circulation) [1]. Nowadays, it is commonly used in China as an efficacious medicinal prescription and a healthy food supplement. Pharmacological studies have found that DBT promotes hematopoietic function [2,3], regulates blood lipid and anti-inflammatory activities in diabetic atherosclerosis [4,5], anti-fibrosis effects [6], prevents osteoporosis [7,8], and increases anti-oxidation activity as well as immune response [9]. According to its original formula, DBT comprises Radix Astragali (RA) and Radix Angelicae Sinensis (RAS) (5:1, w/w). More recently, Radix Hedysari (RH) has been used to replace RA. Thus, in current clinical applications, DBT is prescribed in two forms: RA:RAS (5:1) (called DBT1), and RH:RAS (5:1) (called DBT2) [1,10].

The plants RA and RH belong to the same botanical family but different genus, and have long been widely used as the same crude herb in DBT [1]. This is always a question of whether RH can replace RA in the DBT decoction. Chemically, RA-containing DBT showed higher amounts of calycosin-7-*O*-*β*-d-glucoside, ferulic acid, ononin, calycosin, astragaloside IV, astragaloside III, and *Z*-ligustilide. Only formononetin was higher in RH-containing DBT. In parallel, the estrogenic, osteogenic and erythropoetic effects of RA-containing DBT1 showed better activities than that of RA-containing DBT2 [1]. So far, the chemical differences between DBT1 and DBT2 has not been investigated. Therefore, we designed a systematic comparison of the chemical ingredients of DBT1 and DBT2. 

Two experiments were designed, including thorough elucidation of the chemical profiles of DBT1 and DBT2 crude extracts and illumination of the metabolites of DBT1 and DBT2 after being administrated to rats. The chemical profiles of the two DBTs were compared by determining the total ion chromatogram(TIC) and the multistage mass spectra (MS^n^) from HPLC-DAD-ESI-IT-TOF-MS^n^. Subsequently, DBT1 and DBT2 were identified and compared on the basis of the TIC and the MS^n^ [11]. The results will be provide a solid evidence to understand the chemical profiles of the two different versions of DBT.

## 2. Results and Discussion

### 2.1. Optimization the Conditions of HPLC and Mass Spectrometry

In order to obtain desirable HPLC and mass spectrometry chromatograms, the procedures for preparation of the urine samples and crude extracted samples of the two DBTs were optimized in terms of the extraction solvents and extraction times. Methanol and acetonitrile were initially selected as the extraction solvents, but methanol is less poisonous and produced almost the same chromatograms as acetonitrile, so it was applied as the final extraction solvent. For comparison, different columns (Phenomenex RP C_18_, Agilent RP C_18_) were tested for sample separation, and Phenomenex RP C_18_ gave the best chromatographic resolution. The column was eluted with a gradient mobile phase that consisted of water-formic acid (100:0.1, v/v) (A), acetonitrile (B) and at a flow rate of 1.0000 mL/min, in addition, 0.1% (v/v) formic acid was added to improve the mass spectrometry ionization efficiency and enable symmetric peak shapes [12]. Both the positive ion (PI) and negative ion(NI) modes were tested for the experiment. Since MS and MS^n^ fragmentations gave more information about the isoflavones in PI mode but about saponins in NI mode, the analysis was simultaneously conducted in both PI and NI mode. 

### 2.2. The Identification and Analysis of 19 Reference Compounds

**Figure 1:**
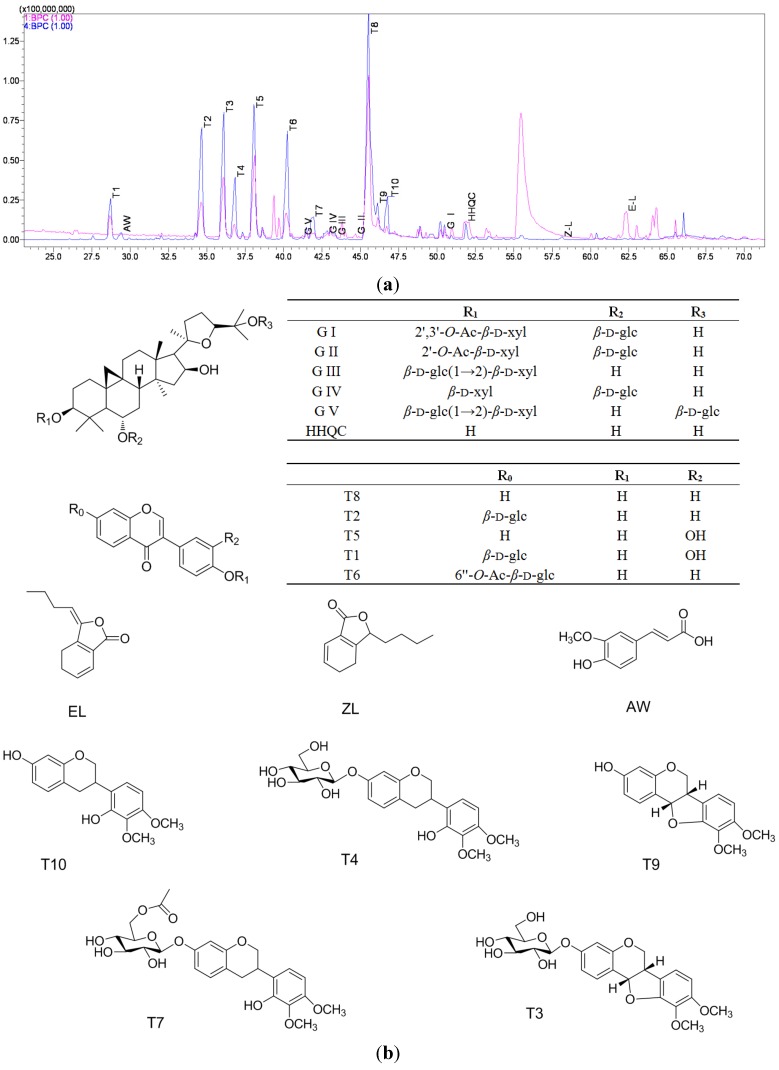
(**a**) LC-MS chromatogram of 19 reference compounds in PI (**1BPC**) and NI (**4BPC**) mode. (**b**) The chemical structures of 19 reference compounds.

Nineteen reference compounds which might represent the major structural types of the DBTs were analyzed. The characteristic fragment ions of 19 references are very useful for determining the structural skeleton and the substitution patterns of those related compounds in two DBTs. Their high resolution mass spectra (HRMS) data are summarized (see Table 1 and Supplementary: pages S2–S8). The base peak chromatograms (BPCs) detected in NI and PI mode were recorded (see Figure 1a). Furthermore, the structure of 19 reference compounds were shown (Figure 1b) [11,13]. 

According to our preliminary research, there three main types of isoflavonoids are found in Radix Astragali which were named as **a**: isoflavone (T1, T2, T5, T6, and T8), **b**: isoflavan (T4, T7, and T10) and c: pterocarpan (T3, and T9) [11,13,14,15]. To facilitate the structural identification of the isoflavonoids in the DBTs, the fragmentation behaviors of the three types of isoflavonoids were analyzed, which might represent the major structural types. We first studied the MS^n^ fragmentation behaviors in PI and NI mode, and found that the fragmentation behaviors in PI mode could give more information about the structure than in NI modes. Then we elucidated the structure of the three types of isoflavonoids mostly from the PI mass spectra.

**Figure 2:**
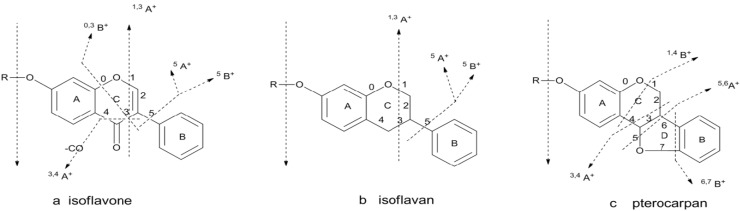
The bond cleavage pathways of the three types of isoflavonoids in RA.

Isoflavone had the characteristic fragment ions ^5^B^+^-2H, ^0,3^B^+^-2H, ^5^A^+^-2H, ^1,3^A^+^-2H, ^3,4^A^+^-2H, *etc.*, isoflavan had the characteristic fragment ions ^5^B^+^-2H, ^5^A^+^-2H, ^1,3^A^+^-2H, *etc.*, and pterocarpan had the characteristic fragment ions ^6,7^B^+^-2H, ^1,4^B^+^-2H, ^3,4^A^+^-2H, ^5,6^A^+^-2H, *etc.*, based on MS^2^ and MS^3^ spectra by HPLC-DAD-ESI-IT-TOF-MS^n^ (see Figure 2). 

For example, we identified the characteristic malonate-glucose-, acetyl-glucose- and glucose-binding ingredients with a neutral loss of 248 Da, 204 Da, and 162 Da, otherwise, glucuronide metabolites with a neutral loss of (−176 Da) and sulfated metabolites with a neutral loss of (−80 Da) from the molecular ion peaka in the MS^2^ spectra [13,16,17].

**Table 1:** The fragment ions of 19 kinds of reference compounds by HPLC-DAD-ESI-IT-TOF-MS^n^.

NO.	T_R_ (min)	[M+H]^+^	[M−H]^−^	Predicted Formula	Fragment Ions Da	Error (ppm)	The Name of the Reference Compounds
1(T1)	28.595	447.1290		C_22_H_22_O_10_	447,285,270,225	0.89	Calycosin-7-*O*-*β*-d-glucopyranoside
2(AW)	29.162		193.0501	C_10_H_10_O_4_	193,178,134	−2.59	Ferulic acid
3(T2)	34.545		431.1326	C_22_H_22_O_9_	431,269,253,237,213,197,163,134,107	−2.55	Ononin
4(T3)	36.038	485.1400	[M+Na]^+^	C_23_H_26_O_10_	485,463,323,301	−3.71	Astrapterocarpan-7-*O*-*β*-d-glucopyranoside
5(T4)	36.785	487.1574	[M+Na]^+^	C_23_H_28_O_10_	487,303,167	−0.21	Astraisoflavan-7-*O*-*β*-d-glucopyranoside
6(T5)	38.055	285.0754		C_16_H_12_O_5_	285,270,253,225,197,137	−1.40	Calycosin
7(T6)	40.123	473.1432		C_24_H_24_O_10_	473,269	−2.11	6''-*O*-acetyl-ononin
8(T7)	41.942	529.1691	[M+Na]^+^	C_25_H_30_O_11_	529,507,303	2.08	6''-*O*-acetyl-astraisoflavan-7-*O*-*β*-d-glucopyranoside
9(T8)	45.410	269.0795		C_16_H_12_O_4_	269,270,237	−4.83	Formononetin
10(T9)	46.062	301.1076		C_17_H_16_O_5_	301,271,251,167,151,134	1.66	(6aR,11aR)-3-hydroxy-9,10-dimethoxypterocarpan; Astrapterocarpan
11(T10)	46.655	303.1213		C_17_H_18_O_5_	303,181,167,149,123	−4.62	(3R)-7,2'-dihydroxy-3',4'-dimethoxyisoflavan; Astraisoflavan
12(GⅤ)	41.110	[M+HCOO]^−^	991.5156	C_47_H_78_O_19_	991,783,397	3.73	Astragaloside Ⅴ
13(GⅣ)	42.775	[M+HCOO]^−^	829.4615	C_41_H_68_O_14_	829,783,621,489,383	2.89	Astragaloside Ⅳ
14(GⅢ)	43.410	[M+HCOO]^−^	829.4585	C_41_H_68_O_14_	829,783,651,489	−0.72	Astragaloside Ⅲ
15(GⅡ)	44.628	[M+HCOO]^−^	871.4693	C_43_H_70_O_15_	871,765,717	−0.46	Astragaloside Ⅱ
16(GⅠ)	50.525	[M+HCOO]^−^	913.4802	C_45_H_72_O_16_	913,867,807	0.00	Astragaloside Ⅰ
17(HHQC)	51.855	513.3550	[M+Na]^+^	C_30_H_50_O_5_	513,515,405,229	0.00	Cycloastragenol(HHQC)
18(ZL)	55.477	191.1056		C_12_H_14_O_2_	191,173,117	−5.76	*Z*-ligustilide
19(EL)	58.102	191.1066		C_12_H_14_O_2_	191,173	−0.52	*E*-ligustilide

### 2.3. Profiling and Identifying Chemical Compounds of the Two Crude Extracts (DBT1, and DBT2) by HPLC-DAD-ESI-IT-TOF-MS^n^

#### 2.3.1. Identification of the Chemical Profiles of DBT1 by HPLC-DAD-ESI-IT-TOF-MS^n^

**Figure 3:**
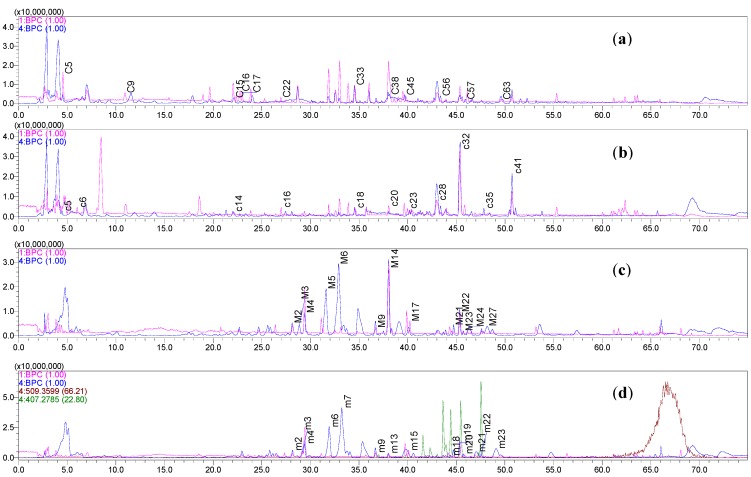
(**a**) The BPC in NI and PI mode of the crude extracts of Danggui Buxue Tang 1 (RA:RAS = 5:1). (**b**) The BPC in NI and PI mode of the crude extracts of Danggui Buxue Tang2 (RH:RAS = 5:1). (**c**) The BPC in NI and PI mode of the urine samples of rats that had been administrated the Danggui Buxue Tang 1 (RA:RAS = 5:1) and (**d**) The BPC in NI and PI mode of the urine samples of rats had been administrated the Danggui Buxue Tang 2 (RH:RAS = 5:1).

The HRMS data of these identified compounds are summarized (see Table 2 and Supplementary: pages S9–S35). The BPCs detected in PI (**1BPC**) and NI (**4BPC**) mode were also recorded (Figure 3a) [17,18,19].

By comparing the fragment ions and retention times and based on the high resolution mass spectra software predicted formulas with the reference compounds from the MS and MS^n^, the compounds **C23**, **C33**, **C36**, **C37**, **C38**, **C44**, **C46**, **C56**, **C58**, **C59**, **C49**, **C53**, **C60**, **C64**, and **C68** were identified as the reference compounds [11,13].

**C27** has a RT at 30.773 min, [M+HCOO]^−^ at *m/z* 671.2155 in MS (predicted formula: C_29_H_38_O_15_: ppm error: −5.66), and characteristic fragment ions at *m/z* 625.2079 (−46 Da) [M-H]^−^, *m/z* 463.1589 [M-162-H], and *m/z* 301.1108 [M-162-162-H] in MS^2^. The neutral loss is mass 46 Da (CH_2_O_2_; identified as HCOOH), 162 Da*2 (C_6_H_10_O_5_; identified as glucopyranoside), and the fragment ion *m/z* 301.1108 predicted as C_17_H_18_O_5_. Then C27 was identified as astraisoflavan-di-7-*O*-*β*-d-glucoside or its isomer. The [M+H]^+^ or [M-H]^−^ of **C24**, **C29**, **C30***,* and **C41** shows the same neutral loss of −162 Da (C_6_H_10_O_5_; glucoside) in them MS^2^, so both of them were identified as the glycosides [17,18,19]. The characteristic fragment ions of **C31**, **C35**, **C43**, **C42**, and **C51** have a neutral loss of −248 Da (C_9_H_12_O_8;_ identified as the 6''-*O*-malonate-glucoside) in them MS^2^, so both of them were identified as glycosides of 6''-*O*-malonate-glucoside [13].The HRM software predicted [M+H]^+^ or [M-H]^−^ of **C32**, **C40**, **C34**, **C47***,* and **C18***,* whose formulas have the same characteristic fragment loss (−204 Da; C_8_H_12_O_6_) which was identified as 6''-*O*-acetylglucosides. In addition, **C32**, **C40**, **C34**, **C4**7, and **C18** were identified as glycosides of 6''-*O*-acetylglucoside [16]. 

For the predicted formulas of [M+H]^+^ or [M-H]^−^, we tentatively identified **C39**, **C50**, **C62**, **C30**, and **C41** as the isoflavonoid-related constituents by referring to the literature [18,19], and **C45**, **C55**, **C48**, **C52**, **C54**, **C61**, and **C65** were tentatively identified as being related to saponins [13,17,18,19].

Using the [M+H]^+^, [M-H]^−^ or [M+Na]^+^ data of **C7**, **C8**, **C10**, **C12**, **C13**, **C14**, **C21**, **C25**, **C26**, and **C28**, we predicted their formulas, which indicates that they are the ingredients of the samples. However, at this point, their exact structures could not be identified.

**Table 2:** The identified proposed compounds of the crude extract samples from Danggui Buxue Tang 1 and Danggui Buxue Tang 2 by HPLC-DAD-ESI-IT-TOF-MS^n^.

NO.	T_R_ (min)	[M+H]^+^	[M−H]^−^	Predicted Formula	Fragment Ions Da	Error ppm	Identification	DBT1	DBT2
1	2.395		173.1044	C_6_H_14_N_4_O_2_		0.00	Arginine	**C1**	**c1**
2	2.692		195.0502	C_6_H_12_O_7_		−4.10	Gluconic acid	**C2**	**c2**
3	2.695		341.1074	C_12_H_22_O_11_		−4.40	d(+)sucrose	**C3**	**c3**
4	3.643		191.0187	C_6_H_8_O_7_	191,173	−5.24	Citric acid	**C4**	**c4**
5	4.477		328.0427	C_11_H_11_N_3_O_9_		1.22	--	**C5**	**c5**
6	6.480	346.0529		C_11_H_11_N_3_O_10_		3.47	--	**C6**	**c6**
7	9.063		433.1364	C_18_H_26_O_12_	301,191	4.91	--	**C7**	**--**
8	9.120	443.1146	[M+Na]^+^	C_17_H_24_O_12_		−3.16	----	**--**	**c7**
9	9.345	267.1369		C_18_H_18_O_2_	267,225	−4.12	Magnolol	**--**	**c8**
10	9.398		433.1364	C_18_ H_26_O_12_	433,351,301,223	2.77	--	**C8**	**--**
11	10.773	188.0688		C_9_ H_11_NO_2_	146	3.19	L-phenylalanine	**C9**	**c9**
12	11.125		431.1192	C_18_ H_24_O_12_	431,299	−0.70	--	**C10**	**--**
13	12.653	384.1127		C_13_H_21_NO_12_		−2.60	--	**C11**	**c10**
14	13.840		461.1283	C_19_H_26_O_13_	461,167	−3.90	--	**--**	**c11**
15	13.847	485.1224	[M+Na]^+^	C_26_H_22_O_8_	485,317	3.50	--	**C12**	**--**
16	17.807		205.0701	C_8_H_14_O_6_		−8.29	--	**C13**	**--**
17	21.147		315.2004	C_20_H_28_O_3_		12.06	--	**C14**	**--**
18	21.207		433.1129	C_21_H_22_O_10_	433,285	−2.54	--	**--**	**c12**
19	21.322		433.1121	C_21_H_22_O_10_	433,285,241	−4.39	--	**--**	**c13**
20	22.020		417.1017	C_17_H_22_O_12_	417,285,152	-5.03	--	**C15**	**c14**
21	23.080	389.2325		C_23_H_32_O_5_		0.51	--	**C16**	**c16**
22	23.018		401.1445	C_18_H_26_O_10_	401,269,161	−1.99	--	**C17**	**c15**
23	23.590		503.1175	C_24_H_24_O_12_	503,443,299	−3.98	6''-*O*-acetyl-pratensein-7-*O*-*β*-d-glucoside	**C18**	**--**
24	24.347		239.0568	C_11_H_12_O_6_		2.93	--	**C19**	**--**
25	25.662	331.2296		C_21_H_30_O_3_	331,299	8.45	--	**C20**	**--**
26	26.743	470.1534		C_18_H_23_N_5_O_10_		3.40	--	**C21**	**--**
27	26.967	289.1747		C_13_H_24_N_2_O_5_	289,272,152	−3.80	--	**C22**	**c17**
28	30.653		479.1492	C_30_H_24_O_6_	479,317	−1.67	--	**C26**	**--**
29	30.773	[M+HCOO]^−^	671.2155	C_29_H_38_O_15_	671,625,463,301	−5.66	Astraisoflavan-di-7-*O*-*β*-d-glucoside	**C27**	**--**
30	31.005		579.2062	C_23_H_36_N_2_O_15_	579,417,387	3.28	--	**C28**	**--**
31	31.348		445.1123	C_22_H_22_O_10_	445,283	−3.82	Glycetein-4'-*O*-*β*-d-glucoside	**C29**	**--**
32	32.078	463.1203		C_22_H_22_O_11_	463,301	−6.91	Kaempferide-7-*O*-*β*-d-glucoside	**C30**	**--**
33	32.662	533.1267		C_25_H_24_O_13_	533,285	−4.31	6''-*O*-malonate-calycosin-7-*O*-*β*-d-glucoside	**C31**	**--**
34	34.207	489.1398		C_24_H_24_O_11_	489,285	1.43	6''-*O*-acetyl-calycosin-7-*O*-*β*-d-glucoside	**C32**	**--**
35	34.517	431.1322		C_22_H_22_O_9_	431,269,237,118	−3.48	Ononin	**C33**	**c18**
36	35.348		489.1340	C_24_H_26_O_11_	489,285,271,159	−12.68	6''-*O*-acetyl-isosakuranetin-7-*O*-*β*-d-glucoside	**C34**	**--**
37	35.580	549.1174		C_25_H_24_O_14_	549,301	−11.84	6''-*O*-malonate-kaempferide-7-*O*-*β*-d-glucoside	**C35**	**--**
38	36.027	463.1615		C_23_H_26_O_10_	485(+Na^+^),463,301	3.45	Astrapterocarpan-7-*O*-*β*-d-glucopyranoside	**C36**	**--**
39	36.773		463.1577	C_23_H_28_O_10_	463,301,271	−7.13	Astraisoflavan-7-*O*-*β*-d-glucopyranoside	**C37**	**--**
40	37.480		255.0657	C_15_H_12_O_4_	255,135	−2.35	Isoliquiritigenin	**--**	**c19**
41	38.035	285.0744		C_16_H_12_O_5_	285,270,225,137	−4.91	Calycosin	**C38**	**c20**
42	39.917		269.0807	C_16_H_14_O_4_	269,253,227	−4.46	Isomer of alpinetin	**--**	**c21**
43	39.977		255.0657	C_15_H_12_O_4_	255,237	−2.35	Liquiritigenin	**--**	**c22**
44	38.653	315.0844		C_17_H_14_O_6_		−6.03	4-methoxy-maackiain or the isomer	**C39**	**--**
45	38.653	473.1445		C_24_H_24_O_10_	473,269	0.63	The isomer of 6''-*O*-acetyl-ononin	**C40**	**--**
46	38.773	447.1264		C_22_H_22_O_10_	447,285	−4.92	Glycetein-7-*O*-*β*-d-glucoside	**C41**	**--**
47	38.997	549.1545		C_26_H_28_O_13_	549,301	−10.56	6''-*O*-malonate-astrapterocarpan-glucoside	**C42**	**--**
48	39.452	517.1301		C_25_H_24_O_12_	517,269	−7.73	6''-*O*-malonate-ononin	**C43**	**--**
49	39.683		505.1699	C_25_H_30_O_11_	505,301	−3.17	6''-*O*-acetyl-astraisoflavan-7-*O*-*β*-d-glucoside	**C44**	**--**
50	39.745		957.5030	C_48_H_78_O_19_	957,541,453	−3.66	Soyasaponin Ba	**C45**	**c23**
51	40.138	473.1424		C_24_H_24_O_10_	473,269	−3.80	6''-*O*-acetyl-ononin	**C46**	**--**
52	40.903		927.4915	C_47_H_76_O_18_		−4.74	Akebia saponin d	**--**	**c24**
53	40.430		503.1154	C_24_H_24_O_12_	503,299	−8.15	6''-*O*-acetyl-kaempferide-7-*O*-*β*-d-glucoside	**C47**	**--**
54	40.825		785.4629	C_41_H_70_O_14_		−8.15	Cyclocanthoside E	**C48**	**--**
55	41.108		991.5086	C_48_H_80_O_21_		−3.33	Astragaloside V	**C49**	**--**
56	41.273		285.0751	C_16_H_14_O_5_	285,194,109	−5.96	Isomer of isosakuranetin	**--**	**c25**
57	41.772		269.0456	C_15_H_10_O_5_	269,237	0.37	Genistein	**--**	**c26**
58	41.778		315.0868	C_17_H_16_O_6_	315,253	−1.90	Astragaluquinone or isomer	**C50**	**--**
59	41.950	533.1243		C_25_H_24_O_13_	533,285	−8.82	6''-*O*-malonate-glycetein-7-*O*-*β*-d-glucoside	**C51**	**--**
60	42.062		867.4635	C_45_H_72_O_16_		−13.03	Isoastragaloside I	**C52**	**--**
61	42.243		283.0602	C_16_H_12_O_5_	283,268,224	−3.53	Glycetein	**--**	**c27**
62	42.612	[M+HCOO]^−^	829.4572	C_41_H_68_O_14_		−2.29	Astragaloside IV	**C53**	**--**
63	42.560		825.4532	C_43_H_70_O_15_	871,825	−13.33	Isoastragaloside II	**C54**	**--**
64	42.965		329.2319	C_18_H_34_O_5_		−4.25	--	**--**	**c28**
65	43.197		955.4857	C_48_H_76_O_19_		−5.34	--	**--**	**c29**
66	44.690		287.0577	C_15_H_12_O_6_		5.57	Dihydro-kaempferol	**--**	**c30**
67	44.982		255.0649	C_15_H_12_O_4_	256,135	−5.49	Isomer of Liquiritigenin	**--**	**c31**
68	45.315	269.0791		C_16_H_12_O_4_	269,254,237,118	−6.32	Formononetin	**C56**	**c32**
69	44.190		941.5081	C_48_H_78_O_18_	941,525,437	−3.61	Soyasaponin Bb	**C55**	**c35**
70	45.745	299.0911		C_17_H_14_O_5_	299,284,166		Pterocarpin	**--**	**c33**
71	45.935		329.2299	C_18_H_34_O_5_		−10.33	--	**C57**	**--**
72	46.080		283.0599	C_16_H_12_O_5_	283,255,240	−4.59	The isomer of glycetein	**--**	**c34**
73	46.158	301.1052		C_17_H_16_O_5_		−6.31	Astraoptercarpan	**C58**	**--**
74	46.708	303.1181		C_17_H_18_O_5_		−15.18	Astraisoflavan	**C59**	**--**
75	46.768	[M+HCOO]^−^	871.4656	C_43_H_70_O_15_	871,825,603	−4.70	Astragaloside II	**C60**	**--**
76	47.172	[M+HCOO]^−^	911.4668	C_45_H_70_O_16_	955,911	2.41	--	**C61**	**--**
77	47.772		299.0552	C_16_H_12_O_6_		−3.01	Kaempferide or isomer	**--**	**c36**
78	47.943		909.4836	C_47_H_74_O_17_		−1.87	Acetylastragaloside I	**--**	**c37**
79	48.347	[M+HCOO]^−^	911.5011	C_46_H_74_O_15_		0.11	Castaraleside H	**--**	**c38**
80	49.432		285.0423	C_15_H_10_O_6_	285,163	6.31	Kaempferol or isomer	**C62**	**--**
81	50.065		939.4925	C_48_H_76_O_18_		−3.62	--	**--**	**c39**
82	50.435	285.0748		C_16_H_12_O_5_	285,253,152	−3.51	Isomer of calycosin	**--**	**c40**
83	50.667	335.2180	[M+Na]^+^	C_18_H_32_O_4_		−3.88	--	**C63**	**c41**
84	50.713	[M+HCOO]^−^	913.4777	C_45_H_72_O_16_		−2.74	Astragaloside I	**C64**	**--**
85	51.560	193.1212		C_12_H_16_O_2_		−5.70	Senkyunolide A	**--**	**c42**
86	51.607	[M+HCOO]^−^	953.4637	C_47_H_72_O_17_	953,909	−12.06	--	**C65**	**--**
87	52.363	437.3374	[M+Na]^+^	C_28_H_46_O_2_		−3.66	--	**C66**	**--**
88	55.212	191.1043		C_12_H_14_O_2_		−12.56	n-butyl-phthalide	**--**	**c43**
89	59.250	213.0876	[M+Na]^+^	C_12_H_14_O_2_	403,213	−4.69	*Z*-ligustilide	**C67**	**c44**
90	61.708	403.1867	[2M+Na]+	C_12_H_14_O_2_	403,381,191	−3.22	*E*-ligustilide	**C68**	**c45**
91	69.093		283.0257	C_15_H_8_O_6_	283,203,147	3.18	--	**C69**	**c46**

#### 2.3.2. Identification of the Chemical Profiles of DBT2 by HPLC-DAD-ESI-IT-TOF-MS^n^

The HRMS data of these identified compounds are summarized (see Table 2 and Supplementary: pages S36–S51). The BPCs detected in NI and PI modes were recorded (see Figure 3b).

Using their fragment ions and retention times in MS data, **c18**, **c20**, **c32**, **c44**, and **c45** were identified as the reference compounds [11,13].

Based the MS data, **c12** shows RT at 21.207 min, [M-H]^−^ at *m/z* 433.1129 in MS (predicted the formula: C_21_H_22_O_10_: ppm error: −2.54), and characteristic fragment ions at *m/z* 285.0744 (−148 Da; C_5_H_8_O_5_; identified as the ribonic acid) and predicted as C_16_H_14_O. Compound **c12** was identified as the isomer of isosakuranetin-ribonic acid. Moreover, **c13** shows a characteristic neutral loss at −148 Da (C_5_H_8_O_5_) with the same as **c12** [16]. 

By the formulas predicted of [M-H]^−^ or [M+H]^+^ and referring to literature [13,17,18,19], **c23**, **c24**, **c29**, **c35**, **c38**, and **c39** were tentatively identified as saponin-related constituents, and **c7**, **c8**, **c11**, **c19**, **c21**, **c22**, **c25**, **c26**, **c27**, **c31**, **c33**, **c34**, **c36**, **c40**, and **c43** were tentatively identified (see in Table 2).

By using the HRMS data (RT, Predicted the formulas and characteristic fragment ions) compared with the BDT1 crude extract samples, **C1**, **c1**; **C2**, **c2**; **C3**, **c3**; **C4**, **c4**; and **C9**, **c9** were identified as the same constituents [17,18,19]. 

The groups of **C5**, **c5**; **C6**, **c6**; **C11**, **c10**; **C15**, **c14**; **C16**, **c16**; **C17**, **c15**; **C22**, **c17**; **C57**, **c28**; **C63**, **c41**; and **C69**, **c46** between DBT1 and DBT2 were tentatively identified as the same compounds with uncertain structures. 

**Figure 4:**
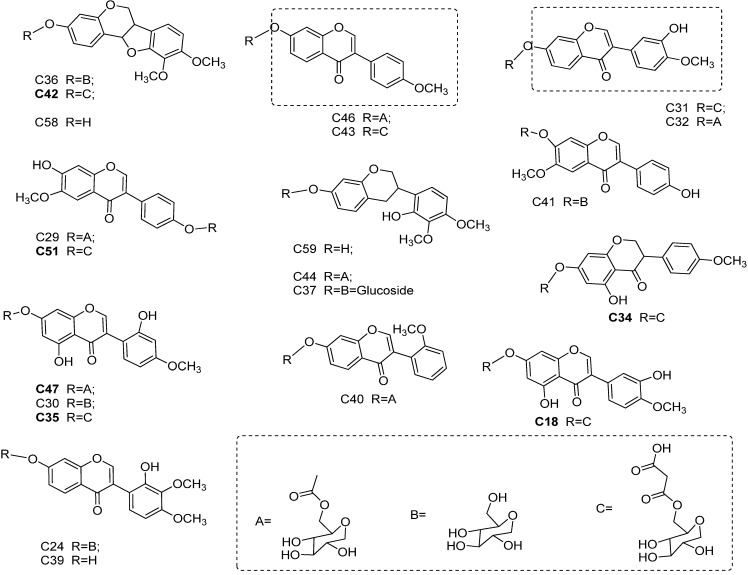
The chemical structures of the main proposed different isoflavonoids in Danggui Buxue Tang 1 more than Danggui Buxue Tang 2.

From the analysis based on the comparison of TIC and MS^n^: 69 compounds (**C1–C69**) were identified from the crude extracts of DBT1, 46 compounds (**c1–c46**) were identified from the crude extracts of DBT2. The isoflavonoids glycosides had experienced acetylation (seven compounds, **C18**, **C32**, **C34**, **C40**, **C44**, **C46**, and **C47**), formed the malonate acid esters (five compounds, **C31**, **C35**, **C42**, **C43**, and **C51**) and with special astragalosides (six compounds, **C49**, **C52**, **C53**, **C54**, **C60**, and **C64**) in DBT1. Thus, the number of identified components in DBT1 was significantly more than in DBT2 (the chemical structural diversity of isoflavonoids which were detected in DBT1 more than in DBT2 are shown in Figure 4). Among these, the 24 common chemical constituents accounted for approximately 27% to the total 91 identified compounds. However, and the proportion of the total isoflavonoids and saponins to the total identified ingredients accounted for nearly 62% (see Table 3). 

**Table 3:** The number comparison of the identified compounds between Danggui Buxue Tang 1 and Danggui Buxue Tang 2.

N.	ID.	S-ID.	T.ID.	S%	H + S	S%
DBT1	69	24	115−24 = 91	26.37	56	61.54
DBT2	46					
DBT1-U	44	19	78−19 = 59	32.20	48	81.35
DBT2-U	34					

ID. Total identified proposed compounds number; S-ID. Identified the common proposed compounds number between two Danggui Buxue Tangs; T.ID. Identified the unfamiliar proposed compounds number between two Danggui Buxue Tangs; H + S: The total isoflavones and the total saponins number; S% The ratio about the selective compounds in the total identified proposed compounds number.

### 2.4. Profiling and Identifying Chemical Profiles of the Urine Samples after Administration of the DBT1 and DBT2 Performed by HPLC-DAD-ESI-IT-TOF-MS^n^

In the study of the existing literature, ingredients such as isoflavones, saponins in the two DBTs had no obvious differences in chemical profiles between the serum and bile samples collected from enterohepatic circulation. In addition, they had a lower concentration in serum samples, even when giving at dosages of 60–120 g/kg (w/w) several times to rats within 24 h [13,17,20]. Thus, this approach is not conducive to tracing these minor components. This study chose the normal usage of 10 g/kg (w/w) by comparing the urine samples of rats that were administrated two different DBTs, so as to improve the detection through enrichment of the treatments. 

#### 2.4.1. Identification of the Chemical Profiles of Urine Sample after Administration of the DBT1 by HPLC-DAD-ESI-IT-TOF-MS^n^

**Figure 5:**
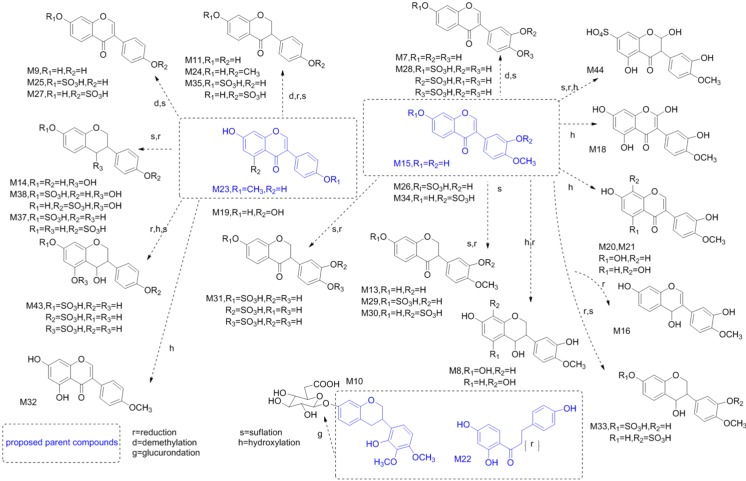
The main proposed metabolites identified from the urine samples of rats that had been administrated Danggui Buxue Tang 1.

The HRMS data of these identified metabolites are summarized (see Table 4 and Supplementary: pages S52–S66). The BPCs detected in NI mode were recorded (see Figure 3c). In addition, the main proposed structures of these metabolites identified from the urine samples of rats that had been administrated DBT1 were showed (see Figure 5). 

Using MS data with the reference compounds, **M15** was identified as calycosin, and **M23** was identified as formononetin [11,13]. 

With the predictions of [M-H]^−^ or [M+HCOO]^−^, and the characteristic fragment ions, **M9**, **M7**, **M19**, **M8**, **M10**, **M11**, **M13**, **M14**, **M22**, **M16**, **M18**, **M20**, **M21**, **M24**, **M32**, and **M36** were tentatively identified as the metabolites of isoflavonoids [13,17,18,19,21].

**M25** shows RT at 46.290 min, [M+H]^+^ at *m/z* 335.0201 in MS (predicted formula: C_15_H_10_O_7_S: ppm error: −5.67), and characteristic fragment ions at *m/z* 255.0637 (−80 Da; SO_3_; identified as the sulfonyl hydroxide) and predicted as C_15_H_10_O_4__._ Then M25 was identified as daidzein after sulfation. **M27**, **M26**, **M34**, **M28**, **M29**, **M30**, **M31**, **M33**, **M35**, **M37**, **M38**, **M43**, and **M44** have the same neutral loss of −80 Da, which was identified as the sulfonyl hydroxide (SO_3_), so they were identified as the sulfated products [13,16].

When predicting their formulas, **M39**, **M41**, and **M42** were identified as related metabolites of saponins [13,17].

**Table 4:** The identified proposed metabolites from the urine samples of rats that had been administrated Danggui Buxue Tang1 and Danggui Buxue Tang 2.

NO.	T_R_ (min)	[M+H]^+^	[M-H]^−^	Predicted Formula	Fragment Ions	Error ppm	Identification	DBT1	DBT2
1	4.132		287.0065	C_10_H_8_O_10_		6.97	--	**M1**	**m1**
2	28.200		231.0768	C_12_H_12_N_2_O_3_	463,231	−3.03	--	**M2**	**m2**
3	29.298		233.0115	C_12_H_2_N_4_O_2_	233,169	4.29	--	**M3**	**m3**
4	29.298		337.1408	C_16_H_22_N_2_O_6_	337,253	0.89	--	**M4**	**m4**
5	31.083	268.1164	266.1021	C_13_H_17_NO_5_		−4.89	--	**--**	**m5**
6	31.338		275.0209	C_13_H_8_O_7_	275,195	4.36	--	**M5**	**m6**
7	32.927		273.0056	C_13_H_6_O_7_	273,193	5.49	--	**M6**	**m7**
8	33.038	271.0585		C_15_H_10_O_5_		−5.90	Hydroxydaidzein	**M7**	**--**
9	34.518	[M+HCOO]^−^	475.1244	C_22_H_22_O_9_	475,267	−0.42	Isomer of ononin	**--**	**m8**
10	36.155		303.0863	C_16_H_16_O_6_	303,151	−3.63	Hydroxylcalycosin, direduction(C^2^=C^3^; C^4^=O)	**M8**	**--**
11	36.705		253.0492	C_15_H_10_O_4_		−5.53	Daidzein	**M9**	**m9**
12	36.808		477.1372	C_23_H_26_O_11_	477,301	−6.29	Astraisoflavan, glucuronidation	**M10**	**--**
13	36.868		255.0662	C_15_H_12_O_4_	255,149	−0.39	Daidzein, reduction(C^2^=C^3^)	**M11**	**m10**
14	37.255	385.1478		C_18_H_24_O_9_		−3.89	Hydroligustilide, glucuronidation	**M12**	**--**
15	37.442		255.0655	C_15_H_12_O_4_		−3.14	Daidzein, reduction(C^4^=O)	**--**	**m11**
16	37.502		285.0751	C_16_H_14_O_5_	285,269,149	−5.96	Calycosin, reduction(C^2^=C^3^)	**M13**	**m12**
17	37.195		257.0809	C_15_H_14_O_4_		−3.89	Daidzein, direduction(C^2^=C^3^; C^4^=O)	**M14**	**--**
18	38.033		283.0608	C_16_H_12_O_5_	283,268	−1.41	Calycosin	**M15**	**m13**
19	38.362		285.0751	C_16_H_14_O_5_	285,270	−5.96	Calycosin, reduction(C^4^=O)	**M16**	**--**
20	39.047		273.0761	C_15_H_14_O_5_	273,240,109	−2.56	Hydroxydaidzein, direduction (C^2^=C^3^; C^4^=O)	**--**	**m14**
21	39.367		233.0098	C_12_H_2_N_4_O_2_		−3.00	--	**M17**	**m15**
22	40.260	[M+HCOO]^−^	363.0748	C_16_H_14_O_7_		7.16	Dihydroxycalycosin, reduction(C^2^=C^3^)	**M18**	**--**
23	41.630		283.0609	C_16_H_12_O_5_	283,268,224	−1.06	Isomer of calycosin		**m16**
24	41.755		269.0441	C_15_H_10_O_5_		−5.20	Hydroxydaidzein	**M19**	**--**
25	42.180		283.0603	C_16_H_12_O_5_	283,268,224	−3.18	Isomer of calycosin	**--**	**m17**
26	42.365		299.0554	C_16_H_12_O_6_	299,284	−2.34	Hydroxycalycosin, or isomer	**M20**	**--**
27	42.923		299.0556	C_16_H_12_O_6_	299,284	−1.67	Hydroxycalycosin	**M21**	**--**
28	44.315		257.0819	C_15_H_14_O_4_		0.00	Isoliquiritigenin, reduction(C=C)	**M20**	**m18**
29	45.322	269.0796		C_16_H_12_O_4_	269,253,237	−4.46	Formononetin	**M23**	**m19**
30	45.733		269.0804	C_16_H_14_O_4_	269,254,135	−5.57	Formononetin, reduction(C^2^=C^3^)	**M24**	**m20**
31	46.290	335.0201		C_15_H_10_O_7_S	335,255	−5.67	Daidzein, sulfation	**M25**	**m21**
32	47.775		299.0556	C_16_H_12_O_6_	299,256	−1.67	Hydroxycalycosin	**--**	**m22**
33	47.862		363.0174	C_16_H_12_O_8_S	363,268	−1.65	Calycosin, sulfation	**M26**	**--**
34	47.922		333.0059	C_15_H_10_O_7_S	333,253,225	−4.50	Daidzein, sulfation	**M27**	**--**
35	48.747		365.0347	C_16_H_14_O_8_S	365,285	2.74	Calycosin, reduction(C^2^=C^3^), sulfation	**M29**	**--**
36	49.072		333.0056	C_15_H_10_O_7_S	333,253,208	−5.41	Daidzein, sulfation	**--**	**m23**
37	49.543		333.0059	C_15_H_10_O_7_S	333,253	−4.50	Isomer of daidzein, sulfation	**--**	**m24**
38	49.193		365.0360	C_16_H_14_O_8_ S	365,285	6.30	Calycosin, reduction(C^2^=C^3^), sulfation	**M30**	**--**
39	49.810		349.0033	C_15_H_10_O_8_S	349,269,225	2.58	Hydroxydaidzein, sulfation	**M28**	**m25**
40	49.623		351.0187	C_15_H_12_O_8_S	351,271,149	1.99	Hydroxydaidzein, reduction(C^2^=C^3^), sulfation	**M31**	**--**
41	50.325		337.0395	C_15_H_14_O_7_S	337,257	2.37	Daidzein, direduction(C^2^=C^3^; C^4^=O), sulfation	**--**	**m27**
42	50.467		283.0595	C_16_H_12_O_5_	283,268	−6.01	Isomer of calycosin	**M32**	**--**
43	50.973		367.0483	C_16_H_16_O_8_S	367,272,150	−2.72	Calycosin, direduction(C^2^=C^3^; C^4^=O), sulfation	**M33**	**--**
44	51.515		363.0180	C_16_H_12_O_8_S	363,283	0.00	Calycosin, sulfation	**M34**	**m26**
45	52.563		335.0250	C_15_H_12_O_7_S	335,255,135	5.67	Daidzein, reduction(C^2^=C^3^), sulfation	**M35**	**--**
46	53.637		343.0835	C_18_H_16_O_7_		3.50	--	**M36**	**--**
47	55.693		351.0172	C_15_H_12_O_8_S	351,271	−2.28	Hydroxydaidzein, reduction(C^2^=C^3^), sufaltion	**--**	**m28**
48	55.165		321.0417	C_15_H_14_O_6_S	321,241	−6.54	Equol, sulfation	**M37**	**m29**
49	59.468		337.0388	C_15_H_14_O_7_S	337,257,243	0.30	Daidzein, direduction(C^2^=C^3^; C^4^=O), sulfation	**M38**	**--**
50	59.728	619.3669	[M+Na]^+^	C_36_H_52_O_7_		10.33	Related to astragaloside	**--**	**m30**
51	61.838	683.4277		C_35_H_64_O_11_		−9.57	Related to astragaloside	**M39**	**--**
52	62.002	639.4061		C_35_H_58_O_10_		−6.57	Related to astragaloside	**M40**	**--**
53	62.113	595.3742		C_33_H_54_O_9_		−16.63	Related to astragaloside	**M41**	**m31**
54	63.292		509.3599	C_33_H_50_O_4_		−7.26	Related to astragaloside	**--**	**m32**
55	62.414	507.3296		C_29_H_46_O_7_		−3.94	Related to astragaloside	**M42**	**--**
56	71.557		353.0324	C_15_H_14_O_8_S	353,273	−3.68	Hydroxydaidzein, direduction(C^2^=C^3^;C^4^=O) sulfation	**M43**	**--**
57	71.557		397.0250	C_16_H_14_O_10_S	397,317	3.78	Dihydroxycalycosin, reduction(C^2^=C^3^), sulfation	**M44**	**--**
58	72.797		363.0197	C_16_H_12_O_8_S	363,283	4.68	Calycosin, sulfation	**--**	**m33**
59	73.543		347.0213	C_16_H_12_O_7_S	347,267	−5.19	Formononetin, sulfation	**--**	**m34**

#### 2.4.2. Identification of the Chemical Profiles of Urine Sample after Administration of the DBT2 by HPLC-DAD-ESI-IT-TOF-MS^n^

**Figure 6:**
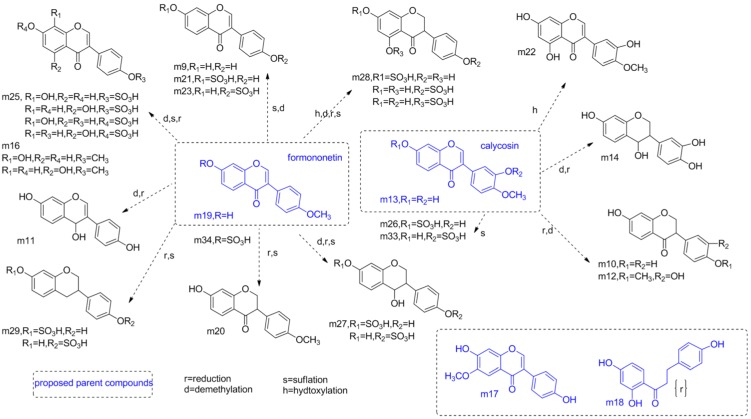
The main proposed metabolites identified from the urine samples of rats that had been administrated Danggui Buxue Tang 2.

The HRMS data of these identified metabolites are summarized (see Table 4 and Supplementary: pages S67–S77). The BPCs detected in NI model were recorded (see in Figure 3d). The main proposed structures of metabolites identified from the urine samples of rats that had been administrated DBT2 were showed (see Figure 6). 

The MS data of **m13**, and **m19** show that they are the reference compounds. In addition, **m13** was identified as calycosin, and **m19** was identified as formononetin [11,13].

Moreover, **m8** has a RT at 34.518 min, [M+HCOO]^−^ at *m/z* 475.1244 in MS (predicted formula: C_22_H_22_O_9_: ppm error: −0.42), and characteristic fragment ions at *m/z* 267.0656 that are predicted as C_16_H_12_O_4_, the neutral loss is 46 Da (HCOOH) + 162 Da (C_6_H_10_O_5_; identified as glucoside). Thus, **m8** was identified as the isomer of ononin [13].

In their MS and MS^2^ Data, **m21**, **m23**, **m22**, m24, **m25**, **m26**, **m33**, **m27**, **m28**, **m29**, and **m34** have the same neutral loss of −80 Da, which was predicted as the sulfonyl hydroxide (SO_3_), so they were identified as the sulfated products [13,16].

By the MS data of [M-H]^−^ or [M+HCOO]^−^ and the characteristic fragment ions, **m9**, **m10**, **m11**, **m12**, **m14**, **m16**, **m17**, **m18**, and **m20** were identified as the metabolites of isoflavonoids [13,14,15,17,18,21]. Using their predictions of [M+Na]^+^ or [M+H]^+^, **m30**, **m31**, and **m32** were tentatively identified as saponin-related metabolites [13,17]. 

Between DBT1 and DBT2, the groups **M1**, **m1**; **M2**, **m2**; **M3**, **m3**; **M4**, **m4**; **M5**, **m6**; **M6**, **m7**; and **M15**, **m17** were identified as the same compounds, respectively. 

In this part of the experiment, decoctions of DBT1 and DBT2 were administered to rats, and an analysis was conducted on the rats’ urine for metabolites: 44 compounds (**M1–M44**) were identified from the urine samples after DBT1 was administrated, 34 compounds (**m1–m34**) were identified from the urine samples after DBT2 was administrated. The number of the chemical components in the urine samples from DBT1 was administrated to rats are slightly more than these of DBT2. The proportion of the 19 common constituents to the total 59 identified compounds accounted for approximately 33%. The proportion of 48 total isoflavonoids and saponins to the total identified compounds, however, reached approximately 82% (See Table 3). 

The phase Ⅱ metabolites from the urine samples of DBT1 and DBT2 were dominantly sulfated products, with rare or no glucuronide metabolites. This result still requires further research as the literature reports the chief presence of mainly glucuronide metabolites [13,14,15,16]. Metabolites that originated from RAS are relatively rare or not detected. This is likely due to the low proportion of RAS in DBT and even the low dosage that was given to rats in this study, those chemical constituents of RAS being easy to volatilize, or a loss when sampling was conducted by concentrated processes [22,23]. At this normal dosage of DBT and due to poor absorption, the content levels of astragalosides are much lower. In addition, saponins and their metabolites that originated from astragalosides are rarely detected [24,25,26,27].

The HPLC-DAD-ESI-IT-TOF-MS*^n^* method adopted in our research was confirmed to be a powerful method to evaluate the chemical profiles of the crude extracts and the related urine samples. As we know, the chemical composition found in a Chinese herbal decoction is rather complicated. In this study, the chemical profile analysis of DBT1 and DBT2 was conducted, which provided a comprehensive understanding of that those isoflavonoids that play an important role in the main common chemical basis when they are used in clinical practice. Some identified metabolites are known to have many bioactivities, such as calycosin, formononetin, daidzein and equol (which are well-known phytoestrogens), and most of them displayed many beneficial effects to humans [28,29,30]. 

Through the comparison of chemical profiles of two DBTs at our used normal dosage, the similarity of urine samples is higher than that of the crude extract samples. This leads us to believe that the main chemical basis of the chemical constituents is almost the same. Whether the DBT2 of RH:RAS can replace the DBT1 of RA:RAS, we need to further investigate different ratios of RH:RAS with the RA: RAS (5:1) when using equivalent pharmacological research.

## 3. Experimental

### 3.1. Materials and Reagents

Radix Astragali was collected from Shanxi Province (Voucher No. 130401, Specimen No. 1167), Radix Hedysari was collected from Neimeng Province (Voucher No. 130401, Specimen No. 1168) and Radix Angelica sinensis was collected from Gansu Province (Voucher No. 130401, Specimen No. 1169). All of those medicinal materials were purchased from Guangzhou Zixing Herbal Company in Guangzhou at June in 2013 by Liu Jing and they are identified by Prof. Chen Hu-Biao. The following reference compounds: (6aR,11aR)-3-hydroxy-9,10-dimethoxypterocarpan (astrapterocarpan), ononin, formononetin, 6''-*O*-acetyl-ononin, calycosin, ferulic acid, (3R)-7,2'-dihydroxy-3',4'-dimethoxyisoflavan (astraisoflavan), calycosin-7-*O*-*β*-d-glucoside, astrapterocarpan-7-*O*-*β*-d-glucoside, *Z*-ligustilide, *E*-ligustilide, astragaloside I, astragaloside II, astragaloside III, astragaloside IV, astragaloside V, astraisoflavan-7-*O*-*β*-d-glucoside and 6''-*O*-acetyl-astraisoflavan-7-*O*-*β*-d-glucoside were prepared and identified in our preliminary work [10,11,12,13,14]. Acetonitrile (Merck Co., Darmstadt, Germany) and formic acid (Mreda Technology Inc., Beijing, China) were of HPLC grade. Ultra-pure water was prepared by a Milli-Q water purification system (Millipore, Billerica, MA, USA). 

### 3.2. Sample Preparation

In clinical use, DBT is typically boiled with water twice, then the two decoctions are combined and applied [1]. Therefore, in our study, DBT1, consisting of RA 100 g and RAS 20 g was boiled in 1,000 mL of water (w:v) for 45 min, and then the decoction was filtered. The residue was again boiled in 700 mL of water (w:v) for 30 min. The two decoctions were evaporated to dryness under reduced pressure at 50 °C to 100 mL volume. Samples of DBT2, consisting of RH 100 g and RAS 20 g, were prepared in the same way. 

### 3.3. Animals and Administration

12 male Sprague-Dawley (SD) rats (220–250 g) were provided by the Experimental Animal Center of Peking University Health Science Center (Beijing, China) and divided into two groups. They were housed in metabolic cages (Type: DXL-DL, Suzhou Fengshi Laboratory Animal Equipment Co. Ltd., Suzhou, China), and kept in an environmentally controlled breeding room for one-week acclimation. Throughout the experiments, rats had unrestricted access to laboratory chow and water. The DBT1 and DBT2 were administrated by oral at a dose of raw medicinal material 10 g/kg body weight once a day (at 17:30 pm) respectively. Totally for 2 days. All procedures used in the animal experiments were conducted in accordance with the Guide for the Care and Use of Laboratory Animals of the US National Institute of Health. The experiments were reviewed by the Biomedical Ethical Committee of Peking University (Approval No. LA2013-193).

### 3.4. Urine Sample Collection and Pretreatment

Urine samples in each group (n = 6) were collected during the first 48 h after administration of the drugs began(Blank urine collected by self-control.); Finally, all urine samples from the same group were merged into one sample, then dried under vacuum at 50 °C using a Heidolph Laborota 4001 rotatory evaporator (Heidolph Instruments GmbH & Co., Schwabach, Germany), and then 1.00 g of the dried samples were reconstituted in 10 mL methanol, followed by 30 min ultrasonic extraction and 15 min centrifugation at 5,000 rpm. Afterward, the supernatant was collected for detection. 

### 3.5. Instrumentations and Conditions

HPLC analysis was performed on a Shimadzu HPLC (Shimadzu, Kyoto, Japan) equipped with two LC-20AD pumps, aCTO-20A column oven, an SIL-20AC autosampler, an SPD-M20A PDA detector and a CBM-20A system controller. The chromatographic separation was carried out on a Phenomenex Gemini C_18_ column (250 × 4.6 mm, 5 μM) protected with a Phenomenex Security Guard column (4 × 3.0 mm, 5 μM) (Phenomenex, Torrance, CA, USA). For each sample, an aliquot of 20 μL was injected with needle wash. The thermostatted auto-sampler was maintained at 15 °C; column oven temperature was kept at 30 °C. The column was eluted with a gradient mobile phase consisted of water-formic acid (100:0.1, v/v) (A) and acetonitrile (B) at the flow rate of 1.0000 mL/min. Gradient program was adopted in the following manner: 5% B at 0–10 min, 5%–15% B at 10–20 min, 15%–40% B at 20–40 min, 40%–65% B at 40–55 min, 65%–100% B at 55–65 min, 100% B at 65–75 min, 5% B at 75–85 min.

High resolution mass spectra were recorded on an IT-TOF mass spectrometer (Shimadzu). The ESI source was operated both in negative and positive ion mode. The mass spectrometry was programmed to carry out full scan over *m/z* 100–1000 Da (MS^1^), *m/z* 50–1000 Da (MS^2^ and MS^3^). A trifluoroacetic acid sodium solution (2.5 mM) was used to calibrate the mass range from 50 to 1000 Da. The other parameters were set as follows: flow rate, 0.20 mL/min (split from 1.00 mL/min HPLC effluent); heat block and curved desolvation line temperature, 200 °C; nebulizing nitrogen gas flow, 1.5 L/min; interface voltage: (+), 4.5 kV; (−), −3.5 kV; detector voltage, 1.70 kV; relative collision-induced dissociation energy (50%) [15].

### 3.6. Data Analysis

All data were recorded and processed by Shimadzu software LCMS solution version 3.60, Formula Predictor version 1.2 and Accurate Mass Calculator (Shimadzu).

## 4. Conclusions

A comparison was conducted on the similarities and differences of crude extracts and urine samples of DBT1 and DBT2. The chemical profiles of the crude extracts comprised a total of 115 proposed chemical components. There were 24 common ingredients, which was accounted for 27% in the total 91 identified components. There were a total of 56 isoflavonoids and saponins identified, which accounted for nearly 62% in the total identified components. Since isoflavonoid glycosides had acetylation (**C18**, **C32**, **C34**, **C40**, **C44**, **C46**, and **C47**), the formation of malonate acid esters (**C31**, **C35**, **C43**, **C42**, and **C51**) and special astragalosides (**C49**, **C52**, **C53**, **C54**, **C60**, and **C64**) in DBT1, the identified compounds from DBT1 were significantly greater than DBT2. Of these, **C18**, **C34**, **C35**, **C42**, **C47**, and **C51** were identified from DBT for the first time.

In total, 78 proposed chemical components in the urine samples of rats that had been administrated DBT1 and DBT2, respectively, were found. These included 19 common ingredients, which accounted for approximately 33% in the total identified constituents. In addition, 48 of total isoflavonoids and saponins were found, which accounted for nearly 82% of the total 59 identified components. The differences between those metabolites in the urine samples were revealed to be less than the crude extracts. These identified metabolites are mainly originated from formononetin, calycosin and their related glycosides, which are formed mainly through the metabolic processes of reduction, deglycosylation, demethylation, hydrogenation and sulfation. Through the comparison of chemical profiles of two DBTs at our used doses, the similarity of urine samples is higher than that of the crude extract samples, one can think the main chemical constituents are almost the same as when administrated to rats.

The HPLC-DAD-ESI-IT-TOF-MS^n^ method was successfully applied for the chemical profile comparison of two different DBTs and its related medicinal materials. The proposed assay provides an important reference and can be a suitable method for the rapid and accurate chemical basis evaluation of TCM or their related prescriptions. 

## Supplementary Materials

Supplementary materials can be accessed at: http://www.mdpi.com/1420-3049/19/5/5650/s1.

## References

[1] Zhang W.L., Choi R.C., Zhan J.Y., Chen J.P., Luk W.K., Yao P., Dong T.T., Tsim K.W. (2013). Can Hedysari Radix replace Astragali Radix in Danggui Buxue Tang, a Chinese herbal decoction for woman aliment?. Phytomedicine.

[2] Zheng K.Y.Z., Choi R.C.Y., Xie H.Q.H., Cheung A.W.H., Guo A.J.Y., Leung K.W., Chen V.P., Bi C.W.C., Zhu K.Y., Chan G.K.L. (2010). The expression of erythropoietin triggered by Danggui Buxue Tang, a Chinese herbal decoction prepared from Radix Astragali and Radix Angelicae Sinensis, is mediated by the hypoxia-inducible factor in cultured HEK293T cells. J. Ethnopharmacol..

[3] Yang M., Chan G.C.F., Deng R.X., Margaret H.N., Cheng S.W., Lau C.P., Ye J.Y., Wang L.J., Liu C. (2009). An herbal decoction of *Radix astragali* and *Radix angelicae sinensis* promotes hematopoiesis and thrombopoiesis. J. Ethnopharmacol..

[4] Zhang H.M., Chen S.W., Deng X.F., Yang X.G., Huang X. (2007). The effects of Danggui-Buxue-Tang on blood lipid and expression of genes related to foam cell formation in the early stage of atherosclerosis in diabetic GK rats. Diabetes Res. Clin. Pr..

[5] Zhang H.M., Chen S.W., Deng X.F., Yang X.G., Huang X. (2006). Danggui–Buxue–Tang decoction has an anti-inflammatory effect in diabetic atherosclerosis rat model. Diabetes Res. Clin. Pr..

[6] Gao J., Huang Y., Li P., Xu D.J., Li J., Liu Y., Huang Z.G., Wu Q., Shao X. (2011). Antifibrosis effects of total glucosides of Danggui-Buxue-Tang in a rat model of bleomycin-induced pulmonary fibrosis. J. Ethnopharmacol..

[7] Xie Q.F., Xie J.H., Dong T.T.X., Su J.Y., Cai D.K., Chen J.P., Liu L.F., Li Y.C., Lai X.P., Tsim K.W.K. (2012). Effect of a derived herbal recipe from an ancient Chinese formula, Danggui BuxueTang, on ovariectomized rats. J. Ethnopharmacol..

[8] Gao Q.T., Choi R.C.Y., Cheung A.W.H., Zhu J.T.T., Li J., Chu G.K.Y., Duan R., Cheung J.K.H., Jiang Z.Y., Dong X.B. (2007). Danggui Buxue Tang-A Chinese herbal decoction activates the phosphorylations of extracellular signal-regulated kinase and estrogen receptor a in cultured MCF-7 cells. FEBS Lett..

[9] Gao Q.T., Cheung J.K.H., Li J., Jiang Z.Y., Chu G.K.Y., Duan R., Cheung A.W.H., Zhao K.J., Choi R.C.Y., Dong T.T.X. (2007). A Chinese herbal decoction, Danggui Buxue Tang, activates extracellular signal-regulated kinase in cultured T-lymphocytes. FEBS Lett..

[10] Li W.Z., Li J., Bi C.W., Cheung A.W., Huang W., Duan R., Choi R.C., Chen I.S., Zhao K.J., Dong T.T. (2009). Can rhizoma chuanxiong replace Radix angelia sinensis in the traditional chinese herbal decoction Danggui Buxue Tang?. Planta Med..

[11] Zhang Y.Z., Xu F., Liang J., Tang J.S., Shang M.Y., Wang X., Cai S.Q. (2012). Isoflavonoids from the roots of *Astragalus membranaceus* var. *Mongholicus*. Zhongguo Zhong Yao Za Zhi.

[12] Fan L.L., Yi T., Xu F., Zhang Y.Z., Zhang J.Y., Li D.P., Xie Y.J., Qin S.D., Chen H.B. (2013). Characterization of flavonoids in the ethomedicine fordiae cauliflorae radix and its adulterant millettiae pulchrae radix by HPLC-DAD-ESI-IT-TOF-MS^n^. Molecules.

[13] Li C.Y., Qi L.W., Li P. (2011). Correlative analysis of metabolite profiling of Danggui Buxue Tang in rat biological fluids by rapid resolution LC-TOF/MS. J. Pharm. Biomed. Anal..

[14] Yi L.Z., Liang Y.Z., Wu H., Yuan D.L. (2009). The analysis of Radix Angelicae Sinensis (Danggui). J. Chromatogr. A.

[15] Polat E., Bedir E., Perrone A., Piacente S., Alankus-Caliskan O. (2010). Triterpenoid saponins from *Astragalus wiedemannianus* Fischer. Phytochemistry.

[16] Zhang Y.Z., Xu F., Dong J., Liang J., Hashi Y., Shang M.Y., Yang D.H., Wang X., Cai S.Q. (2012). Profiling and identification of the metabolites of calycosin in rat hepatic 9000 × *g* supernatant incubation system and the metabolites of calycosin-7-*O*-*β*-d-glucoside in rat urine by HPLC–DAD–ESI-IT-TOF-MS^n^ technique. J. Pharm. Biomed. Anal..

[17] Wen X.D., Liu E.H., Yang J., Li C.Y., Gao W., Qi L.W., Wang C.Z., Yuan C.S., Li P. (2012). Identification of metabolites of Buyang Huanwu decoction in rat urine using liquid chromatography–quadrupole time-of-flight mass spectrometry. J. Pharm. Biomed. Anal..

[18] Qi L.W., Cao J., Li P., Yu Q.T., Wen X.D., Wang Y.X., Li C.Y., Bao K.D., Ge X.X., Cheng X.L. (2008). Qualitative and quantitative analysis of Radix Astragali products by fast high-performance liquid chromatography-diode array detection coupled with time-of-flight mass spectrometry through dynamic adjustment of fragmentor voltage. J. Chromatogr. A.

[19] Liu M.H., Tong X., Wang J.X., Zou W., Cao H., Sua W.W. (2013). Rapid separation and identification of multiple constituents in traditional Chinese medicine formula Shenqi Fuzheng Injection by ultra-fast liquid chromatography combined with quadrupole-time-of-flight mass spectrometry. J. Pharm. Biomed. Anal..

[20] Liu C.F., Qiao X., Liu K.D., Miao W.J., Li Y.J., Liu Y., Jiang Y.Y., Bo T., Shi R.B., Guo D.A. (2013). *In vivo* metabolites and plasma exposure of TongMai Keli analyzed by UHPLC/DAD/qTOF-MS and LC/MS/MS. J. Ethnopharmacol..

[21] Qi L.W., Wen X.D., Cao J., Li C.Y., Li P., Yi L., Wang Y.X., Cheng X.L., Ge X.X. (2008). Rapid and sensitive screening and characterization of phenolic acids, phthalides, saponins and isoflavonoids in Danggui Buxue Tang by rapid resolution liquid chromatography/diode-array detection coupled with time-of-flight mass spectrometry. Rapid Commun. Mass Sp..

[22] Wu W.N., McKown L.A. (2004). Optimization in Drug Discovery.

[23] Williams C.A., Andersen O.M., Markham K.R. (2006). Flavonoids: Chemistry, Biochemistry and Applications.

[24] Zuo A.H., Wang L., Xiao H.B., Li L.M., Liu Y.H., Yi J.H. (2011). Identification of the absorbed components and metabolites in rat plasma after oral administration of Rhizoma Chuanxiong decoction by HPLC-ESI-MS/MS. J. Pharm. Biomed. Anal..

[25] Lao S.C., Li S.P., Kelvin K.W., Kan Li P., Wan J.B., Wang Y.T., Dong T.T., Tsim K.W. (2004). Identification and quantification of 13 components in *Angelica sinensis* (Danggui) by gas chromatography–mass spectrometry coupled with pressurized liquid extraction. Anal. Chim. Acta.

[26] Tanaka K., Tamura T., Fukuda S., Batkhuu J., Sanchir C., Komatsu K. (2008). Quality evaluation of Astragali Radix using a multivariate statistical approach. Phytochemistry.

[27] Napolitano A., Akay S., Maria A., Bedir E., Pizza C., Piacente S. (2013). An analytical approach based on ESI-MS, LC–MS and PCA for the quali-quantitative analysis of cycloartane derivatives in Astragalus spp.. J. Pharm. Biomed. Anal..

[28] Wen X.D., Qi L.W., Li P., Bao K.D., Yan X.W., Yi L., Li C.Y. (2008). Simultaneous determination of calycosin-7-O-*β*-d-glucoside, ononin, astragaloside IV, astragaloside I and ferulic acid in rat plasma after oral administration of Danggui Buxue Tang extract for their pharmacokinetic studies by liquid chromatography-mass spectrometry. J. Chromatogr. B.

[29] Xu F., Zhang Y., Xiao S., Lu X., Yang D.H., Yang X., Li C., Shang M., Tu P., Cai S. (2006). Absorption and metabolism of Astragali Radix decoction: *In silico*, *in vitro*, and a case study *in vivo*. Drug Metab. Dispos..

[30] Yu D., Duan Y., Bao Y., Wei C., An L. (2005). Isoflavonoids from Astragalus mongholicus protect PC12 cells from toxicity induced by l-glutamate. J. Ethnopharmacol..

