# Supplementary material for: Chemical Profile Analysis and Comparison of Two Versions of the Classic TCM Formula Danggui Buxue Tang by HPLC-DAD-ESI-IT-TOF-MSn
Source: Molecules. 2014 Apr 30;19(5):5650–73. doi: 10.3390/molecules19055650 (PMC6270708; doi:10.3390/molecules19055650)
Supplement: Supplementary file 1 [file molecules-19-05650-s001.pdf]

Supplementary File

1. **The HRMS data of 19 reference compounds** S2–S8
2. **The HRMS data of DBT1 boiling samples** S9–S35
3. **The HRMS data of DBT2 boiling samples** S36–S51
4. **The HRMS data of DBT1 urine samples** S52–S66
5. **The HRMS data of DBT2 urine samples** S67–S77
6. **The supplemental data of tables S1–S6** S78–S87

1. The HRMS data of 19 reference compounds

In PI model.

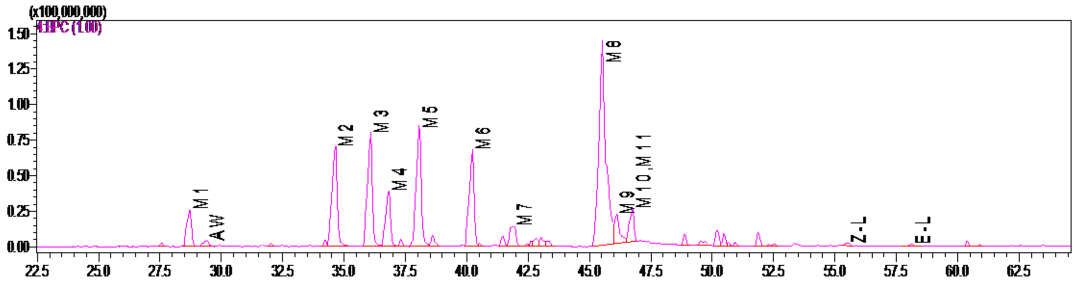

In NI model.

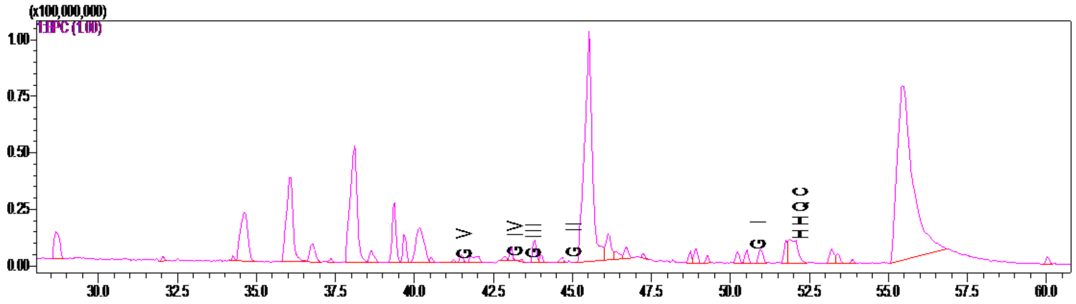

Reference compounds

D1Ferulic acid in NI mode

Event#: 4 MS(E-) Ret. Time : 29.162 Scan# : 2853

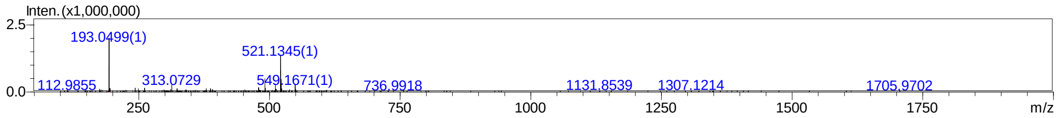

Event#: 5 MS/MS(E-) Ret. Time : 29.162 Scan# : 2854 Precursor : 193.0499 Cutoff : 53

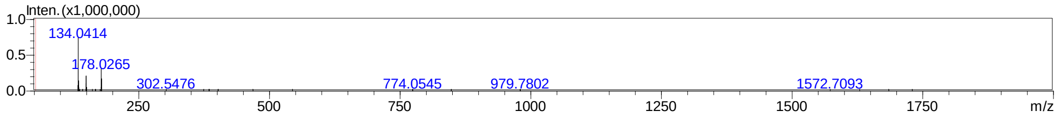

Rank	Score	Formula (M)	Ion	Meas. m/z	Pred. m/z	Diff (mDa)	Diff (ppm)	Iso Score	DBE
1	65.37	C10 H10 O4	[M-H]-	193.0501	193.0506	-0.5	-2.59	68.08	6.0

D2 *Z*-ligustilide in PI mode

Event#: 1 MS(E+) Ret. Time : 58.102 Scan# : 5719

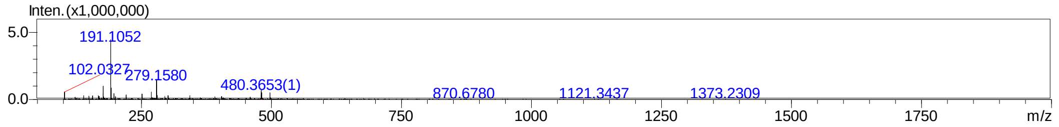

Rank	Score	Formula (M)	Ion	Meas. m/z	Pred. m/z	Diff (mDa)	Diff (ppm)	Iso Score	DBE
1	57.22	C12 H14 O2	[M+H]+	191.1066	191.1067	-0.1	-0.52	57.22	6.0

E-L

Event#: 1 MS(E+) Ret. Time : 62.323 Scan# : 6138

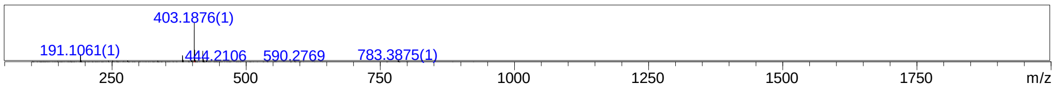

Event#: 2 MS/MS(E+) Ret. Time : 62.323 Scan# : 6139 Precursor : 403.1876 Cutoff : 111

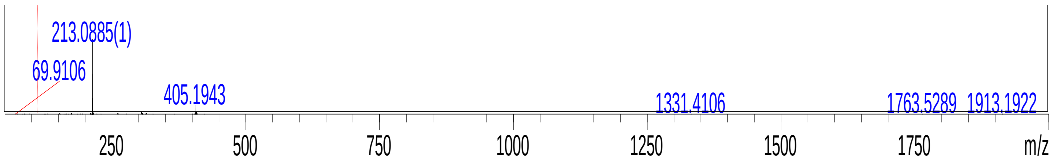

Rank	Score	Formula (M)	Ion	Meas. m/z	Pred. m/z	Diff (mDa)	Diff (ppm)	Iso Score	DBE
1	52.67	C12 H14 O2	[M+H]+	191.1061	191.1067	-0.6	-3.14	55.64	6.0

D3 Calycosin-7-O-*β*-D-glucopyranoside

Event#: 1 MS(E+) Ret. Time : 28.595 Scan# : 2794

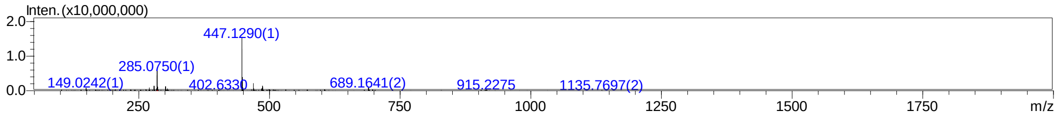

Event#: 4 MS(E-) Ret. Time : 28.595 Scan# : 2797

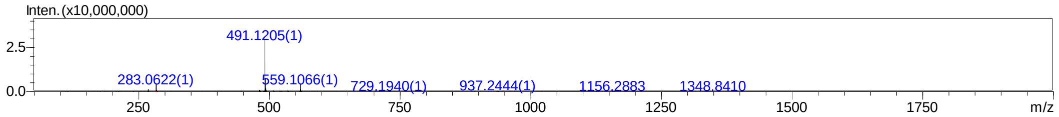

Rank	Score	Formula (M)	Ion	Meas. m/z	Pred. m/z	Diff (mDa)	Diff (ppm)	Iso Score	DBE
1	93.45	C22 H22 O10	[M+H]+	447.1290	447.1286	0.4	0.89	93.45	12.0

D4 ononin

Event#: 1 MS(E+) Ret. Time : 34.545 Scan# : 3382

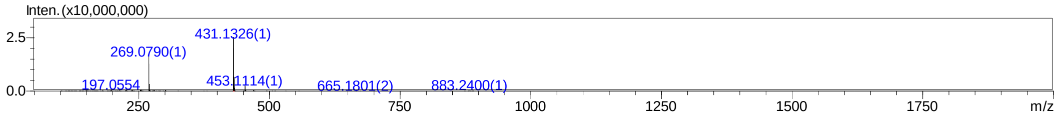

Event#: 2 MS/MS(E+) Ret. Time : 34.665 Scan# : 3395 Precursor : 269.0803 Cutoff : 74

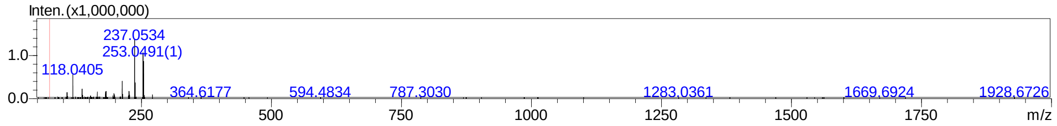

Event#: 4 MS(E-) Ret. Time : 34.545 Scan# : 3385

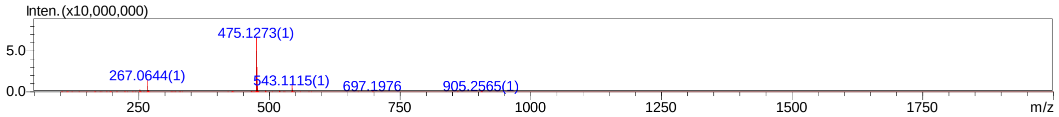

Rank	Score	Formula (M)	Ion	Meas. m/z	Pred. m/z	Diff (mDa)	Diff (ppm)	Iso Score	DBE
1	93.09	C22 H22 O9	[M+H]+	431.1326	431.1337	-1.1	-2.55	96.84	12.0

D5 astrapterocarpan-7-O-β-D-glucopyranoside

Event#: 1 MS(E+) Ret. Time : 36.038 Scan# : 3530

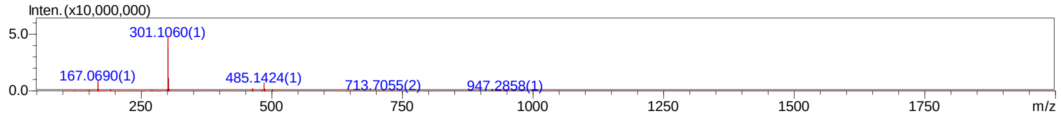

Event#: 4 MS(E-) Ret. Time : 36.038 Scan# : 3533

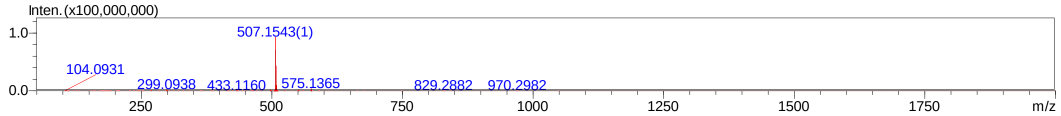

Rank	Score	Formula (M)	Ion	Meas. m/z	Pred. m/z	Diff (mDa)	Diff (ppm)	Iso Score	DBE
2	81.60	C23 H26 O10	[M+Na]+	485.1400	485.1418	-1.8	-3.71	87.53	11.0

D6 astraisoflavan-7-O-β-D-glucopyranoside

Event#: 1 MS(E+) Ret. Time : 36.785 Scan# : 3604

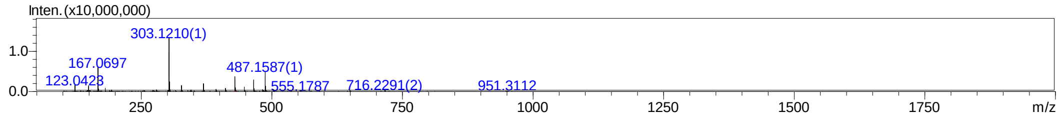

Event#: 4 MS(E-) Ret. Time : 36.785 Scan# : 3607

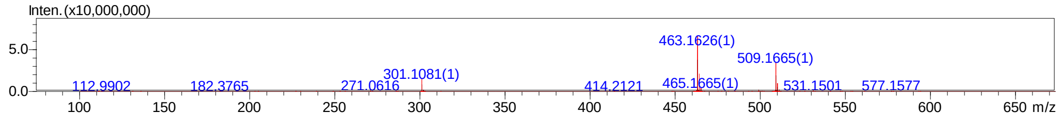

Rank	Score	Formula (M)	Ion	Meas. m/z	Pred. m/z	Diff (mDa)	Diff (ppm)	Iso Score	DBE
1	87.96	C23 H28 O10	[M+Na]+	487.1574	487.1575	-0.1	-0.21	87.96	10.0

D7 calycosin

Event#: 1 MS(E+) Ret. Time : 38.055 Scan# : 3730

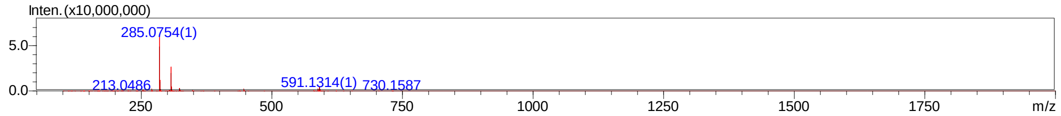

Event#: 2 MS/MS(E+) Ret. Time : 38.055 Scan# : 3731 Precursor : 285.0754 Cutoff : 78

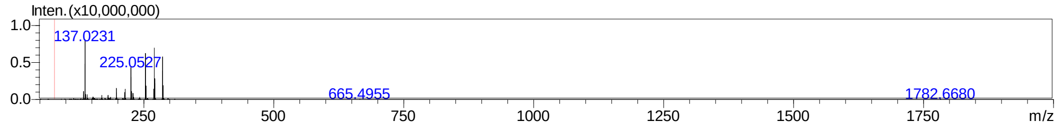

Event#: 4 MS(E-) Ret. Time : 38.055 Scan# : 3733

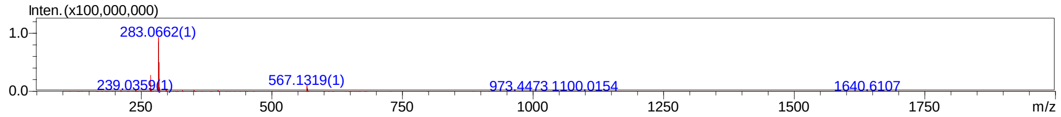

Rank	Score	Formula (M)	Ion	Meas. m/z	Pred. m/z	Diff (mDa)	Diff (ppm)	Iso Score	DBE
1	85.82	C16 H12 O5	[M+H]+	285.0754	285.0758	-0.4	-1.40	86.69	11.0

D8 6"-O-acetyl-ononin

Event#: 1 MS(E+) Ret. Time : 40.123 Scan# : 3935

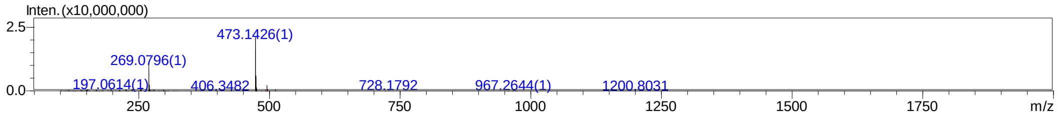

Event#: 4 MS(E-) Ret. Time : 40.123 Scan# : 3938

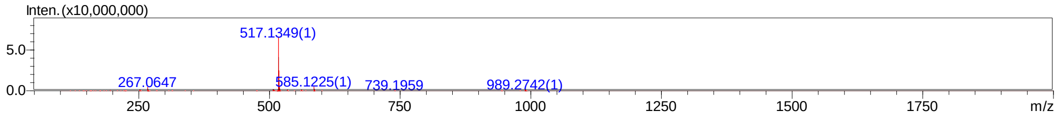

Rank	Score	Formula (M)	Ion	Meas. m/z	Pred. m/z	Diff (mDa)	Diff (ppm)	Iso Score	DBE
1	85.99	C24 H24 O10	[M+H]+	473.1432	473.1442	-1.0	-2.11	88.44	13.0

D9 6"-O-acetyl-astraisoflavan-7-O-*β*-D-glucopyranoside

Event#: 1 MS(E+) Ret. Time : 41.942 Scan# : 4116

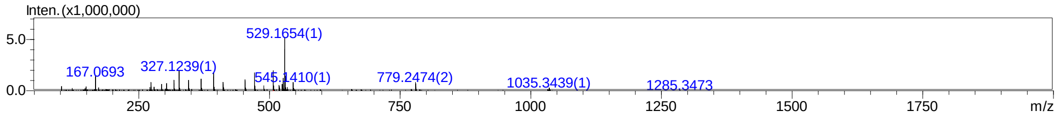

Event#: 4 MS(E-) Ret. Time : 41.942 Scan# : 4118

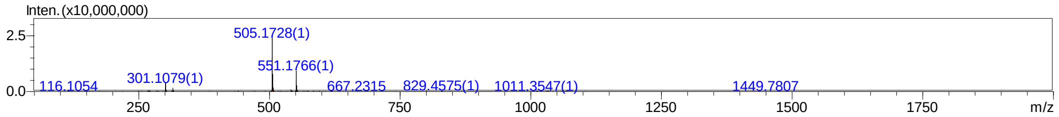

Rank	Score	Formula (M)	Ion	Meas. m/z	Pred. m/z	Diff (mDa)	Diff (ppm)	Iso Score	DBE
1	91.36	C25 H30 O11	[M+Na]+	529.1691	529.1680	1.1	2.08	93.90	11.0

D10 formonontein

Event#: 1 MS(E+) Ret. Time : 45.410 Scan# : 4459

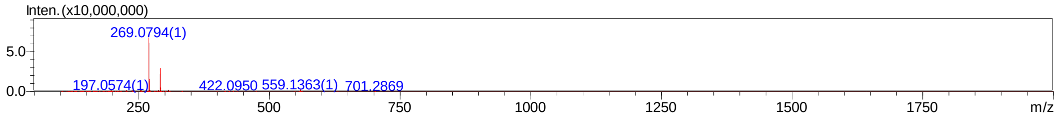

Event#: 4 MS(E-) Ret. Time : 45.410 Scan# : 4462

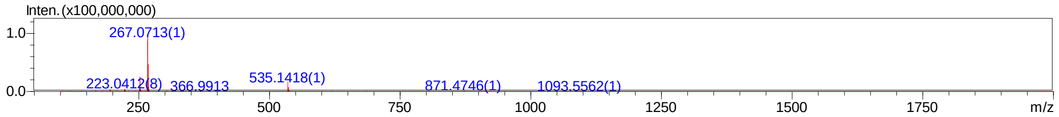

Rank	Score	Formula (M)	Ion	Meas. m/z	Pred. m/z	Diff (mDa)	Diff (ppm)	Iso Score	DBE
2	75.86	C16 H12 O4	[M+H]+	269.0795	269.0808	-1.3	-4.83	83.89	11.0

D11 astrapterocarpan

Event#: 1 MS(E+) Ret. Time : 46.062 Scan# : 4524

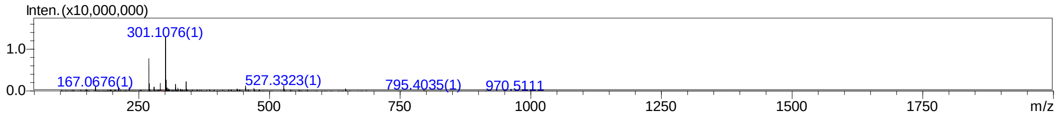

Rank	Score	Formula (M)	Ion	Meas. m/z	Pred. m/z	Diff (mDa)	Diff (ppm)	Iso Score	DBE
1	78.61	C17 H16 O5	[M+H]+	301.1076	301.1071	0.5	1.66	79.93	10.0

D12 astraisoflavan

Event#: 1 MS(E+) Ret. Time : 46.655 Scan# : 4583

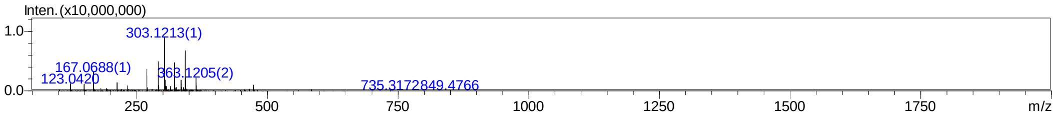

Rank	Score	Formula (M)	Ion	Meas. m/z	Pred. m/z	Diff (mDa)	Diff (ppm)	Iso Score	DBE
2	64.21	C17 H18 O5	[M+H]+	303.1213	303.1227	-1.4	-4.62	70.60	9.0

ZG-V

Event#: 4 MS(E-) Ret. Time : 41.110 Scan# : 4036

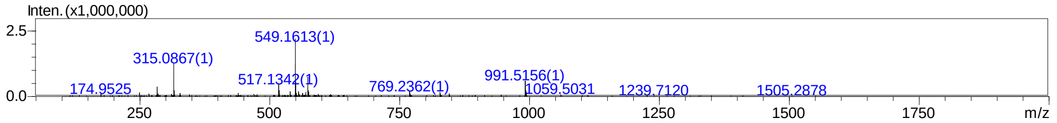

Event#: 5 MS/MS(E-) Ret. Time : 41.110 Scan# : 4037 Precursor : 991.5156 Cutoff : 274

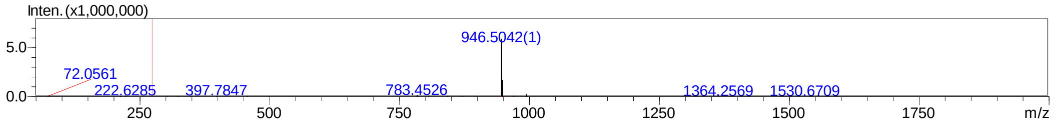

Rank	Score	Formula (M)	Ion	Meas. m/z	Pred. m/z	Diff (mDa)	Diff (ppm)	Iso Score	DBE
**3**	**36.36**	**C47 H78 O19**	**[M+HCOO]-**	**991.5156**	**991.5119**	**3.7**	**3.73**	**39.03**	**9.0**

C6H10O5

ZG-IV

Event#: 4 MS(E-) Ret. Time : 42.775 Scan# : 4201

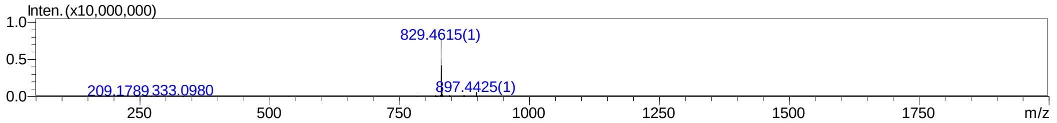

Event#: 5 MS/MS(E-) Ret. Time : 42.775 Scan# : 4202 Precursor : 829.7059 Cutoff : 229

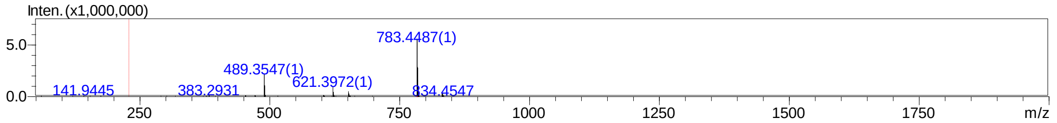

Rank	Score	Formula (M)	Ion	Meas. m/z	Pred. m/z	Diff (mDa)	Diff (ppm)	Iso Score	DBE
1	90.31	C42 H70 O16	[M-H]-	829.4615	829.4591	2.4	2.89	94.78	8.0
**2**	**90.31**	**C41 H68 O14**	**[M+HCOO]-**	**829.4615**	**829.4591**	**2.4**	**2.89**	**94.78**	**8.0**

C2H2O

GIII

Event#: 4 MS(E-) Ret. Time : 43.410 Scan# : 4264

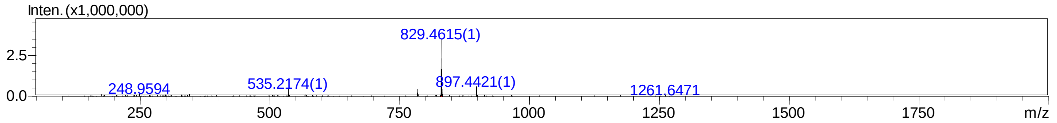

Event#: 5 MS/MS(E-) Ret. Time : 43.410 Scan# : 4265 Precursor : 829.4617 Cutoff : 229

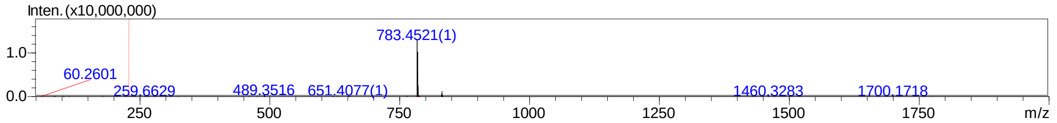

Event#: 6 MS3(E-) Ret. Time : 43.410 Scan# : 4266 Precursor : 783.4520 Cutoff : 216

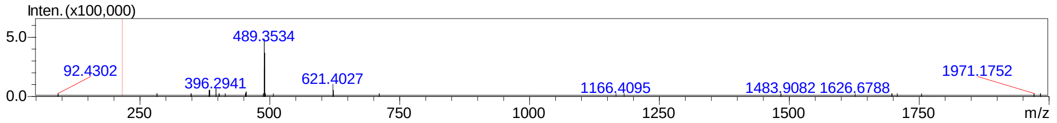

Rank	Score	Formula (M)	Ion	Meas. m/z	Pred. m/z	Diff (mDa)	Diff (ppm)	Iso Score	DBE
1	59.30	C42 H70 O16	[M-H]-	829.4585	829.4591	-0.6	-0.72	59.30	8.0
2	59.30	C41 H68 O14	[M+HCOO]-	829.4585	829.4591	-0.6	-0.72	59.30	8.0

ZG-II

Event#: 1 MS(E+) Ret. Time : 44.628 Scan# : 4382

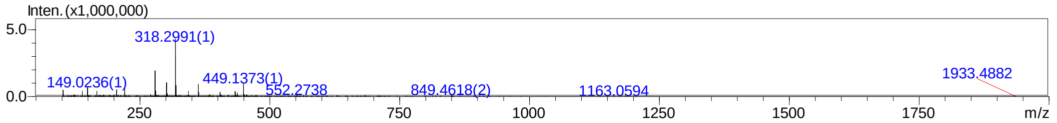

Event#: 4 MS(E-) Ret. Time : 44.628 Scan# : 4385

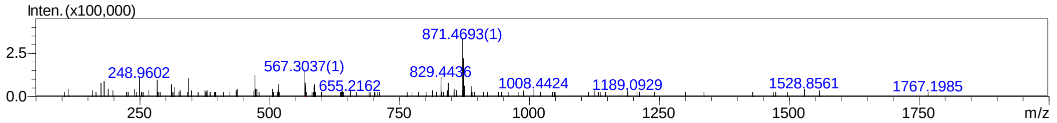

Rank	Score	Formula (M)	Ion	Meas. m/z	Pred. m/z	Diff (mDa)	Diff (ppm)	Iso Score	DBE
**5**	**44.80**	**C43 H70 O15**	**[M+HCOO]-**	**871.4693**	**871.4697**	**-0.4**	**-0.46**	**44.80**	**9.0**

ZG--I

Event#: 4 MS(E-) Ret. Time : 50.525 Scan# : 4970

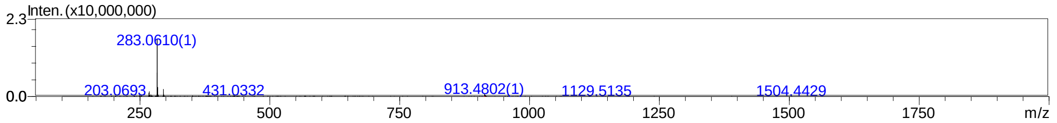

Event#: 5 MS/MS(E-) Ret. Time : 50.525 Scan# : 4971 Precursor : 913.4805 Cutoff : 252

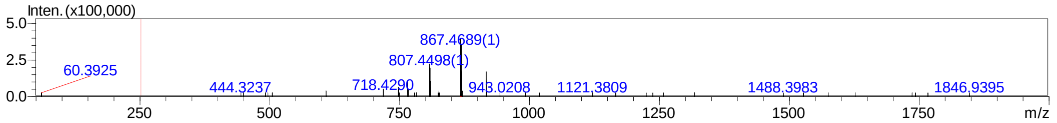

Rank	Score	Formula (M)	Ion	Meas. m/z	Pred. m/z	Diff (mDa)	Diff (ppm)	Iso Score	DBE
**1**	**46.02**	**C45 H72 O16**	**[M+HCOO]-**	**913.4802**	**913.4802**	**0.0**	**0.00**	**46.02**	**10.0**

C2H2O

HHQC

Event#: 1 MS(E+) Ret. Time : 51.855 Scan# : 5099

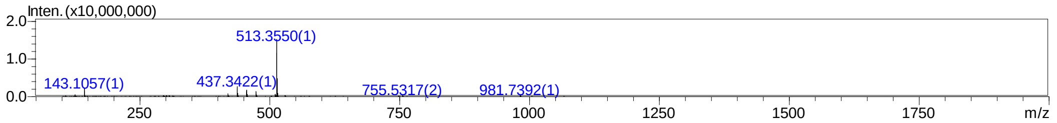

Event#: 4 MS(E-) Ret. Time : 52.482 Scan# : 5164

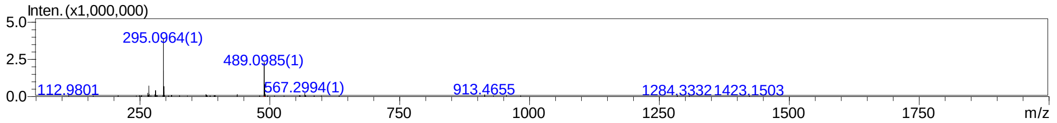

Rank	Score	Formula (M)	Ion	Meas. m/z	Pred. m/z	Diff (mDa)	Diff (ppm)	Iso Score	DBE
**1**	**94.74**	**C30 H50 O5**	**[M+Na]+**	**513.3550**	**513.3550**	**0.0**	**0.00**	**94.74**	**6.0**

2. The HRMS data of DBT1 boiling samples

In PI and NI model.

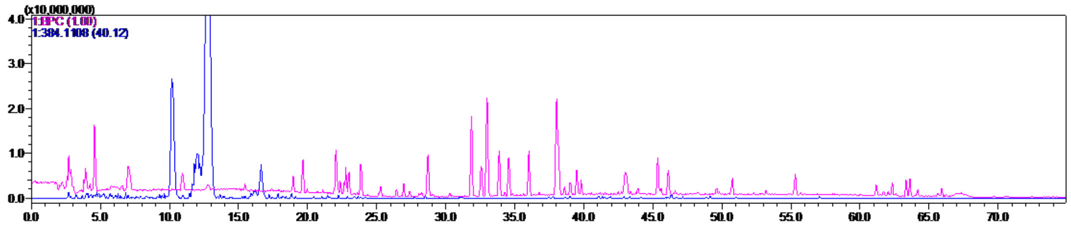

X24+A5+Z11+Y23=69

C1 A1

Event#: 1 MS(E+) Ret. Time : 2.280 -> 2.572 Scan# : 224 -> 253

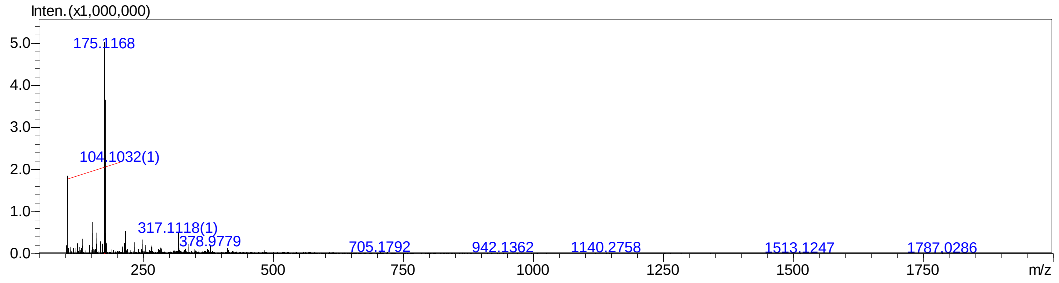

MS/MS(E+) Ret. Time : 2.280 -> 2.572 Scan# : 225 -> 254 Precursor : 175.1180 Cutoff : 48

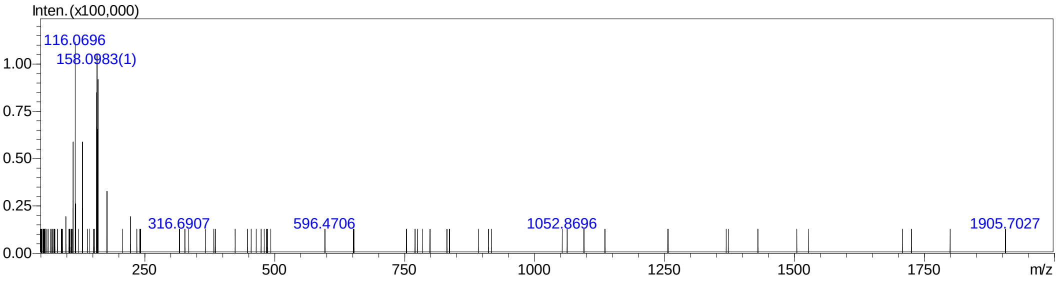

Rank	Score	Formula (M)	Ion	Meas. m/z	Pred. m/z	Diff (mDa)	Diff (ppm)	Iso Score	DBE
1	18.01	C6 H14 N4 O2	[M+H]+	175.1168	175.1190	-2.2	-12.56	54.29	2.0

C2 A4

Event#: 4 MS(E-) Ret. Time : 2.692 Scan# : 267

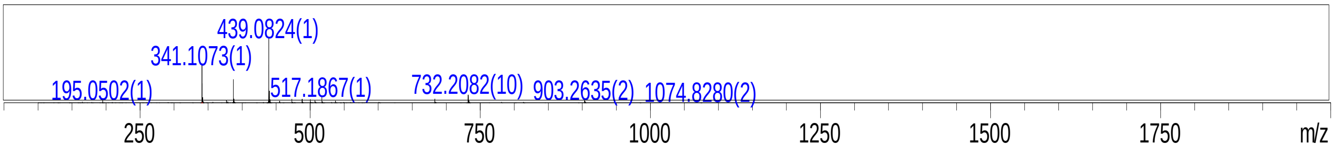

Rank	Score	Formula (M)	Ion	Meas. m/z	Pred. m/z	Diff (mDa)	Diff (ppm)	Iso Score	DBE
1	52.45	C6 H12 O7	[M-H]-	195.0502	195.0510	-0.8	-4.10	56.86	1.0

C3 T1

Event#: 1 MS(E+) Ret. Time : 3.095 Scan# : 305

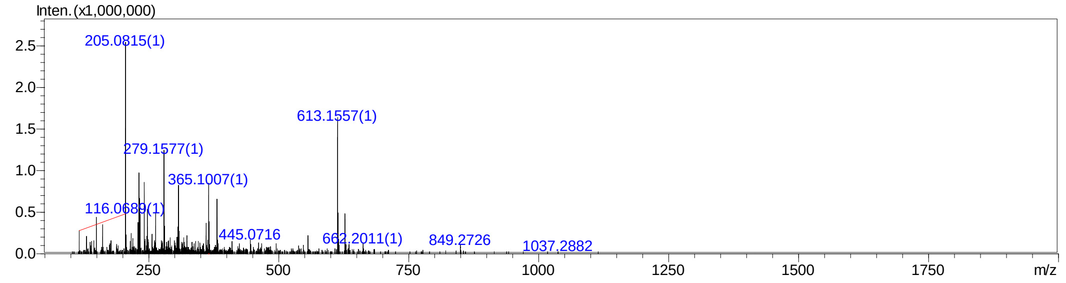

MS/MS(E+) Ret. Time : 3.095 Scan# : 306 Precursor : 365.1008 Cutoff : 101

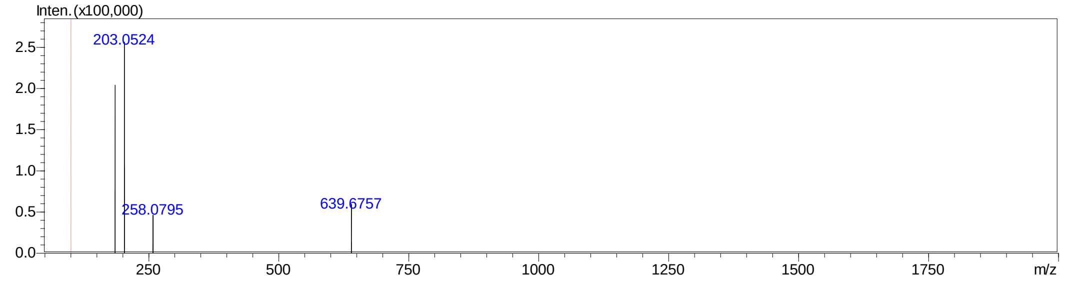

Rank	Score	Formula (M)	Ion	Meas. m/z	Pred. m/z	Diff (mDa)	Diff (ppm)	Iso Score	DBE
1	47.34	C21 H16 O6	[M+H]+	365.1007	365.1020	-1.3	-3.56	50.58	14.0
2	46.62	C19 H18 O6	[M+Na]+	365.1007	365.0996	1.1	3.01	49.09	11.0
3	17.33	C28 H12 O	[M+H]+	365.1007	365.0961	4.6	12.60	52.40	23.0
**4**	**13.68**	**C12 H22 O11**	**[M+Na]+**	**365.1007**	**365.1054**	**-4.7**	**-12.87**	**42.30**	**2.0**

C4 A2 **Citric acid**

Event#: 4 MS(E-) Ret. Time : 3.682 -> 4.437 Scan# : 365 -> 440

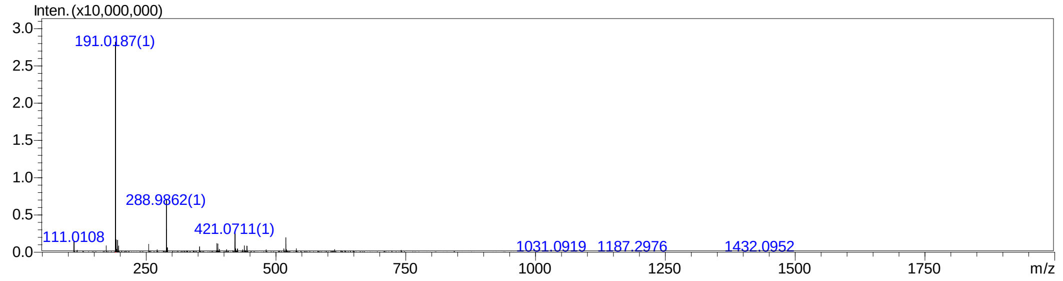

MS/MS(E-) Ret. Time : 3.682 -> 4.437 Scan# : 366 -> 441 Precursor : 191.0181 Cutoff : 52

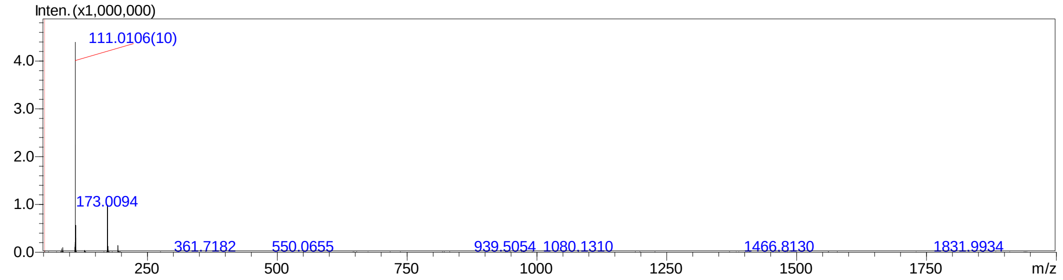

Rank	Score	Formula (M)	Ion	Meas. m/z	Pred. m/z	Diff (mDa)	Diff (ppm)	Iso Score	DBE
**1**	**73.92**	**C6 H8 O7**	**[M-H]-**	**191.0187**	**191.0197**	**-1.0**	**-5.24**	**84.39**	**3.0**

C9 A3

Event#: 1 MS(E+) Ret. Time : 10.773 -> 11.005 Scan# : 1062 -> 1085

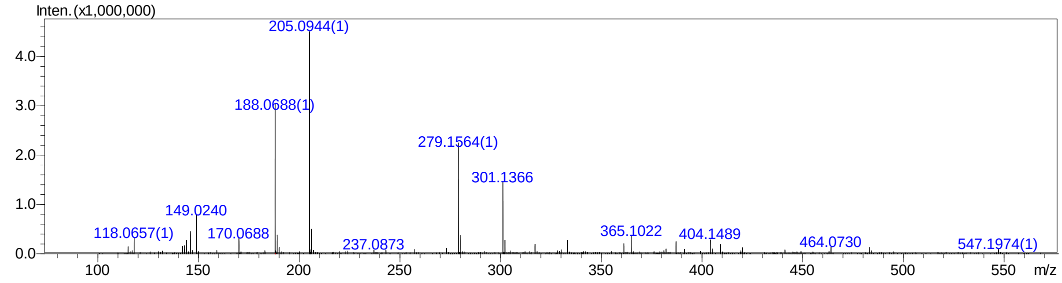

MS/MS(E+) Ret. Time : 10.773 -> 11.005 Scan# : 1063 -> 1086 Precursor : 188.0702 Cutoff : 52

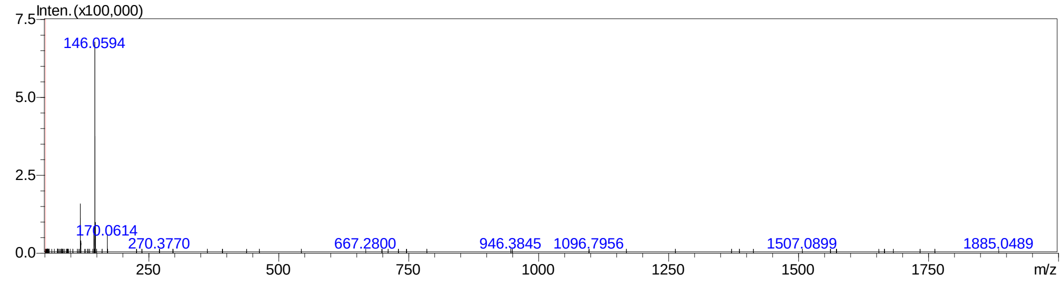

Rank	Score	Formula (M)	Ion	Meas. m/z	Pred. m/z	Diff (mDa)	Diff (ppm)	Iso Score	DBE
1	73.75	C9 H11 N O2	[M+Na]+	188.0688	188.0682	0.6	3.19	78.02	5.0

C18 Y1

Event#: 4 MS(E-) Ret. Time : 23.590 Scan# : 2321

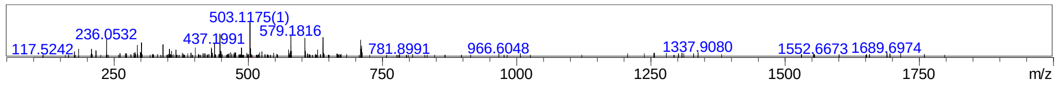

Event#: 5 MS/MS(E-) Ret. Time : 23.590 Scan# : 2322 Precursor : 503.1172 Cutoff : 139

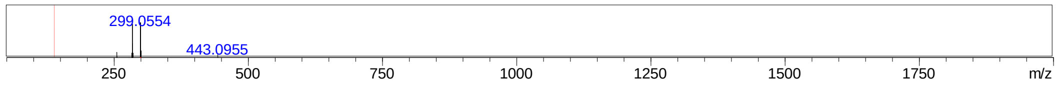

Rank	Score	Formula (M)	Ion	Meas. m/z	Pred. m/z	Diff (mDa)	Diff (ppm)	Iso Score	DBE
1	42.44	C24 H24 O12	[M-H]-	503.1175	503.1195	-2.0	-3.98	45.85	13.0

Rank	Score	Formula (M)	Ion	Meas. m/z	Pred. m/z	Diff (mDa)	Diff (ppm)	Iso Score	DBE
1	51.24	C16 H12 O6	[M-H]-	299.0554	299.0561	-0.7	-2.34	53.02	11.0

C16H12O5

C23 Y2 calycosin-glucoside

Event#: 1 MS(E+) Ret. Time : 28.830 Scan# : 2834

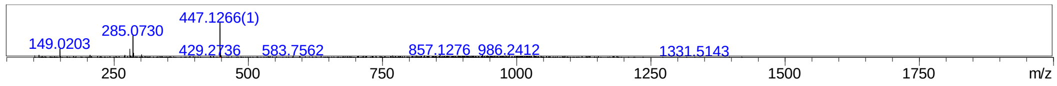

Rank	Score	Formula (M)	Ion	Meas. m/z	Pred. m/z	Diff (mDa)	Diff (ppm)	Iso Score	DBE
**2**	**91.01**	**C22 H22 O10**	**[M+H]+**	**447.1266**	**447.1286**	**-2.0**	**-4.47**	**99.66**	**12.0**

C24 Y3

Event#: 1 MS(E+) Ret. Time : 29.233 Scan# : 2874

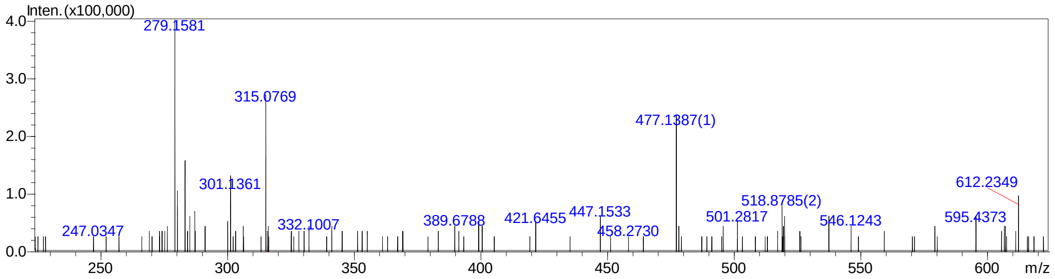

MS/MS(E+) Ret. Time : 29.233 Scan# : 2875 Precursor : 315.0768 Cutoff : 87

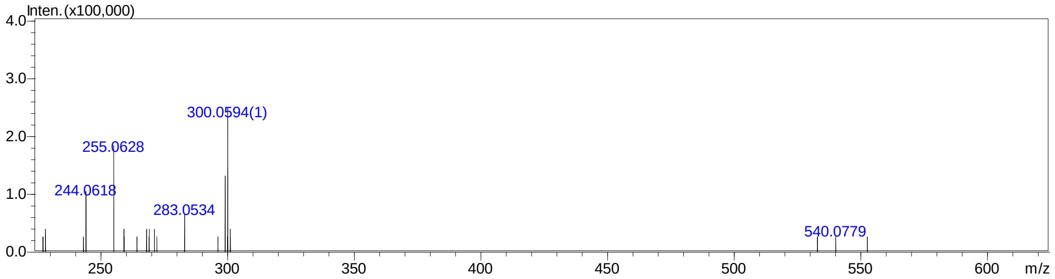

Rank Score Formula (M) Ion Meas. m/z Pred. m/z Diff (mDa) Diff (ppm) Iso Score DBE

1 41.50 C23 H24 O11 [M+H]+ 477.1387 477.1391 -0.4 -0.84 41.50 12.0

Rank Score Formula (M) Ion Meas. m/z Pred. m/z Diff (mDa) Diff (ppm) Iso Score DBE

1 31.05 C17 H14 O6 [M+H]+ 315.0872 315.0863 0.9 2.86 32.57 11.0

C6H10O5 C17H14O6 （16H12O5）CH2O

C26 Y4

Event#: 4 MS(E-) Ret. Time : 30.653 Scan# : 3017

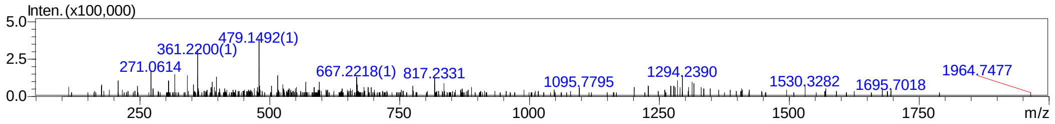

Event#: 5 MS/MS(E-) Ret. Time : 30.653 Scan# : 3018 Precursor : 479.1494 Cutoff : 132

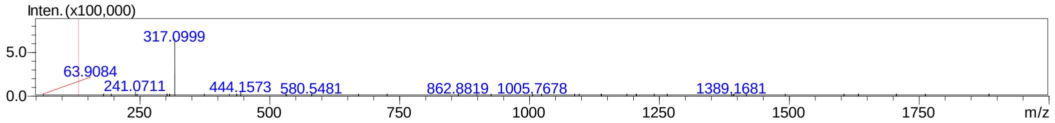

Rank Score Formula (M) Ion Meas. m/z Pred. m/z Diff (mDa) Diff (ppm) Iso Score DBE

1 28.90 C30 H24 O6 [M-H]- 479.1492 479.1500 -0.8 -1.67 29.39 19.0

Rank Score Formula (M) Ion Meas. m/z Pred. m/z Diff (mDa) Diff (ppm) Iso Score DBE

1 0.00 C24 H14 O [M-H]- 317.0999 317.0972 2.7 8.51 0.00 18.0

C6H10O5

C27 Y5

Event#: 4 MS(E-) Ret. Time : 30.773 Scan# : 3029

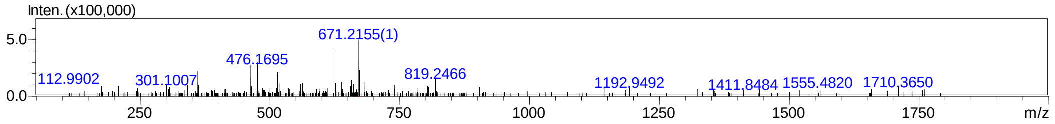

Event#: 5 MS/MS(E-) Ret. Time : 30.773 Scan# : 3030 Precursor : 671.2156 Cutoff : 185

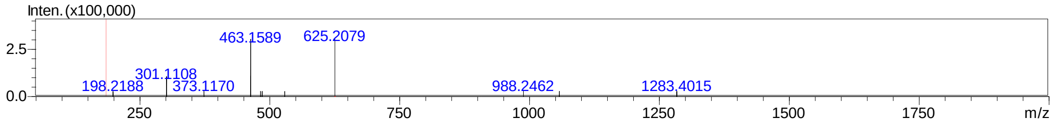

Event#: 6 MS3(E-) Ret. Time : 30.773 Scan# : 3031 Precursor : 625.2078 Cutoff : 173

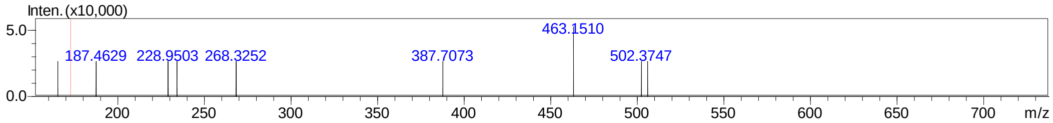

Rank Score Formula (M) Ion Meas. m/z Pred. m/z Diff (mDa) Diff (ppm) Iso Score DBE

4 52.07 C29 H38 O15 [M+HCOO]- 671.2155 671.2193 -3.8 -5.66 62.43 11.0

Rank Score Formula (M) Ion Meas. m/z Pred. m/z Diff (mDa) Diff (ppm) Iso Score DBE

5 0.00 C29 H38 O15 [M-H]- 625.2079 625.2138 -5.9 -9.44 0.00 11.0

Rank Score Formula (M) Ion Meas. m/z Pred. m/z Diff (mDa) Diff (ppm) Iso Score DBE

1 0.00 C23 H28 O10 [M-H]- 463.1589 463.1610 -2.1 -4.53 0.00 10.0

Rank Score Formula (M) Ion Meas. m/z Pred. m/z Diff (mDa) Diff (ppm) Iso Score DBE

1 0.00 C17 H18 O5 [M-H]- 301.1108 301.1081 2.7 8.97 0.00 9.0

C29 Y6

Event#: 4 MS(E-) Ret. Time : 31.348 Scan# : 3086

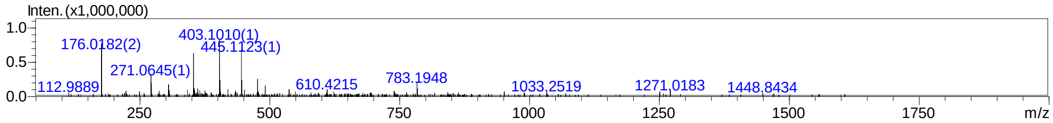

Event#: 5 MS/MS(E-) Ret. Time : 31.348 Scan# : 3087 Precursor : 445.1123 Cutoff : 123

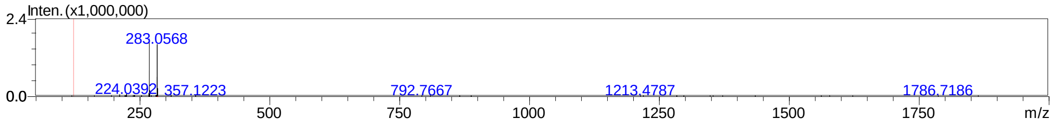

Rank Score Formula (M) Ion Meas. m/z Pred. m/z Diff (mDa) Diff (ppm) Iso Score DBE

1 67.00 C22 H22 O10 [M-H]- 445.1123 445.1140 -1.7 -3.82 72.08 12.0

Rank Score Formula (M) Ion Meas. m/z Pred. m/z Diff (mDa) Diff (ppm) Iso Score DBE

2 9.46 C16 H12 O5 [M-H]- 283.0568 283.0612 -4.4 -15.54 37.49 11.0

C30 Y7 M=300

Event#: 1 MS(E+) Ret. Time : 32.078 Scan# : 3155

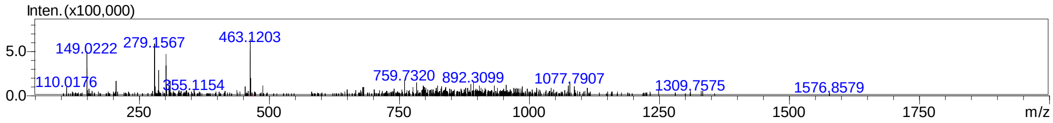

Event#: 2 MS/MS(E+) Ret. Time : 32.078 Scan# : 3156 Precursor : 463.1203 Cutoff : 128

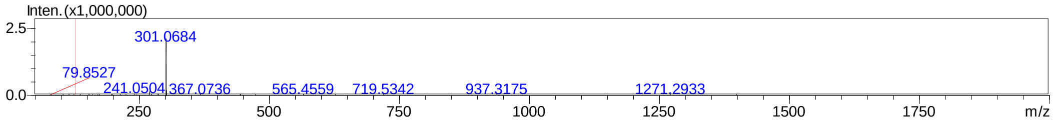

Event#: 4 MS(E-) Ret. Time : 32.078 Scan# : 3158

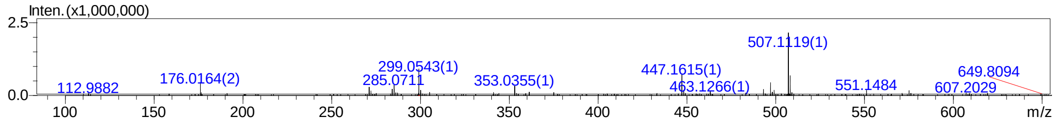

Rank	Score	Formula (M)	Ion	Meas. m/z	Pred. m/z	Diff (mDa)	Diff (ppm)	Iso Score	DBE
**2**	**40.84**	**C22 H22 O11**	**[M+H]+**	**463.1203**	**463.1235**	**-3.2**	**-6.91**	**57.61**	**12.0**

Rank	Score	Formula (M)	Ion	Meas. m/z	Pred. m/z	Diff (mDa)	Diff (ppm)	Iso Score	DBE
2	30.14	C16 H12 O6	[M+H]+	301.0684	301.0707	-2.3	-7.64	47.39	11.0

C6H10O5

C31 Y8 532=284+ 248==162+86==

Event#: 1 MS(E+) Ret. Time : 32.662 Scan# : 3213

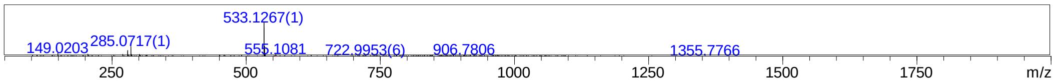

Event#: 2 MS/MS(E+) Ret. Time : 32.542 Scan# : 3202 Precursor : 533.1259 Cutoff : 147

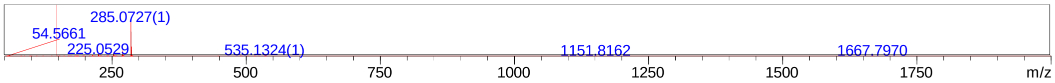

Rank	Score	Formula (M)	Ion	Meas. m/z	Pred. m/z	Diff (mDa)	Diff (ppm)	Iso Score	DBE
1	82.95	C23 H26 O13	[M+Na]+	533.1267	533.1266	0.1	0.19	82.95	11.0
2	76.61	C25 H24 O13	[M+H]+	533.1267	533.1290	-2.3	-4.31	83.52	14.0

Rank	Score	Formula (M)	Ion	Meas. m/z	Pred. m/z	Diff (mDa)	Diff (ppm)	Iso Score	DBE
3	18.81	C16 H12 O5	[M+H]+	285.0717	285.0758	-4.1	-14.38	66.43	11.0

C9H12O8 ---H2O C6H10O5 C3H2O3-

C32 Y9

Event#: 1 MS(E+) Ret. Time : 34.207 Scan# : 3366

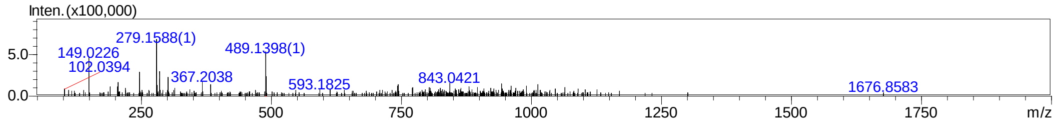

Event#: 2 MS/MS(E+) Ret. Time : 34.207 Scan# : 3367 Precursor : 489.1398 Cutoff : 135

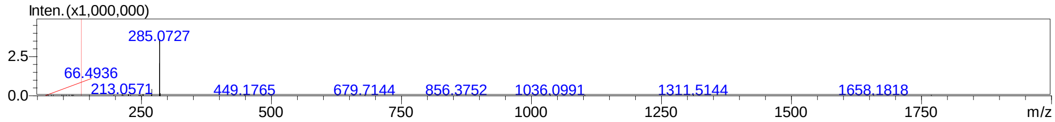

1	49.97	C24 H24 O11	[M+H]+	489.1398	489.1391	0.7	1.43	50.51	13.0

C16H12O5 C8H16O7+

C33 Y10 ononin

Event#: 1 MS(E+) Ret. Time : 34.550 Scan# : 3400

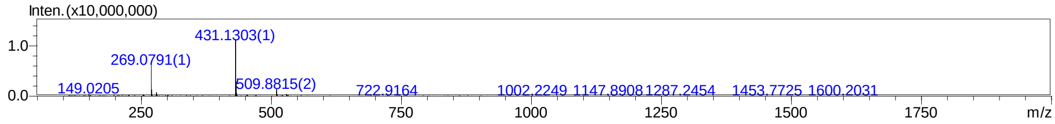

Event#: 4 MS(E-) Ret. Time : 34.550 Scan# : 3403

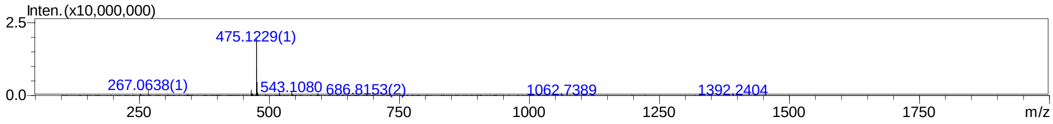

Rank	Score	Formula (M)	Ion	Meas. m/z	Pred. m/z	Diff (mDa)	Diff (ppm)	Iso Score	DBE
1	66.82	C29 H18 O4	[M+H]+	431.1303	431.1278	2.5	5.80	81.48	21.0
**2**	**56.98**	**C22 H22 O9**	**[M+H]+**	**431.1303**	**431.1337**	**-3.4**	**-7.89**	**93.25**	**12.0**

Rank	Score	Formula (M)	Ion	Meas. m/z	Pred. m/z	Diff (mDa)	Diff (ppm)	Iso Score	DBE
2	75.50	C22 H22 O9	[M+HCOO]-	475.1229	475.1246	-1.7	-3.58	80.71	12.0

C34 Y11 （286+204）

Event#: 4 MS(E-) Ret. Time : 35.348 Scan# : 3482

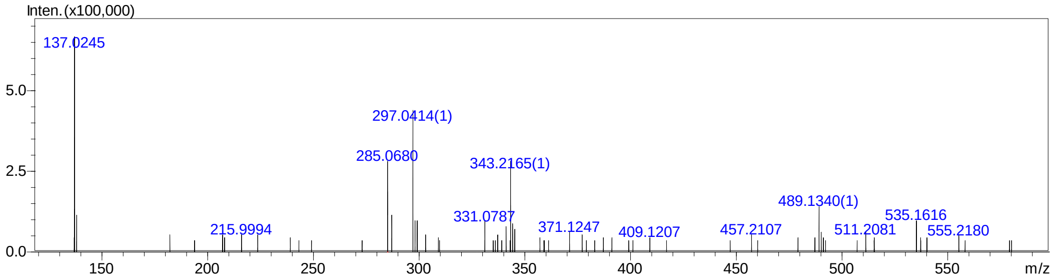

MS/MS(E-) Ret. Time : 35.348 Scan# : 3483 Precursor : 285.0680 Cutoff : 78

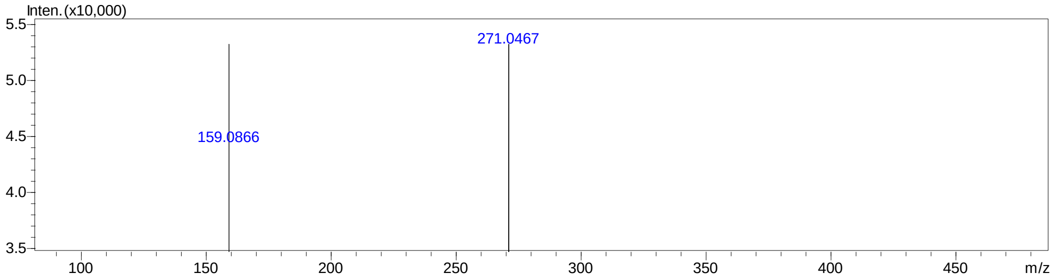

Rank	Score	Formula (M)	Ion	Meas. m/z	Pred. m/z	Diff (mDa)	Diff (ppm)	Iso Score	DBE
4	7.73	C24 H26 O11	[M-H]-	489.1340	489.1402	-6.2	-12.68	23.53	12.0

C8H16O7+

C16H12O5

C35 Y12

Event#: 1 MS(E+) Ret. Time : 35.580 Scan# : 3502

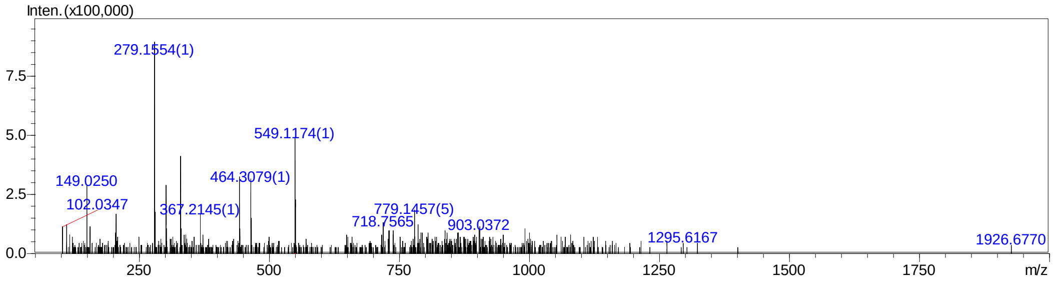

MS/MS(E+) Ret. Time : 35.580 Scan# : 3503 Precursor : 549.1174 Cutoff : 151

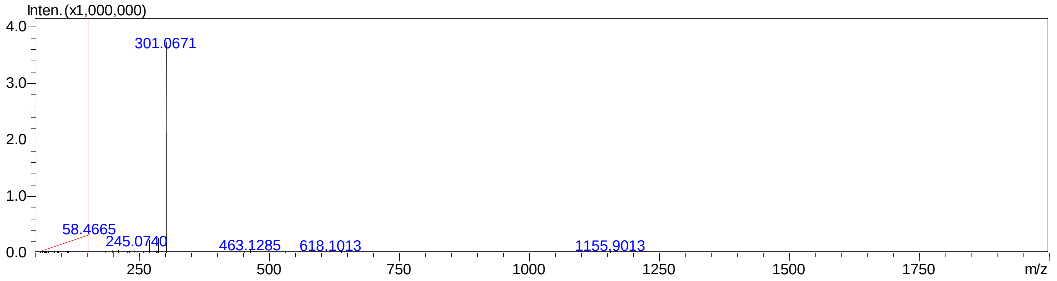

Rank	Score	Formula (M)	Ion	Meas. m/z	Pred. m/z	Diff (mDa)	Diff (ppm)	Iso Score	DBE
4	20.29	C25 H24 O14	[M+H]+	549.1174	549.1239	-6.5	-11.84	57.82	14.0

Rank	Score	Formula (M)	Ion	Meas. m/z	Pred. m/z	Diff (mDa)	Diff (ppm)	Iso Score	DBE
3	17.81	C16 H12 O6	[M+H]+	301.0671	301.0707	-3.6	-11.96	51.21	11.0

C36 Y13

Event#: 1 MS(E+) Ret. Time : 36.027 Scan# : 3546

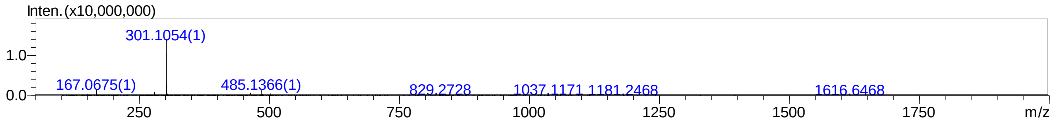

Event#: 2 MS/MS(E+) Ret. Time : 36.027 Scan# : 3547 Precursor : 463.1591 Cutoff : 128

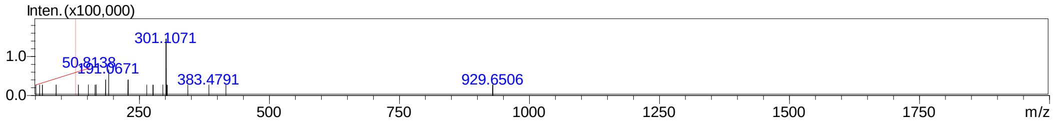

Event#: 4 MS(E-) Ret. Time : 36.027 Scan# : 3549

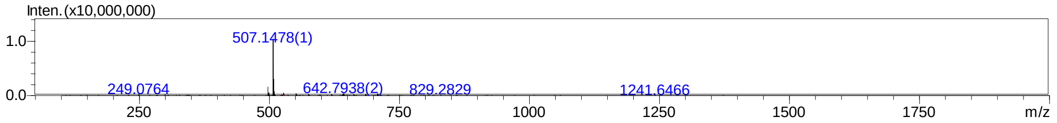

Rank	Score	Formula (M)	Ion	Meas. m/z	Pred. m/z	Diff (mDa)	Diff (ppm)	Iso Score	DBE
1	35.85	C23 H26 O10	[M+H]+	463.1615	463.1599	1.6	3.45	38.19	11.0

Rank	Score	Formula (M)	Ion	Meas. m/z	Pred. m/z	Diff (mDa)	Diff (ppm)	Iso Score	DBE
1	27.23	C17 H16 O5	[M+H]+	301.1071	301.1071	0.0	0.00	27.23	10.0

C6H10O5---H2O

C37 Y14

Event#: 1 MS(E+) Ret. Time : 36.773 Scan# : 3620

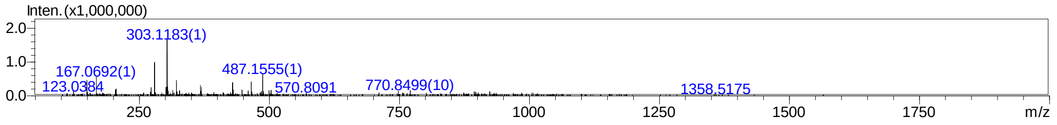

Event#: 2 MS/MS(E+) Ret. Time : 36.773 Scan# : 3621 Precursor : 303.1185 Cutoff : 83

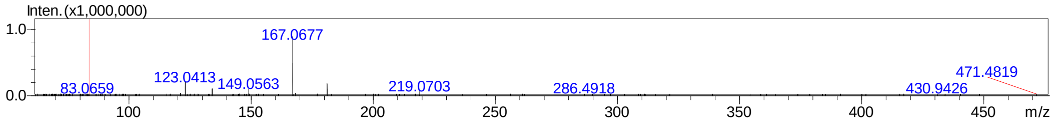

Event#: 4 MS(E-) Ret. Time : 36.773 Scan# : 3623

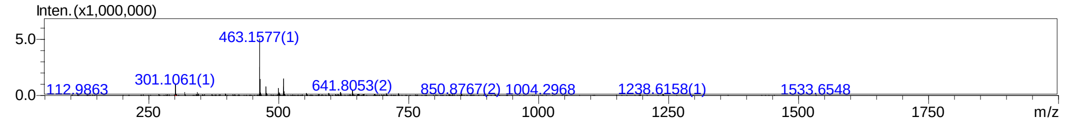

Event#: 5 MS/MS(E-) Ret. Time : 36.773 Scan# : 3624 Precursor : 301.1061 Cutoff : 83

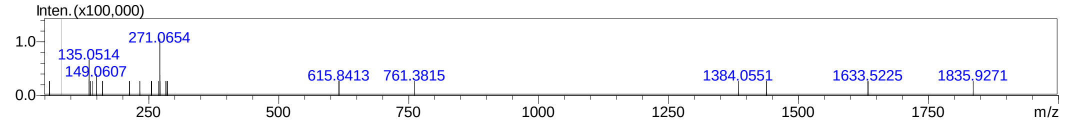

Rank	Score	Formula (M)	Ion	Meas. m/z	Pred. m/z	Diff (mDa)	Diff (ppm)	Iso Score	DBE
1	65.33	C23 H28 O10	[M-H]-	463.1577	463.1610	-3.3	-7.13	95.10	10.0

C38 Y15 calycosin（DZ）

Event#: 1 MS(E+) Ret. Time : 38.035 Scan# : 3745

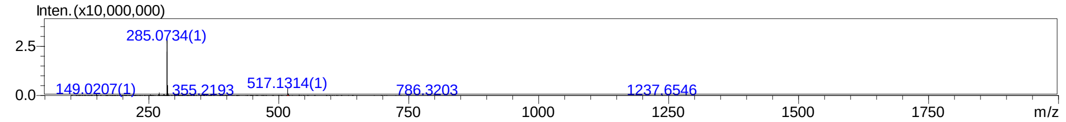

Event#: 4 MS(E-) Ret. Time : 38.035 Scan# : 3748

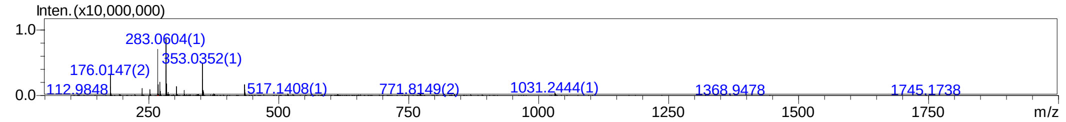

Event#: 2 MS/MS(E+) Ret. Time : 37.915 Scan# : 3734 Precursor : 285.0744 Cutoff : 78

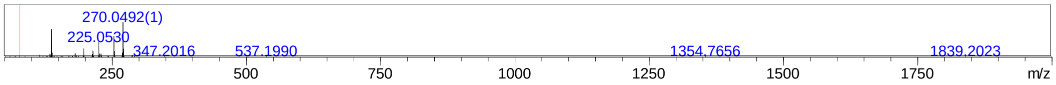

Rank	Score	Formula (M)	Ion	Meas. m/z	Pred. m/z	Diff (mDa)	Diff (ppm)	Iso Score	DBE
1	82.64	C16 H12 O5	[M+H]+	285.0744	285.0758	-1.4	-4.91	91.59	11.0

C38 C43 Y16 517=269+ 248==162+86==

Event#: 1 MS(E+) Ret. Time : 38.035 -> 39.452 Scan# : 3745 -> 3885

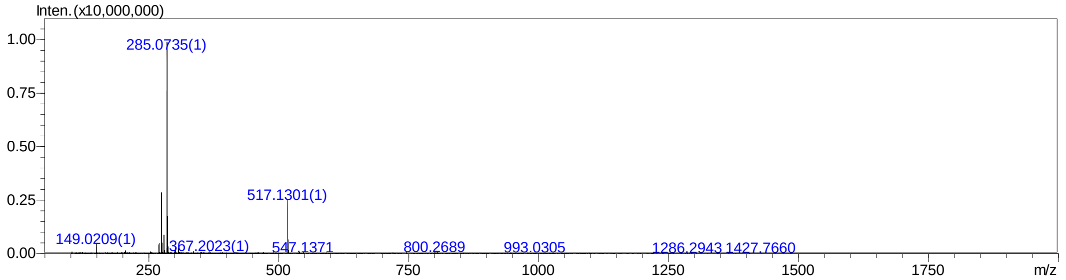

MS/MS(E+) Ret. Time : 38.035 -> 39.452 Scan# : 3746 -> 3886 Precursor : 517.1313 Cutoff : 143

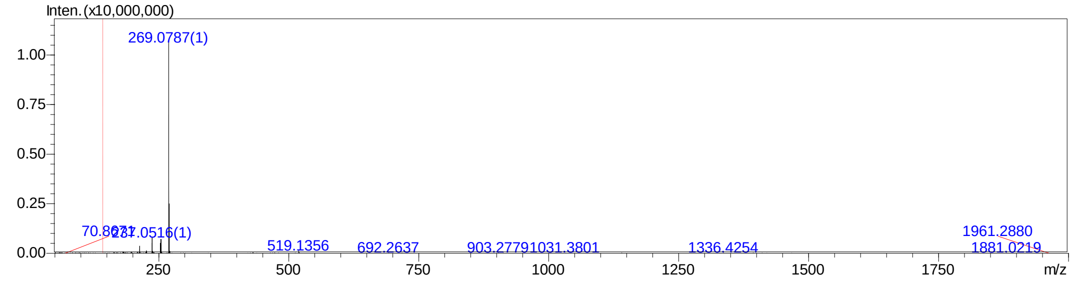

Rank	Score	Formula (M)	Ion	Meas. m/z	Pred. m/z	Diff (mDa)	Diff (ppm)	Iso Score	DBE
1	95.25	C23 H26 O12	[M+Na]+	517.1301	517.1316	-1.5	-2.90	100.00	11.0
**3**	**62.40**	**C25 H24 O12**	**[M+H]+**	**517.1301**	**517.1341**	**-4.0**	**-7.73**	**99.53**	**14.0**

Rank	Score	Formula (M)	Ion	Meas. m/z	Pred. m/z	Diff (mDa)	Diff (ppm)	Iso Score	DBE
1	72.73	C14 H14 O4	[M+Na]+	269.0787	269.0784	0.3	1.11	72.93	8.0
2	46.45	C16 H12 O4	[M+H]+	269.0787	269.0808	-2.1	-7.80	74.91	11.0

C9H12O8 ---H2O=== C9H14O9

C39 Y17

Event#: 1 MS(E+) Ret. Time : 38.653 Scan# : 3806

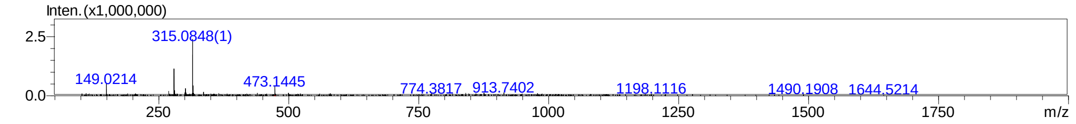

Rank	Score	Formula (M)	Ion	Meas. m/z	Pred. m/z	Diff (mDa)	Diff (ppm)	Iso Score	DBE
1	72.52	C15 H16 O6	[M+Na]+	315.0844	315.0839	0.5	1.59	73.60	8.0
2	63.21	C17 H14 O6	[M+H]+	315.0844	315.0863	-1.9	-6.03	79.31	11.0

（C16 H12 O5）CH2O

C40 Y18

Event#: 1 MS(E+) Ret. Time : 38.653 Scan# : 3806

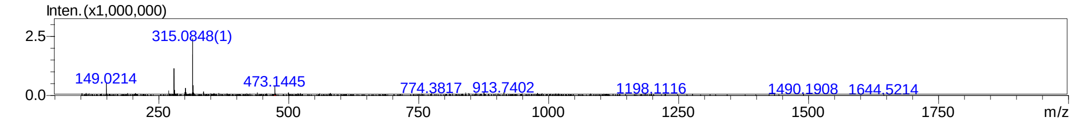

Event#: 2 MS/MS(E+) Ret. Time : 38.653 Scan# : 3807 Precursor : 473.1445 Cutoff : 130

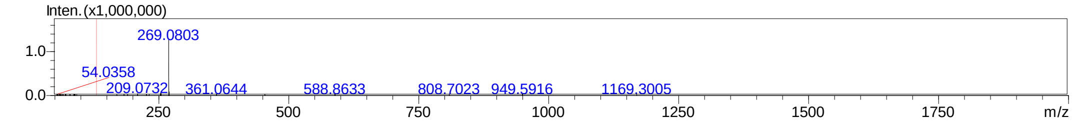

Rank	Score	Formula (M)	Ion	Meas. m/z	Pred. m/z	Diff (mDa)	Diff (ppm)	Iso Score	DBE
1	22.21	C24 H24 O10	[M+H]+	473.1445	473.1442	0.3	0.63	22.21	13.0

C41 Y19

Event#: 1 MS(E+) Ret. Time : 38.773 Scan# : 3818

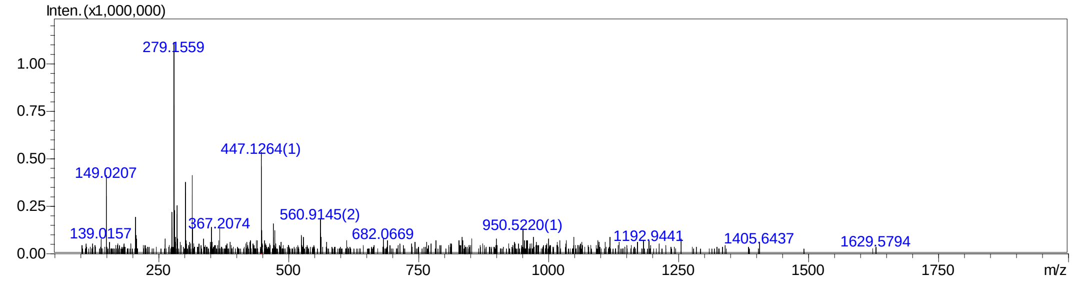

MS/MS(E+) Ret. Time : 38.773 Scan# : 3819 Precursor : 447.1264 Cutoff : 123

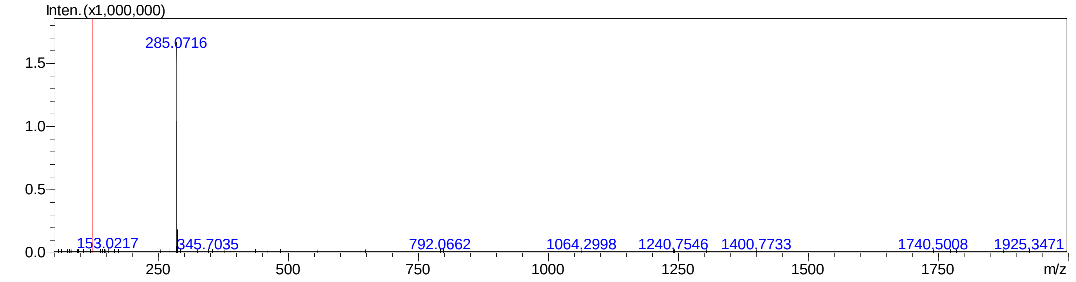

Rank	Score	Formula (M)	Ion	Meas. m/z	Pred. m/z	Diff (mDa)	Diff (ppm)	Iso Score	DBE
2	45.08	C22 H22 O10	[M+H]+	447.1264	447.1286	-2.2	-4.92	49.97	12.0

C6H10O5

C16H12O5

C42 Y20 300 532=284+ 248==162+86==

Event#: 1 MS(E+) Ret. Time : 38.997 Scan# : 3840

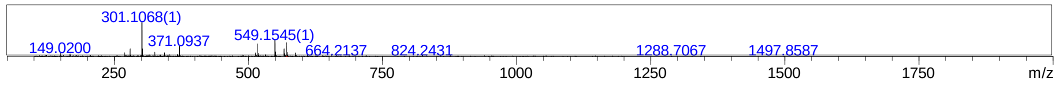

Event#: 4 MS(E-) Ret. Time : 38.997 Scan# : 3843

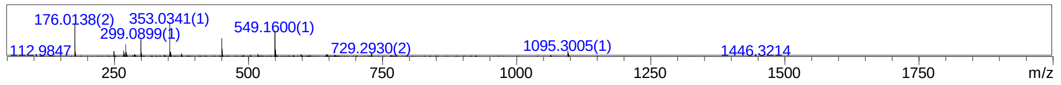

Rank	Score	Formula (M)	Ion	Meas. m/z	Pred. m/z	Diff (mDa)	Diff (ppm)	Iso Score	DBE
1	82.39	C17 H16 O5	[M+H]+	301.1068	301.1071	-0.3	-1.00	82.39	10.0

Rank	Score	Formula (M)	Ion	Meas. m/z	Pred. m/z	Diff (mDa)	Diff (ppm)	Iso Score	DBE
5	38.51	C26 H28 O13	[M+H]+	549.1545	549.1603	-5.8	-10.56	100.00	13.0

C44 Y21

Event#: 4 MS(E-) Ret. Time : 39.683 Scan# : 3911

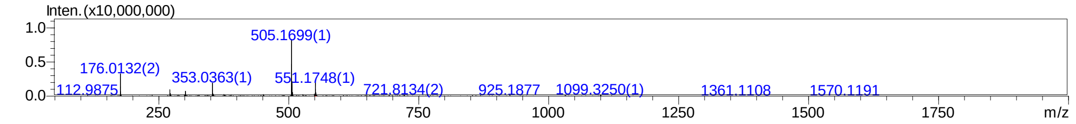

Event#: 5 MS/MS(E-) Ret. Time : 39.683 Scan# : 3912 Precursor : 551.1747 Cutoff : 152

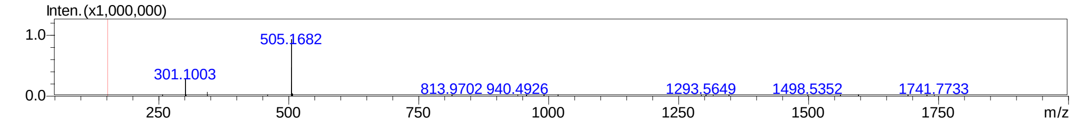

Rank Score Formula (M) Ion Meas. m/z Pred. m/z Diff (mDa) Diff (ppm) Iso Score DBE
1 92.99 C25 H30 O11 [M-H]- 505.1699 505.1715 -1.6 -3.17 98.32 11.0
C8H14O7 C17H18O5

C46 Y22
Event#: 1 MS(E+) Ret. Time : 40.138 Scan# : 3953

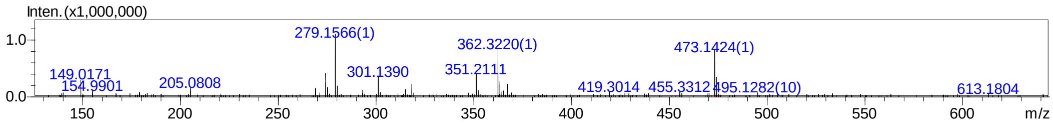

Event#: 2 MS/MS(E+) Ret. Time : 40.138 Scan# : 3954 Precursor : 473.1422 Cutoff : 130

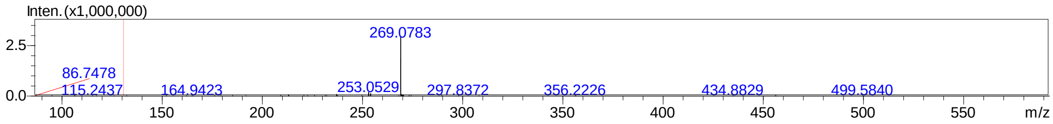

Rank Score Formula (M) Ion Meas. m/z Pred. m/z Diff (mDa) Diff (ppm) Iso Score DBE
1 63.78 C22 H26 O10 [M+Na]+ 473.1424 473.1418 0.6 1.27 64.22 10.0
2 63.47 C24 H24 O10 [M+H]+ 473.1424 473.1442 -1.8 -3.80 68.25 13.0
C16H12O4 C6H16O7
Rank Score Formula (M) Ion Meas. m/z Pred. m/z Diff (mDa) Diff (ppm) Iso Score DBE
2 16.74 C16 H12 O4 [M+H]+ 269.0783 269.0808 -2.5 -9.29 35.55 11.0
C8H14O7

C47 Y23
Event#: 4 MS(E-) Ret. Time : 40.430 Scan# : 3985

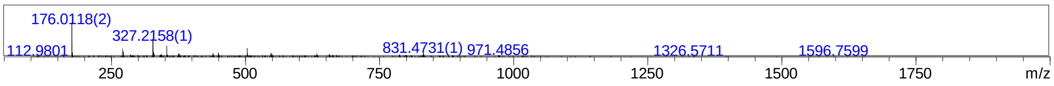

Event#: 5 MS/MS(E-) Ret. Time : 40.430 Scan# : 3986 Precursor : 503.1153 Cutoff : 139

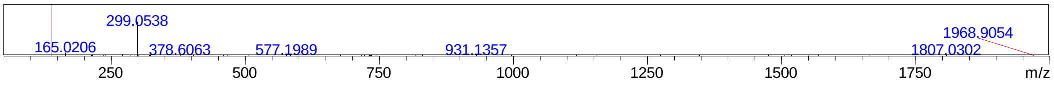

Rank Score Formula (M) Ion Meas. m/z Pred. m/z Diff (mDa) Diff (ppm) Iso Score DBE
1 0.00 C16 H12 O6 [M-H]- 299.0538 299.0561 -2.3 -7.69 0.00 11.0
Rank Score Formula (M) Ion Meas. m/z Pred. m/z Diff (mDa) Diff (ppm) Iso Score DBE
1 45.39 C31 H20 O7 [M-H]- 503.1154 503.1136 1.8 3.58 48.52 22.0
4 37.04 C24 H24 O12 [M-H]- 503.1154 503.1195 -4.1 -8.15 63.32 13.0
C8H14O7 C16H12O6

Y24
Event#: 4 MS(E-) Ret. Time : 41.675 Scan# : 4108

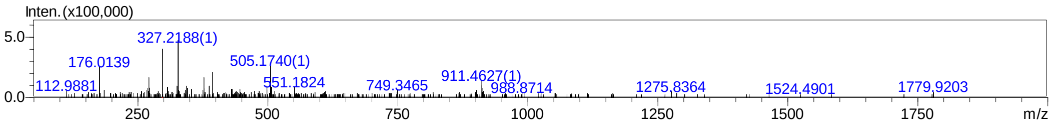

Rank Score Formula (M) Ion Meas. m/z Pred. m/z Diff (mDa) Diff (ppm) Iso Score DBE
1 24.09 C25 H30 O11 [M-H]- 505.1740 505.1715 2.5 4.95 26.73 11.0
C8H16O7

C51 Y25 532=284+ 248==162+86==

Event#: 1 MS(E+) Ret. Time : 41.950 Scan# : 4132

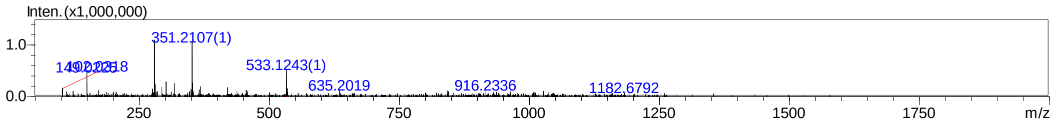

Event#: 2 MS/MS(E+) Ret. Time : 41.950 Scan# : 4133 Precursor : 533.1244 Cutoff : 147

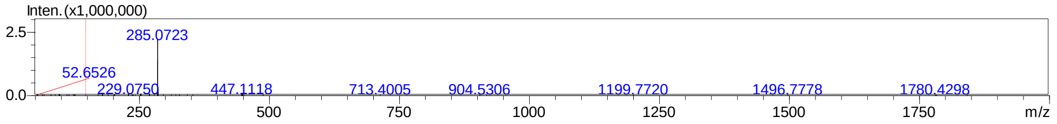

Rank	Score	Formula (M)	Ion	Meas. m/z	Pred. m/z	Diff (mDa)	Diff (ppm)	Iso Score	DBE
4	26.13	C25 H24 O13	[M+H]+	533.1243	533.1290	-4.7	-8.82	50.45	14.0
5	9.41	C16 H30 O18	[M+Na]+	533.1243	533.1324	-8.1	-15.19	35.96	2.0
6	5.32	C18 H28 O18	[M+H]+	533.1243	533.1348	-10.5	-19.70	37.62	5.0

Rank	Score	Formula (M)	Ion	Meas. m/z	Pred. m/z	Diff (mDa)	Diff (ppm)	Iso Score	DBE
2	22.07	C16 H12 O5	[M+H]+	285.0723	285.0758	-3.5	-12.28	65.07	11.0

C50 Y26

Event#: 2 MS/MS(E+) Ret. Time : 41.778 Scan# : 4116 Precursor : 317.1023 Cutoff : 87

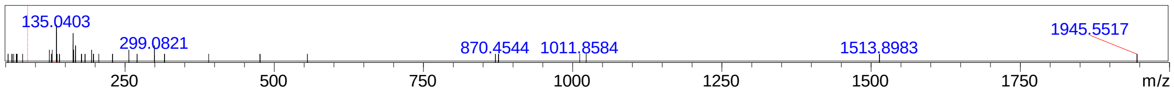

Event#: 4 MS(E-) Ret. Time : 41.778 Scan# : 4118

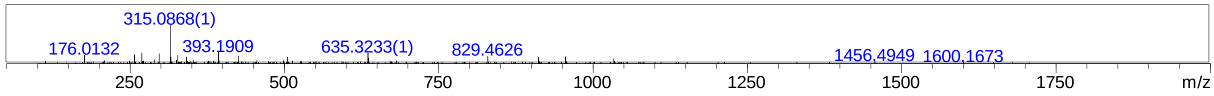

Rank	Score	Formula (M)	Ion	Meas. m/z	Pred. m/z	Diff (mDa)	Diff (ppm)	Iso Score	DBE
1	74.37	C17 H16 O6	[M-H]-	315.0868	315.0874	-0.6	-1.90	76.09	10.0

C56 Y27 formononetin

Event#: 1 MS(E+) Ret. Time : 45.315 Scan# : 4465

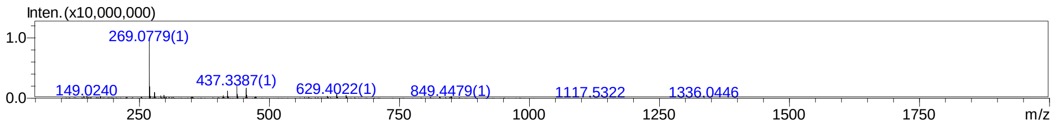

Event#: 2 MS/MS(E+) Ret. Time : 45.255 Scan# : 4460 Precursor : 269.0791 Cutoff : 74

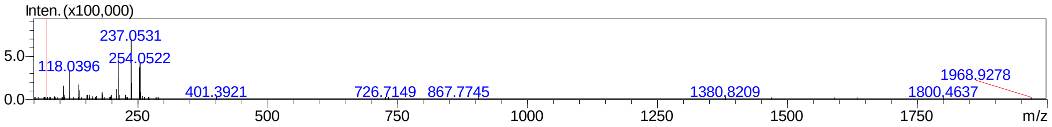

Event#: 4 MS(E-) Ret. Time : 45.315 Scan# : 4468

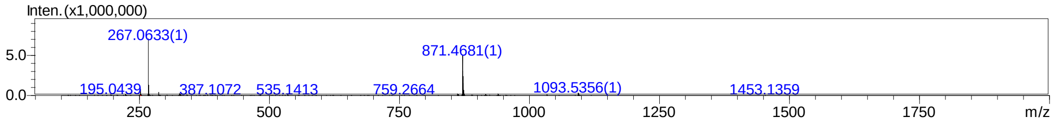

Rank Score Formula (M) Ion Meas. m/z Pred. m/z Diff (mDa) Diff (ppm) Iso Score DBE

2 61.30 C16 H12 O4 [M+H]+ 269.0791 269.0808 -1.7 -6.32 79.82 11.0

C58 Y28 astrapterocarpan

Event#: 1 MS(E+) Ret. Time : 46.158 Scan# : 4547

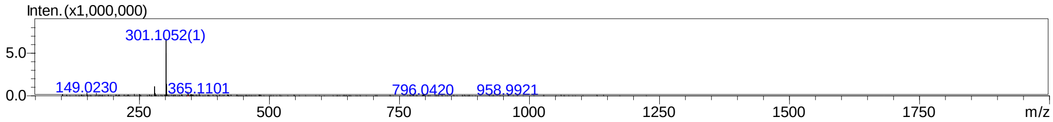

Rank Score Formula (M) Ion Meas. m/z Pred. m/z Diff (mDa) Diff (ppm) Iso Score DBE

1 56.73 C17 H16 O5 [M+H]+ 301.1052 301.1071 -1.9 -6.31 73.77 10.0

C59 Y29 astraisoflavan

Event#: 1 MS(E+) Ret. Time : 46.708 Scan# : 4601

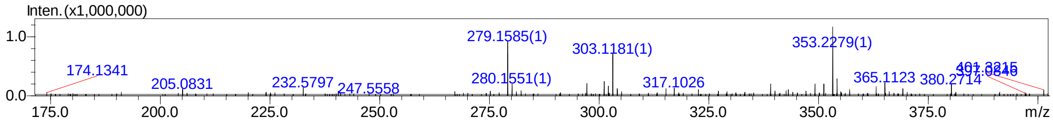

Rank Score Formula (M) Ion Meas. m/z Pred. m/z Diff (mDa) Diff (ppm) Iso Score DBE

2 12.80 C17 H18 O5 [M+H]+ 303.1181 303.1227 -4.6 -15.18 48.89 9.0

C62 Y30 286

Event#: 4 MS(E-) Ret. Time : 49.432 Scan# : 4872

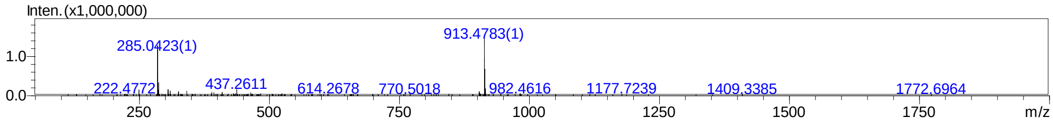

Event#: 5 MS/MS(E-) Ret. Time : 49.432 Scan# : 4873 Precursor : 285.0422 Cutoff : 78

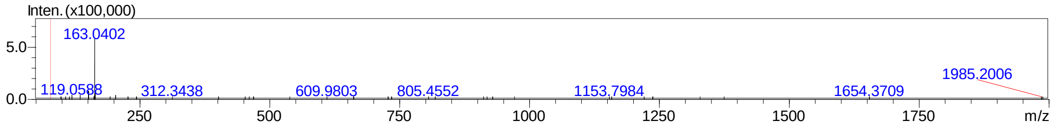

Rank Score Formula (M) Ion Meas. m/z Pred. m/z Diff (mDa) Diff (ppm) Iso Score DBE

1 26.48 C15 H10 O6 [M-H]- 285.0423 285.0405 1.8 6.31 34.43 11.0

C45 Z1

Event#: 4 MS(E-) Ret. Time : 39.743 Scan# : 3917

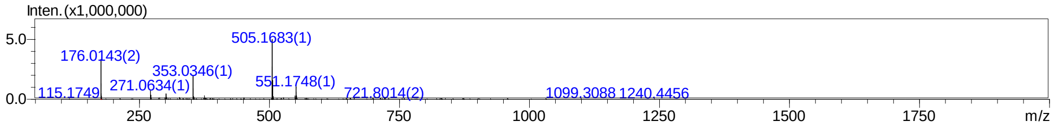

Rank Score Formula (M) Ion Meas. m/z Pred. m/z Diff (mDa) Diff (ppm) Iso Score DBE

10 0.00 C48 H78 O19 [M-H]- 957.5126 957.5065 6.1 6.37 0.00 10.0

C48 Z1

Event#: 4 MS(E-) Ret. Time : 40.825 Scan# : 4024

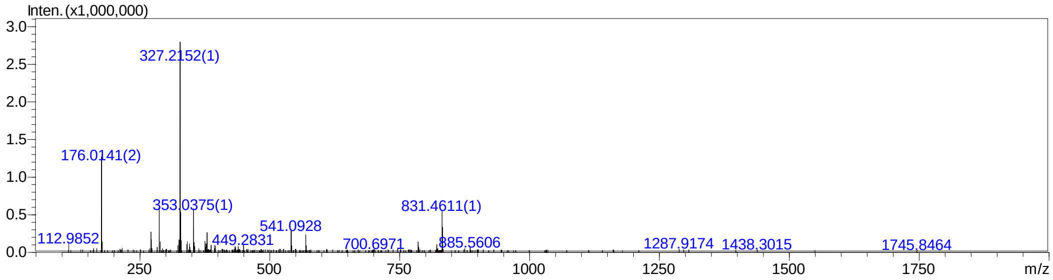

MS/MS(E-) Ret. Time : 40.825 Scan# : 4025 Precursor : 831.4614 Cutoff : 230

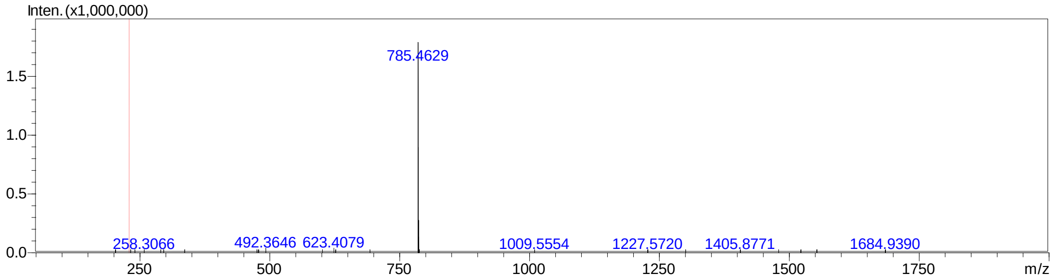

Rank Score Formula (M) Ion Meas. m/z Pred. m/z Diff (mDa) Diff (ppm) Iso Score DBE

7 0.00 C41 H70 O14 [M-H]- 785.4629 785.4693 -6.4 -8.15 0.00 7.0

C49 Z2 **ZG-V**

Event#: 4 MS(E-) Ret. Time : 41.108 Scan# : 4052

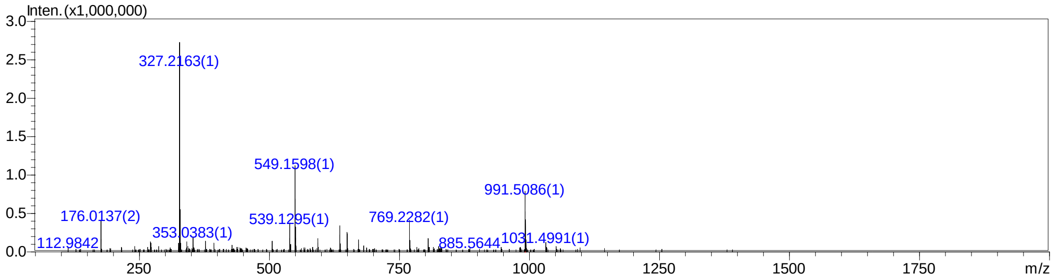

MS/MS(E-) Ret. Time : 41.108 Scan# : 4053 Precursor : 991.5089 Cutoff : 274

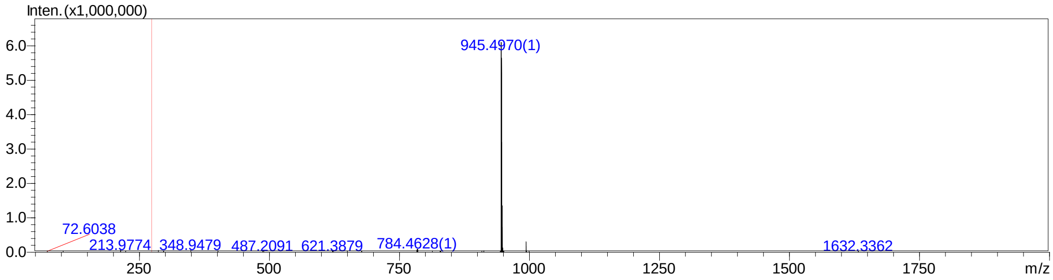

Rank Score Formula (M) Ion Meas. m/z Pred. m/z Diff (mDa) Diff (ppm) Iso Score DBE

1 68.58 C48 H80 O21 [M-H]- 991.5086 991.5119 -3.3 -3.33 72.82 9.0

2 68.58 C46 H76 O19 [M+CH3COO]- 991.5086 991.5119 -3.3 -3.33 72.82 9.0

3 68.58 C47 H78 O19 [M+HCOO]- 991.5086 991.5119 -3.3 -3.33 72.82 9.0

C53 Z3 **ZG-IV**

Event#: 4 MS(E-) Ret. Time : 42.612 -> 43.135 Scan# : 4200 -> 4252

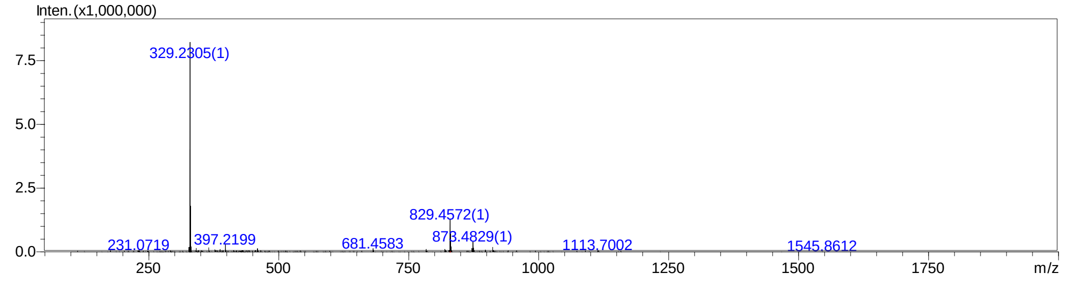

MS/MS(E-) Ret. Time : 42.612 -> 43.135 Scan# : 4201 -> 4253 Precursor : 829.4586 Cutoff : 229

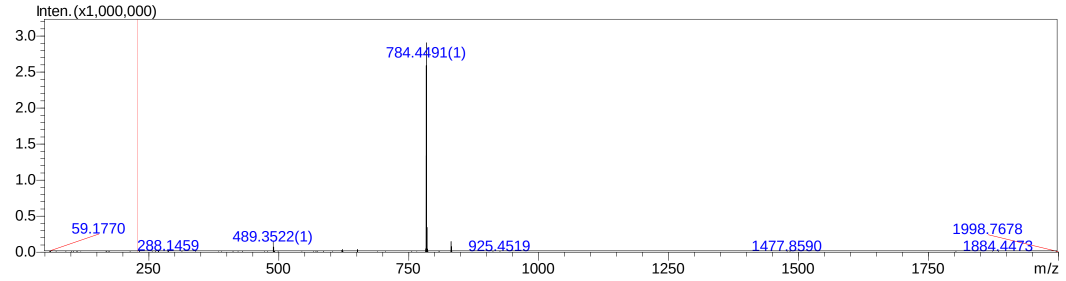

Rank Score Formula (M) Ion Meas. m/z Pred. m/z Diff (mDa) Diff (ppm) Iso Score DBE

2 81.60 C41 H68 O14 [M+HCOO]- 829.4572 829.4591 -1.9 -2.29 84.32 8.0

C52 Z4 =G I

Event#: 4 MS(E-) Ret. Time : 42.062 Scan# : 4145

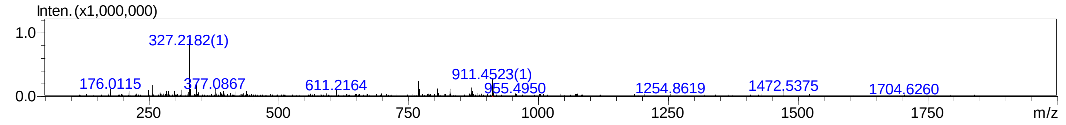

Event#: 5 MS/MS(E-) Ret. Time : 42.062 Scan# : 4146 Precursor : 911.4525 Cutoff : 252

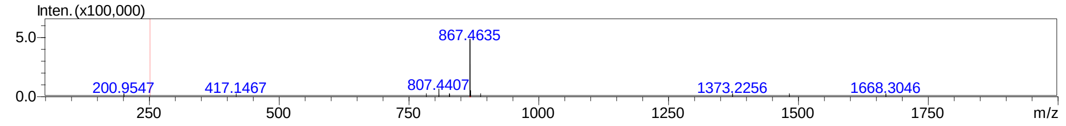

Rank Score Formula (M) Ion Meas. m/z Pred. m/z Diff (mDa) Diff (ppm) Iso Score DBE

14 0.00 C45 H72 O16 [M-H]- 867.4635 867.4748 -11.3 -13.03 0.00 10.0

C54 Z5 ISO ZG II

Event#: 4 MS(E-) Ret. Time : 42.560 Scan# : 4194

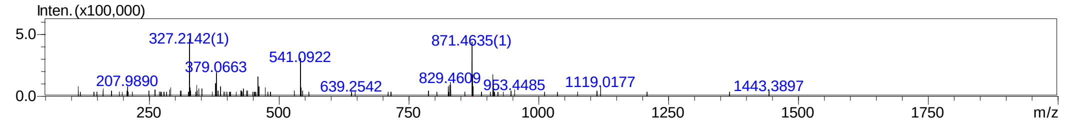

Event#: 5 MS/MS(E-) Ret. Time : 42.560 Scan# : 4195 Precursor : 871.4641 Cutoff : 241

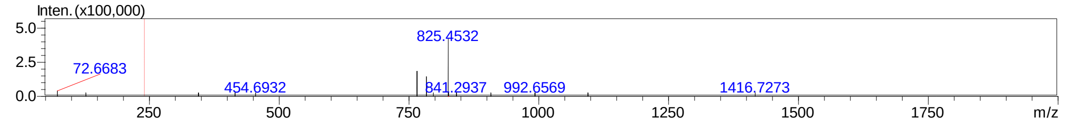

Rank	Score	Formula (M)	Ion	Meas. m/z	Pred. m/z	Diff (mDa)	Diff (ppm)	Iso Score	DBE
**14**	**0.00**	**C43 H70 O15**	**[M-H]-**	**825.4532**	**825.4642**	**-11.0**	**-13.33**	**0.00**	**9.0**

C55 Z6 Bb

Event#: 4 MS(E-) Ret. Time : 44.190 Scan# : 4357

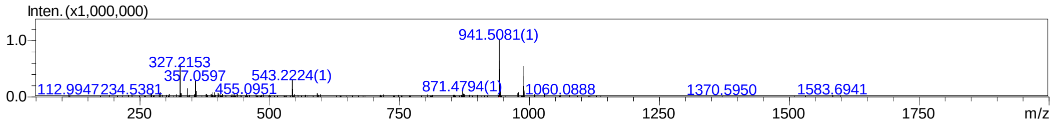

Event#: 5 MS/MS(E-) Ret. Time : 44.190 Scan# : 4358 Precursor : 941.5081 Cutoff : 260

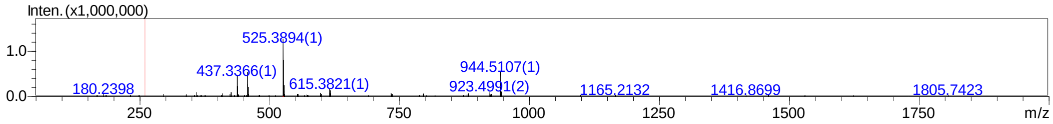

Rank	Score	Formula (M)	Ion	Meas. m/z	Pred. m/z	Diff (mDa)	Diff (ppm)	Iso Score	DBE
1	44.34	C48 H78 O18	[M-H]-	941.5081	941.5115	-3.4	-3.61	47.43	10.0

C60 Z7 G-II

Event#: 1 MS(E+) Ret. Time : 46.768 Scan# : 4607

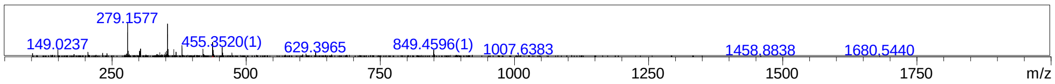

Event#: 5 MS/MS(E-) Ret. Time : 46.708 Scan# : 4605 Precursor : 871.4660 Cutoff : 241

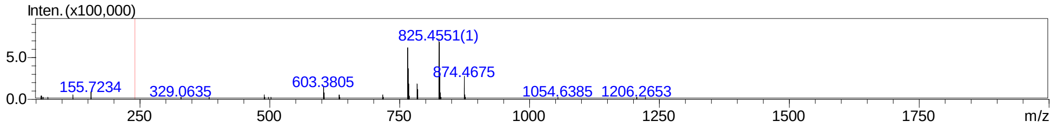

Rank	Score	Formula (M)	Ion	Meas. m/z	Pred. m/z	Diff (mDa)	Diff (ppm)	Iso Score	DBE
1	54.12	C43 H70 O15	[M+Na]+	849.4596	849.4607	-1.1	-1.29	54.52	9.0

Rank	Score	Formula (M)	Ion	Meas. m/z	Pred. m/z	Diff (mDa)	Diff (ppm)	Iso Score	DBE
1	66.10	C44 H72 O17	[M-H]-	871.4656	871.4697	-4.1	-4.70	72.83	9.0
**2**	**66.10**	**C43 H70 O15**	**[M+HCOO]-**	**871.4656**	**871.4697**	**-4.1**	**-4.70**	**72.83**	**9.0**

C61 Z8 ==G I

Event#: 4 MS(E-) Ret. Time : 47.172 -> 48.985 Scan# : 4649 -> 4828

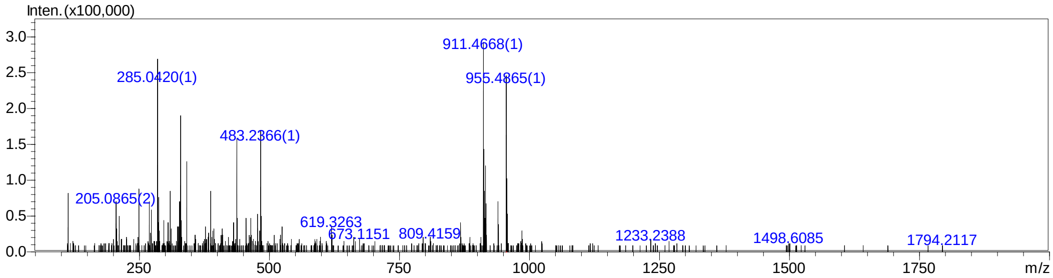

MS/MS(E-) Ret. Time : 47.172 -> 48.985 Scan# : 4650 -> 4829 Precursor : 955.4807 Cutoff : 264

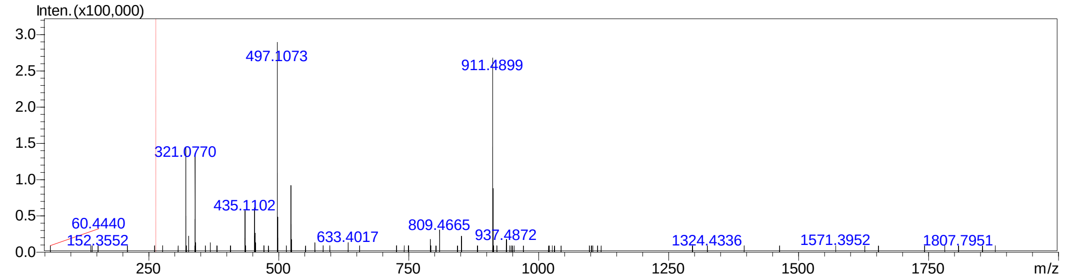

Rank Score Formula (M) Ion Meas. m/z Pred. m/z Diff (mDa) Diff (ppm) Iso Score DBE

1 54.01 C45 H70 O16 [M+HCOO]- 911.4668 911.4646 2.2 2.41 55.98 11.0

Event#: 4 MS(E-) Ret. Time : 47.223 Scan# : 4655

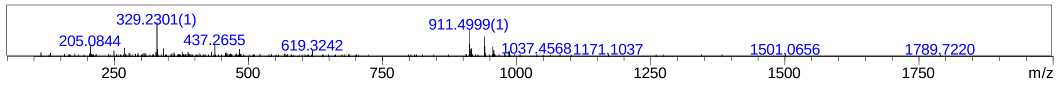

Event#: 5 MS/MS(E-) Ret. Time : 47.223 Scan# : 4656 Precursor : 911.5002 Cutoff : 252

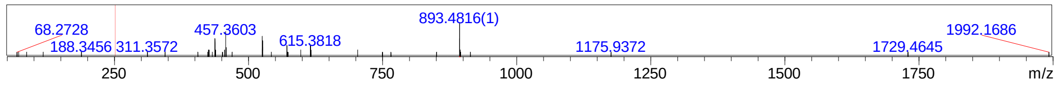

Rank Score Formula (M) Ion Meas. m/z Pred. m/z Diff (mDa) Diff (ppm) Iso Score DBE

C64 Z9 **i-ZG--I**

Event#: 4 MS(E-) Ret. Time : 50.713 Scan# : 4997

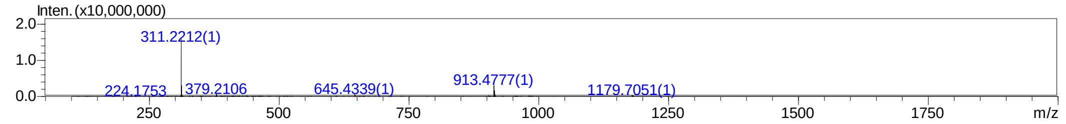

Rank	Score	Formula (M)	Ion	Meas. m/z	Pred. m/z	Diff (mDa)	Diff (ppm)	Iso Score	DBE
1	95.07	C46 H74 O18	[M-H]-	913.4777	913.4802	-2.5	-2.74	99.40	10.0
2	95.07	C44 H70 O16	[M+CH3COO]-	913.4777	913.4802	-2.5	-2.74	99.40	10.0
3	95.07	C45 H72 O16	[M+HCOO]-	913.4777	913.4802	-2.5	-2.74	99.40	10.0

C65 Z10

Event#: 4 MS(E-) Ret. Time : 51.607 Scan# : 5085

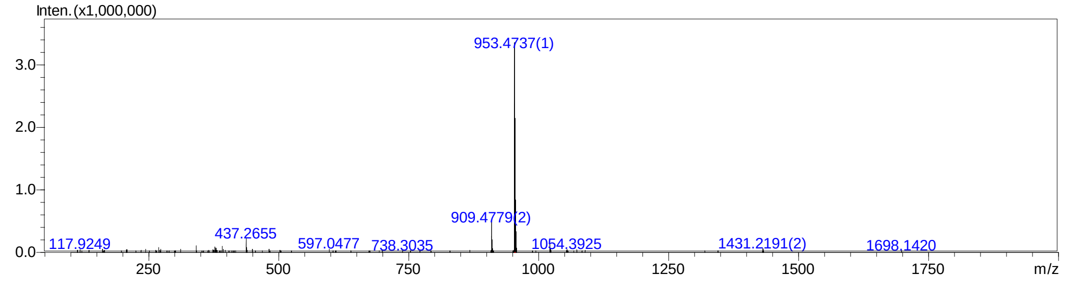

MS/MS(E-) Ret. Time : 51.607 Scan# : 5086 Precursor : 953.9829 Cutoff : 264

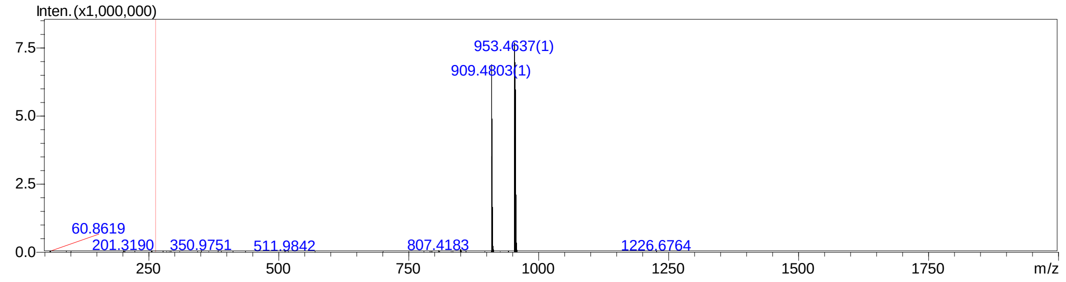

Rank Score Formula (M) Ion Meas. m/z Pred. m/z Diff (mDa) Diff (ppm) Iso Score DBE

5 40.65 C54 H68 O12 [M+HCOO]- 953.4637 953.4693 -5.6 -5.87 50.00 21.0

7 32.11 C43 H72 O20 [M+HCOO]- 953.4637 953.4599 3.8 3.99 34.70 8.0

8 32.11 C44 H74 O22 [M-H]- 953.4637 953.4599 3.8 3.99 34.70 8.0

10 24.70 C68 H60 O2 [M+HCOO]- 953.4637 953.4575 6.2 6.50 32.94 39.0

15 13.14 C47 H72 O17 [M+HCOO]- 953.4637 953.4752 -11.5 -12.06 38.08 12.0

C2H2O C45H70O16

C67 Z-

Event#: 1 MS(E+) Ret. Time : 59.933 -> 62.997 Scan# : 5900 -> 6204

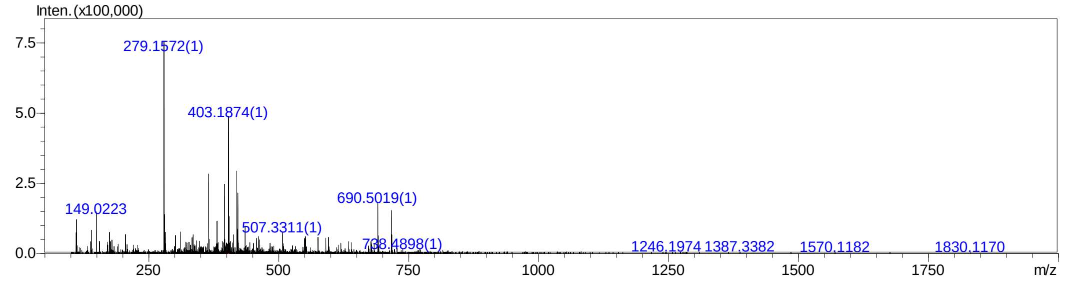

MS/MS(E+) Ret. Time : 59.933 -> 62.997 Scan# : 5901 -> 6205 Precursor : 403.1850 Cutoff : 111

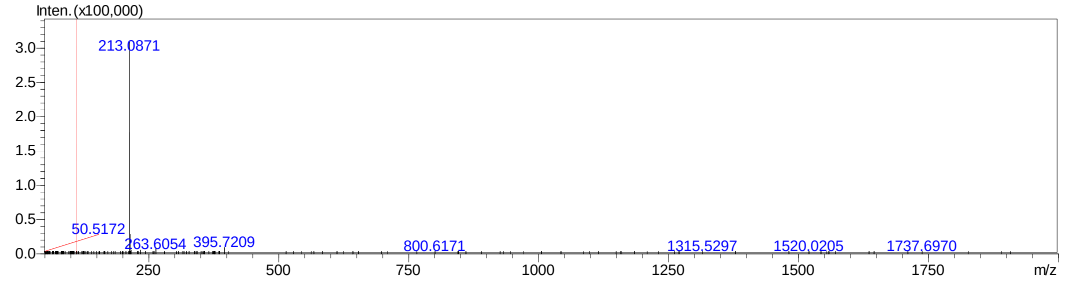

Rank Score Formula (M) Ion Meas. m/z Pred. m/z Diff (mDa) Diff (ppm) Iso Score DBE

1 36.86 C12 H14 O2 [M+Na]+ 213.0871 213.0886 -1.5 -7.04 52.96 6.0

2M+NA+=403

C68 E-

Event#: 1 MS(E+) Ret. Time : 61.708 -> 62.542 Scan# : 6077 -> 6159

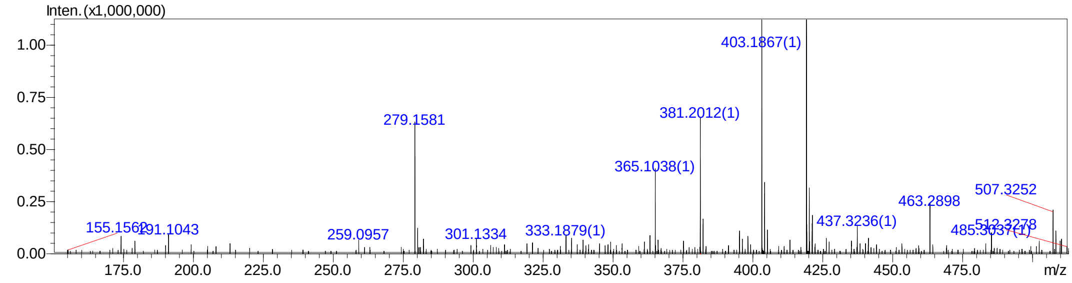

MS/MS(E+) Ret. Time : 61.708 -> 62.542 Scan# : 6078 -> 6160 Precursor : 381.2017 Cutoff : 105

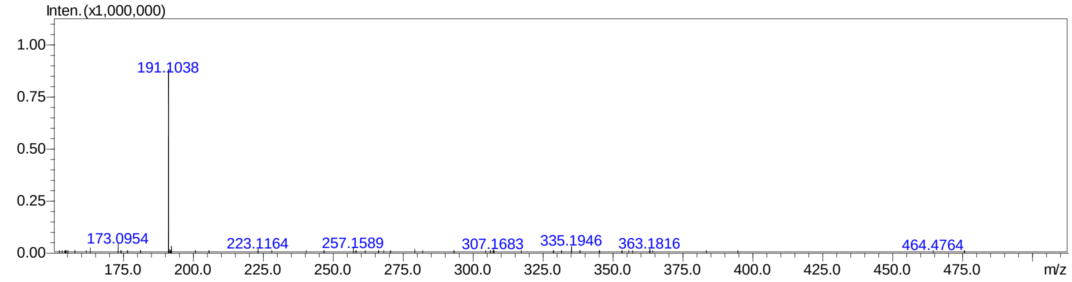

Rank	Score	Formula (M)	Ion	Meas. m/z	Pred. m/z	Diff (mDa)	Diff (ppm)	Iso Score	DBE
1	78.12	C24 H28 O4	[M+Na]+	403.1867	403.1880	-1.3	-3.22	82.71	11.0

Rank	Score	Formula (M)	Ion	Meas. m/z	Pred. m/z	Diff (mDa)	Diff (ppm)	Iso Score	DBE
1	59.05	C22 H30 O4	[M+Na]+	381.2012	381.2036	-2.4	-6.30	76.69	8.0
2	18.30	C24 H28 O4	[M+H]+	381.2012	381.2060	-4.8	-12.59	55.30	11.0

Rank	Score	Formula (M)	Ion	Meas. m/z	Pred. m/z	Diff (mDa)	Diff (ppm)	Iso Score	DBE
2	12.97	C12 H14 O2	[M+H]+	191.1038	191.1067	-2.9	-15.17	49.46	6.0

C5 X1 =

Event#: 1 MS(E+) Ret. Time : 4.498 Scan# : 443

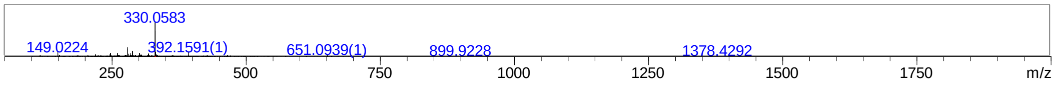

Event#: 4 MS(E-) Ret. Time : 4.498 Scan# : 446

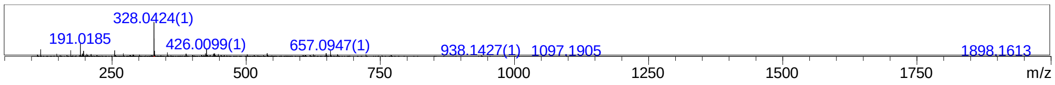

Rank	Score	Formula (M)	Ion	Meas. m/z	Pred. m/z	Diff (mDa)	Diff (ppm)	Iso Score	DBE
1	61.39	C11 H11 N3 O9	[M+H]+	330.0583	330.0568	1.5	4.54	67.35	8.0

C6 X2 =

Event#: 1 MS(E+) Ret. Time : 6.480 Scan# : 638

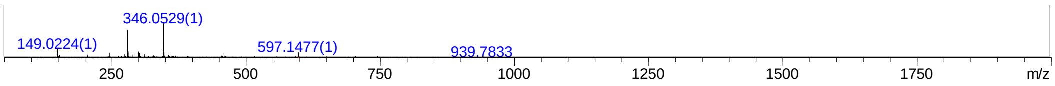

Event#: 4 MS(E-) Ret. Time : 6.480 Scan# : 641

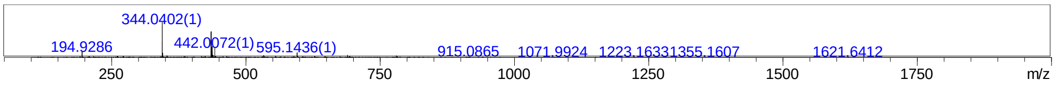

Rank	Score	Formula (M)	Ion	Meas. m/z	Pred. m/z	Diff (mDa)	Diff (ppm)	Iso Score	DBE
3	65.91	C11 H11 N3 O10	[M+H]+	346.0529	346.0517	1.2	3.47	70.25	8.0

C7

Event#: 1 MS(E+) Ret. Time : 9.227 Scan# : 910

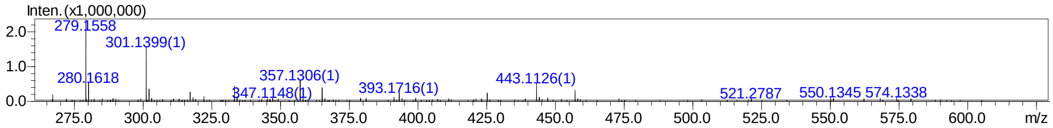

Event#: 2 MS/MS(E+) Ret. Time : 9.227 Scan# : 911 Precursor : 443.1126 Cutoff : 122

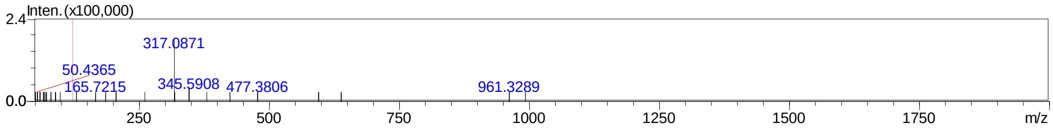

Rank	Score	Formula (M)	Ion	Meas. m/z	Pred. m/z	Diff (mDa)	Diff (ppm)	Iso Score	DBE
1	59.89	C26 H18 O7	[M+H]+	443.1126	443.1125	0.1	0.23	59.89	18.0

C8 X3

Event#: 4 MS(E-) Ret. Time : 9.398 Scan# : 929

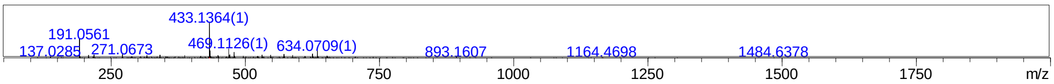

Event#: 5 MS/MS(E-) Ret. Time : 9.398 Scan# : 930 Precursor : 433.1364 Cutoff : 119

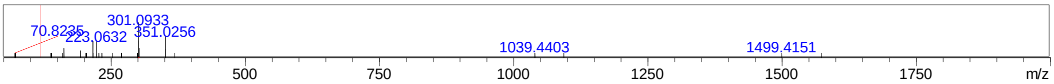

Rank	Score	Formula (M)	Ion	Meas. m/z	Pred. m/z	Diff (mDa)	Diff (ppm)	Iso Score	DBE
1	71.60	C18 H26 O12	[M-H]-	433.1364	433.1352	1.2	2.77	74.91	6.0

Rank	Score	Formula (M)	Ion	Meas. m/z	Pred. m/z	Diff (mDa)	Diff (ppm)	Iso Score	DBE
1	37.82	C13 H18 O8	[M-H]-	301.0933	301.0929	0.4	1.33	38.13	5.0

C5H10O5

C10 X3 benzoic acid+xylan

Event#: 4 MS(E-) Ret. Time : 11.125 Scan# : 1099

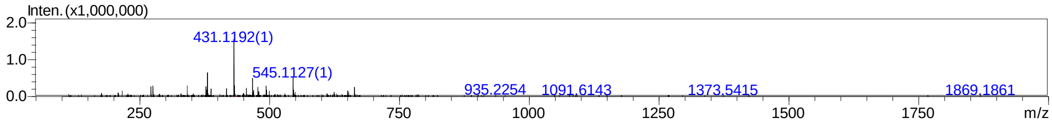

Event#: 5 MS/MS(E-) Ret. Time : 11.125 Scan# : 1100 Precursor : 431.1190 Cutoff : 119

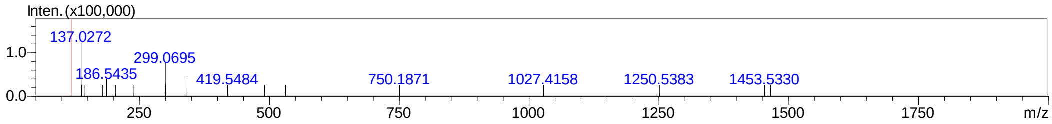

Rank	Score	Formula (M)	Ion	Meas. m/z	Pred. m/z	Diff (mDa)	Diff (ppm)	Iso Score	DBE
1	89.05	C18 H24 O12	[M-H]-	431.1192	431.1195	-0.3	-0.70	89.05	7.0

C5H8O4 C13H16O8

C11 X4 =

Event#: 1 MS(E+) Ret. Time : 12.653 Scan# : 1248

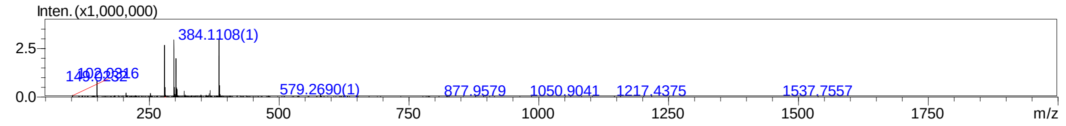

Event#: 4 MS(E-) Ret. Time : 12.653 Scan# : 1251

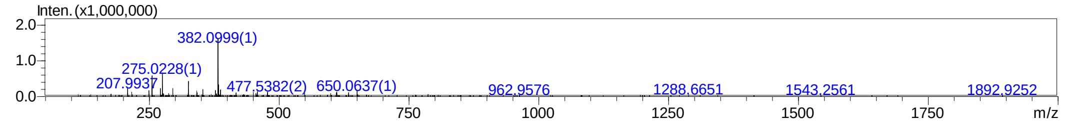

Rank	Score	Formula (M)	Ion	Meas. m/z	Pred. m/z	Diff (mDa)	Diff (ppm)	Iso Score	DBE
1	79.82	C13 H21 N O12	[M+H]+	384.1127	384.1137	-1.0	-2.60	83.14	4.0

C12 X5

Event#: 1 MS(E+) Ret. Time : 13.847 Scan# : 1366

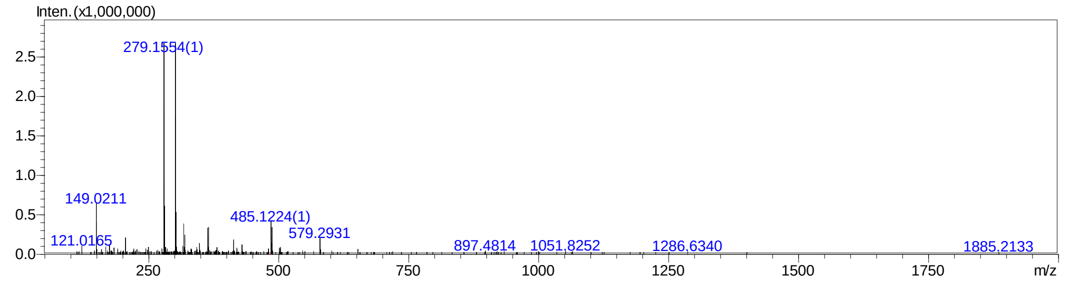

MS/MS(E+) Ret. Time : 13.847 Scan# : 1367 Precursor : 485.1225 Cutoff : 134

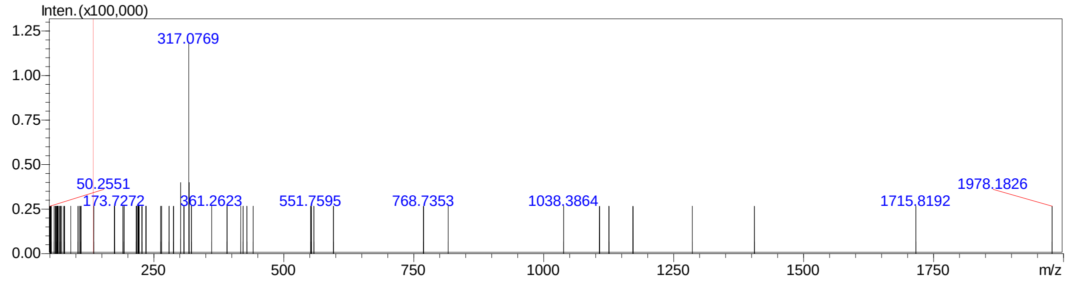

Rank	Score	Formula (M)	Ion	Meas. m/z	Pred. m/z	Diff (mDa)	Diff (ppm)	Iso Score	DBE
1	15.37	C26 H22 O8	[M+Na]+	485.1224	485.1207	1.7	3.50	16.39	16.0

Rank	Score	Formula (M)	Ion	Meas. m/z	Pred. m/z	Diff (mDa)	Diff (ppm)	Iso Score	DBE
2	10.77	C20 H12 O4	[M+H]+	317.0769	317.0808	-3.9	-12.30	31.80	15.0

+C6H12O5

C13 X8

Event#: 4 MS(E-) Ret. Time : 17.807 Scan# : 1758

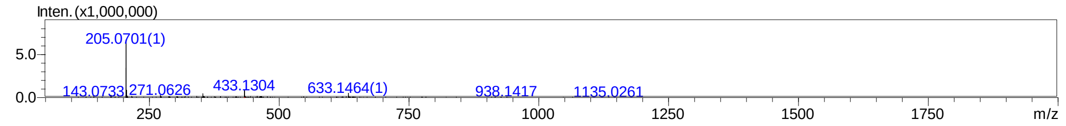

Event#: 5 MS/MS(E-) Ret. Time : 17.807 Scan# : 1759 Precursor : 433.1305 Cutoff : 119

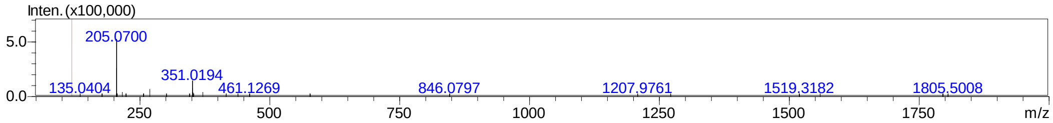

Event#: 4 MS(E-) Ret. Time : 18.557 Scan# : 1832

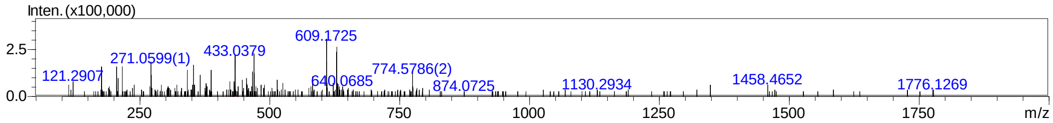

Rank	Score	Formula (M)	Ion	Meas. m/z	Pred. m/z	Diff (mDa)	Diff (ppm)	Iso Score	DBE
1	35.69	C8 H14 O6	[M-H]-	205.0701	205.0718	-1.7	-8.29	62.50	2.0

C14 X9 lambertianic acid

Event#: 1 MS(E+) Ret. Time : 21.147 Scan# : 2082

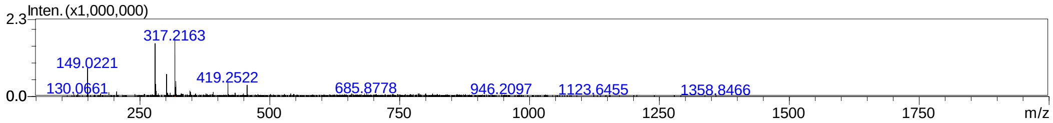

Event#: 2 MS/MS(E+) Ret. Time : 21.147 Scan# : 2083 Precursor : 317.2162 Cutoff : 87

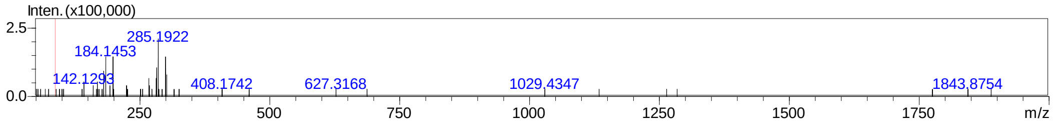

Rank	Score	Formula (M)	Ion	Meas. m/z	Pred. m/z	Diff (mDa)	Diff (ppm)	Iso Score	DBE
1	5.39	C20 H28 O3	[M+H]+	317.2163	317.2111	5.2	16.39	23.48	7.0

Event#: 4 MS(E-) Ret. Time : 22.590 Scan# : 2223

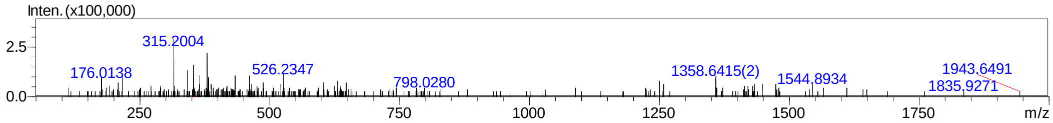

Rank	Score	Formula (M)	Ion	Meas. m/z	Pred. m/z	Diff (mDa)	Diff (ppm)	Iso Score	DBE
1	20.54	C20 H28 O3	[M-H]-	315.2004	315.1966	3.8	12.06	59.51	7.0

C15 X10 =

Event#: 4 MS(E-) Ret. Time : 22.132 Scan# : 2180

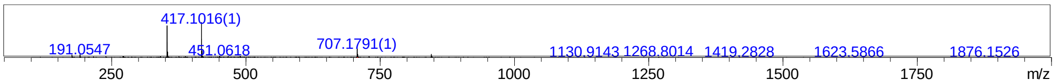

Rank	Score	Formula (M)	Ion	Meas. m/z	Pred. m/z	Diff (mDa)	Diff (ppm)	Iso Score	DBE
1	78.66	C17 H22 O12	[M-H]-	417.1016	417.1038	-2.2	-5.27	90.10	7.0

Rank	Score	Formula (M)	Ion	Meas. m/z	Pred. m/z	Diff (mDa)	Diff (ppm)	Iso Score	DBE
1	35.52	C7 H12 O6	[M-H]-	191.0547	191.0561	-1.4	-7.33	53.25	2.0

$C_{10}H_{12}O_7$

C16 X11 Cyclopentaneacetic acid =

Event#: 1 MS(E+) Ret. Time : 22.950 Scan# : 2256

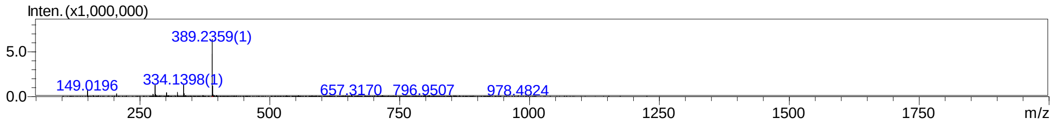

Event#: 4 MS(E-) Ret. Time : 22.950 Scan# : 2259

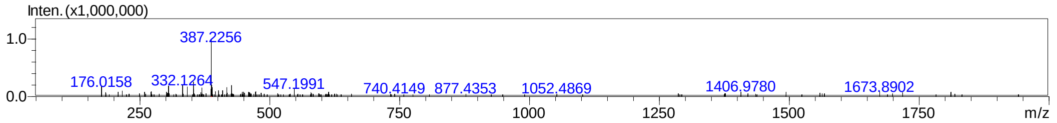

Rank Score Formula (M) Ion Meas. m/z Pred. m/z Diff (mDa) Diff (ppm) Iso Score DBE

1 4.02 C27 H32 O2 [M-H]- 387.2256 387.2330 -7.4 -19.11 25.57 12.0

Rank Score Formula (M) Ion Meas. m/z Pred. m/z Diff (mDa) Diff (ppm) Iso Score DBE

1 22.96 C23 H32 O5 [M+H]+ 389.2359 389.2323 3.6 9.25 48.33 8.0

C17 X12 +xylan =

Event#: 4 MS(E-) Ret. Time : 23.012 Scan# : 2264

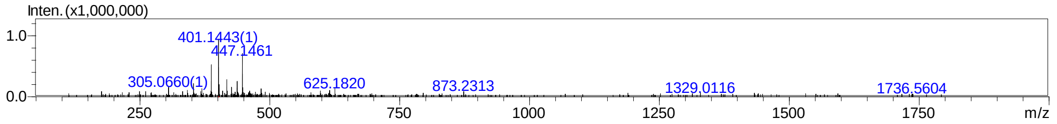

Event#: 5 MS/MS(E-) Ret. Time : 23.012 Scan# : 2265 Precursor : 401.1442 Cutoff : 111

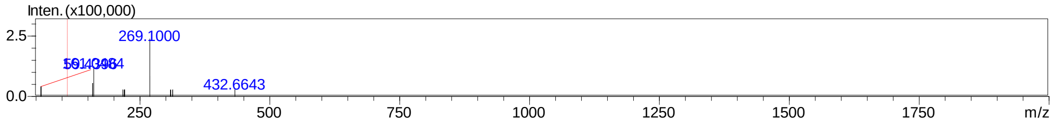

Rank Score Formula (M) Ion Meas. m/z Pred. m/z Diff (mDa) Diff (ppm) Iso Score DBE

1 58.11 C18 H26 O10 [M-H]- 401.1443 401.1453 -1.0 -2.49 60.36 6.0

Rank Score Formula (M) Ion Meas. m/z Pred. m/z Diff (mDa) Diff (ppm) Iso Score DBE

2 0.00 C13 H18 O6 [M-H]- 269.1000 269.1031 -3.1 -11.52 0.00 5.0

C5H8O4 xylan

C19 X13 (4-formyl-2,6-dimethoxyphenxoy)Acetic acid =

Event#: 4 MS(E-) Ret. Time : 24.347 Scan# : 2395

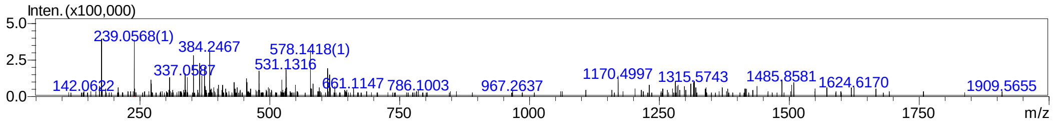

Event#: 5 MS/MS(E-) Ret. Time : 24.347 Scan# : 2396 Precursor : 239.0567 Cutoff : 66

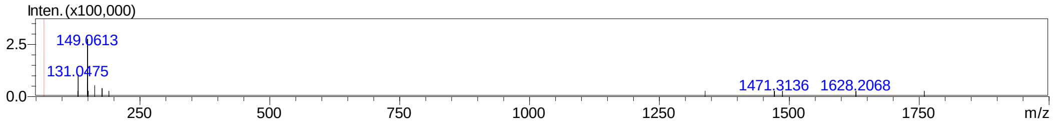

Rank Score Formula (M) Ion Meas. m/z Pred. m/z Diff (mDa) Diff (ppm) Iso Score DBE

1 62.78 C11 H12 O6 [M-H]- 239.0568 239.0561 0.7 2.93 65.96 6.0

C20 X14 Testosteroneon acetate

Event#: 1 MS(E+) Ret. Time : 25.662 Scan# : 2521

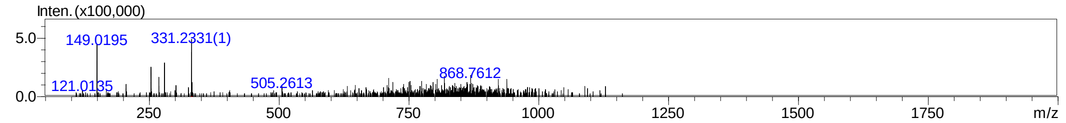

Event#: 2 MS/MS(E+) Ret. Time : 25.662 Scan# : 2522 Precursor : 331.2330 Cutoff : 91

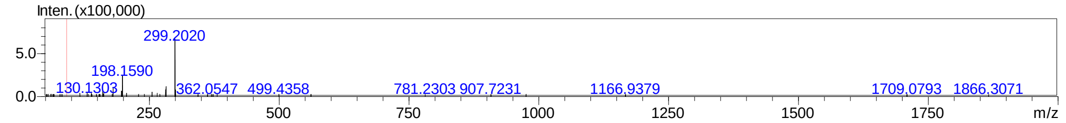

Rank	Score	Formula (M)	Ion	Meas. m/z	Pred. m/z	Diff (mDa)	Diff (ppm)	Iso Score	DBE
1	50.18	C21 H30 O3	[M+H]+	331.2296	331.2268	2.8	8.45	90.41	7.0

Rank	Score	Formula (M)	Ion	Meas. m/z	Pred. m/z	Diff (mDa)	Diff (ppm)	Iso Score	DBE
1	33.48	C20 H26 O2	[M+H]+	299.2020	299.2006	1.4	4.68	36.88	8.0

C21 X15

Event#: 1 MS(E+) Ret. Time : 26.743 Scan# : 2628

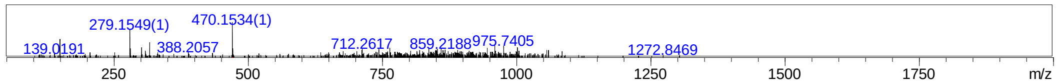

Event#: 4 MS(E-) Ret. Time : 26.743 Scan# : 2631

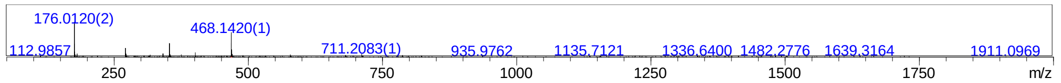

Event#: 5 MS/MS(E-) Ret. Time : 26.743 Scan# : 2632 Precursor : 468.1417 Cutoff : 129

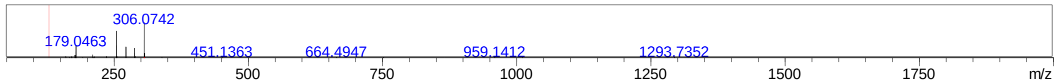

Rank	Score	Formula (M)	Ion	Meas. m/z	Pred. m/z	Diff (mDa)	Diff (ppm)	Iso Score	DBE
1	49.45	C18 H23 N5 O10	[M+H]+	470.1534	470.1518	1.6	3.40	52.61	10.0

Rank	Score	Formula (M)	Ion	Meas. m/z	Pred. m/z	Diff (mDa)	Diff (ppm)	Iso Score	DBE
7	23.10	C18 H23 N5 O10	[M-H]-	468.1420	468.1372	4.8	10.25	58.74	10.0

C22 X16

Event#: 1 MS(E+) Ret. Time : 26.967 Scan# : 2650

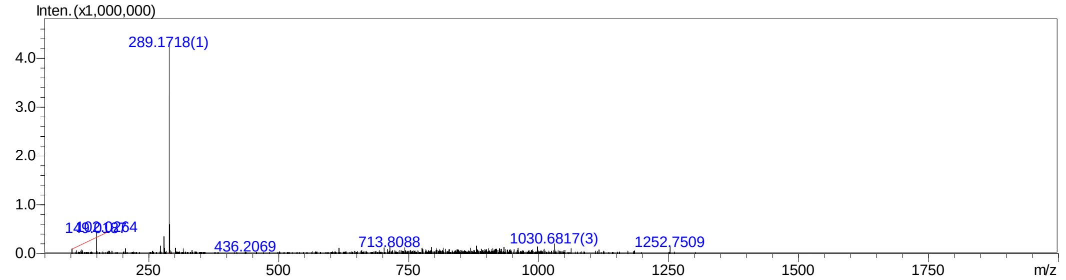

MS/MS(E+) Ret. Time : 26.967 Scan# : 2651 Precursor : 289.4785 Cutoff : 80

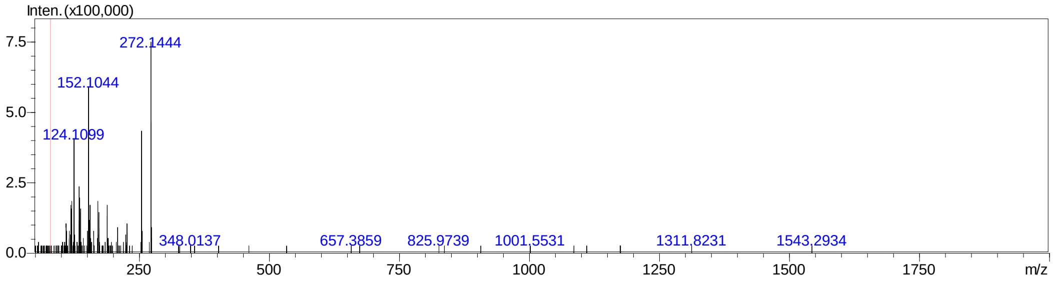

Rank Score Formula (M) Ion Meas. m/z Pred. m/z Diff (mDa) Diff (ppm) Iso Score DBE

2 47.38 C13 H24 N2 O5 [M+H]+ 289.1747 289.1758 -1.1 -3.80 50.94 3.0

C25 X18

Event#: 4 MS(E-) Ret. Time : 30.070 Scan# : 2959

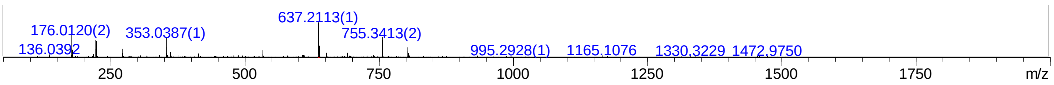

Event#: 5 MS/MS(E-) Ret. Time : 30.070 Scan# : 2960 Precursor : 637.2112 Cutoff : 176

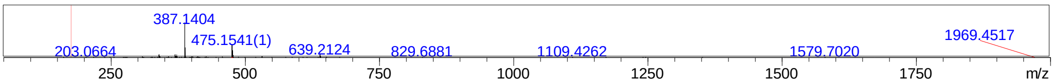

Event#: 6 MS3(E-) Ret. Time : 30.070 Scan# : 2961 Precursor : 475.1541 Cutoff : 131

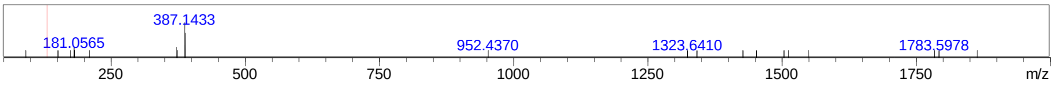

Rank Score Formula (M) Ion Meas. m/z Pred. m/z Diff (mDa) Diff (ppm) Iso Score DBE

2 73.32 C30 H38 O15 [M-H]- 637.2113 637.2138 -2.5 -3.92 79.09 12.0

Rank Score Formula (M) Ion Meas. m/z Pred. m/z Diff (mDa) Diff (ppm) Iso Score DBE

5 11.69 C24 H28 O10 [M-H]- 475.1541 475.1610 -6.9 -14.52 41.85 11.0

Rank Score Formula (M) Ion Meas. m/z Pred. m/z Diff (mDa) Diff (ppm) Iso Score DBE

4 27.11 C21 H24 O7 [M-H]- 387.1404 387.1449 -4.5 -11.62 75.98 10.0

C3H6O3

C28 X19

Event#: 4 MS(E-) Ret. Time : 31.005 Scan# : 3052

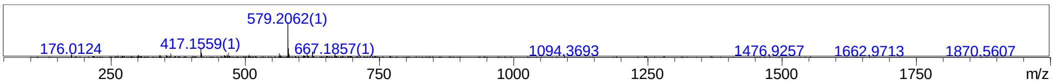

Event#: 5 MS/MS(E-) Ret. Time : 31.005 Scan# : 3053 Precursor : 417.1560 Cutoff : 115

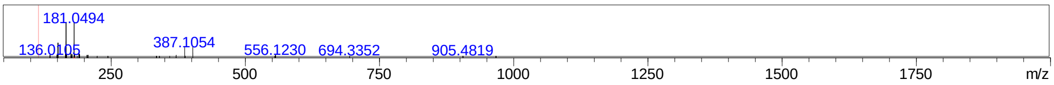

Rank Score Formula (M) Ion Meas. m/z Pred. m/z Diff (mDa) Diff (ppm) Iso Score DBE

2 73.85 C23 H36 N2 O15 [M-H]- 579.2062 579.2043 1.9 3.28 78.31 7.0

Rank Score Formula (M) Ion Meas. m/z Pred. m/z Diff (mDa) Diff (ppm) Iso Score DBE

4 17.39 C17 H26 N2 O10 [M-H]- 417.1559 417.1515 4.4 10.55 45.14 6.0

Rank Score Formula (M) Ion Meas. m/z Pred. m/z Diff (mDa) Diff (ppm) Iso Score DBE

1 54.29 C15 H20 N2 O10 [M-H]- 387.1054 387.1045 0.9 2.32 56.14 7.0

C57 X20 **Hexanedioic acid**

Event#: 1 MS(E+) Ret. Time : 45.935 Scan# : 4525

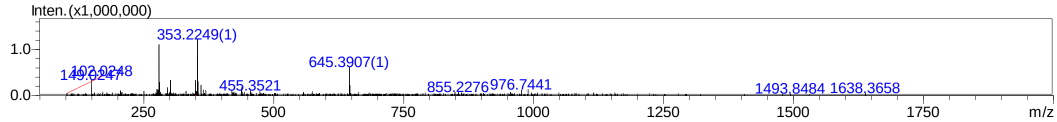

Event#: 4 MS(E-) Ret. Time : 45.935 Scan# : 4528

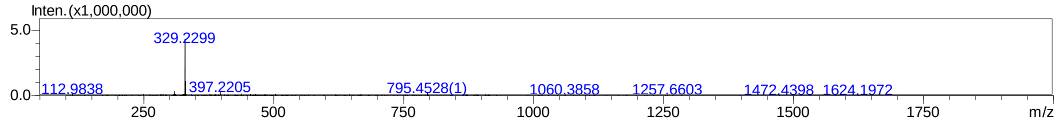

Rank	Score	Formula (M)	Ion	Meas. m/z	Pred. m/z	Diff (mDa)	Diff (ppm)	Iso Score	DBE
1	32.21	C18 H34 O5	[M-H]-	329.2299	329.2333	-3.4	-10.33	82.34	2.0

C63 X21

Event#: 1 MS(E+) Ret. Time : 50.670 Scan# : 4990

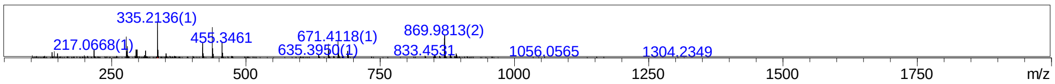

Event#: 4 MS(E-) Ret. Time : 50.670 Scan# : 4992

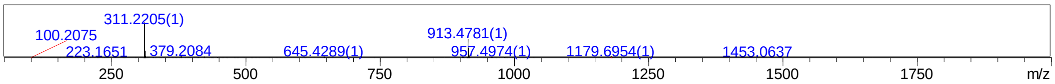

Rank	Score	Formula (M)	Ion	Meas. m/z	Pred. m/z	Diff (mDa)	Diff (ppm)	Iso Score	DBE
1	88.60	C18 H32 O4	[M-H]-	311.2212	311.2228	-1.6	-5.14	100.00	3.0

C66 X21

Event#: 1 MS(E+) Ret. Time : 52.363 Scan# : 5156

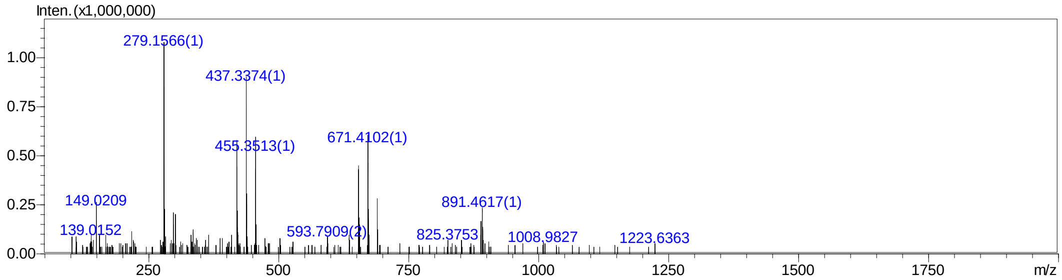

Rank	Score	Formula (M)	Ion	Meas. m/z	Pred. m/z	Diff (mDa)	Diff (ppm)	Iso Score	DBE
1	62.38	C28 H46 O2	[M+Na]+	437.3374	437.3390	-1.6	-3.66	66.82	6.0

C69 X23

Event#: 4 MS(E-) Ret. Time : 69.377 Scan# : 6837

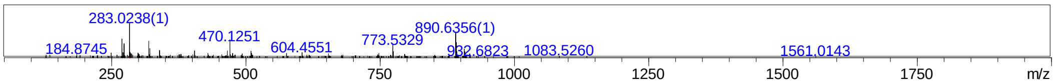

Rank	Score	Formula (M)	Ion	Meas. m/z	Pred. m/z	Diff (mDa)	Diff (ppm)	Iso Score	DBE
1	44.73	C15 H8 O6	[M-H]-	283.0238	283.0248	-1.0	-3.53	47.74	12.0

3. The HRMS data of DBT2 boiling samples

X13+D4+Z7+Y19+6=49

c03 T1

Event#: 4 MS(E-) Ret. Time : 1.293 -> 2.695 Scan# : 127 -> 265

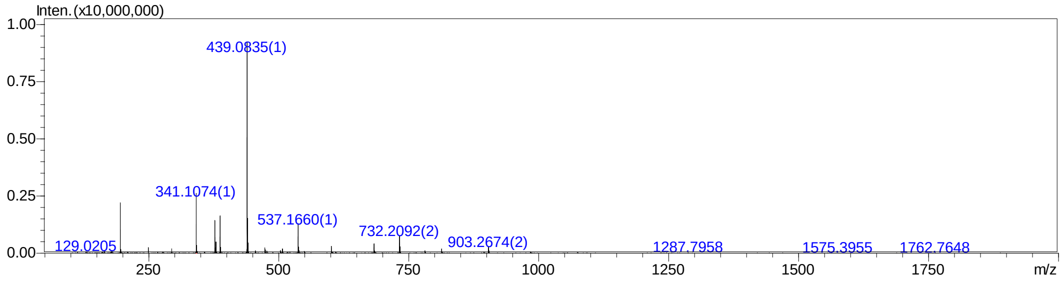

Rank	Score	Formula (M)	Ion	Meas. m/z	Pred. m/z	Diff (mDa)	Diff (ppm)	Iso Score	DBE
1	83.58	C12 H22 O11	[M-H]-	341.1074	341.1089	-1.5	-4.40	91.34	2.0

c01 A1

Event#: 1 MS(E+) Ret. Time : 2.395 Scan# : 232

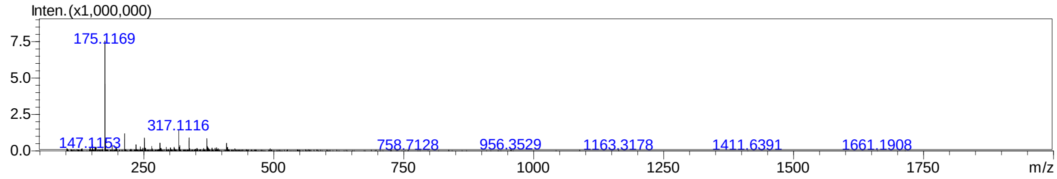

Event#: 4 MS(E-) Ret. Time : 2.395 Scan# : 235

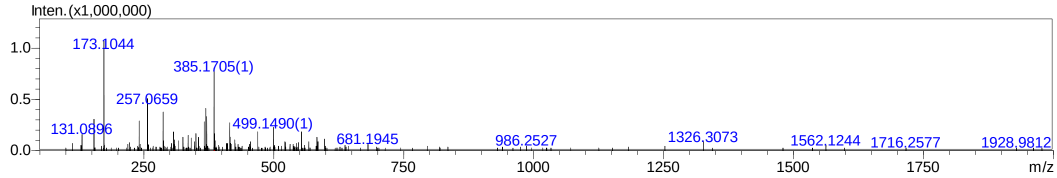

Rank	Score	Formula (M)	Ion	Meas. m/z	Pred. m/z	Diff (mDa)	Diff (ppm)	Iso Score	DBE
1	60.64	C6 H14 N4 O2	[M-H]-	173.1044	173.1044	0.0	0.00	60.64	2.0

c02 A4

Event#: 4 MS(E-) Ret. Time : 2.575 Scan# : 253

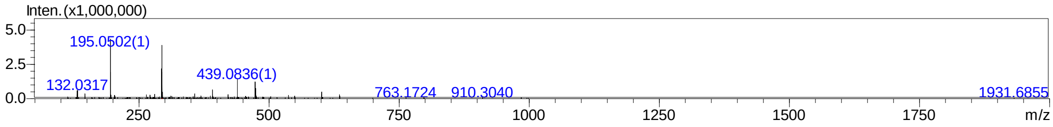

Rank	Score	Formula (M)	Ion	Meas. m/z	Pred. m/z	Diff (mDa)	Diff (ppm)	Iso Score	DBE
1	46.80	C6 H12 O7	[M-H]-	195.0502	195.0510	-0.8	-4.10	50.73	1.0

c04 A2

Event#: 4 MS(E-) Ret. Time : 3.643 Scan# : 358

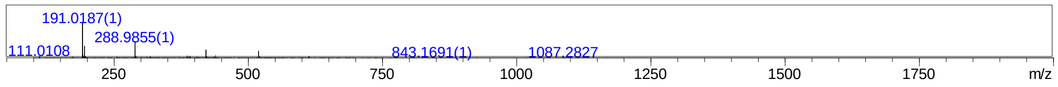

Rank	Score	Formula (M)	Ion	Meas. m/z	Pred. m/z	Diff (mDa)	Diff (ppm)	Iso Score	DBE
1	82.32	C6 H8 O7	[M-H]-	191.0187	191.0197	-1.0	-5.24	93.97	3.0

c09 A3

Event#: 1 MS(E+) Ret. Time : 10.842 Scan# : 1059

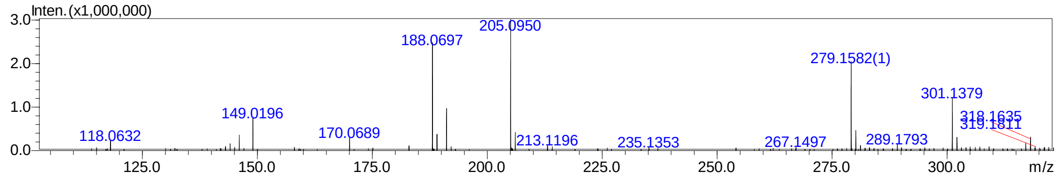

Rank	Score	Formula (M)	Ion	Meas. m/z	Pred. m/z	Diff (mDa)	Diff (ppm)	Iso Score	DBE
1	69.53	C9 H11 N O2	[M+Na]+	188.0674	188.0682	-0.8	-4.25	75.68	5.0

c08

Event#: 1 MS(E+) Ret. Time : 8.643 -> 9.345 Scan# : 843 -> 912

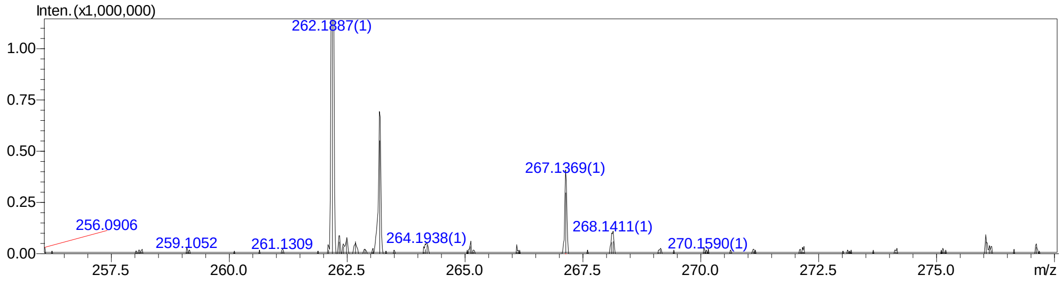

MS/MS(E+) Ret. Time : 8.643 -> 9.345 Scan# : 844 -> 913 Precursor : 267.1328 Cutoff : 73

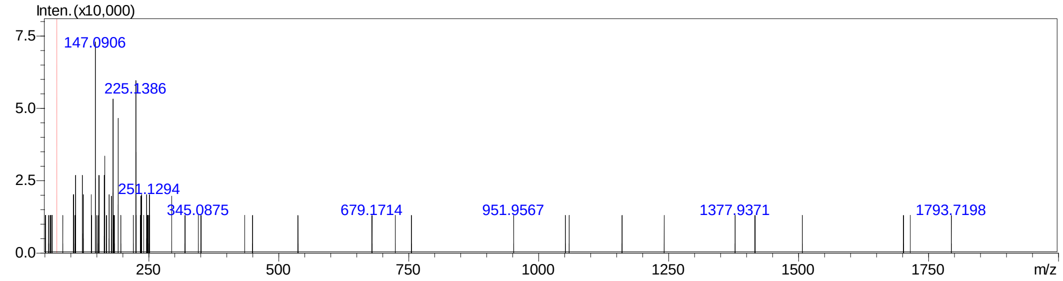

Rank	Score	Formula (M)	Ion	Meas. m/z	Pred. m/z	Diff (mDa)	Diff (ppm)	Iso Score	DBE
1	57.25	C18 H18 O2	[M+H]+	267.1369	267.1380	-1.1	-4.12	62.09	10.0

c012 Y01

Event#: 4 MS(E-) Ret. Time : 21.207 Scan# : 2069

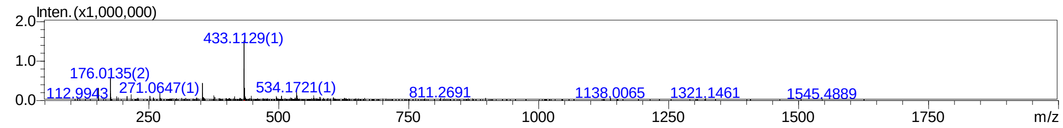

Event#: 5 MS/MS(E-) Ret. Time : 21.207 Scan# : 2070 Precursor : 433.1129 Cutoff : 119

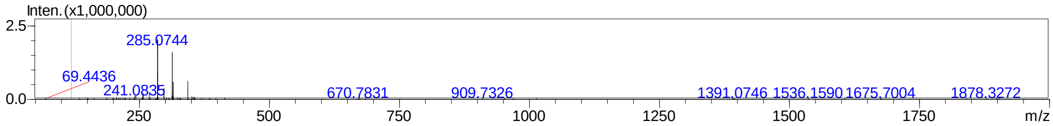

Rank	Score	Formula (M)	Ion	Meas. m/z	Pred. m/z	Diff (mDa)	Diff (ppm)	Iso Score	DBE
1	66.52	C21 H22 O10	[M-H]-	433.1129	433.1140	-1.1	-2.54	69.18	11.0

Rank	Score	Formula (M)	Ion	Meas. m/z	Pred. m/z	Diff (mDa)	Diff (ppm)	Iso Score	DBE
1	19.29	C16 H14 O5	[M-H]-	285.0744	285.0768	-2.4	-8.42	34.56	10.0

C5H10O6

c013 Y2

Event#: 4 MS(E-) Ret. Time : 21.322 Scan# : 2080

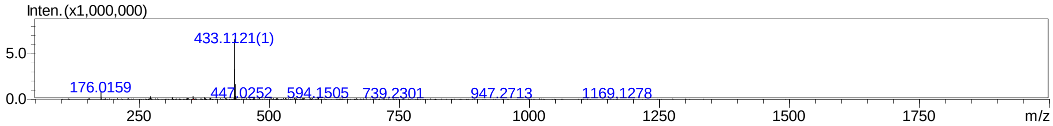

Rank	Score	Formula (M)	Ion	Meas. m/z	Pred. m/z	Diff (mDa)	Diff (ppm)	Iso Score	DBE
1	88.43	C21 H22 O10	[M-H]-	433.1121	433.1140	-1.9	-4.39	96.62	11.0

C15H12O5

c018 Y3 ononin

Event#: 1 MS(E+) Ret. Time : 34.517 Scan# : 3373

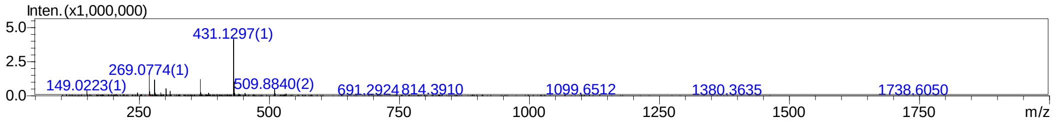

Event#: 2 MS/MS(E+) Ret. Time : 34.517 Scan# : 3374 Precursor : 269.0774 Cutoff : 74

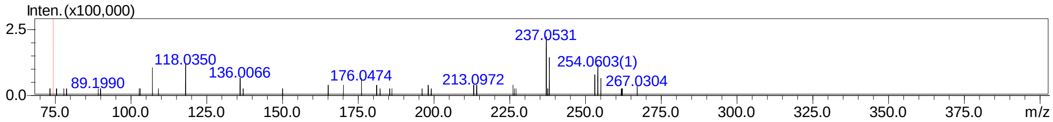

Event#: 4 MS(E-) Ret. Time : 34.517 Scan# : 3376

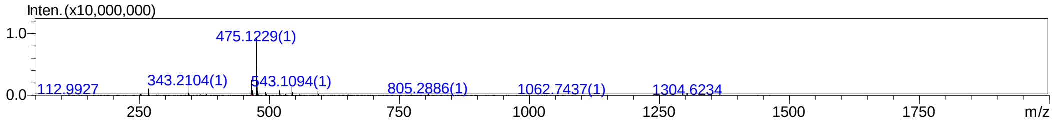

Rank	Score	Formula (M)	Ion	Meas. m/z	Pred. m/z	Diff (mDa)	Diff (ppm)	Iso Score	DBE
2	87.28	C22 H22 O9	[M+H]+	431.1322	431.1337	-1.5	-3.48	93.05	12.0

c019 Y3 $C_{15}H_{12}O_4$

Event#: 4 MS(E-) Ret. Time : 37.480 -> 39.977 Scan# : 3669 -> 3917

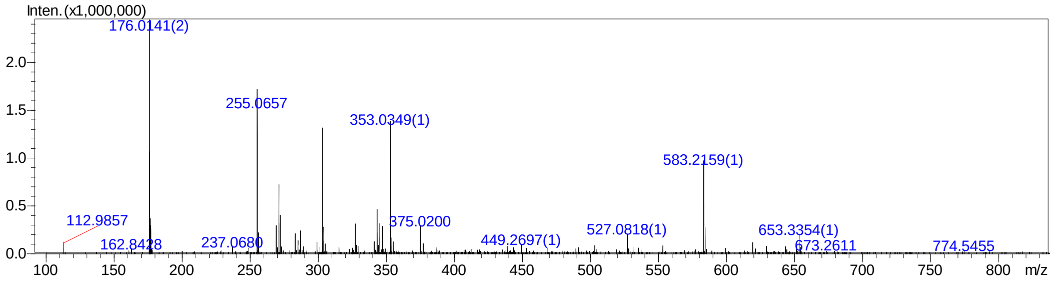

Event#: 5 MS/MS(E-) Ret. Time : 37.480 Scan# : 3670 Precursor : 255.0657 Cutoff : 70

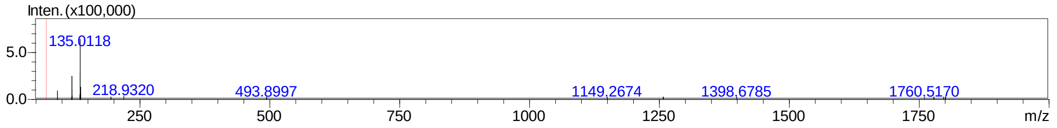

Rank	Score	Formula (M)	Ion	Meas. m/z	Pred. m/z	Diff (mDa)	Diff (ppm)	Iso Score	DBE
1	59.93	C15 H12 O4	[M-H]-	255.0657	255.0663	-0.6	-2.35	62.02	10.0

c20 Y4 calycosin

Event#: 1 MS(E+) Ret. Time : 38.072 Scan# : 3725

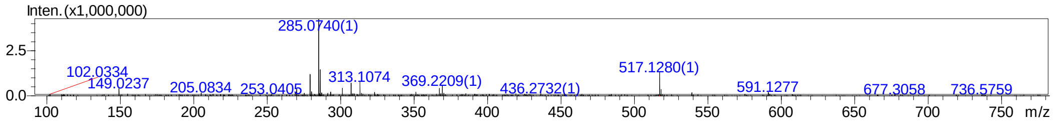

Event#: 2 MS/MS(E+) Ret. Time : 37.952 Scan# : 3714 Precursor : 285.0741 Cutoff : 78

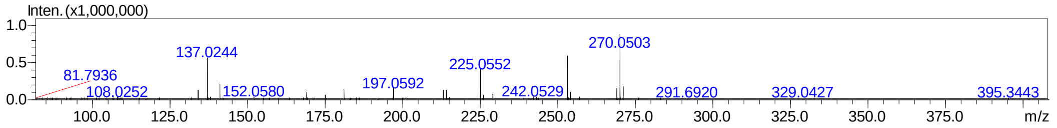

Event#: 4 MS(E-) Ret. Time : 38.072 Scan# : 3728

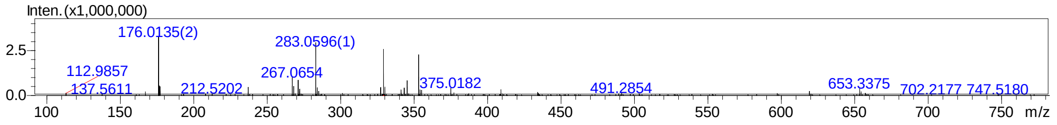

Rank	Score	Formula (M)	Ion	Meas. m/z	Pred. m/z	Diff (mDa)	Diff (ppm)	Iso Score	DBE
1	51.62	C16 H12 O5	[M-H]-	283.0596	283.0612	-1.6	-5.65	61.82	11.0

c025 Y5

Event#: 2 MS/MS(E+) Ret. Time : 41.273 Scan# : 4043 Precursor : 287.0885 Cutoff : 79

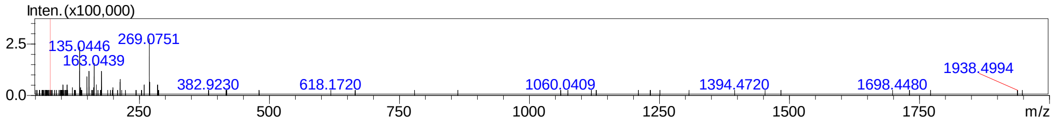

Event#: 4 MS(E-) Ret. Time : 41.273 Scan# : 4045

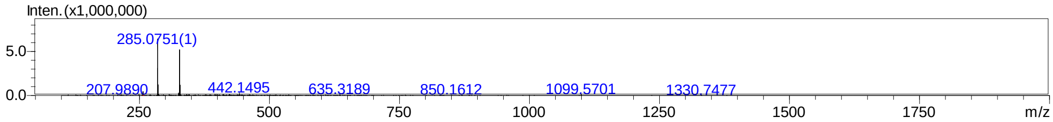

Event#: 5 MS/MS(E-) Ret. Time : 41.222 Scan# : 4040 Precursor : 285.0750 Cutoff : 78

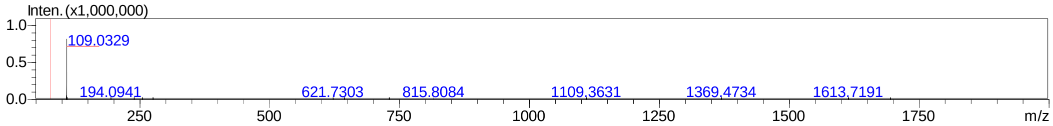

Rank	Score	Formula (M)	Ion	Meas. m/z	Pred. m/z	Diff (mDa)	Diff (ppm)	Iso Score	DBE
1	47.28	C16 H14 O5	[M-H]-	285.0751	285.0768	-1.7	-5.96	58.80	10.0

c021 Y7

Event#: 4 MS(E-) Ret. Time : 39.917 Scan# : 3911

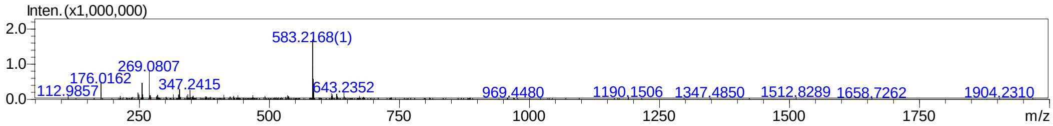

Event#: 5 MS/MS(E-) Ret. Time : 39.917 Scan# : 3912 Precursor : 269.0807 Cutoff : 74

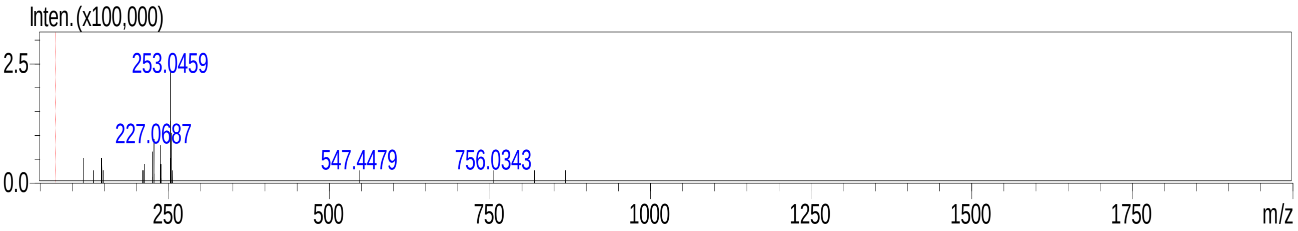

Rank	Score	Formula (M)	Ion	Meas. m/z	Pred. m/z	Diff (mDa)	Diff (ppm)	Iso Score	DBE
1	40.72	C16 H14 O4	[M-H]-	269.0807	269.0819	-1.2	-4.46	44.58	10.0

c022 Y8

Event#: 4 MS(E-) Ret. Time : 39.977 Scan# : 3917

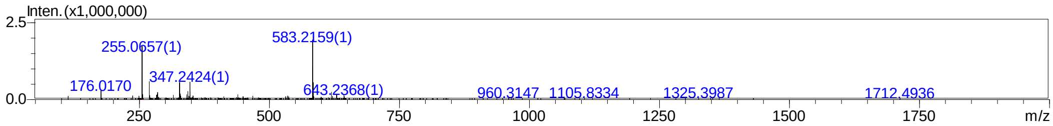

Event#: 5 MS/MS(E-) Ret. Time : 39.977 Scan# : 3918 Precursor : 255.0657 Cutoff : 70

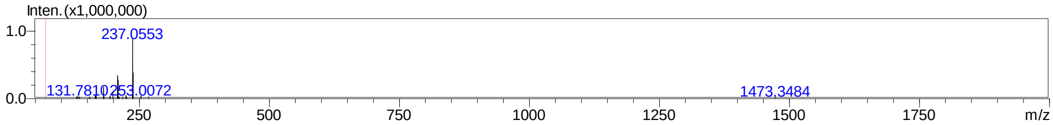

Rank	Score	Formula (M)	Ion	Meas. m/z	Pred. m/z	Diff (mDa)	Diff (ppm)	Iso Score	DBE
1	52.28	C15 H12 O4	[M-H]-	255.0657	255.0663	-0.6	-2.35	54.10	10.0

C15 H12 O4

c025 Y9

Event#: 4 MS(E-) Ret. Time : 41.273 Scan# : 4045

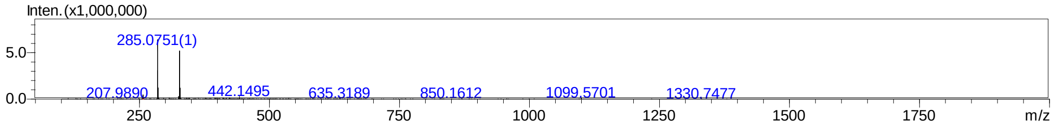

Rank	Score	Formula (M)	Ion	Meas. m/z	Pred. m/z	Diff (mDa)	Diff (ppm)	Iso Score	DBE
1	47.28	C16 H14 O5	[M-H]-	285.0751	285.0768	-1.7	-5.96	58.80	10.0

c026 Y10（genistein）

Event#: 4 MS(E-) Ret. Time : 41.772 Scan# : 4094

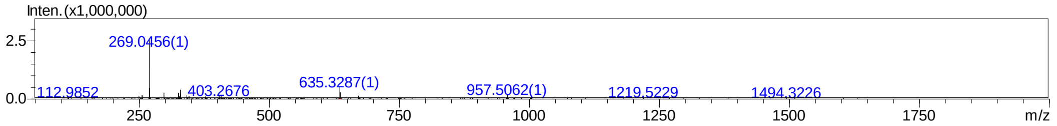

Rank	Score	Formula (M)	Ion	Meas. m/z	Pred. m/z	Diff (mDa)	Diff (ppm)	Iso Score	DBE
1	60.17	C15 H10 O5	[M-H]-	269.0456	269.0455	0.1	0.37	60.17	11.0

Event#: 5 MS/MS(E-) Ret. Time : 41.712 Scan# : 4089 Precursor : 269.0527 Cutoff : 74

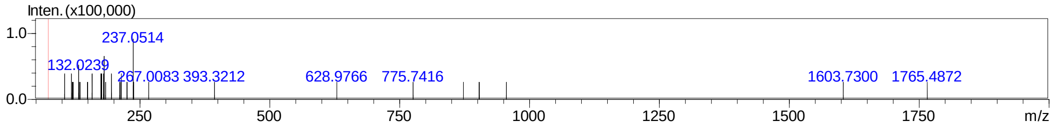

c027 Y11 isomer of calycosin

Event#: 4 MS(E-) Ret. Time : 42.243 Scan# : 4141

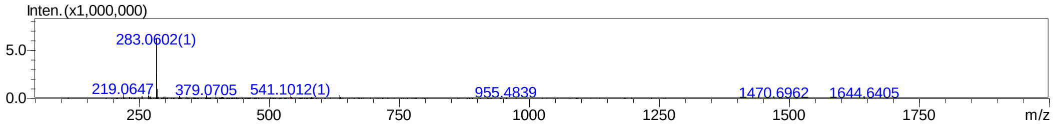

Rank	Score	Formula (M)	Ion	Meas. m/z	Pred. m/z	Diff (mDa)	Diff (ppm)	Iso Score	DBE
1	67.47	C16 H12 O5	[M-H]-	283.0602	283.0612	-1.0	-3.53	72.02	11.0

Event#: 5 MS/MS(E-) Ret. Time : 42.363 Scan# : 4154 Precursor : 283.0599 Cutoff : 78

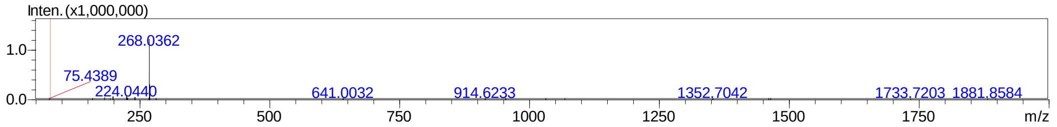

c030 Y12

Event#: 4 MS(E-) Ret. Time : 44.690 Scan# : 4383

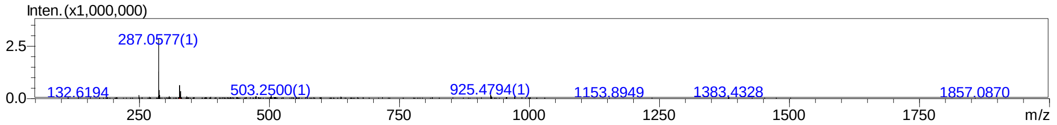

Rank	Score	Formula (M)	Ion	Meas. m/z	Pred. m/z	Diff (mDa)	Diff (ppm)	Iso Score	DBE

1	57.35	C15 H12 O6	[M-H]-	287.0577	287.0561	1.6	5.57	68.03	10.0

c031 Y13 Liquiritigenin C15 H12 O4

Event#: 4 MS(E-) Ret. Time : 44.982 Scan# : 4412

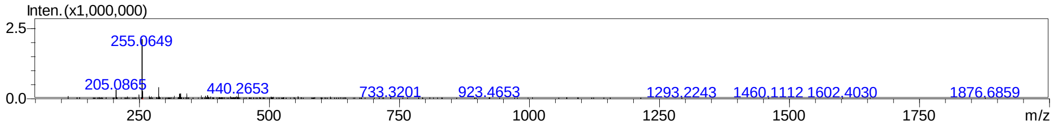

Rank	Score	Formula (M)	Ion	Meas. m/z	Pred. m/z	Diff (mDa)	Diff (ppm)	Iso Score	DBE
1	38.99	C15 H12 O4	[M-H]-	255.0649	255.0663	-1.4	-5.49	45.81	10.0

Event#: 5 MS/MS(E-) Ret. Time : 44.982 Scan# : 4413 Precursor : 256.0677 Cutoff : 70

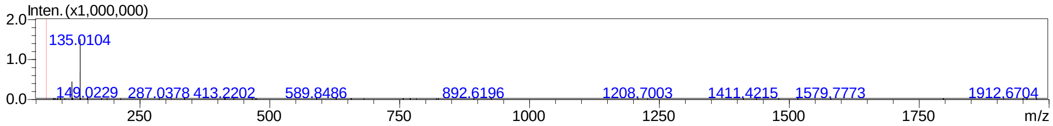

c032 Y14 formononetin

Event#: 1 MS(E+) Ret. Time : 45.393 Scan# : 4450

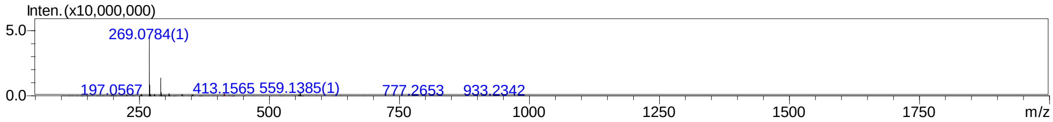

Event#: 2 MS/MS(E+) Ret. Time : 45.453 Scan# : 4457 Precursor : 269.0790 Cutoff : 74

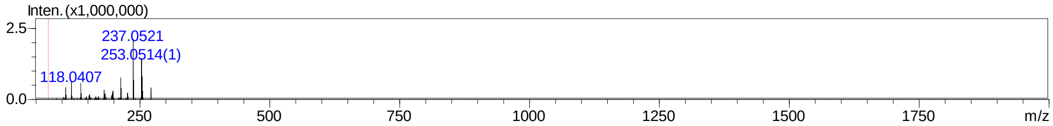

Event#: 4 MS(E-) Ret. Time : 45.393 Scan# : 4453

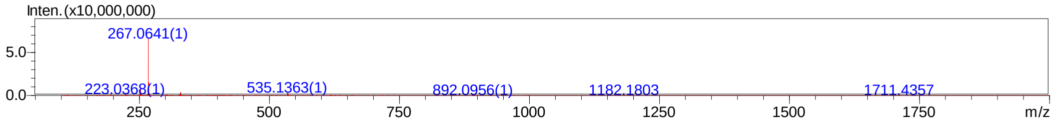

Event#: 5 MS/MS(E-) Ret. Time : 45.333 Scan# : 4448 Precursor : 267.2028 Cutoff : 73

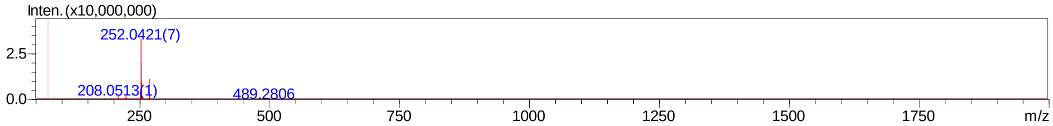

Rank	Score	Formula (M)	Ion	Meas. m/z	Pred. m/z	Diff (mDa)	Diff (ppm)	Iso Score	DBE
1	39.84	C16 H12 O4	[M+H]+	269.0784	269.0808	-2.4	-8.92	78.43	11.0

c033 Y15 astrapterocarpan

Event#: 1 MS(E+) Ret. Time : 45.745 Scan# : 4485

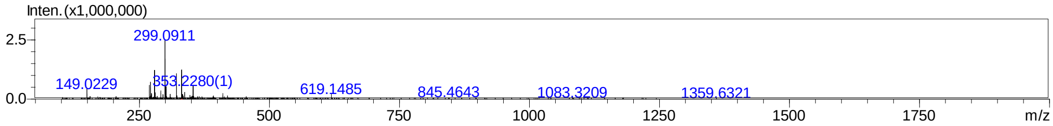

Event#: 2 MS/MS(E+) Ret. Time : 45.805 Scan# : 4492 Precursor : 299.0888 Cutoff :

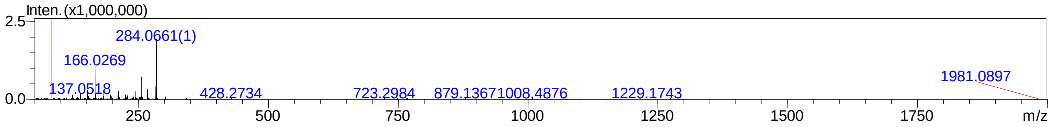

82

Rank	Score	Formula (M)	Ion	Meas. m/z	Pred. m/z	Diff (mDa)	Diff (ppm)	Iso Score	DBE
1	38.67	C17 H14 O5	[M+H]+	299.0911	299.0914	-0.3	-1.00	38.67	11.0

C17H16O5

c034 Y16 wogonin

Event#: 4 MS(E-) Ret. Time : 46.080 Scan# : 4520

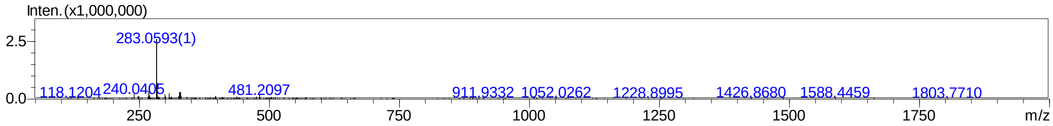

Event#: 5 MS/MS(E-) Ret. Time : 46.028 Scan# : 4516 Precursor : 283.0606 Cutoff :

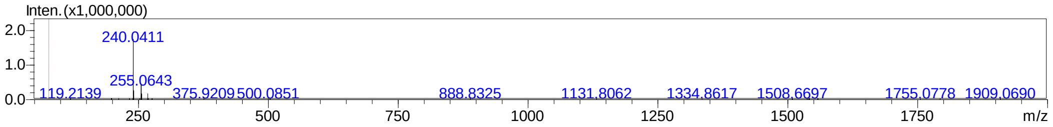

78

Rank	Score	Formula (M)	Ion	Meas. m/z	Pred. m/z	Diff (mDa)	Diff (ppm)	Iso Score	DBE
1	34.44	C16 H12 O5	[M-H]-	283.0599	283.0612	-1.3	-4.59	37.84	11.0

c036 Y17

Event#: 1 MS(E+) Ret. Time : 47.772 Scan# : 4685

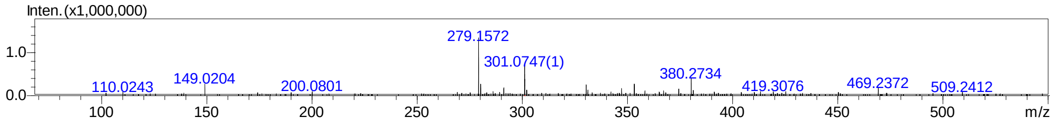

vent#: 2 MS/MS(E+) Ret. Time : 47.772 Scan# : 4686 Precursor : 301.0748 Cutoff : 83

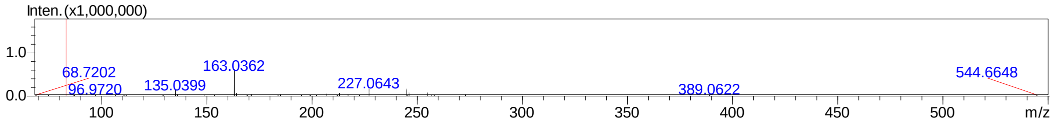

Event#: 4 MS(E-) Ret. Time : 47.772 Scan# : 4688

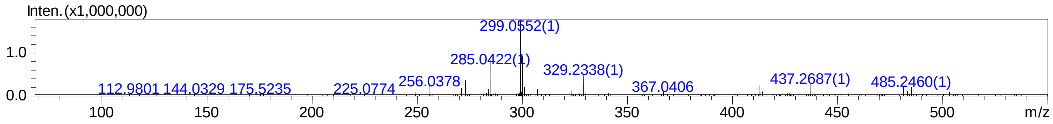

Rank	Score	Formula (M)	Ion	Meas. m/z	Pred. m/z	Diff (mDa)	Diff (ppm)	Iso Score	DBE
1	59.51	C16 H12 O6	[M-H]-	299.0552	299.0561	-0.9	-3.01	62.66	11.0

C16 H12 O5

c033 Y6

Event#: 1 MS(E+) Ret. Time : 45.805 Scan# : 4491

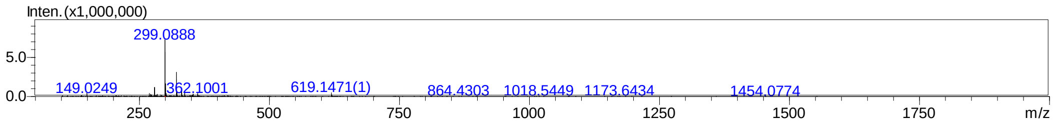

Event#: 2 MS/MS(E+) Ret. Time : 45.805 Scan# : 4492 Precursor : 299.0888 Cutoff : 82

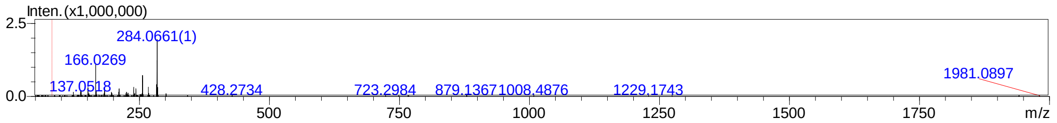

Rank	Score	Formula (M)	Ion	Meas. m/z	Pred. m/z	Diff (mDa)	Diff (ppm)	Iso Score	DBE
1	88.60	C15 H16 O5	[M+Na]+	299.0888	299.0890	-0.2	-0.67	88.60	8.0
2	50.90	C17 H14 O5	[M+H]+	299.0888	299.0914	-2.6	-8.69	95.86	11.0

c040 Y18

Event#: 1 MS(E+) Ret. Time : 50.435 Scan# : 4947

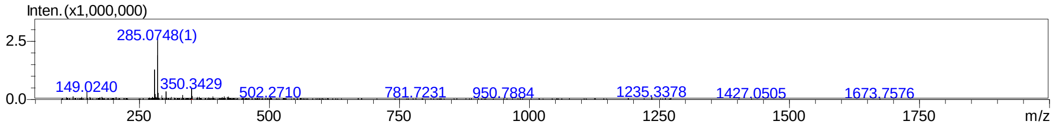

Event#: 2 MS/MS(E+) Ret. Time : 50.375 Scan# : 4942 Precursor : 285.0750 Cutoff : 78

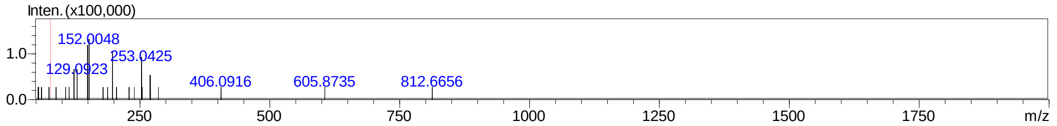

Event#: 4 MS(E-) Ret. Time : 50.375 Scan# : 4944

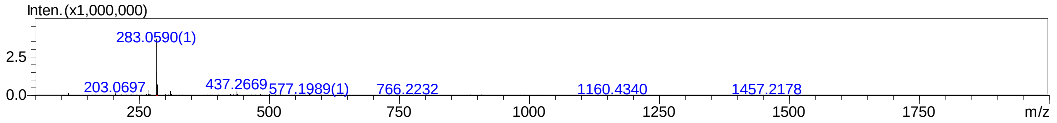

Event#: 5 MS/MS(E-) Ret. Time : 50.375 Scan# : 4945 Precursor : 283.0590 Cutoff : 78

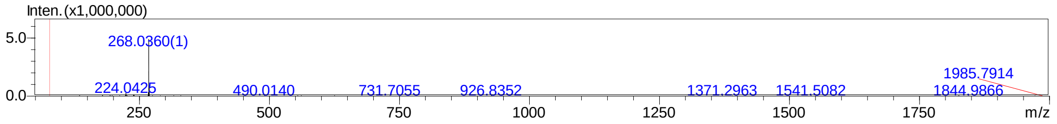

Rank	Score	Formula (M)	Ion	Meas. m/z	Pred. m/z	Diff (mDa)	Diff (ppm)	Iso Score	DBE
1	79.60	C16 H12 O5	[M+H]+	285.0748	285.0758	-1.0	-3.51	84.93	11.0

c023 Z1 Ba

Event#: 4 MS(E-) Ret. Time : 39.745 Scan# : 3894

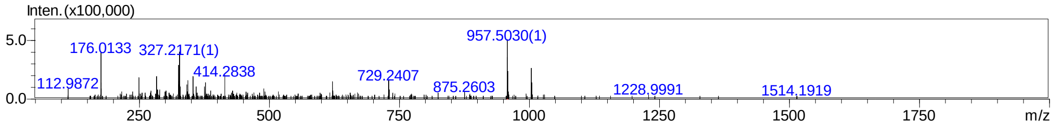

Event#: 5 MS/MS(E-) Ret. Time : 39.745 Scan# : 3895 Precursor : 957.5031 Cutoff : 265

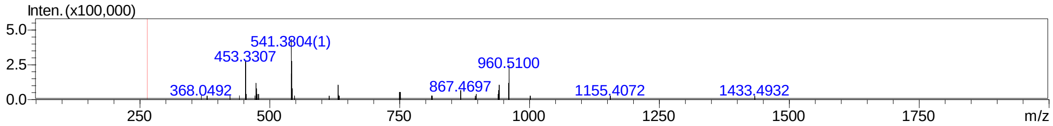

Rank	Score	Formula (M)	Ion	Meas. m/z	Pred. m/z	Diff (mDa)	Diff (ppm)	Iso Score	DBE
1	51.55	C48 H78 O19	[M-H]-	957.5030	957.5065	-3.5	-3.66	55.22	10.0

c024 Z2 Akebia saponin D

Event#: 4 MS(E-) Ret. Time : 40.903 Scan# : 4008

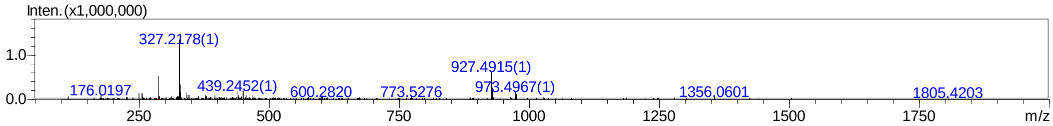

Rank	Score	Formula (M)	Ion	Meas. m/z	Pred. m/z	Diff (mDa)	Diff (ppm)	Iso Score	DBE
1	63.25	C47 H76 O18	[M-H]-	927.4915	927.4959	-4.4	-4.74	69.77	10.0

c029 Z3 人参皂苷 Ro

Event#: 4 MS(E-) Ret. Time : 43.197 Scan# : 4235

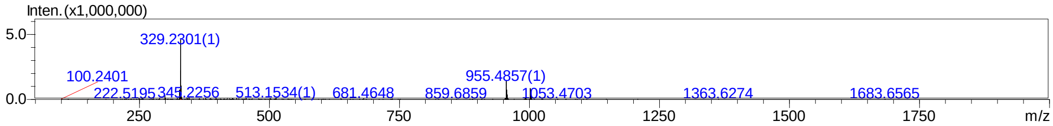

Rank	Score	Formula (M)	Ion	Meas. m/z	Pred. m/z	Diff (mDa)	Diff (ppm)	Iso Score	DBE
4	50.61	C48 H76 O19	[M-H]-	955.4857	955.4908	-5.1	-5.34	58.45	11.0

c035 Z4 Bb

Event#: 4 MS(E-) Ret. Time : 47.592 Scan# : 4670

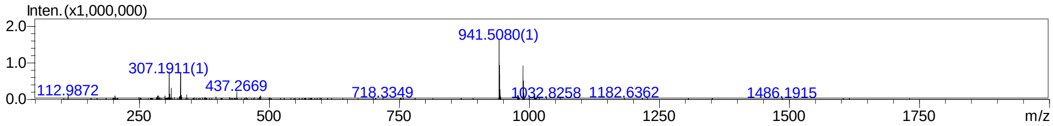

Event#: 5 MS/MS(E-) Ret. Time : 47.592 Scan# : 4671 Precursor : 941.5083 Cutoff : 260

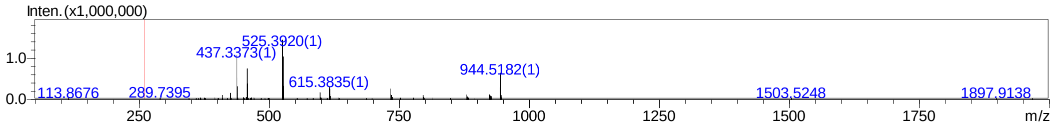

Rank	Score	Formula (M)	Ion	Meas. m/z	Pred. m/z	Diff (mDa)	Diff (ppm)	Iso Score	DBE
1	82.82	C48 H78 O18	[M-H]-	941.5080	941.5115	-3.5	-3.72	88.86	10.0

c037 Z5

Event#: 4 MS(E-) Ret. Time : 47.943 Scan# : 4705

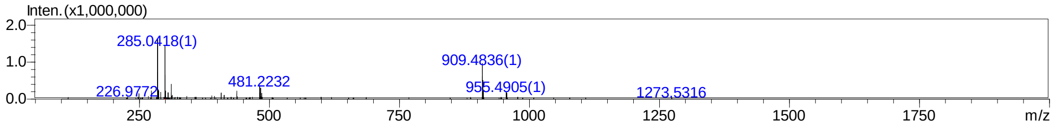

Rank Score Formula (M) Ion Meas. m/z Pred. m/z Diff (mDa) Diff (ppm) Iso Score DBE

4 59.79 C47 H74 O17 [M-H]- 909.4836 909.4853 -1.7 -1.87 61.12 11.0

c038 Z6 Castaraleside H

Event#: 4 MS(E-) Ret. Time : 48.347 Scan# : 4745

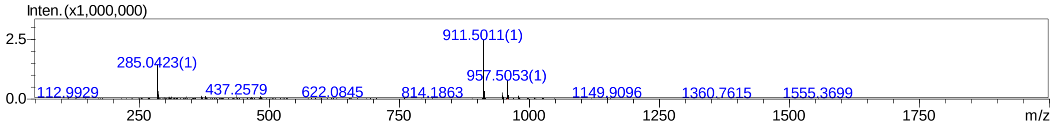

Rank Score Formula (M) Ion Meas. m/z Pred. m/z Diff (mDa) Diff (ppm) Iso Score DBE

1 78.66 C46 H74 O15 [M+HCOO]- 911.5011 911.5010 0.1 0.11 78.66 10.0

G—I? ? ?

Rank Score Formula (M) Ion Meas. m/z Pred. m/z Diff (mDa) Diff (ppm) Iso Score DBE

1 86.89 C46 H74 O15 [M+HCOO]- 911.5012 911.5010 0.2 0.22 86.89 10.0

2 86.89 C47 H76 O17 [M-H]- 911.5012 911.5010 0.2 0.22 86.89 10.0

c039 Z7

Event#: 4 MS(E-) Ret. Time : 50.065 Scan# : 4914

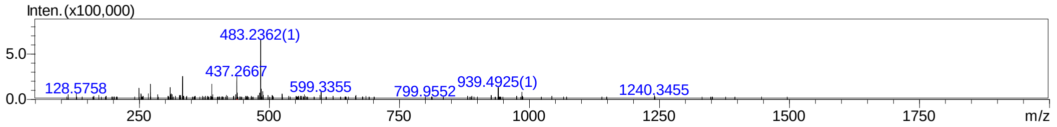

Rank Score Formula (M) Ion Meas. m/z Pred. m/z Diff (mDa) Diff (ppm) Iso Score DBE

1 33.31 C48 H76 O18 [M-H]- 939.4925 939.4959 -3.4 -3.62 35.64 11.0

c042 D1 A

Event#: 1 MS(E+) Ret. Time : 51.560 Scan# : 5058

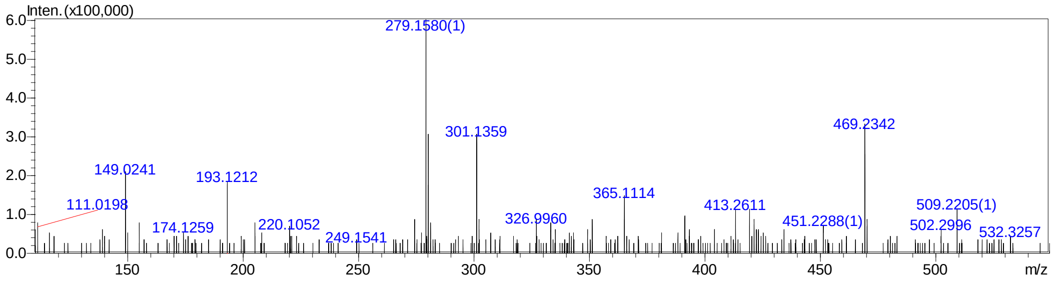

MS/MS(E+) Ret. Time : 51.560 Scan# : 5059 Precursor : 193.1212 Cutoff : 53

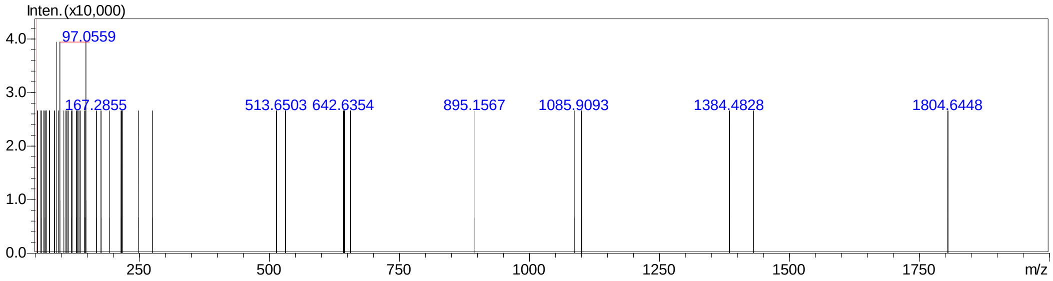

Rank	Score	Formula (M)	Ion	Meas. m/z	Pred. m/z	Diff (mDa)	Diff (ppm)	Iso Score	DBE
1	71.30	C12 H16 O2	[M+H]+	193.1212	193.1223	-1.1	-5.70	85.91	5.0

c043 D2

Event#: 1 MS(E+) Ret. Time : 55.212 Scan# : 5417

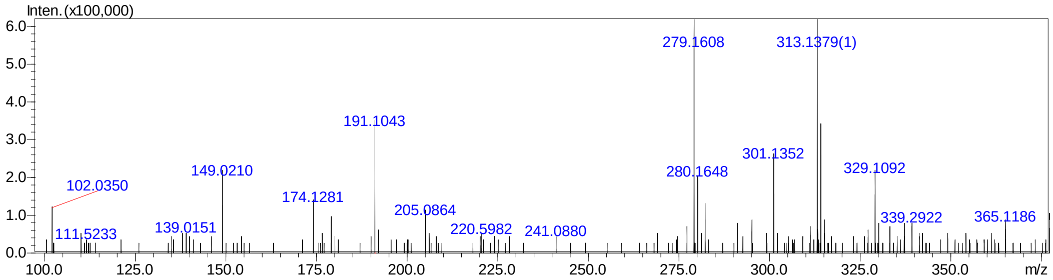

Rank	Score	Formula (M)	Ion	Meas. m/z	Pred. m/z	Diff (mDa)	Diff (ppm)	Iso Score	DBE
1	85.23	C10 H16 O2	[M+Na]+	191.1043	191.1043	0.0	0.00	85.23	3.0
2	4.70	C12 H14 O2	[M+H]+	191.1043	191.1067	-2.4	-12.56	14.18	6.0

c044 D3 Z-

Event#: 1 MS(E+) Ret. Time : 59.250 Scan# : 5814

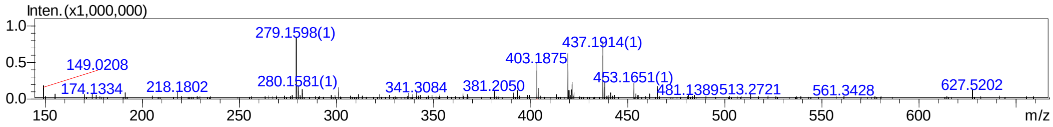

Event#: 2 MS/MS(E+) Ret. Time : 59.250 Scan# : 5815 Precursor : 403.1875 Cutoff : 111

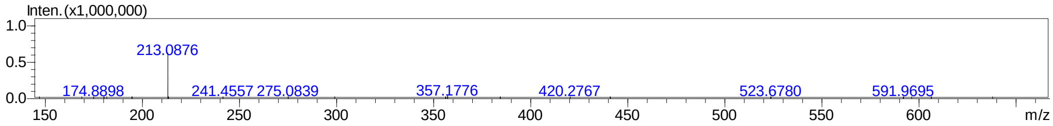

Rank	Score	Formula (M)	Ion	Meas. m/z	Pred. m/z	Diff (mDa)	Diff (ppm)	Iso Score	DBE
1	0.00	C12 H14 O2	[M+Na]+	213.0876	213.0886	-1.0	-4.69	0.00	6.0

c045 D4 E-

Event#: 1 MS(E+) Ret. Time : 61.765 Scan# : 6063

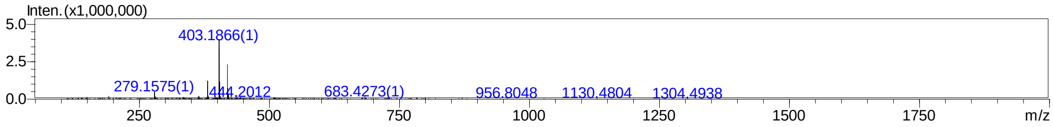

Event#: 2 MS/MS(E+) Ret. Time : 61.765 Scan# : 6064 Precursor : 381.2059 Cutoff : 105

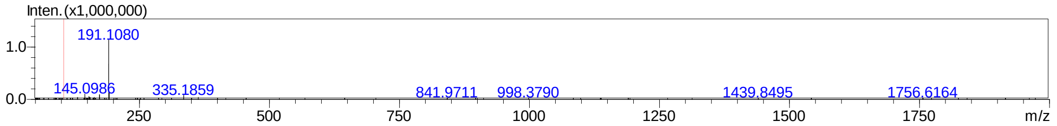

Rank	Score	Formula (M)	Ion	Meas. m/z	Pred. m/z	Diff (mDa)	Diff (ppm)	Iso Score	DBE
1	38.11	C12 H14 O2	[M+H]+	191.1080	191.1067	1.3	6.80	52.94	6.0

Rank	Score	Formula (M)	Ion	Meas. m/z	Pred. m/z	Diff (mDa)	Diff (ppm)	Iso Score	DBE
1	42.13	C12 H14 O2	[M+Na]+	213.0893	213.0886	0.7	3.29	44.69	6.0

c05 X1

Event#: 1 MS(E+) Ret. Time : 4.477 Scan# : 437

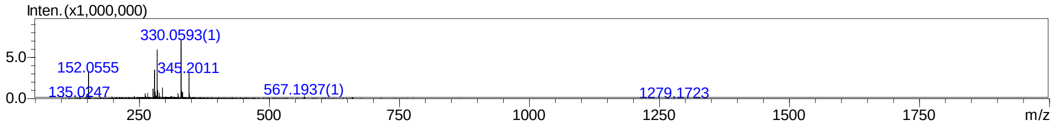

Event#: 4 MS(E-) Ret. Time : 4.477 Scan# : 440

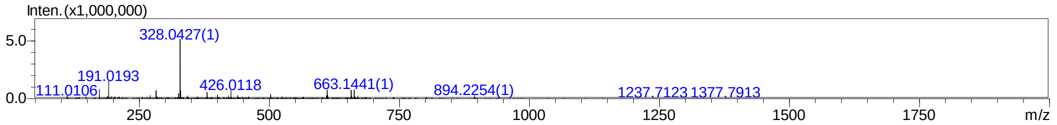

Rank	Score	Formula (M)	Ion	Meas. m/z	Pred. m/z	Diff (mDa)	Diff (ppm)	Iso Score	DBE
1	99.45	C11 H11 N3 O9	[M-H]-	328.0427	328.0423	0.4	1.22	100.00	8.0

c06 X2

Event#: 1 MS(E+) Ret. Time : 6.172 Scan# : 602

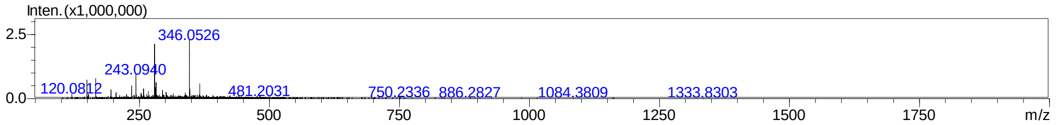

Event#: 4 MS(E-) Ret. Time : 6.172 Scan# : 605

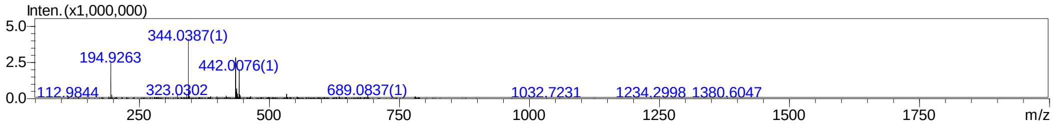

Rank	Score	Formula (M)	Ion	Meas. m/z	Pred. m/z	Diff (mDa)	Diff (ppm)	Iso Score	DBE
1	81.82	C11 H11 N3 O10	[M-H]-	344.0387	344.0372	1.5	4.36	89.32	8.0

c07 X4

Event#: 1 MS(E+) Ret. Time : 9.120 Scan# : 890

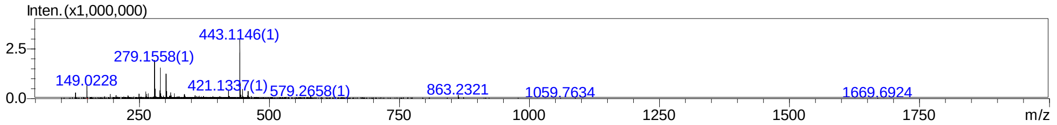

Event#: 4 MS(E-) Ret. Time : 9.120 Scan# : 892

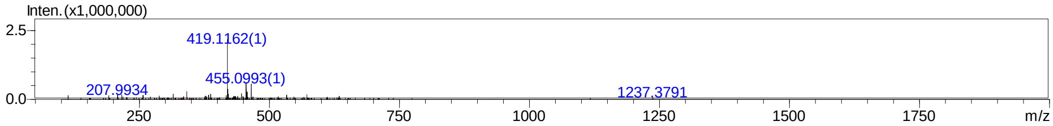

Rank	Score	Formula (M)	Ion	Meas. m/z	Pred. m/z	Diff (mDa)	Diff (ppm)	Iso Score	DBE
1	84.70	C17 H24 O12	[M+Na]+	443.1146	443.1160	-1.4	-3.16	89.54	6.0

c010 X5

Event#: 1 MS(E+) Ret. Time : 12.765 Scan# : 1250

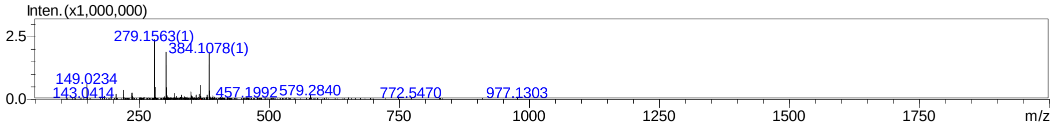

Event#: 4 MS(E-) Ret. Time : 12.765 Scan# : 1253

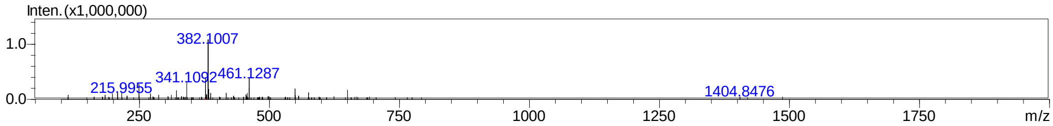

Rank	Score	Formula (M)	Ion	Meas. m/z	Pred. m/z	Diff (mDa)	Diff (ppm)	Iso Score	DBE
1	63.30	C13 H21 N O12	[M-H]-	382.1007	382.0991	1.6	4.19	68.79	4.0

c011 X6

Event#: 1 MS(E+) Ret. Time : 13.840 Scan# : 1355

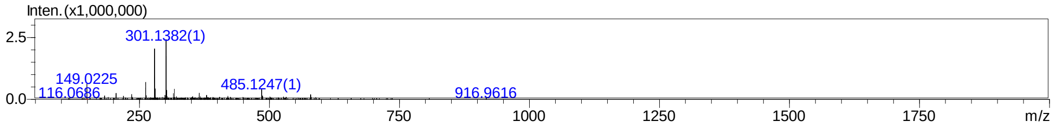

Event#: 4 MS(E-) Ret. Time : 13.840 Scan# : 1358

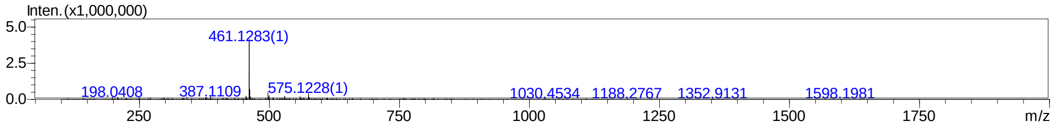

Rank	Score	Formula (M)	Ion	Meas. m/z	Pred. m/z	Diff (mDa)	Diff (ppm)	Iso Score	DBE
1	69.28	C19 H26 O13	[M-H]-	461.1283	461.1301	-1.8	-3.90	74.69	7.0

c014 X7+

Event#: 4 MS(E-) Ret. Time : 22.020 Scan# : 2147

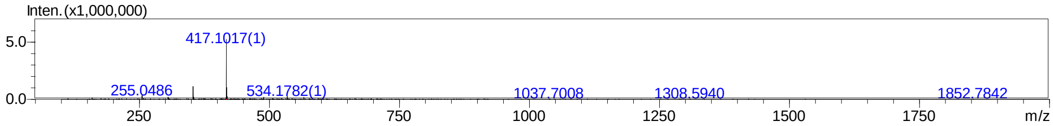

Event#: 5 MS/MS(E-) Ret. Time : 22.020 Scan# : 2148 Precursor : 417.1018 Cutoff : 115

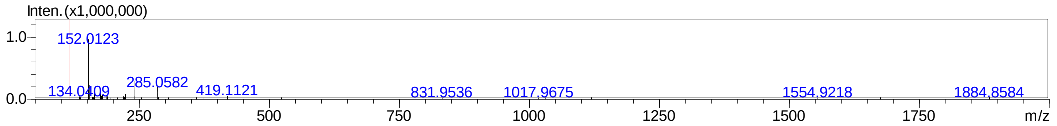

Rank Score Formula (M) Ion Meas. m/z Pred. m/z Diff (mDa) Diff (ppm) Iso Score DBE

1 86.57 C17 H22 O12 [M-H]- 417.1017 417.1038 -2.1 -5.03 96.51 7.0

Rank Score Formula (M) Ion Meas. m/z Pred. m/z Diff (mDa) Diff (ppm) Iso Score DBE

4 9.29 C12 H14 O8 [M-H]- 285.0582 285.0616 -3.4 -11.93 26.65 6.0

c015 X8 =

Event#: 4 MS(E-) Ret. Time : 23.018 Scan# : 2243

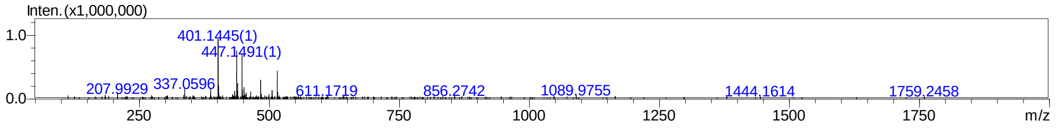

Event#: 5 MS/MS(E-) Ret. Time : 23.018 Scan# : 2244 Precursor : 401.1445 Cutoff : 111

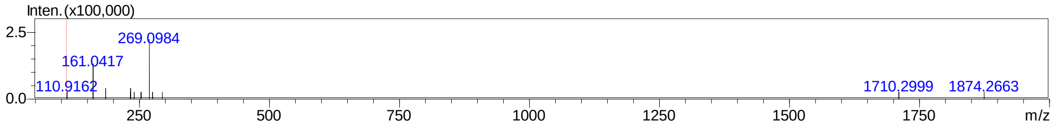

Rank Score Formula (M) Ion Meas. m/z Pred. m/z Diff (mDa) Diff (ppm) Iso Score DBE

1 86.04 C18 H26 O10 [M-H]- 401.1445 401.1453 -0.8 -1.99 88.22 6.0

C5H8O4 xylan

c016 X9 =

Event#: 1 MS(E+) Ret. Time : 23.080 Scan# : 2246

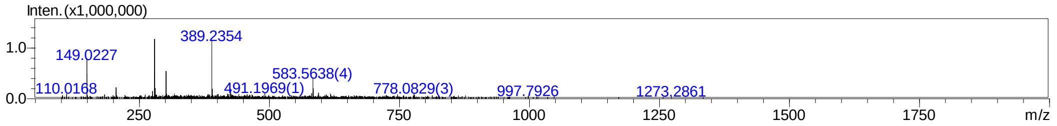

Rank Score Formula (M) Ion Meas. m/z Pred. m/z Diff (mDa) Diff (ppm) Iso Score DBE

1 59.93 C23 H32 O5 [M+H]+ 389.2325 389.2323 0.2 0.51 59.93 8.0

c017 X10 =

Event#: 1 MS(E+) Ret. Time : 26.998 Scan# : 2628

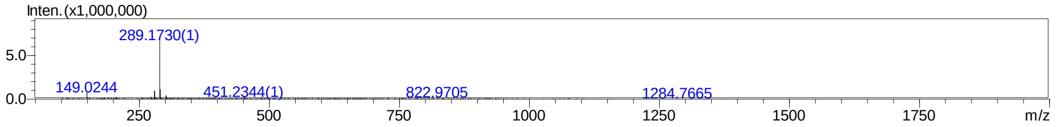

Event#: 4 MS(E-) Ret. Time : 26.998 Scan# : 2630

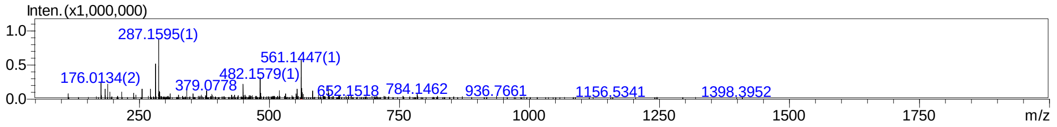

Rank	Score	Formula (M)	Ion	Meas. m/z	Pred. m/z	Diff (mDa)	Diff (ppm)	Iso Score	DBE
2	35.86	C13 H24 N2 O5	[M+H]+	289.1730	289.1758	-2.8	-9.68	83.01	3.0

Rank	Score	Formula (M)	Ion	Meas. m/z	Pred. m/z	Diff (mDa)	Diff (ppm)	Iso Score	DBE
1	54.79	C13 H24 N2 O5	[M-H]-	287.1595	287.1612	-1.7	-5.92	67.81	3.0

c028 X11 **Hexanedioic acid** =

Event#: 1 MS(E+) Ret. Time : 42.965 Scan# : 4209

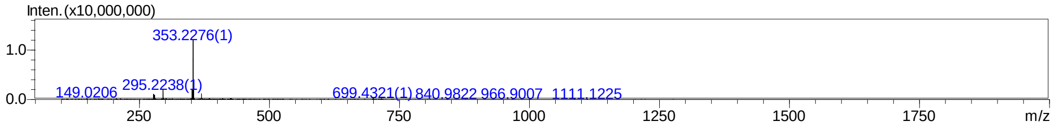

Event#: 4 MS(E-) Ret. Time : 42.965 Scan# : 4212

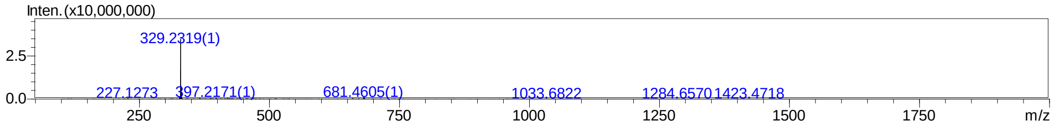

Rank	Score	Formula (M)	Ion	Meas. m/z	Pred. m/z	Diff (mDa)	Diff (ppm)	Iso Score	DBE
3	55.25	C18 H34 O5	[M+Na]+	353.2276	353.2298	-2.2	-6.23	71.10	2.0
1	80.09	C18 H34 O5	[M-H]-	329.2319	329.2333	-1.4	-4.25	87.18	2.0

c041 X12 =

Event#: 1 MS(E+) Ret. Time : 50.667 Scan# : 4970

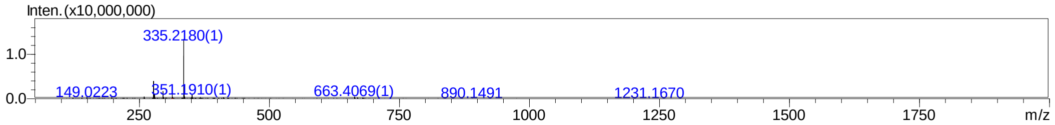

Event#: 4 MS(E-) Ret. Time : 50.667 Scan# : 4973

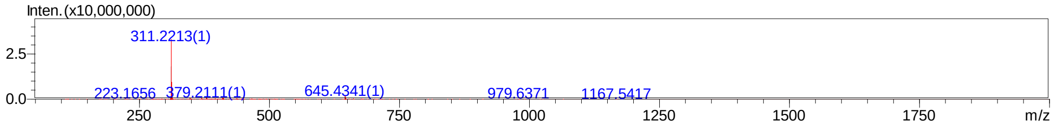

Rank	Score	Formula (M)	Ion	Meas. m/z	Pred. m/z	Diff (mDa)	Diff (ppm)	Iso Score	DBE
2	79.68	C18 H32 O4	[M+Na]+	335.2180	335.2193	-1.3	-3.88	85.86	3.0
3	51.89	C19 H28 N4	[M+Na]+	335.2180	335.2206	-2.6	-7.76	83.15	8.0

Rank	Score	Formula (M)	Ion	Meas. m/z	Pred. m/z	Diff (mDa)	Diff (ppm)	Iso Score	DBE
1	75.98	C18 H32 O4	[M-H]-	311.2213	311.2228	-1.5	-4.82	84.01	3.0
4	44.87	C19 H28 N4	[M-H]-	311.2213	311.2241	-2.8	-9.00	89.75	8.0

c046 X13

Event#: 4 MS(E-) Ret. Time : 69.093 Scan# : 6786

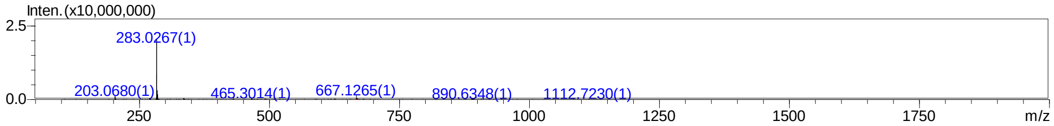

Rank	Score	Formula (M)	Ion	Meas. m/z	Pred. m/z	Diff (mDa)	Diff (ppm)	Iso Score	DBE
1	61.26	C15 H8 O6	[M-H]-	283.0257	283.0248	0.9	3.18	64.79	12.0

4. The HRMS data of DBT1 urine samples

M1

Event#: 1 MS(E+) Ret. Time : 4.130 Scan# : 403

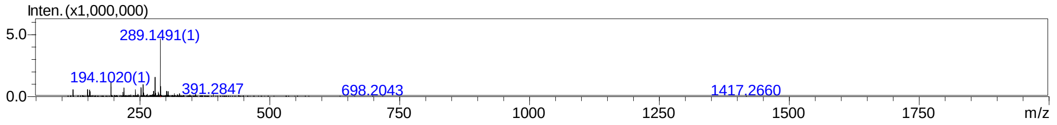

vent#: 2 MS/MS(E+) Ret. Time : 4.130 Scan# : 404 Precursor : 289.1490 Cutoff : 80

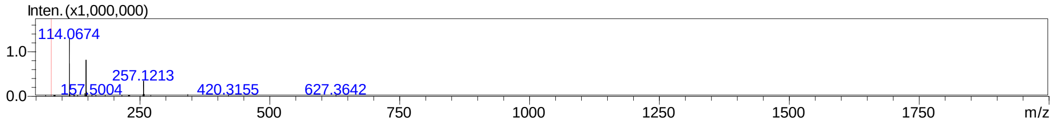

Event#: 4 MS(E-) Ret. Time : 4.130 Scan# : 406

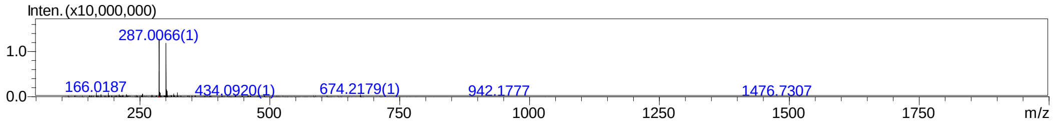

Rank	Score	Formula (M)	Ion	Meas. m/z	Pred. m/z	Diff (mDa)	Diff (ppm)	Iso Score	DBE
1	45.75	C10 H8 O10	[M-H]-	287.0066	287.0045	2.1	7.32	68.49	7.0

Rank	Score	Formula (M)	Ion	Meas. m/z	Pred. m/z	Diff (mDa)	Diff (ppm)	Iso Score	DBE
1	10.69	C17 H20 O4	[M+H]+	289.1491	289.1434	5.7	19.71	75.76	8.0

M2

Event#: 4 MS(E-) Ret. Time : 28.110 Scan# : 2764

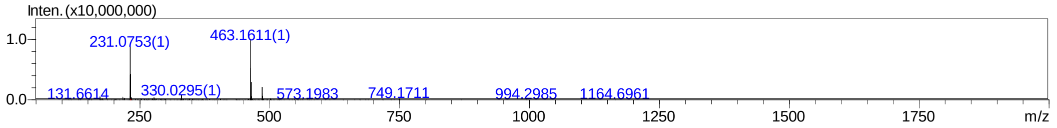

Event#: 5 MS/MS(E-) Ret. Time : 28.110 Scan# : 2765 Precursor : 231.9906 Cutoff : 64

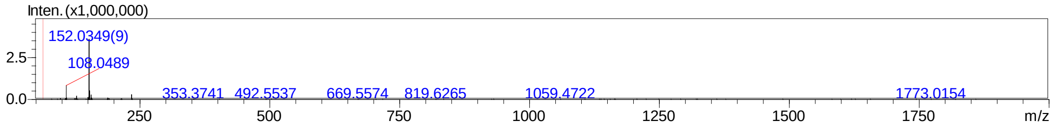

Rank	Score	Formula (M)	Ion	Meas. m/z	Pred. m/z	Diff (mDa)	Diff (ppm)	Iso Score	DBE
1	67.52	C12 H12 N2 O3	[M+H]+	233.0909	233.0921	-1.2	-5.15	76.29	8.0

M3

Event#: 4 MS(E-) Ret. Time : 28.848 Scan# : 2838

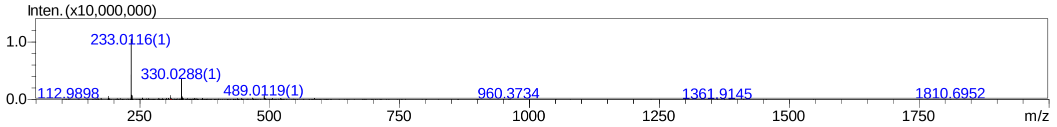

Rank	Score	Formula (M)	Ion	Meas. m/z	Pred. m/z	Diff (mDa)	Diff (ppm)	Iso Score	DBE
1	51.23	C12 H2 N4 O2	[M-H]-	233.0116	233.0105	1.1	4.72	56.48	14.0

M4

Event#: 1 MS(E+) Ret. Time : 29.415 Scan# : 2891

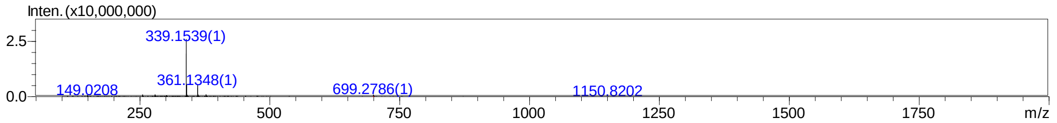

Event#: 4 MS(E-) Ret. Time : 29.415 Scan# : 2894

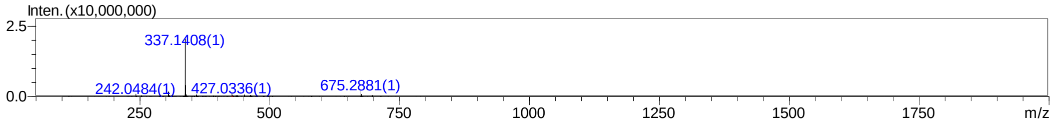

Rank	Score	Formula (M)	Ion	Meas. m/z	Pred. m/z	Diff (mDa)	Diff (ppm)	Iso Score	DBE
1	67.69	C16 H22 N2 O6	[M-H]-	337.1407	337.1405	0.2	0.59	67.69	7.0

M5

Event#: 4 MS(E-) Ret. Time : 31.338 Scan# : 3083

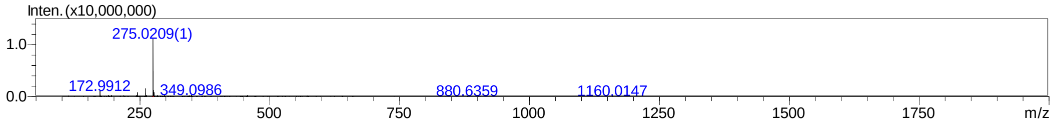

Event#: 5 MS/MS(E-) Ret. Time : 31.338 Scan# : 3084 Precursor : 275.0209 Cutoff : 76

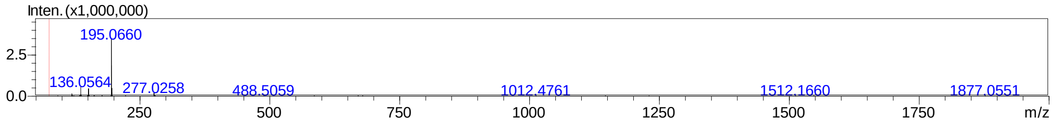

Rank	Score	Formula (M)	Ion	Meas. m/z	Pred. m/z	Diff (mDa)	Diff (ppm)	Iso Score	DBE
1	57.28	C13 H8 O7	[M-H]-	275.0209	275.0197	1.2	4.36	62.53	10.0

M6

Event#: 4 MS(E-) Ret. Time : 32.678 Scan# : 3216

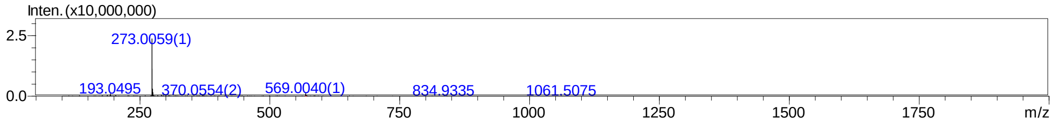

Event#: 5 MS/MS(E-) Ret. Time : 32.858 Scan# : 3235 Precursor : 273.0063 Cutoff : 75

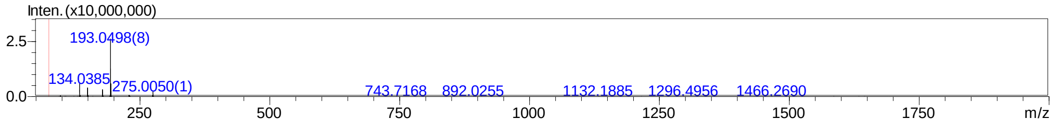

Rank	Score	Formula (M)	Ion	Meas. m/z	Pred. m/z	Diff (mDa)	Diff (ppm)	Iso Score	DBE
1	39.52	C13 H6 O7	[M-H]-	273.0063	273.0041	2.2	8.06	66.53	11.0

Rank	Score	Formula (M)	Ion	Meas. m/z	Pred. m/z	Diff (mDa)	Diff (ppm)	Iso Score	DBE
1	50.55	C15 H12 O5	[M+H]+	273.0750	273.0758	-0.8	-2.93	53.11	10.0

M7

Event#: 1 MS(E+) Ret. Time : 33.038 Scan# : 3249

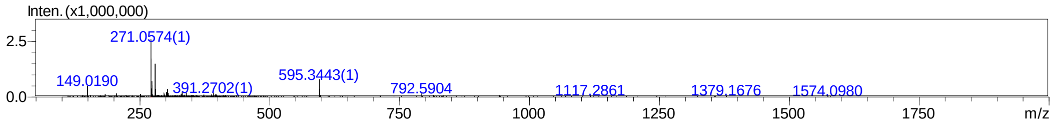

Event#: 2 MS/MS(E+) Ret. Time : 33.038 Scan# : 3250 Precursor : 271.0574 Cutoff : 75

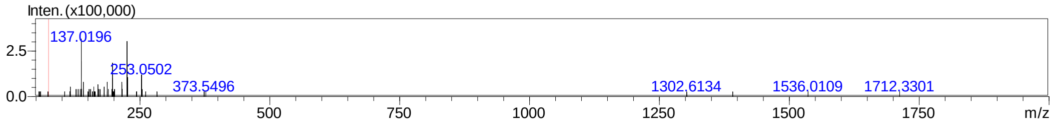

Rank	Score	Formula (M)	Ion	Meas. m/z	Pred. m/z	Diff (mDa)	Diff (ppm)	Iso Score	DBE
3	29.45	C15 H10 O5	[M+H]+	271.0585	271.0601	-1.6	-5.90	36.36	11.0

M8

Event#: 4 MS(E-) Ret. Time : 36.155 Scan# : 3559

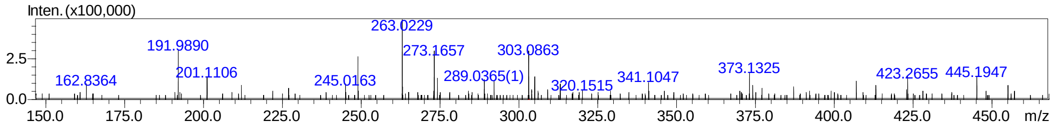

Event#: 5 MS/MS(E-) Ret. Time : 36.155 Scan# : 3560 Precursor : 303.0865 Cutoff : 83

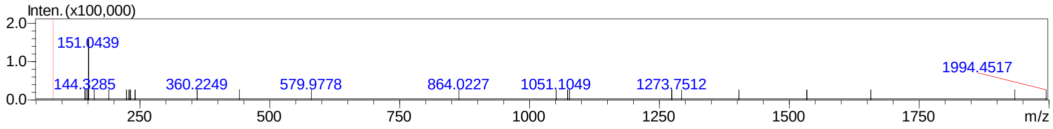

Rank	Score	Formula (M)	Ion	Meas. m/z	Pred. m/z	Diff (mDa)	Diff (ppm)	Iso Score	DBE
1	15.77	C16 H16 O6	[M-H]-	303.0863	303.0874	-1.1	-3.63	16.88	9.0
2	15.77	C15 H14 O4	[M+HCOO]-	303.0863	303.0874	-1.1	-3.63	16.88	9.0

M9

Event#: 1 MS(E+) Ret. Time : 36.705 Scan# : 3611

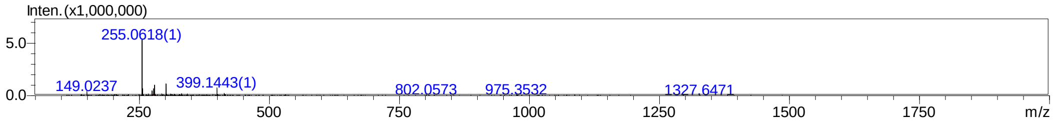

Event#: 4 MS(E-) Ret. Time : 36.705 Scan# : 3613

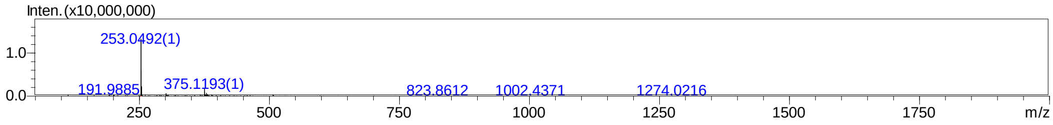

Rank	Score	Formula (M)	Ion	Meas. m/z	Pred. m/z	Diff (mDa)	Diff (ppm)	Iso Score	DBE
1	73.78	C15 H10 O4	[M-H]-	253.0492	253.0506	-1.4	-5.53	87.11	11.0

M10

Event#: 4 MS(E-) Ret. Time : 36.808 Scan# : 3624

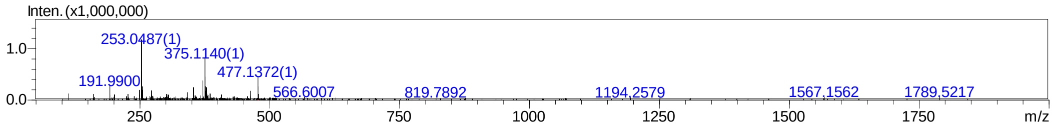

Event#: 5 MS/MS(E-) Ret. Time : 36.808 Scan# : 3625 Precursor : 477.1372 Cutoff : 132

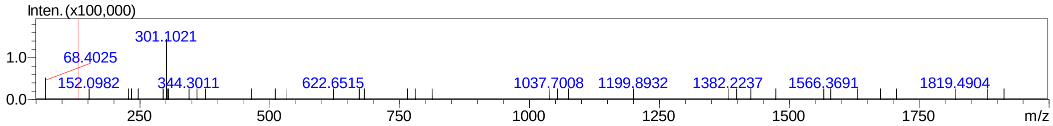

Rank	Score	Formula (M)	Ion	Meas. m/z	Pred. m/z	Diff (mDa)	Diff (ppm)	Iso Score	DBE
4	43.96	C23 H26 O11	[M-H]-	477.1372	477.1402	-3.0	-6.29	57.01	11.0

M11

Event#: 4 MS(E-) Ret. Time : 36.868 Scan# : 3629

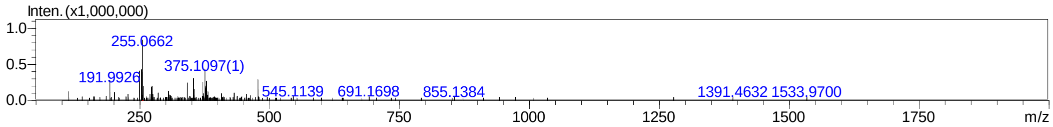

Event#: 5 MS/MS(E-) Ret. Time : 36.868 Scan# : 3630 Precursor : 255.0662 Cutoff : 70

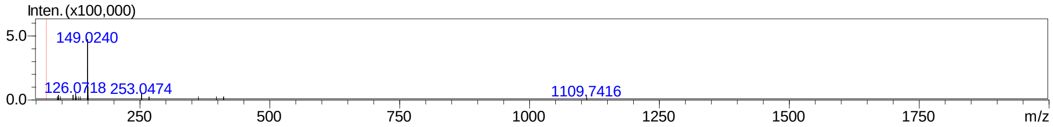

Rank	Score	Formula (M)	Ion	Meas. m/z	Pred. m/z	Diff (mDa)	Diff (ppm)	Iso Score	DBE
1	53.81	C15 H12 O4	[M-H]-	255.0662	255.0663	-0.1	-0.39	53.81	10.0

M13

Event#: 4 MS(E-) Ret. Time : 37.435 Scan# : 3686

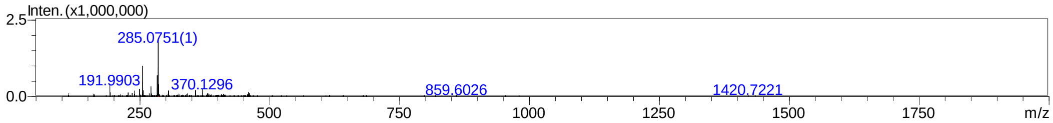

Event#: 5 MS/MS(E-) Ret. Time : 37.435 Scan# : 3687 Precursor : 285.0751 Cutoff : 78

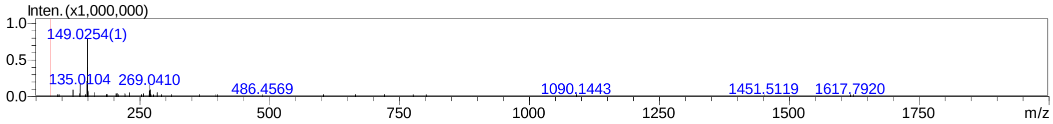

Rank	Score	Formula (M)	Ion	Meas. m/z	Pred. m/z	Diff (mDa)	Diff (ppm)	Iso Score	DBE
1	54.35	C16 H14 O5	[M-H]-	285.0751	285.0768	-1.7	-5.96	67.60	10.0

M14

Event#: 4 MS(E-) Ret. Time : 37.195 Scan# : 3662

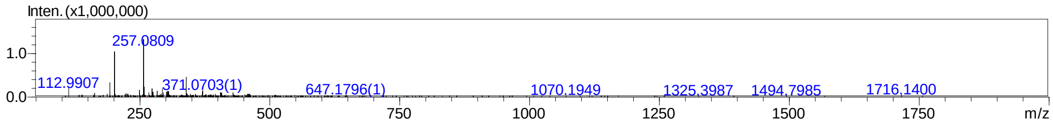

Event#: 5 MS/MS(E-) Ret. Time : 37.195 Scan# : 3663 Precursor : 257.0808 Cutoff : 71

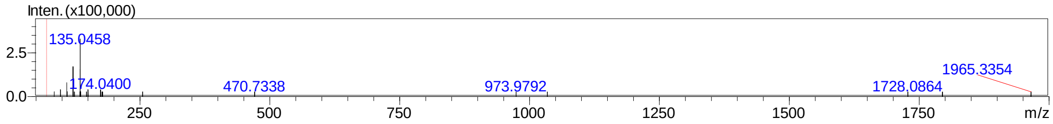

Rank	Score	Formula (M)	Ion	Meas. m/z	Pred. m/z	Diff (mDa)	Diff (ppm)	Iso Score	DBE
1	24.88	C15 H14 O4	[M-H]-	257.0809	257.0819	-1.0	-3.89	26.81	9.0

M15

Event#: 1 MS(E+) Ret. Time : 38.070 Scan# : 3746

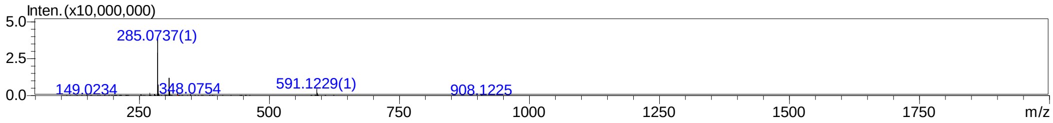

vent#: 4 MS(E-) Ret. Time : 38.070 Scan# : 3749

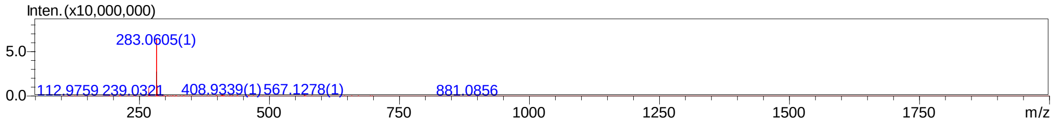

Rank	Score	Formula (M)	Ion	Meas. m/z	Pred. m/z	Diff (mDa)	Diff (ppm)	Iso Score	DBE
1	71.00	C16 H12 O5	[M-H]-	283.0605	283.0612	-0.7	-2.47	73.71	11.0

M16

Event#: 1 MS(E+) Ret. Time : 38.362 Scan# : 3775

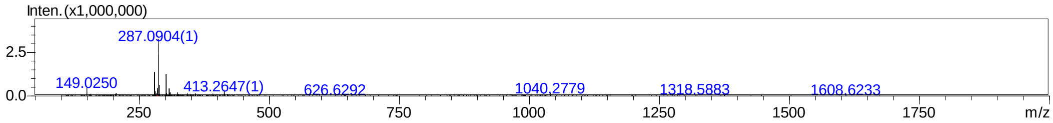

Event#: 4 MS(E-) Ret. Time : 38.362 Scan# : 3778

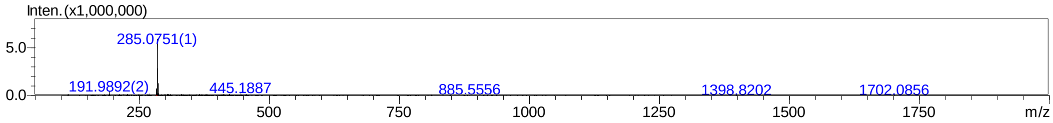

Rank	Score	Formula (M)	Ion	Meas. m/z	Pred. m/z	Diff (mDa)	Diff (ppm)	Iso Score	DBE
1	48.12	C16 H14 O5	[M-H]-	285.0751	285.0768	-1.7	-5.96	59.85	10.0

M17

Event#: 4 MS(E-) Ret. Time : 39.367 Scan# : 3876

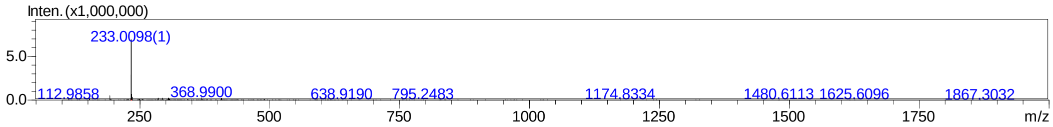

Event#: 5 MS/MS(E-) Ret. Time : 39.367 Scan# : 3877 Precursor : 233.0098 Cutoff : 64

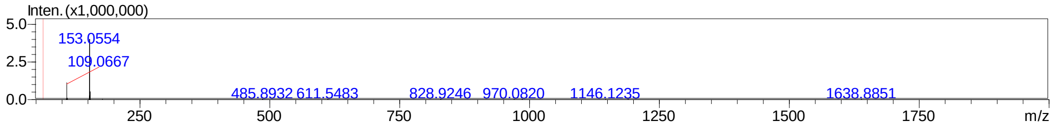

Rank	Score	Formula (M)	Ion	Meas. m/z	Pred. m/z	Diff (mDa)	Diff (ppm)	Iso Score	DBE
1	72.09	C11 H6 O6	[M-H]-	233.0098	233.0092	0.6	2.57	75.04	9.0
4	54.66	C12 H2 N4 O2	[M-H]-	233.0098	233.0105	-0.7	-3.00	57.54	14.0

M18

Event#: 4 MS(E-) Ret. Time : 40.260 Scan# : 3965

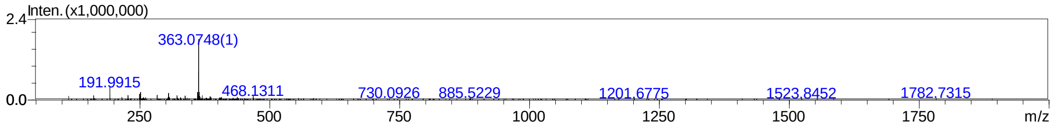

Rank	Score	Formula (M)	Ion	Meas. m/z	Pred. m/z	Diff (mDa)	Diff (ppm)	Iso Score	DBE
2	53.49	C13 H18 O7 S	[M+HCOO]-	363.0748	363.0755	-0.7	-1.93	54.76	5.0
4	40.39	C17 H16 O9	[M-H]-	363.0748	363.0722	2.6	7.16	59.06	10.0
5	40.39	C16 H14 O7	[M+HCOO]-	363.0748	363.0722	2.6	7.16	59.06	10.0

M19

Event#: 4 MS(E-) Ret. Time : 41.755 Scan# : 4112

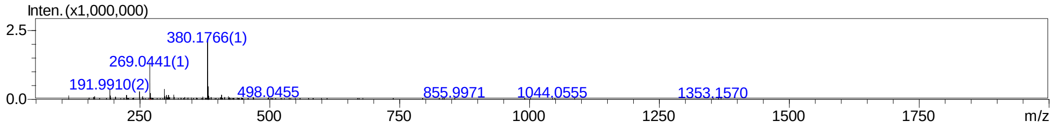

Event#: 5 MS/MS(E-) Ret. Time : 41.755 Scan# : 4113 Precursor : 269.0441 Cutoff : 74

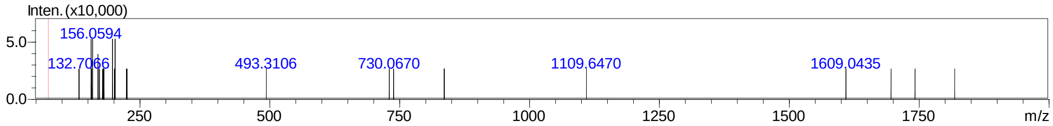

Rank	Score	Formula (M)	Ion	Meas. m/z	Pred. m/z	Diff (mDa)	Diff (ppm)	Iso Score	DBE
1	40.88	C15 H10 O5	[M-H]-	269.0441	269.0455	-1.4	-5.20	46.46	11.0

M20

Event#: 4 MS(E-) Ret. Time : 42.365 Scan# : 4172

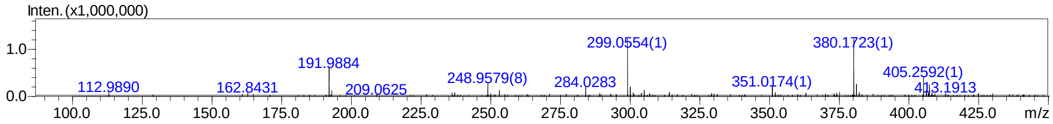

Event#: 5 MS/MS(E-) Ret. Time : 42.365 Scan# : 4173 Precursor : 299.0555 Cutoff : 82

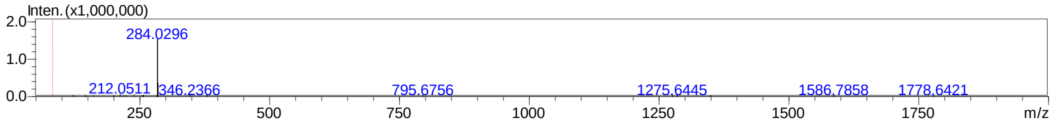

Rank	Score	Formula (M)	Ion	Meas. m/z	Pred. m/z	Diff (mDa)	Diff (ppm)	Iso Score	DBE
1	51.88	C16 H12 O6	[M-H]-	299.0554	299.0561	-0.7	-2.34	53.67	11.0

M21

Event#: 4 MS(E-) Ret. Time : 42.923 Scan# : 4227

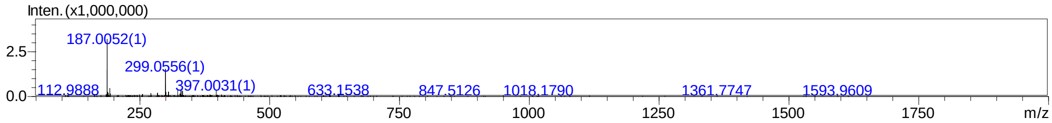

Event#: 5 MS/MS(E-) Ret. Time : 42.923 Scan# : 4228 Precursor : 299.0556 Cutoff : 82

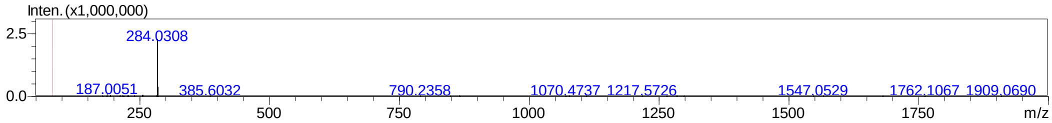

Rank	Score	Formula (M)	Ion	Meas. m/z	Pred. m/z	Diff (mDa)	Diff (ppm)	Iso Score	DBE
1	52.63	C16 H12 O6	[M-H]-	299.0556	299.0561	-0.5	-1.67	53.53	11.0

M22

Event#: 4 MS(E-) Ret. Time : 44.315 Scan# : 4364

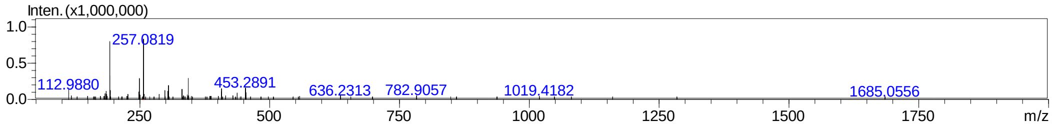

Event#: 5 MS/MS(E-) Ret. Time : 44.315 Scan# : 4365 Precursor : 257.0819 Cutoff : 71

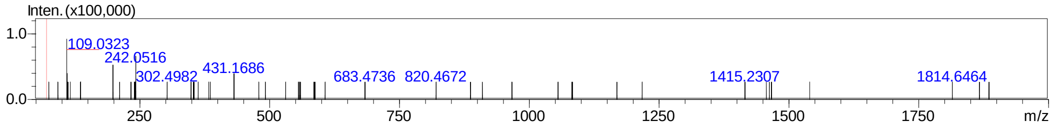

Rank	Score	Formula (M)	Ion	Meas. m/z	Pred. m/z	Diff (mDa)	Diff (ppm)	Iso Score	DBE
1	53.92	C15 H14 O4	[M-H]-	257.0819	257.0819	0.0	0.00	53.92	9.0

M23

Event#: 1 MS(E+) Ret. Time : 45.328 Scan# : 4461

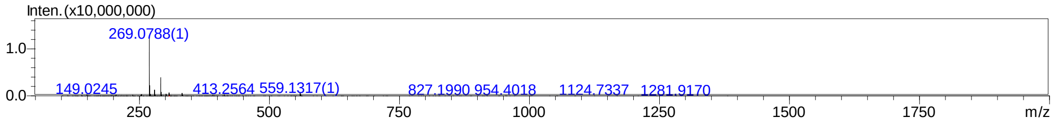

Event#: 4 MS(E-) Ret. Time : 45.328 Scan# : 4463

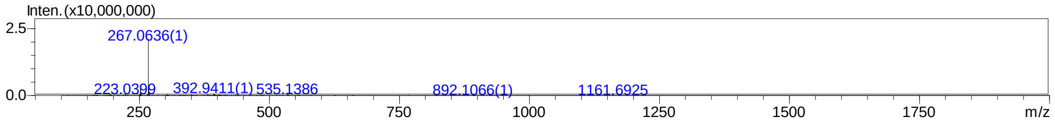

Rank	Score	Formula (M)	Ion	Meas. m/z	Pred. m/z	Diff (mDa)	Diff (ppm)	Iso Score	DBE
3	63.60	C16 H12 O4	[M+H]+	269.0793	269.0808	-1.5	-5.57	75.44	11.0

M24

Event#: 1 MS(E+) Ret. Time : 45.732 Scan# : 4501

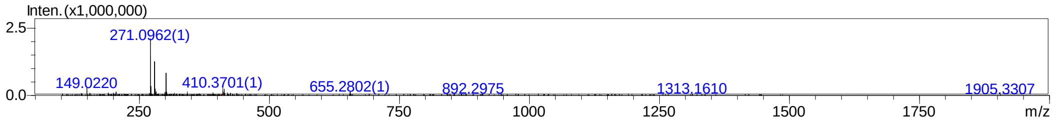

Event#: 4 MS(E-) Ret. Time : 45.732 Scan# : 4504

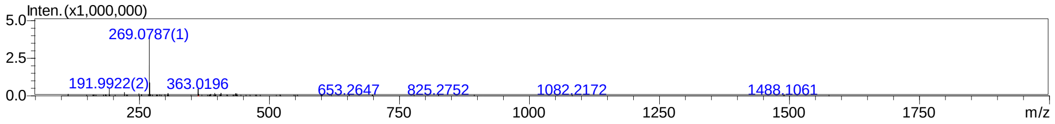

Rank	Score	Formula (M)	Ion	Meas. m/z	Pred. m/z	Diff (mDa)	Diff (ppm)	Iso Score	DBE
1	43.94	C16 H14 O4	[M-H]-	269.0798	269.0819	-2.1	-7.80	70.87	10.0

M25

Event#: 1 MS(E+) Ret. Time : 46.290 Scan# : 4556

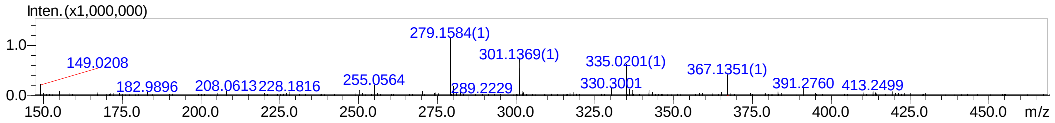

vent#: 2 MS/MS(E+) Ret. Time : 46.170 Scan# : 4545 Precursor : 335.0203 Cutoff : 92

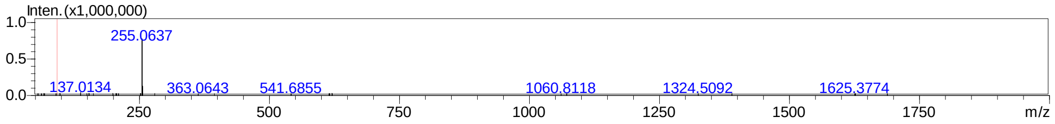

Event#: 4 MS(E-) Ret. Time : 46.170 Scan# : 4547

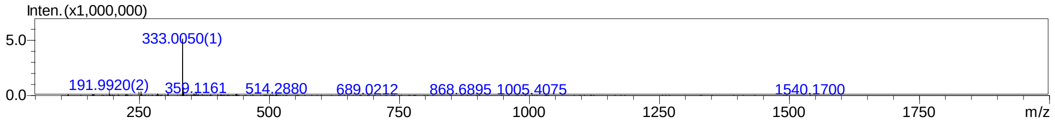

Rank	Score	Formula (M)	Ion	Meas. m/z	Pred. m/z	Diff (mDa)	Diff (ppm)	Iso Score	DBE
1	63.03	C15 H10 O4	[M+H]+	255.0637	255.0652	-1.5	-5.88	77.62	11.0

Rank	Score	Formula (M)	Ion	Meas. m/z	Pred. m/z	Diff (mDa)	Diff (ppm)	Iso Score	DBE
1	47.61	C15 H10 O7 S	[M+H]+	335.0201	335.0220	-1.9	-5.67	57.15	11.0

M26

Event#: 4 MS(E-) Ret. Time : 47.862 Scan# : 4714

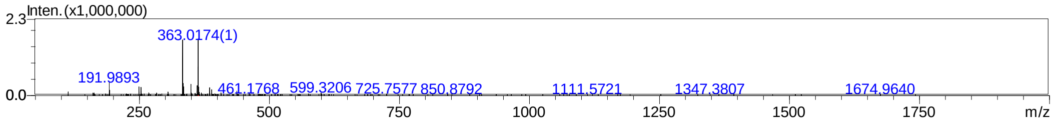

Event#: 5 MS/MS(E-) Ret. Time : 47.862 Scan# : 4715 Precursor : 363.0175 Cutoff : 100

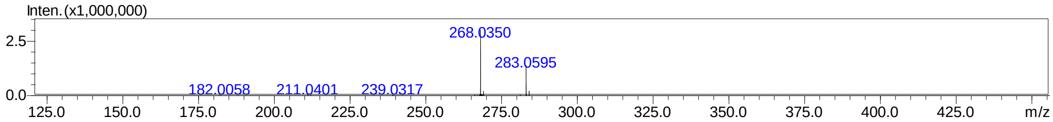

Rank	Score	Formula (M)	Ion	Meas. m/z	Pred. m/z	Diff (mDa)	Diff (ppm)	Iso Score	DBE
1	73.29	C16 H12 O8 S	[M-H]-	363.0174	363.0180	-0.6	-1.65	74.50	11.0

M27

Event#: 4 MS(E-) Ret. Time : 47.922 Scan# : 4719

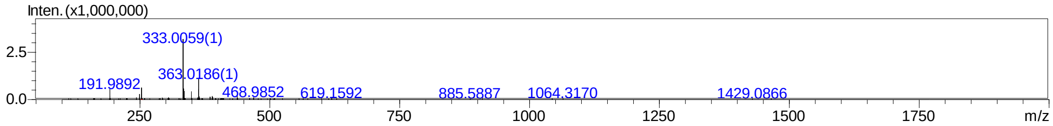

Event#: 5 MS/MS(E-) Ret. Time : 47.922 Scan# : 4720 Precursor : 253.0469 Cutoff : 70

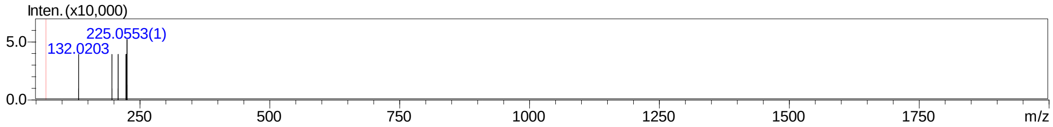

Rank	Score	Formula (M)	Ion	Meas. m/z	Pred. m/z	Diff (mDa)	Diff (ppm)	Iso Score	DBE
1	70.96	C15 H10 O7 S	[M-H]-	333.0059	333.0074	-1.5	-4.50	77.76	11.0

M28

Event#: 4 MS(E-) Ret. Time : 48.205 Scan# : 4748

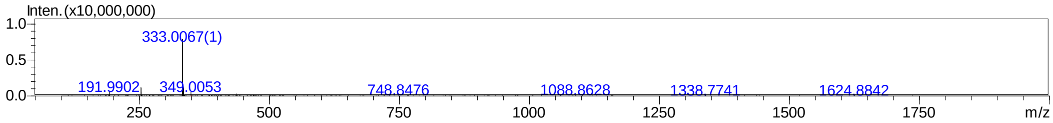

Event#: 5 MS/MS(E-) Ret. Time : 48.205 Scan# : 4749 Precursor : 349.0053 Cutoff : 96

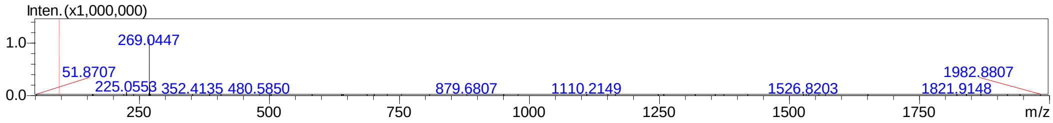

Rank	Score	Formula (M)	Ion	Meas. m/z	Pred. m/z	Diff (mDa)	Diff (ppm)	Iso Score	DBE
4	31.44	C15 H10 O8 S	[M-H]-	349.0053	349.0024	2.9	8.31	55.25	11.0

Rank	Score	Formula (M)	Ion	Meas. m/z	Pred. m/z	Diff (mDa)	Diff (ppm)	Iso Score	DBE
1	58.01	C15 H10 O7 S	[M-H]-	333.0067	333.0074	-0.7	-2.10	59.65	11.0

M30

Event#: 4 MS(E-) Ret. Time : 49.193 Scan# : 4844

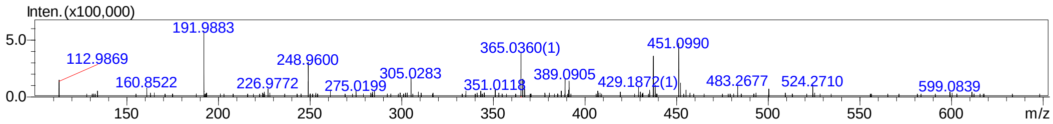

Event#: 5 MS/MS(E-) Ret. Time : 49.193 Scan# : 4845 Precursor : 365.0361 Cutoff : 101

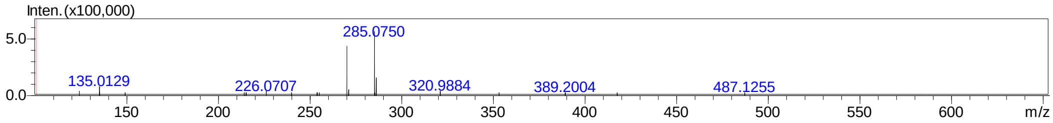

Rank	Score	Formula (M)	Ion	Meas. m/z	Pred. m/z	Diff (mDa)	Diff (ppm)	Iso Score	DBE
1	23.06	C16 H14 O8 S	[M-H]-	365.0360	365.0337	2.3	6.30	29.95	10.0

M29

Event#: 4 MS(E-) Ret. Time : 48.747 Scan# : 4800

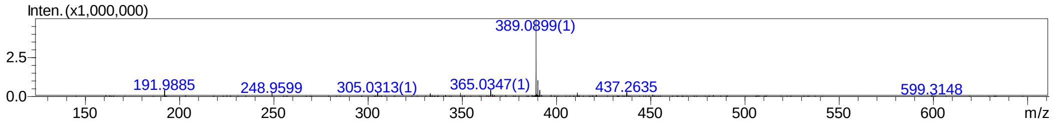

Event#: 5 MS/MS(E-) Ret. Time : 48.747 Scan# : 4801 Precursor : 365.0349 Cutoff : 101

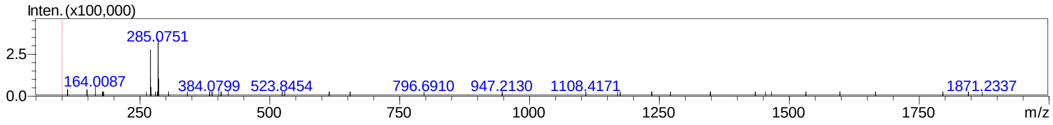

Rank	Score	Formula (M)	Ion	Meas. m/z	Pred. m/z	Diff (mDa)	Diff (ppm)	Iso Score	DBE
1	38.94	C16 H14 O8 S	[M-H]-	365.0347	365.0337	1.0	2.74	40.72	10.0

M31

Event#: 4 MS(E-) Ret. Time : 49.623 Scan# : 4886

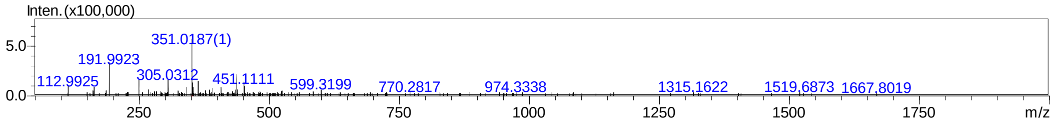

Event#: 5 MS/MS(E-) Ret. Time : 49.623 Scan# : 4887 Precursor : 351.0188 Cutoff : 97

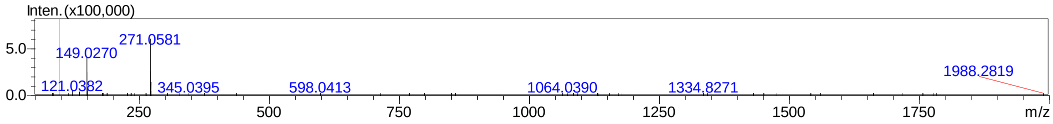

Rank	Score	Formula (M)	Ion	Meas. m/z	Pred. m/z	Diff (mDa)	Diff (ppm)	Iso Score	DBE
1	41.71	C15 H12 O8 S	[M-H]-	351.0187	351.0180	0.7	1.99	42.76	10.0

M32

Event#: 4 MS(E-) Ret. Time : 50.467 Scan# : 4968

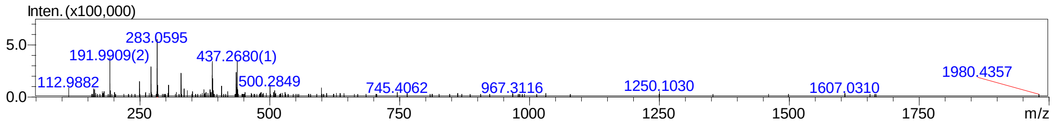

Event#: 5 MS/MS(E-) Ret. Time : 50.467 Scan# : 4969 Precursor : 283.0595 Cutoff : 78

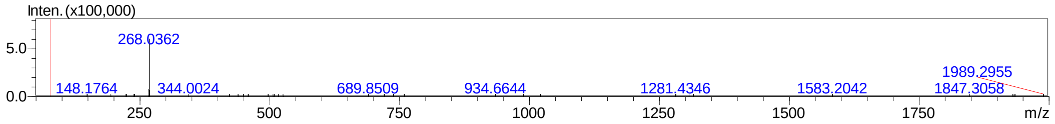

Rank	Score	Formula (M)	Ion	Meas. m/z	Pred. m/z	Diff (mDa)	Diff (ppm)	Iso Score	DBE
1	46.79	C16 H12 O5	[M-H]-	283.0595	283.0612	-1.7	-6.01	58.56	11.0

M33

Event#: 4 MS(E-) Ret. Time : 50.973 Scan# : 5019

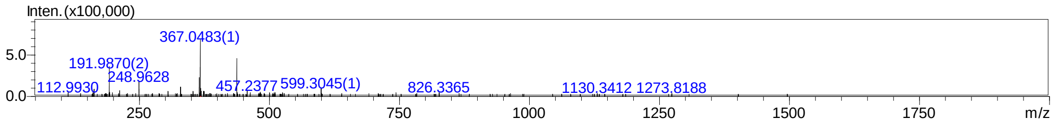

Event#: 5 MS/MS(E-) Ret. Time : 50.973 Scan# : 5020 Precursor : 367.0485 Cutoff : 101

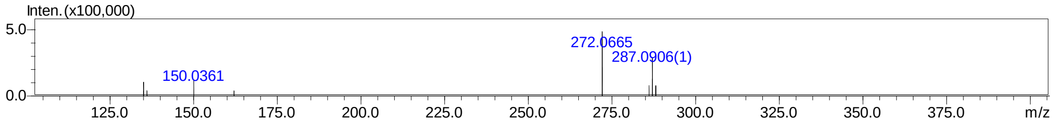

Rank	Score	Formula (M)	Ion	Meas. m/z	Pred. m/z	Diff (mDa)	Diff (ppm)	Iso Score	DBE
1	55.98	C16 H16 O8 S	[M-H]-	367.0483	367.0493	-1.0	-2.72	58.50	9.0

M34

Event#: 4 MS(E-) Ret. Time : 51.515 Scan# : 5072

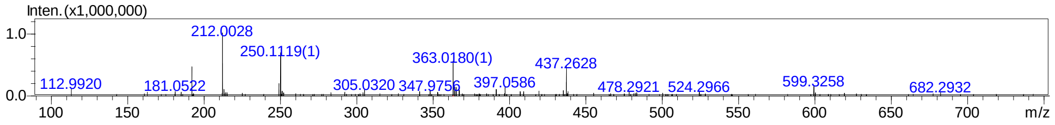

Event#: 5 MS/MS(E-) Ret. Time : 51.515 Scan# : 5073 Precursor : 363.0182 Cutoff : 100

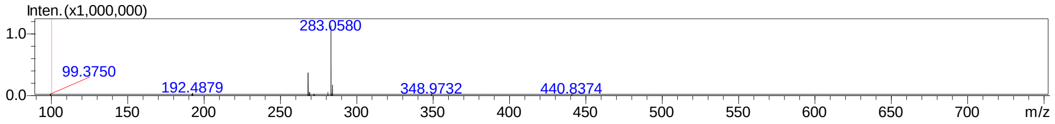

Rank	Score	Formula (M)	Ion	Meas. m/z	Pred. m/z	Diff (mDa)	Diff (ppm)	Iso Score	DBE
1	46.31	C16 H12 O8 S	[M-H]-	363.0180	363.0180	0.0	0.00	46.31	11.0

M35

Event#: 5 MS/MS(E-) Ret. Time : 52.563 Scan# : 5175 Precursor : 335.0251 Cutoff : 92

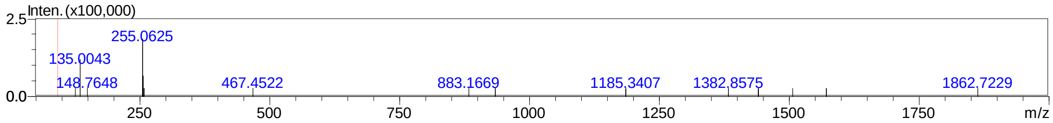

Rank Score Formula (M) Ion Meas. m/z Pred. m/z Diff (mDa) Diff (ppm) Iso Score DBE

1 31.53 C15 H12 O7 S [M-H]- 335.0250 335.0231 1.9 5.67 37.85 10.0

Rank Score Formula (M) Ion Meas. m/z Pred. m/z Diff (mDa) Diff (ppm) Iso Score DBE

1 13.95 C21 H42 O7 S [M-H]- 437.2628 437.2578 5.0 11.43 38.55 1.0

M36

Event#: 4 MS(E-) Ret. Time : 53.637 Scan# : 5280

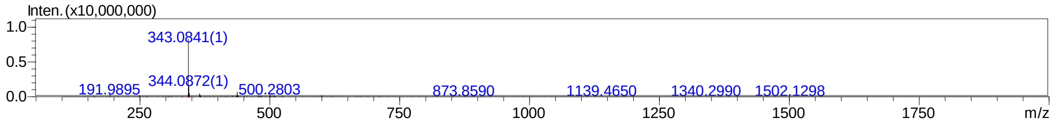

Event#: 5 MS/MS(E-) Ret. Time : 53.637 Scan# : 5281 Precursor : 343.0843 Cutoff : 94

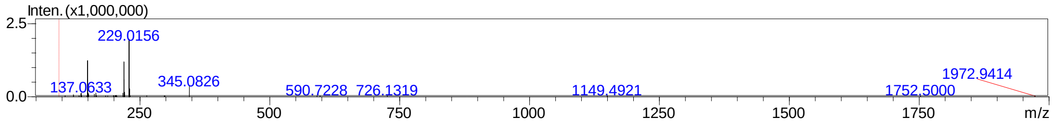

Rank Score Formula (M) Ion Meas. m/z Pred. m/z Diff (mDa) Diff (ppm) Iso Score DBE

1 30.24 C18 H16 O7 [M-H]- 343.0835 343.0823 1.2 3.50 32.26 11.0

2 30.24 C17 H14 O5 [M+HCOO]- 343.0835 343.0823 1.2 3.50 32.26 11.0

M37

Event#: 4 MS(E-) Ret. Time : 55.165 Scan# : 5431

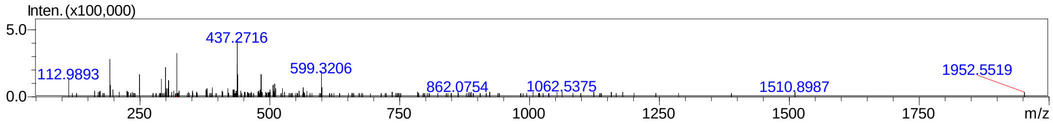

Event#: 5 MS/MS(E-) Ret. Time : 55.165 Scan# : 5432 Precursor : 321.0398 Cutoff : 88

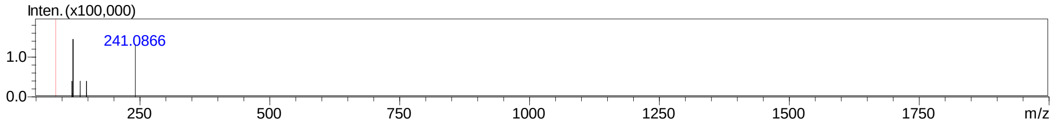

Rank Score Formula (M) Ion Meas. m/z Pred. m/z Diff (mDa) Diff (ppm) Iso Score DBE

1 0.00 C18 H10 O6 [M-H]- 321.0417 321.0405 1.2 3.74 0.00 14.0

4 0.00 C15 H14 O6 S [M-H]- 321.0417 321.0438 -2.1 -6.54 0.00 9.0

M38

Event#: 4 MS(E-) Ret. Time : 59.468 Scan# : 5856

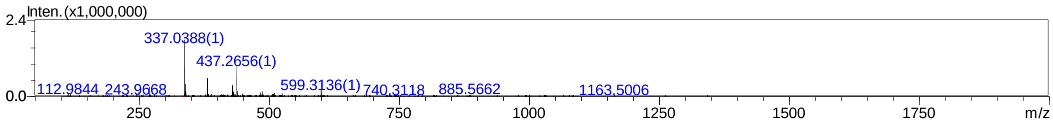

Event#: 5 MS/MS(E-) Ret. Time : 59.468 Scan# : 5857 Precursor : 337.0389 Cutoff : 93

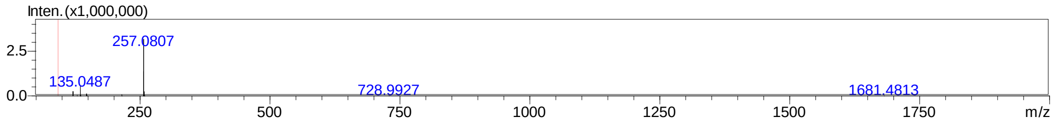

Rank	Score	Formula (M)	Ion	Meas. m/z	Pred. m/z	Diff (mDa)	Diff (ppm)	Iso Score	DBE
1	78.95	C15 H14 O7 S	[M-H]-	337.0388	337.0387	0.1	0.30	78.95	9.0

M43

Event#: 4 MS(E-) Ret. Time : 71.557 Scan# : 7045

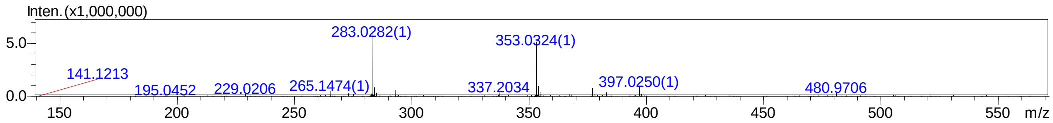

vent#: 5 MS/MS(E-) Ret. Time : 72.485 Scan# : 7137 Precursor : 353.0319 Cutoff : 97

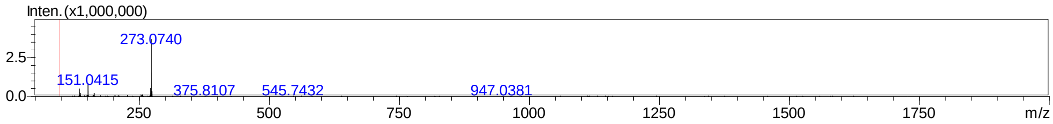

Rank	Score	Formula (M)	Ion	Meas. m/z	Pred. m/z	Diff (mDa)	Diff (ppm)	Iso Score	DBE
1	85.66	C15 H14 O8 S	[M-H]-	353.0324	353.0337	-1.3	-3.68	91.81	9.0

M44

Event#: 5 MS/MS(E-) Ret. Time : 71.557 Scan# : 7046 Precursor : 397.0250 Cutoff : 109

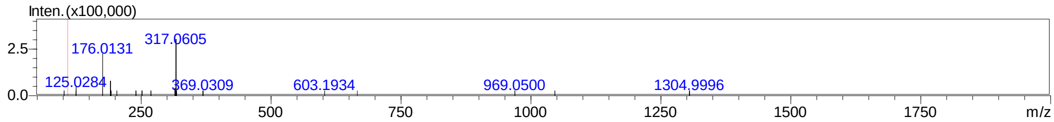

Rank	Score	Formula (M)	Ion	Meas. m/z	Pred. m/z	Diff (mDa)	Diff (ppm)	Iso Score	DBE
1	56.86	C16 H14 O10 S	[M-H]-	397.0250	397.0235	1.5	3.78	61.10	10.0

M39

Event#: 1 MS(E+) Ret. Time : 61.838 Scan# : 6087

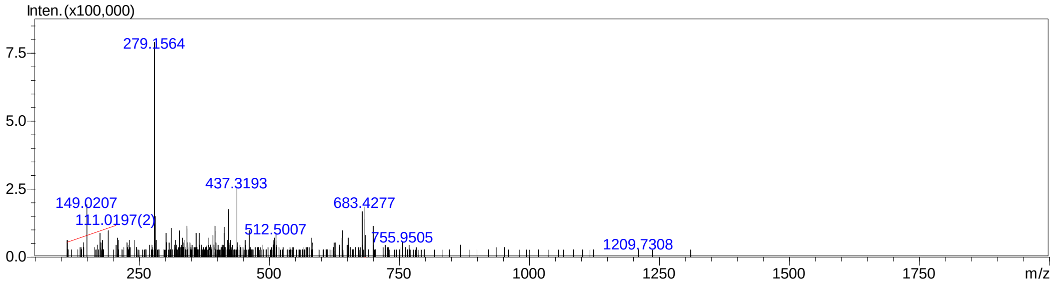

MS/MS(E+) Ret. Time : 61.838 Scan# : 6088 Precursor : 683.4276 Cutoff : 189

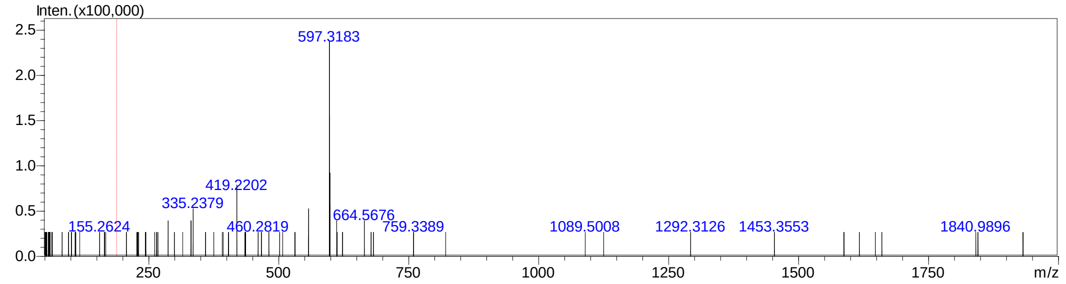

Rank	Score	Formula (M)	Ion	Meas. m/z	Pred. m/z	Diff (mDa)	Diff (ppm)	Iso Score	DBE
1	0.00	C42 H60 O6	[M+Na]+	683.4277	683.4282	-0.5	-0.73	0.00	13.0
**2**	**0.00**	**C35 H64 O11**	**[M+Na]+**	**683.4277**	**683.4341**	**-6.4**	**-9.36**	**0.00**	**4.0**

M40

Event#: 1 MS(E+) Ret. Time : 62.002 Scan# : 6103

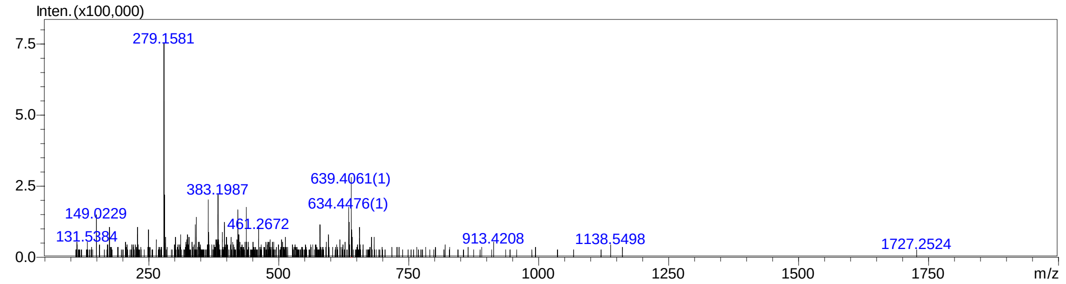

MS/MS(E+) Ret. Time : 62.002 Scan# : 6104 Precursor : 639.4062 Cutoff : 176

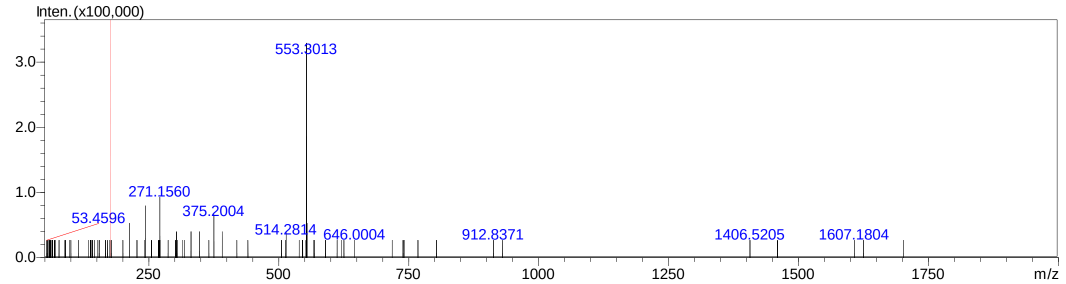

Rank	Score	Formula (M)	Ion	Meas. m/z	Pred. m/z	Diff (mDa)	Diff (ppm)	Iso Score	DBE
8	0.00	C44 H56 O2	[M+Na]+	639.4061	639.4173	-11.2	-17.52	0.00	17.0
7	1.92	C42 H54 O5	[M+H]+	639.4061	639.4044	1.7	2.66	2.00	16.0
6	2.45	C40 H56 O5	[M+Na]+	639.4061	639.4020	4.1	6.41	3.23	13.0
5	3.83	C47 H52	[M+Na]+	639.4061	639.3961	10.0	15.64	15.34	22.0
**4**	**4.21**	**C35 H58 O10**	**[M+H]+**	**639.4061**	**639.4103**	**-4.2**	**-6.57**	**5.67**	**7.0**
3	4.51	C49 H50	[M+H]+	639.4061	639.3985	7.6	11.89	12.90	25.0
2	5.45	C31 H58 O13	[M+H]+	639.4061	639.3950	11.1	17.36	26.73	3.0
1	6.02	C33 H60 O10	[M+Na]+	639.4061	639.4079	-1.8	-2.82	6.31	4.0

M41

Event#: 1 MS(E+) Ret. Time : 62.113 Scan# : 6114

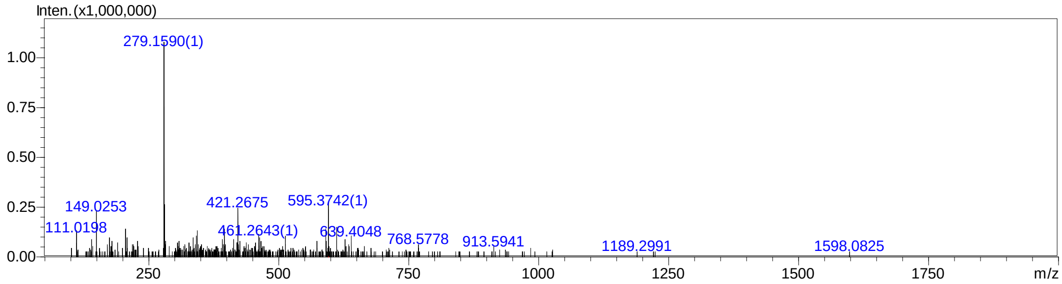

MS/MS(E+) Ret. Time : 62.113 Scan# : 6115 Precursor : 595.3742 Cutoff : 164

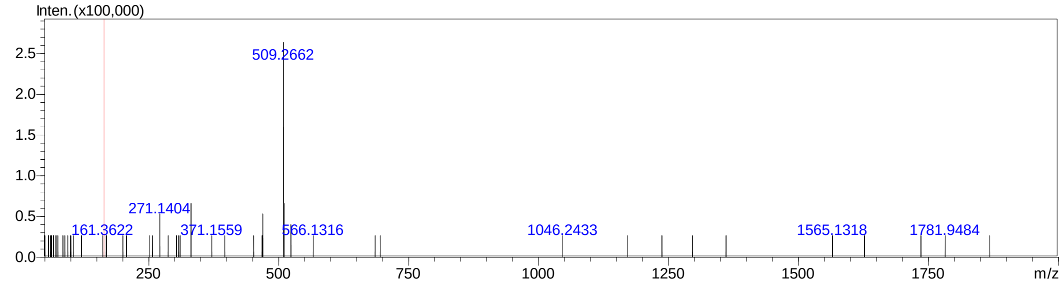

Rank	Score	Formula (M)	Ion	Meas. m/z	Pred. m/z	Diff (mDa)	Diff (ppm)	Iso Score	DBE
1	15.05	C38 H52 O4	[M+Na]+	595.3742	595.3758	-1.6	-2.69	15.72	13.0
2	11.49	C29 H54 O12	[M+H]+	595.3742	595.3688	5.4	9.07	23.30	3.0
3	7.41	C27 H56 O12	[M+Na]+	595.3742	595.3664	7.8	13.10	23.34	0.0
4	6.99	C31 H56 O9	[M+Na]+	595.3742	595.3817	-7.5	-12.60	21.15	4.0
**5**	**3.80**	**C33 H54 O9**	**[M+H]+**	**595.3742**	**595.3841**	**-9.9**	**-16.63**	**17.02**	**7.0**

M42

Event#: 1 MS(E+) Ret. Time : 62.413 Scan# : 6144

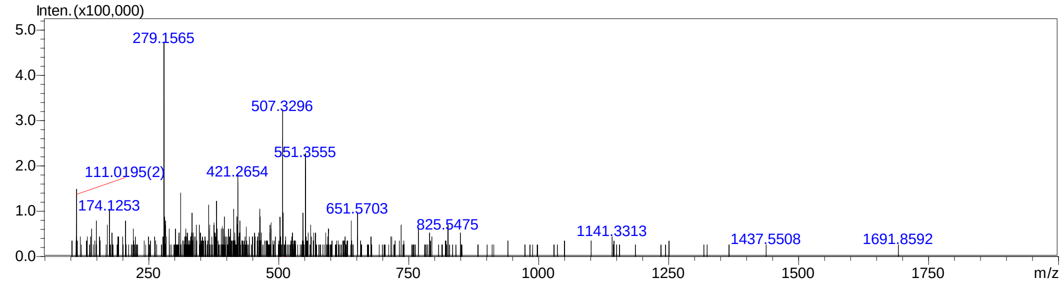

MS/MS(E+) Ret. Time : 62.413 Scan# : 6145 Precursor : 507.3294 Cutoff : 140

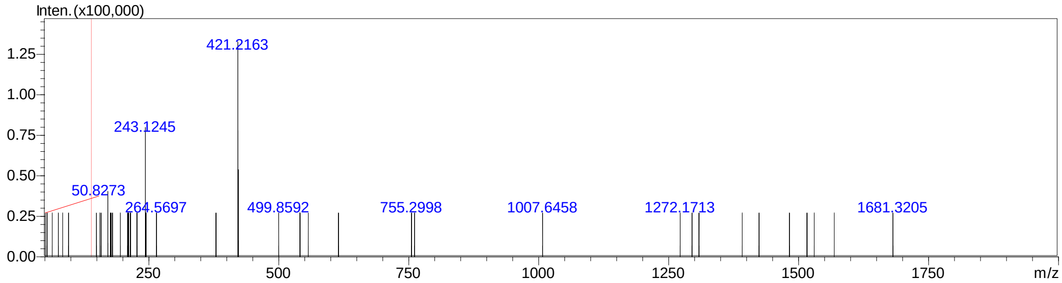

Rank	Score	Formula (M)	Ion	Meas. m/z	Pred. m/z	Diff (mDa)	Diff (ppm)	Iso Score	DBE
1	41.90	C27 H48 O7	[M+Na]+	507.3296	507.3292	0.4	0.79	41.90	4.0
**2**	**29.20**	**C29 H46 O7**	**[M+H]+**	**507.3296**	**507.3316**	**-2.0**	**-3.94**	**31.52**	**7.0**

5. The HRMS data of DBT2 urine samples

m1

Event#: 1 MS(E+) Ret. Time : 4.132 Scan# : 403

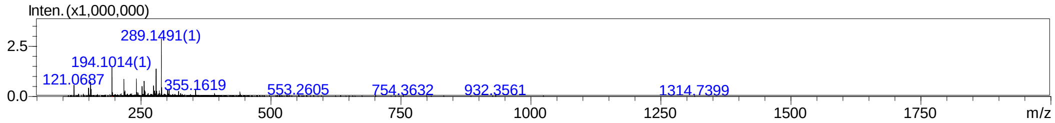

Event#: 4 MS(E-) Ret. Time : 4.132 Scan# : 406

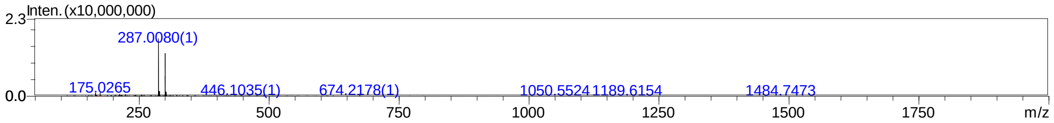

Rank	Score	Formula (M)	Ion	Meas. m/z	Pred. m/z	Diff (mDa)	Diff (ppm)	Iso Score	DBE
1	44.14	C10 H8 O10	[M-H]-	287.0065	287.0045	2.0	6.97	62.79	7.0

m2

Event#: 1 MS(E+) Ret. Time : 28.200 Scan# : 2772

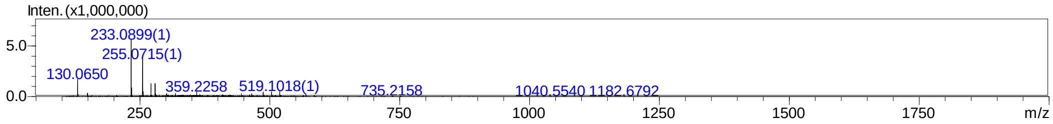

Event#: 4 MS(E-) Ret. Time : 28.200 Scan# : 2774

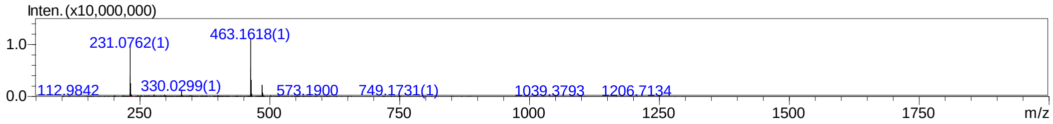

Event#: 5 MS/MS(E-) Ret. Time : 28.147 Scan# : 2770 Precursor : 463.1629 Cutoff : 128

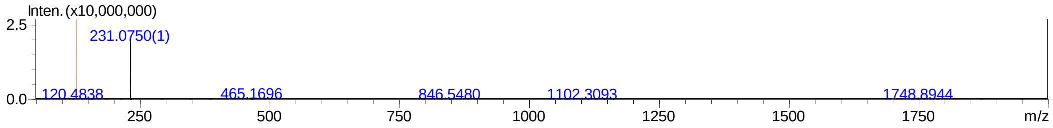

2M-H

Rank	Score	Formula (M)	Ion	Meas. m/z	Pred. m/z	Diff (mDa)	Diff (ppm)	Iso Score	DBE
1	84.13	C12 H12 N2 O3	[M-H]-	231.0768	231.0775	-0.7	-3.03	88.63	8.0

m3

Event#: 4 MS(E-) Ret. Time : 29.298 Scan# : 2883

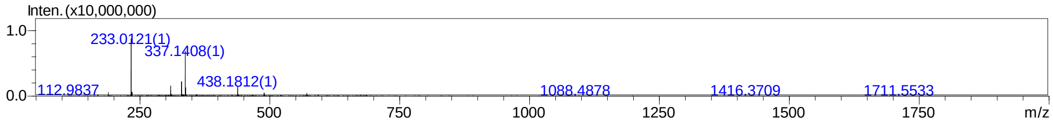

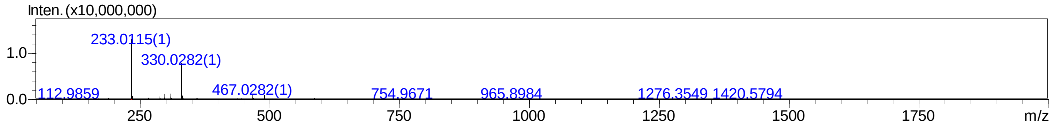

2M-H

Rank	Score	Formula (M)	Ion	Meas. m/z	Pred. m/z	Diff (mDa)	Diff (ppm)	Iso Score	DBE
1	56.14	C12 H2 N4 O2	[M-H]-	233.0115	233.0105	1.0	4.29	61.17	14.0

m4

Event#: 1 MS(E+) Ret. Time : 29.298 Scan# : 2880

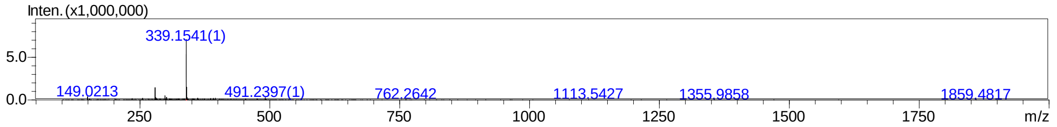

Event#: 4 MS(E-) Ret. Time : 29.298 Scan# : 2883

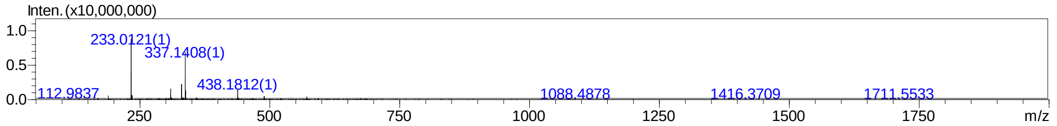

Rank	Score	Formula (M)	Ion	Meas. m/z	Pred. m/z	Diff (mDa)	Diff (ppm)	Iso Score	DBE
1	62.13	C16 H22 N2 O6	[M-H]-	337.1408	337.1405	0.3	0.89	62.13	7.0

m5

Event#: 1 MS(E+) Ret. Time : 31.083 Scan# : 3056

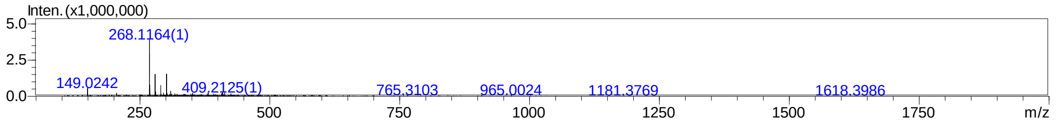

Event#: 4 MS(E-) Ret. Time : 31.083 Scan# : 3059

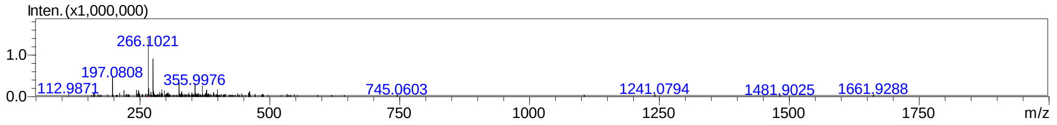

Rank	Score	Formula (M)	Ion	Meas. m/z	Pred. m/z	Diff (mDa)	Diff (ppm)	Iso Score	DBE
1	35.90	C13 H17 N O5	[M-H]-	266.1021	266.1034	-1.3	-4.89	39.76	6.0

m6

Event#: 4 MS(E-) Ret. Time : 31.970 Scan# : 3146

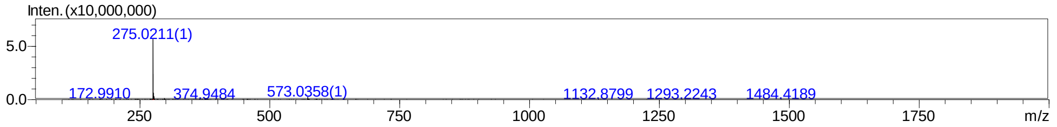

Event#: 5 MS/MS(E-) Ret. Time : 31.970 Scan# : 3147 Precursor : 275.3296 Cutoff : 76

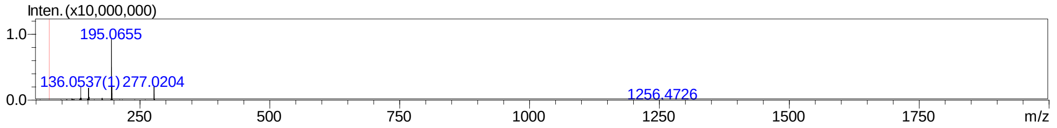

Rank	Score	Formula (M)	Ion	Meas. m/z	Pred. m/z	Diff (mDa)	Diff (ppm)	Iso Score	DBE
1	65.68	C13 H8 O7	[M-H]-	275.0211	275.0197	1.4	5.09	73.71	10.0

m07

Event#: 4 MS(E-) Ret. Time : 32.927 Scan# : 3240

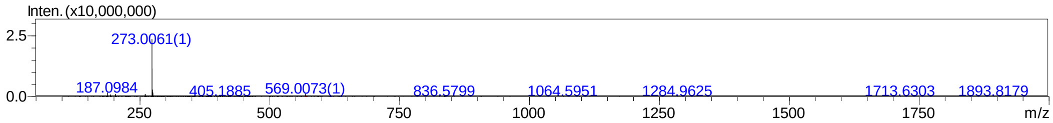

Event#: 5 MS/MS(E-) Ret. Time : 32.927 Scan# : 3241 Precursor : 569.0071 Cutoff : 157

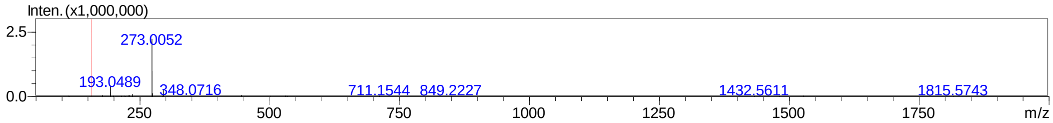

Rank Score Formula (M) Ion Meas. m/z Pred. m/z Diff (mDa) Diff (ppm) Iso Score DBE

1 55.73 C13 H6 O7 [M-H]- 273.0056 273.0041 1.5 5.49 65.49 11.0

m08

Event#: 4 MS(E-) Ret. Time : 34.518 Scan# : 3397

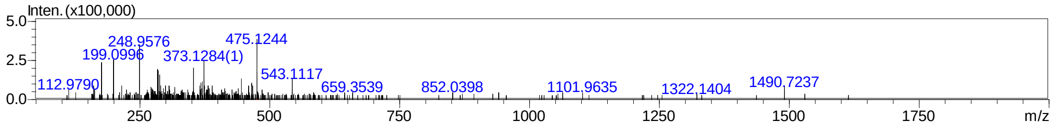

Event#: 5 MS/MS(E-) Ret. Time : 34.518 Scan# : 3398 Precursor : 475.1243 Cutoff : 131

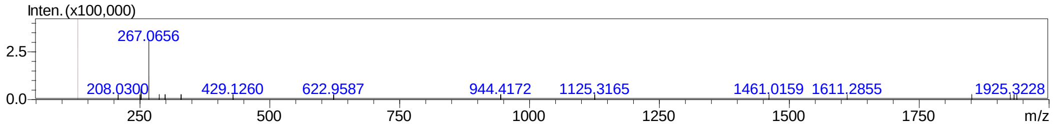

Rank Score Formula (M) Ion Meas. m/z Pred. m/z Diff (mDa) Diff (ppm) Iso Score DBE

1 0.00 C16 H12 O4 [M-H]- 267.0656 267.0663 -0.7 -2.62 0.00 11.0

Rank Score Formula (M) Ion Meas. m/z Pred. m/z Diff (mDa) Diff (ppm) Iso Score DBE

1 29.80 C23 H24 O11 [M-H]- 475.1244 475.1246 -0.2 -0.42 29.80 12.0

2 29.80 C22 H22 O9 [M+HCOO]- 475.1244 475.1246 -0.2 -0.42 29.80 12.0

C6H12O6 C7H16O8

m09

Event#: 1 MS(E+) Ret. Time : 36.583 Scan# : 3598

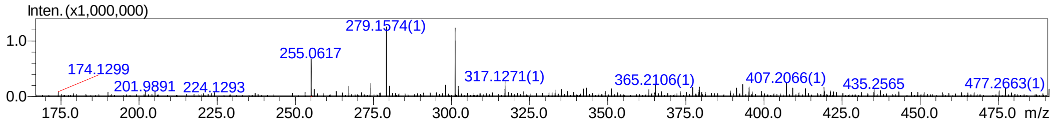

Event#: 4 MS(E-) Ret. Time : 36.583 Scan# : 3601

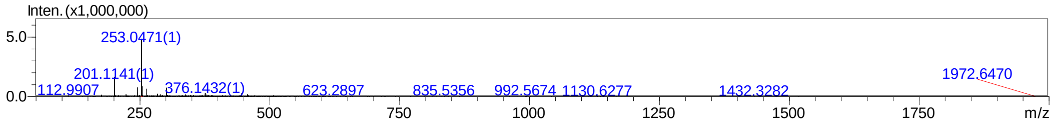

Event#: 5 MS/MS(E-) Ret. Time : 36.583 Scan# : 3602 Precursor : 253.0470 Cutoff : 70

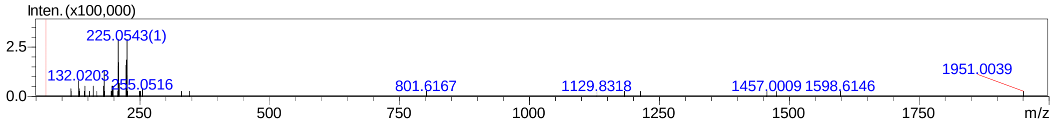

Rank	Score	Formula (M)	Ion	Meas. m/z	Pred. m/z	Diff (mDa)	Diff (ppm)	Iso Score	DBE
2	52.81	C15 H10 O4	[M+H]+	255.0637	255.0652	-1.5	-5.88	65.04	11.0

m10

Event#: 4 MS(E-) Ret. Time : 36.918 Scan# : 3634

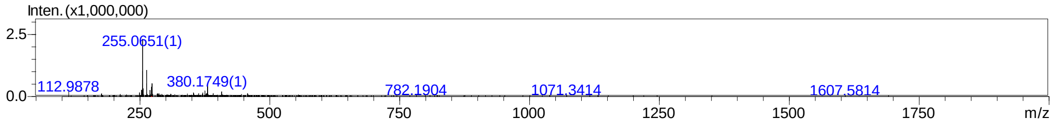

Rank	Score	Formula (M)	Ion	Meas. m/z	Pred. m/z	Diff (mDa)	Diff (ppm)	Iso Score	DBE
1	51.19	C15 H12 O4	[M-H]-	255.0651	255.0663	-1.2	-4.70	56.41	10.0

m11

Event#: 4 MS(E-) Ret. Time : 37.442 Scan# : 3686

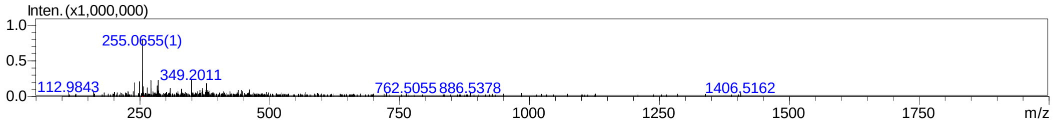

Rank	Score	Formula (M)	Ion	Meas. m/z	Pred. m/z	Diff (mDa)	Diff (ppm)	Iso Score	DBE
1	49.24	C15 H12 O4	[M-H]-	255.0655	255.0663	-0.8	-3.14	52.02	10.0

m12

Event#: 4 MS(E-) Ret. Time : 37.502 Scan# : 3692

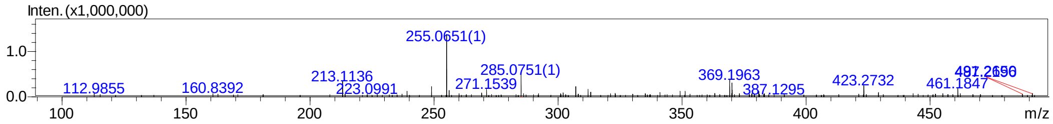

Event#: 5 MS/MS(E-) Ret. Time : 37.502 Scan# : 3693 Precursor : 285.0750 Cutoff : 78

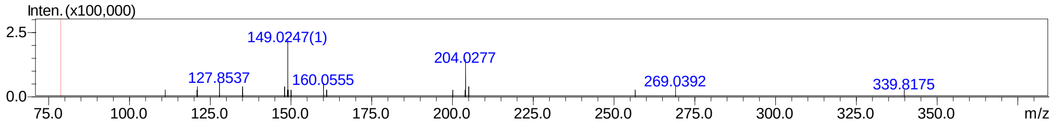

Rank	Score	Formula (M)	Ion	Meas. m/z	Pred. m/z	Diff (mDa)	Diff (ppm)	Iso Score	DBE
1	41.72	C16 H14 O5	[M-H]-	285.0751	285.0768	-1.7	-5.96	51.89	10.0

m13

Event#: 1 MS(E+) Ret. Time : 38.033 Scan# : 3742

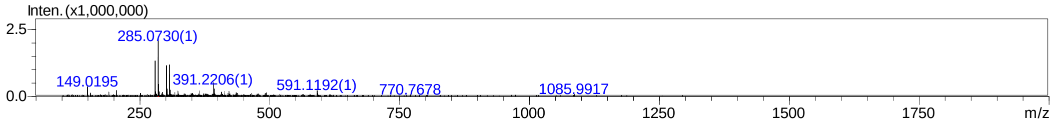

Event#: 4 MS(E-) Ret. Time : 37.973 Scan# : 3739

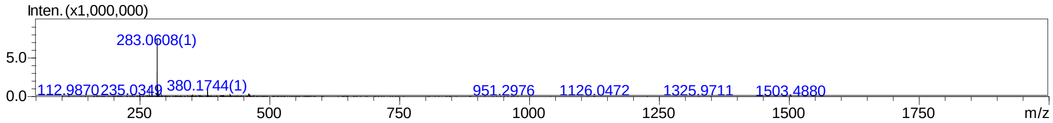

vent#: 5 MS/MS(E-) Ret. Time : 37.973 Scan# : 3740 Precursor : 459.1729 Cutoff : 127

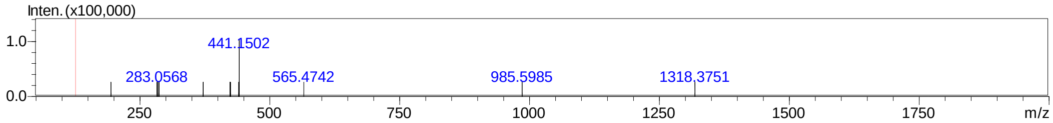

Rank	Score	Formula (M)	Ion	Meas. m/z	Pred. m/z	Diff (mDa)	Diff (ppm)	Iso Score	DBE
1	58.71	C16 H12 O5	[M-H]-	283.0608	283.0612	-0.4	-1.41	59.32	11.0

m14

Event#: 4 MS(E-) Ret. Time : 39.047 Scan# : 3844

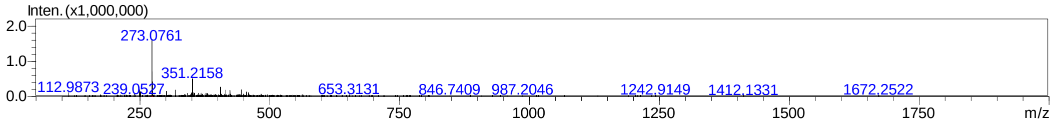

Event#: 5 MS/MS(E-) Ret. Time : 39.047 Scan# : 3845 Precursor : 273.0760 Cutoff : 75

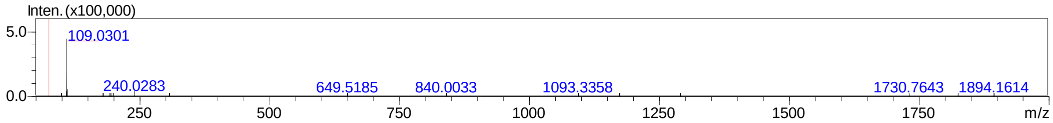

Rank	Score	Formula (M)	Ion	Meas. m/z	Pred. m/z	Diff (mDa)	Diff (ppm)	Iso Score	DBE
1	67.76	C15 H14 O5	[M-H]-	273.0761	273.0768	-0.7	-2.56	70.51	9.0

m15

Event#: 4 MS(E-) Ret. Time : 40.128 Scan# : 3952

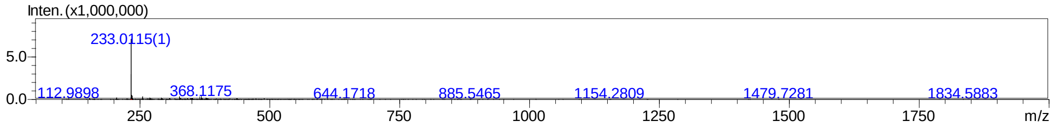

Rank	Score	Formula (M)	Ion	Meas. m/z	Pred. m/z	Diff (mDa)	Diff (ppm)	Iso Score	DBE
1	50.48	C12 H2 N4 O2	[M-H]-	233.0115	233.0105	1.0	4.29	55.01	14.0

m16

Event#: 4 MS(E-) Ret. Time : 41.630 Scan# : 4101

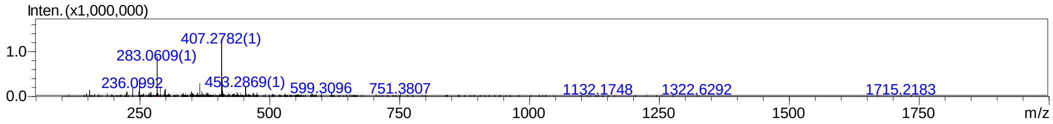

Event#: 5 MS/MS(E-) Ret. Time : 41.630 Scan# : 4102 Precursor : 283.0609 Cutoff : 78

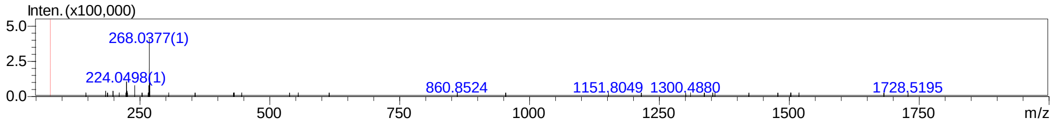

Rank	Score	Formula (M)	Ion	Meas. m/z	Pred. m/z	Diff (mDa)	Diff (ppm)	Iso Score	DBE
1	45.89	C16 H12 O5	[M-H]-	283.0609	283.0612	-0.3	-1.06	45.96	11.0

m17

Event#: 4 MS(E-) Ret. Time : 42.180 Scan# : 4155

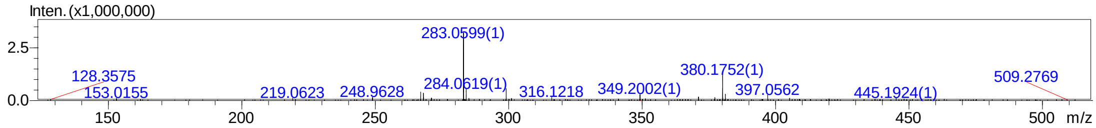

Event#: 5 MS/MS(E-) Ret. Time : 42.180 Scan# : 4156 Precursor : 349.2003 Cutoff : 96

Rank	Score	Formula (M)	Ion	Meas. m/z	Pred. m/z	Diff (mDa)	Diff (ppm)	Iso Score	DBE
1	54.91	C16 H12 O5	[M-H]-	283.0603	283.0612	-0.9	-3.18	58.08	11.0

m18

Event#: 4 MS(E-) Ret. Time : 44.327 Scan# : 4367

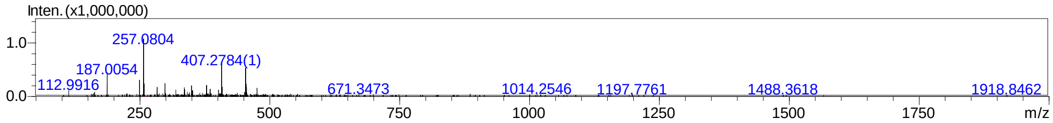

Rank	Score	Formula (M)	Ion	Meas. m/z	Pred. m/z	Diff (mDa)	Diff (ppm)	Iso Score	DBE
1	31.69	C15 H14 O4	[M-H]-	257.0804	257.0819	-1.5	-5.83	38.79	9.0

m19

vent#: 1 MS(E+) Ret. Time : 45.322 Scan# : 4463

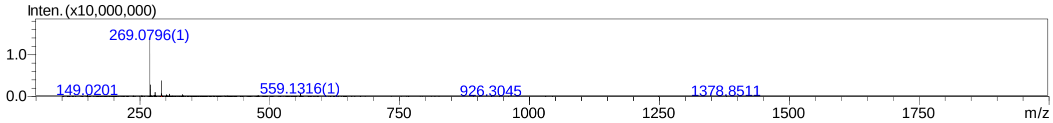

vent#: 4 MS(E-) Ret. Time : 45.322 Scan# : 4465

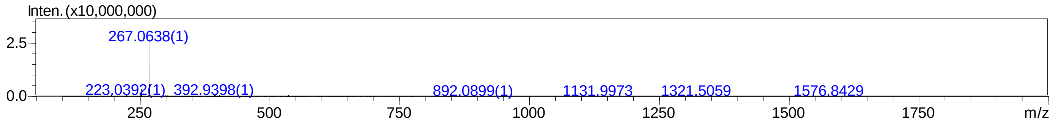

Rank	Score	Formula (M)	Ion	Meas. m/z	Pred. m/z	Diff (mDa)	Diff (ppm)	Iso Score	DBE
1	73.96	C16 H12 O4	[M+H]+	269.0796	269.0808	-1.2	-4.46	80.96	11.0

m20

Event#: 1 MS(E+) Ret. Time : 45.733 Scan# : 4504

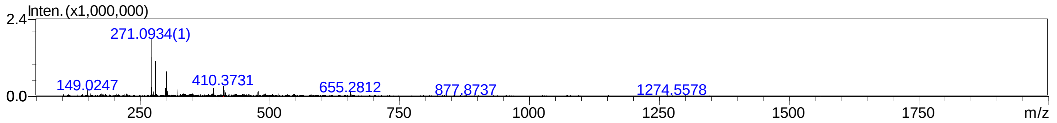

Event#: 4 MS(E-) Ret. Time : 45.673 Scan# : 4501

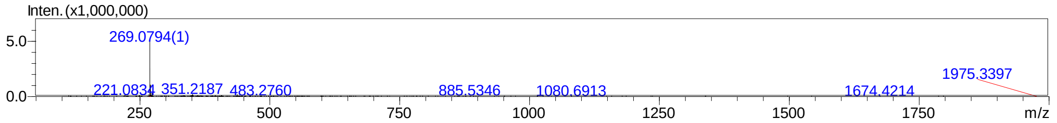

Rank	Score	Formula (M)	Ion	Meas. m/z	Pred. m/z	Diff (mDa)	Diff (ppm)	Iso Score	DBE
1	67.33	C16 H14 O4	[M-H]-	269.0804	269.0819	-1.5	-5.57	79.87	10.0

m21

Event#: 4 MS(E-) Ret. Time : 47.175 Scan# : 4650

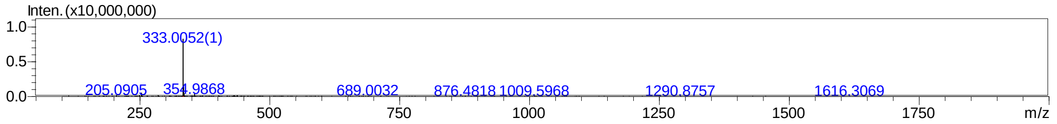

Event#: 5 MS/MS(E-) Ret. Time : 47.175 Scan# : 4651 Precursor : 333.0054 Cutoff : 92

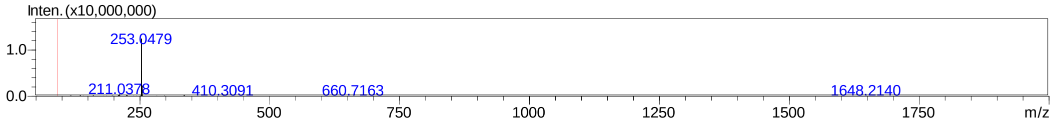

Rank	Score	Formula (M)	Ion	Meas. m/z	Pred. m/z	Diff (mDa)	Diff (ppm)	Iso Score	DBE
1	64.86	C15 H10 O7 S	[M-H]-	333.0052	333.0074	-2.2	-6.61	87.76	11.0

m22

Event#: 4 MS(E-) Ret. Time : 47.775 Scan# : 4709

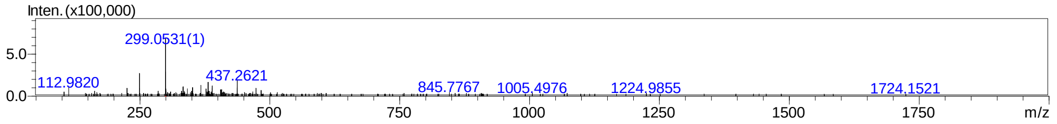

Rank	Score	Formula (M)	Ion	Meas. m/z	Pred. m/z	Diff (mDa)	Diff (ppm)	Iso Score	DBE
1	41.92	C16 H12 O6	[M-H]-	299.0556	299.0561	-0.5	-1.67	42.64	11.0

m23

Event#: 4 MS(E-) Ret. Time : 49.072 Scan# : 4838

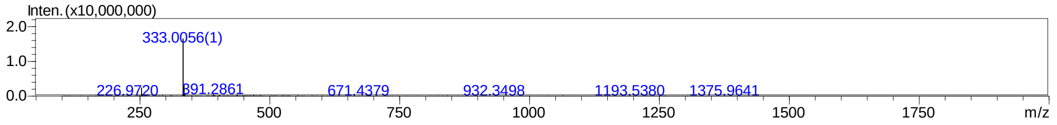

Event#: 5 MS/MS(E-) Ret. Time : 49.072 Scan# : 4839 Precursor : 333.0055 Cutoff : 92

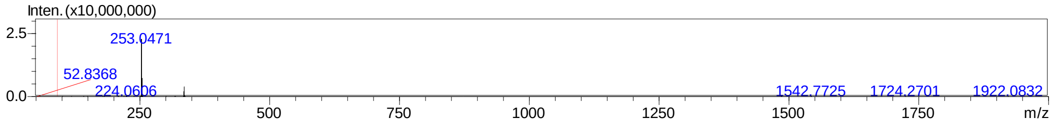

Rank	Score	Formula (M)	Ion	Meas. m/z	Pred. m/z	Diff (mDa)	Diff (ppm)	Iso Score	DBE
1	71.66	C15 H10 O7 S	[M-H]-	333.0056	333.0074	-1.8	-5.41	83.42	11.0

m25

Event#: 4 MS(E-) Ret. Time : 49.810 Scan# : 4911

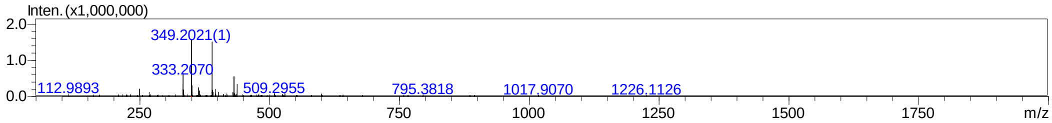

vent#: 5 MS/MS(E-) Ret. Time : 49.810 Scan# : 4912 Precursor : 349.0033 Cutoff : 96

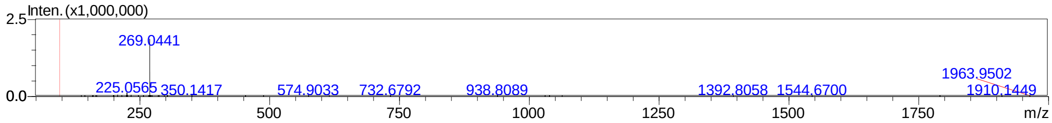

Rank	Score	Formula (M)	Ion	Meas. m/z	Pred. m/z	Diff (mDa)	Diff (ppm)	Iso Score	DBE
1	37.85	C15 H10 O8 S	[M-H]-	349.0033	349.0024	0.9	2.58	39.41	11.0

Rank	Score	Formula (M)	Ion	Meas. m/z	Pred. m/z	Diff (mDa)	Diff (ppm)	Iso Score	DBE
1	27.31	C15 H10 O5	[M-H]-	269.0438	269.0455	-1.7	-6.32	35.56	11.0

m24

Event#: 4 MS(E-) Ret. Time : 49.543 Scan# : 4884

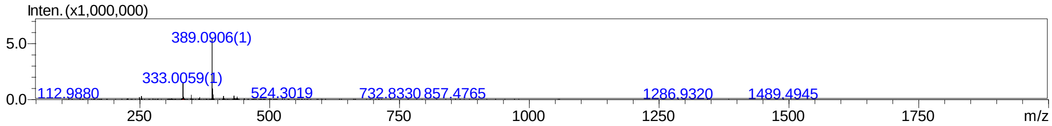

Event#: 5 MS/MS(E-) Ret. Time : 49.698 Scan# : 4901 Precursor : 333.2078 Cutoff : 92

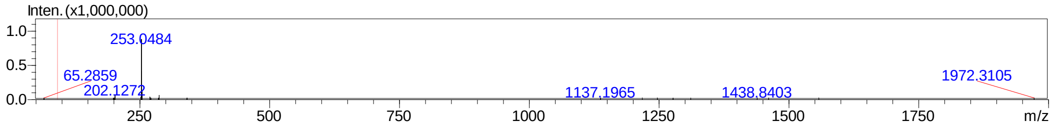

Rank	Score	Formula (M)	Ion	Meas. m/z	Pred. m/z	Diff (mDa)	Diff (ppm)	Iso Score	DBE
1	36.99	C15 H10 O4	[M-H]-	253.0484	253.0506	-2.2	-8.69	69.66	11.0

Rank	Score	Formula (M)	Ion	Meas. m/z	Pred. m/z	Diff (mDa)	Diff (ppm)	Iso Score	DBE
1	78.22	C15 H10 O7 S	[M-H]-	333.0059	333.0074	-1.5	-4.50	85.72	11.0

m26

Event#: 4 MS(E-) Ret. Time : 49.930 Scan# : 4923

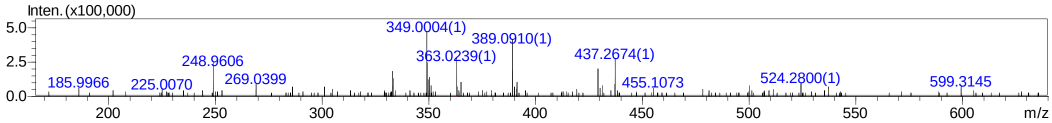

Event#: 5 MS/MS(E-) Ret. Time : 49.930 Scan# : 4924 Precursor : 363.0240 Cutoff : 100

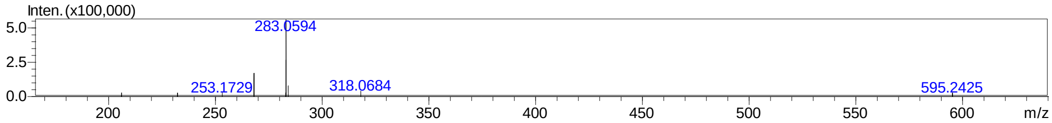

Rank	Score	Formula (M)	Ion	Meas. m/z	Pred. m/z	Diff (mDa)	Diff (ppm)	Iso Score	DBE
12	6.04	C16 H12 O8 S	[M-H]-	363.0239	363.0180	5.9	16.25	25.87	11.0

Rank	Score	Formula (M)	Ion	Meas. m/z	Pred. m/z	Diff (mDa)	Diff (ppm)	Iso Score	DBE
4	13.46	C16 H12 O8 S	[M-H]-	363.0184	363.0180	0.4	1.10	13.50	11.0

m27

Event#: 4 MS(E-) Ret. Time : 50.325 Scan# : 4962

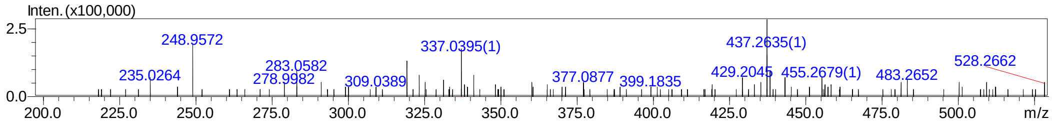

Event#: 5 MS/MS(E-) Ret. Time : 50.325 Scan# : 4963 Precursor : 337.0397 Cutoff : 93

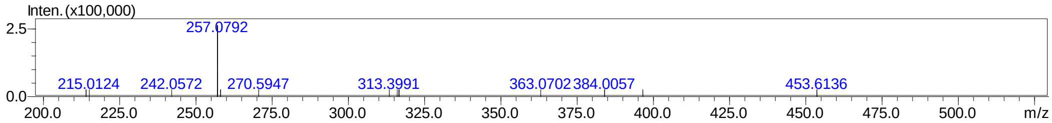

Rank	Score	Formula (M)	Ion	Meas. m/z	Pred. m/z	Diff (mDa)	Diff (ppm)	Iso Score	DBE
1	48.84	C15 H14 O7 S	[M-H]-	337.0395	337.0387	0.8	2.37	50.58	9.0

m28

Event#: 4 MS(E-) Ret. Time : 55.693 Scan# : 5491

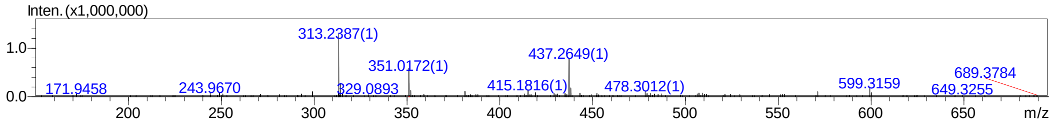

Event#: 5 MS/MS(E-) Ret. Time : 55.693 Scan# : 5492 Precursor : 351.0172 Cutoff : 97

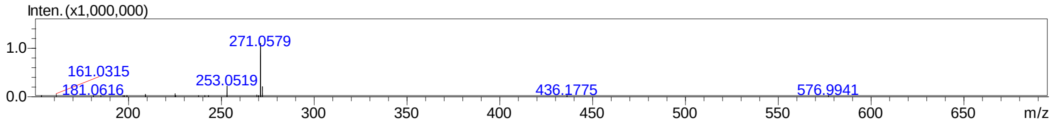

Rank	Score	Formula (M)	Ion	Meas. m/z	Pred. m/z	Diff (mDa)	Diff (ppm)	Iso Score	DBE
1	53.68	C15 H12 O8 S	[M-H]-	351.0172	351.0180	-0.8	-2.28	55.46	10.0

Event#: 4 MS(E-) Ret. Time : 57.015 Scan# : 5622

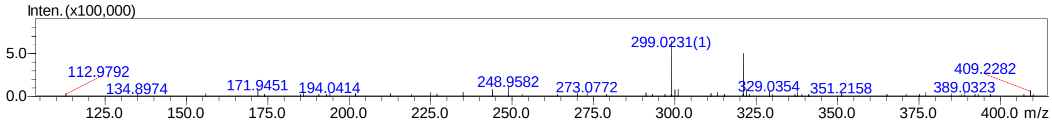

m29

Event#: 5 MS/MS(E-) Ret. Time : 57.015 Scan# : 5623 Precursor : 321.0414 Cutoff : 88

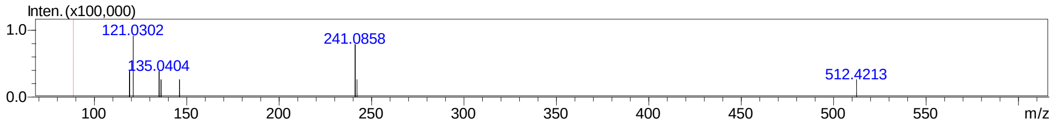

Rank Score Formula (M) Ion Meas. m/z Pred. m/z Diff (mDa) Diff (ppm) Iso Score DBE

1 0.00 C15 H14 O6 S [M-H]- 321.0414 321.0438 -2.4 -7.48 0.00 9.0

M033

Event#: 5 MS/MS(E-) Ret. Time : 72.797 Scan# : 7175 Precursor : 363.0198 Cutoff : 100

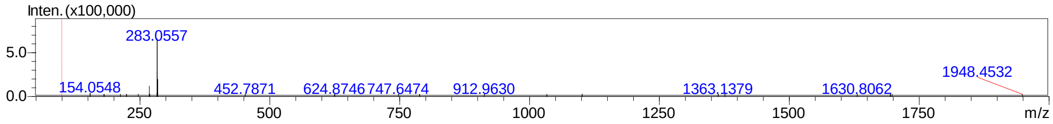

Rank Score Formula (M) Ion Meas. m/z Pred. m/z Diff (mDa) Diff (ppm) Iso Score DBE

1 29.40 C16 H12 O8 S [M-H]- 363.0197 363.0180 1.7 4.68 32.38 11.0

m34

Event#: 4 MS(E-) Ret. Time : 73.543 Scan# : 7249

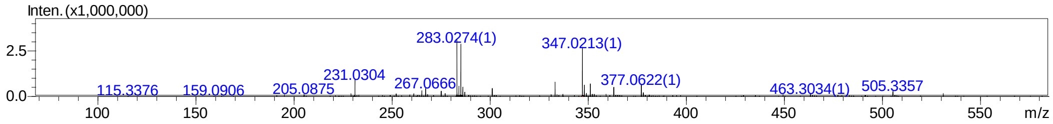

Event#: 5 MS/MS(E-) Ret. Time : 73.543 Scan# : 7250 Precursor : 347.0214 Cutoff : 96

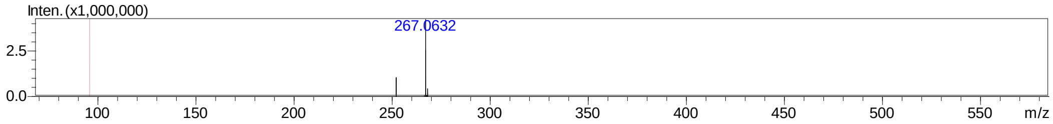

Rank Score Formula (M) Ion Meas. m/z Pred. m/z Diff (mDa) Diff (ppm) Iso Score DBE

1 60.34 C16 H12 O7 S [M-H]- 347.0213 347.0231 -1.8 -5.19 68.49 11.0

m31

Event#: 1 MS(E+) Ret. Time : 62.218 Scan# : 6133

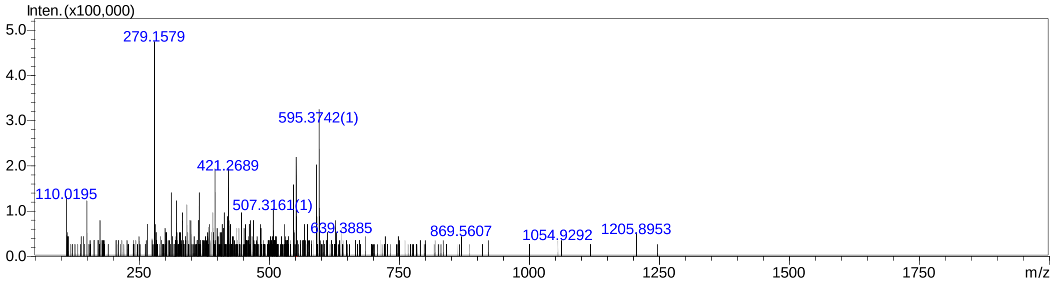

Rank Score Formula (M) Ion Meas. m/z Pred. m/z Diff (mDa) Diff (ppm) Iso Score DBE

1 11.03 C29 H54 O12 [M+H]+ 595.3742 595.3688 5.4 9.07 22.37 3.0

2 9.28 C27 H56 O12 [M+Na]+ 595.3742 595.3664 7.8 13.10 29.24 0.0

3 6.34 C38 H52 O4 [M+Na]+ 595.3742 595.3758 -1.6 -2.69 6.62 13.0

4	3.26	C31 H56 O9	[M+Na]+	595.3742	595.3817	-7.5	-12.60	9.87	4.0
5	2.98	C40 H50 O4	[M+H]+	595.3742	595.3782	-4.0	-6.72	4.10	16.0
6	1.99	C36 H50 O7	[M+H]+	595.3742	595.3629	11.3	18.98	12.40	12.0
**7**	**1.58**	**C33 H54 O9**	**[M+H]+**	**595.3742**	**595.3841**	**-9.9**	**-16.63**	**7.09**	**7.0**

m30

Event#: 1 MS(E+) Ret. Time : 59.728 -> 59.995 Scan# : 5887 -> 5913

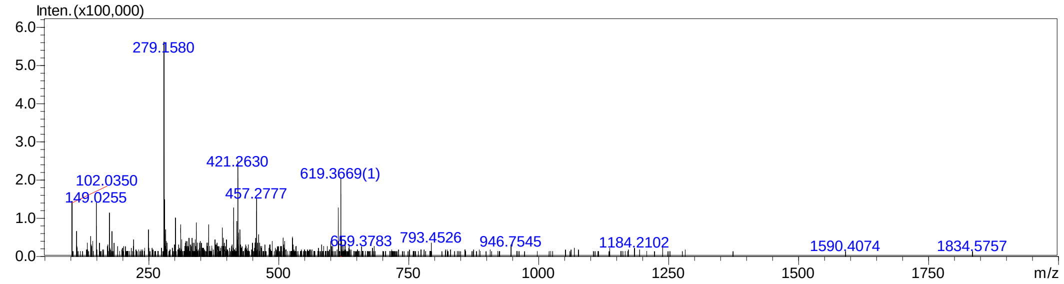

MS/MS(E+) Ret. Time : 59.728 -> 59.995 Scan# : 5888 -> 5914 Precursor : 619.3650 Cutoff : 171

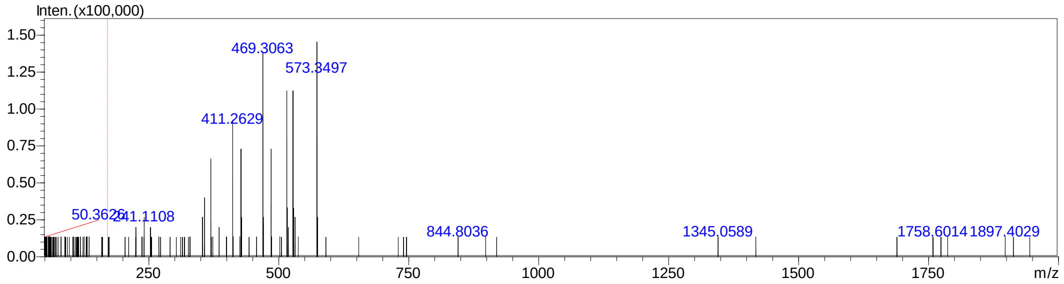

Rank	Score	Formula (M)	Ion	Meas. m/z	Pred. m/z	Diff (mDa)	Diff (ppm)	Iso Score	DBE
1	24.07	C29 H56 O12	[M+Na]+	619.3669	619.3664	0.5	0.81	24.07	2.0
2	8.39	C38 H50 O7	[M+H]+	619.3669	619.3629	4.0	6.46	11.12	14.0
**3**	**5.59**	**C36 H52 O7**	**[M+Na]+**	**619.3669**	**619.3605**	**6.4**	**10.33**	**14.29**	**11.0**

m32

Event#: 4 MS(E-) Ret. Time : 63.292 -> 68.353 Scan# : 6241 -> 6739

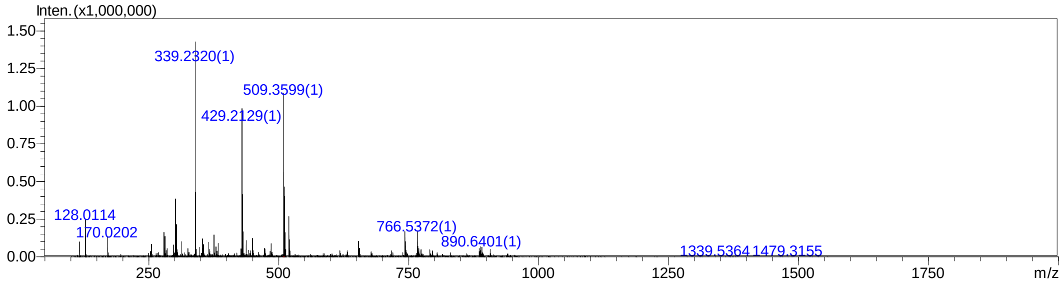

MS/MS(E-) Ret. Time : 63.292 -> 68.353 Scan# : 6242 -> 6740 Precursor : 509.3632 Cutoff : 140

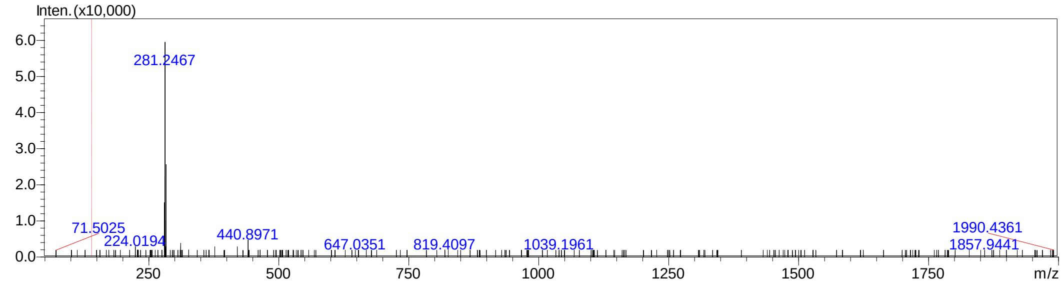

Rank	Score	Formula (M)	Ion	Meas. m/z	Pred. m/z	Diff (mDa)	Diff (ppm)	Iso Score	DBE
**1**	**22.16**	**C33 H50 O4**	**[M-H]-**	**509.3599**	**509.3636**	**-3.7**	**-7.26**	**32.89**	**9.0**

6. The supplemental data of tables S1–S6

Table S1. The ion fragment of 19 kinds of reference compounds by HPLC-IT-TOF-MS^n.

NO.	T_R (min)	$[M+H]^+$	$[M-H]^-$	Predicted Formula	Fragment ions	Error (ppm)	The name of the reference compounds
1 (T1)	28.595	447.1290		$C_{22}H_{22}O_{10}$	447,285,270,225	0.89	Calycosin-7-O-*β*-D-glucopyranoside
2(AW)	29.162		193.0501	$C_{10}H_{10}O_4$	193,178,134	−2.59	Ferulic acid
3(T2)	34.545		431.1326	$C_{22}H_{22}O_9$	431,269,253,237,118	−2.55	Ononin
4 (T3)	36.038	485.1400	$[M+Na]^+$	$C_{23}H_{26}O_{10}$	485,463,323,301	−3.71	Astrapterocarpan-7-O-*β*-D-glucopyranoside
5 (T4)	36.785	487.1574	$[M+Na]^+$	$C_{23}H_{28}O_{10}$	487,303,167	−0.21	Astraisoflavan-7-O-*β*-D-glucopyranoside
6 (T5)	38.055	285.0754		$C_{16}H_{12}O_5$	285,270,225，137	−1.40	Calycosin
7 (T6)	40.123	473.1432		$C_{24}H_{24}O_{10}$	473,269	−2.11	6''-O-acetyl-ononin
8(T7)	41.942	529.1691	$[M+Na]^+$	$C_{25}H_{30}O_{11}$	529,507,303	2.08	6''-O-acetyl-astraisoflavan-7-O-*β*-D-glucopyranoside
9(T8)	45.410	269.0795		$C_{16}H_{12}O_4$	269,270,237	−4.83	Formononetin
10(T9)	46.062	301.1076		$C_{17}H_{16}O_5$		1.66	(6aR,11aR)-3-hydroxy-9,10-dimethoxypterocarpan; Astrapterocarpan
11(T10)	46.655	303.1213		$C_{17}H_{18}O_5$	303,167,123	−4.62	(3R)-7,2'-dihydroxy-3',4'-dimethoxyisoflavan; Astraisoflavan
12(GⅤ)	41.110	$[M+HCOO]^-$	991.5156	$C_{47}H_{78}O_{19}$	991,783,397	3.73	Astragaloside Ⅴ,Ⅵ,Ⅶ
13(GⅣ)	42.775	$[M+HCOO]^-$	829.4615	$C_{41}H_{68}O_{14}$	829,783,621,489,383	2.89	Astragaloside Ⅳ
14(GⅢ)	43.410	$[M+HCOO]^-$	829.4585	$C_{41}H_{68}O_{14}$	829,783,651,489	−0.72	Astragaloside Ⅲ
15(GⅡ)	44.628	$[M+HCOO]^-$	871.4693	$C_{43}H_{70}O_{15}$	871,765,717	−0.46	Astragaloside Ⅱ
16(GⅠ)	50.525	$[M+HCOO]^-$	913.4802	$C_{45}H_{72}O_{16}$	913,867,807	0.00	Astragaloside Ⅰ
17(HHQC)	51.855	513.3550	$[M+Na]^+$	$C_{30}H_{50}O_5$	513,515,405,229	0.00	Cycloastragenol(HHQC)
18(ZL)	55.477	191.1056		$C_{12}H_{14}O_2$	191,173,117	−5.76	Z-ligustilide
19(EL)	58.102	191.1066		$C_{12}H_{14}O_2$	191,173	−0.52	E-ligustilide

Table S2. The identified proposed compounds from DBT1 boiled sample by HPLC-IT-TOF-MS^n.

NO.	T_R (min)	$[M+H]^+$	$[M-H]^-$	Predicted Formula	Fragment ions	Error ppm	Identification	T	IT	S
C1	2.280	175.1168		$C_6H_{14}N_4O_2$		−12.56	Arginine	●		
C2	2.692		195.0502	$C_6H_{12}O_7$		−4.10	Gluconic acid	●		
C3	3.095	365.1007		$C_{12}H_{22}O_{11}$		−12.87	D(+)sucrose	●		
C4	3.682		191.0187	$C_6H_8O_7$		−5.24	Citric acid	●		
C5	4.498	330.0583		$C_{11}H_{11}N_3O_9$		4.54	--	●		
C6	6.480	346.0529		$C_{11}H_{11}N_3O_{10}$		3.47	--	●		
C7	9.063		433.1364	$C_{18}H_{26}O_{12}$	301,191	4.91	--			◎
C8	9.398		433.1364	$C_{18}H_{26}O_{12}$	433,351,301,223	2.77	--			◎
C9	10.773	188.0688		$C_9H_{11}NO_2$	146	3.19	L-phenylalanine	●		
C10	11.125		431.1192	$C_{18}H_{24}O_{12}$	431,299	−0.70	--			◎
C11	12.653	384.1127		$C_{13}H_{21}NO_{12}$		−2.60	--	●		
C12	13.847	485.1224	$[M+Na]^+$	$C_{26}H_{22}O_8$	485,317	3.50	--			◎
C13	17.807		205.0701	$C_8H_{14}O_6$		−8.29	--			◎
C14	21.147		315.2004	$C_{20}H_{28}O_3$		12.06	--			◎
C15	22.132		417.1016	$C_{17}H_{22}O_{12}$		−5.27	--		★	
C16	22.950	389.2359		$C_{23}H_{32}O_5$		9.25	--	●		
C17	23.012		401.1443	$C_{18}H_{26}O_{10}$	401,269	−2.49	--	●		
C18	23.590		503.1175	$C_{24}H_{24}O_{12}$	503,443,299	−3.98	6``-O-acetyl-pratensein-7-O-β-D-glucoside			◎
C19	24.347		239.0568	$C_{11}H_{12}O_6$		2.93	--			◎
C20	25.662	331.2296		$C_{21}H_{30}O_3$	331,299	8.45	--			◎
C21	26.743	470.1534		$C_{18}H_{23}N_5O_{10}$		3.40	--			◎
C22	26.967	289.1747		$C_{13}H_{24}N_2O_5$	289,272,152	−3.80	--	●		
C23	28.830	447.1266		$C_{22}H_{22}O_{10}$	447,285	−4.47	Calycosin-7-O-β-D –glucoside			◎
C24	29.233	477.1387		$C_{23}H_{24}O_{11}$	477,315,300,283,255	−0.84	4-methoxy-maackiain-7-O-β-D –glucoside			◎
C25	30.070		637.2113	$C_{30}H_{38}O_{15}$	637,475,387	−3.92	--			◎

Table S2. *Cont.*

NO.	T_R (min)	$[M+H]^+$	$[M-H]^-$	Predicted Formula	Fragment ions	Error ppm	Identification	T	IT	S
C26	30.653		479.1492	$C_{30}H_{24}O_6$	479,317	−1.67	--			◎
C27	30.773	$[M+HCOO]^-$	671.2155	$C_{29}H_{38}O_{15}$	671,625,463,301	−5.66	Astraisoflavan-di-7-O-*β*-D-glucoside			◎
C28	31.005		579.2062	$C_{23}H_{36}N_2O_{15}$	579,417,387	3.28	--			◎
C29	31.348		445.1123	$C_{22}H_{22}O_{10}$	445,283	−3.82	Glycetein-4'-O-*β*-D-glucoside			◎
C30	32.078	463.1203		$C_{22}H_{22}O_{11}$	463,301	−6.91	Kaempferide-7-O-*β*-D-glucoside			◎
C31	32.662	533.1267		$C_{25}H_{24}O_{13}$	533,285	−4.31	6''-O-malonate-calycosin-7-O-*β*-D-glucoside			◎
C32	34.207	489.1398		$C_{24}H_{24}O_{11}$	489,285	1.43	6''-O-acetyl-calycosin-7-O-*β*-D-glucoside			◎
C33	34.550	431.1303		$C_{22}H_{22}O_9$	431,269	−7.89	Ononin		★	
C34	35.348		489.1340	$C_{24}H_{26}O_{11}$	489,285,271,159	−12.68	6''-O-acetyl-isosakuranetin-7-O-*β*-D –glucoside			◎
C35	35.580	549.1174		$C_{25}H_{24}O_{14}$	549,301	−11.84	6''-O-malonate-kaempferide-7-O-*β*-D–glucoside			◎
C36	36.027	463.1615		$C_{23}H_{26}O_{10}$	485(+Na^+),463,301	3.45	Astrapterocarpan-7-O-*β*-D -glucopyranoside			◎
C37	36.773		463.1577	$C_{23}H_{28}O_{10}$	463,301,271	−7.13	Astraisoflavan-7-O-*β*-D -glucopyranoside			◎
C38	38.035	285.0744		$C_{16}H_{12}O_5$	285,270,225,137	−4.91	Calycosin		★	
C39	38.653	315.0844		$C_{17}H_{14}O_6$		−6.03	4-methoxy-maackiain or the isomer			◎
C40	38.653	473.1445		$C_{24}H_{24}O_{10}$	473,269	0.63	The isomer of 6''-O-acetyl-ononin			◎
C41	38.773	447.1264		$C_{22}H_{22}O_{10}$	447,285	−4.92	Glycetein-7-O-*β*-D-glucoside			◎
C42	38.997	549.1545		$C_{26}H_{28}O_{13}$	549,301	−10.56	6''-O-malonate-astrapterocarpan-glucoside			◎
C43	39.452	517.1301		$C_{25}H_{24}O_{12}$	517,269	−7.73	6''-O-malonate-ononin			◎
C44	39.683		505.1699	$C_{25}H_{30}O_{11}$	505,301	−3.17	6''-O-acetyl-astraisoflavan-7-O-*β*-D-glucoside			◎
C45	39.743		957.5126	$C_{48}H_{78}O_{19}$		6.37	Soyasaponin Ba		★	
C46	40.138	473.1424		$C_{24}H_{24}O_{10}$	473,269	−3.80	6''-O-acetyl-ononin			◎
C47	40.430		503.1154	$C_{24}H_{24}O_{12}$	503,299	−8.15	6''-O-acetyl-kaempferide-7-O-*β*-D-glucoside			◎
C48	40.825		785.4629	$C_{41}H_{70}O_{14}$		−8.15	Cyclocanthoside E			◎
C49	41.108		991.5086	$C_{48}H_{80}O_{21}$		−3.33	Astragaloside V			◎
C50	41.778		315.0868	$C_{17}H_{16}O_6$	315,253	−1.90	Astragaluquinone or isomer			◎

Table S2. *Cont.*

NO.	T_R (min)	$[M+H]^+$	$[M-H]^-$	Predicted Formula	Fragment ions	Error ppm	Identification	T	IT	S
C51	41.950	533.1243		$C_{25}H_{24}O_{13}$	533,285	−8.82	6"-O-malonate-glycetein-7-O-β-D-glucoside			◎
C52	42.062		867.4635	$C_{45}H_{72}O_{16}$		−13.03	Isoastragaloside I			◎
C53	42.612	$[M+HCOO]^-$	829.4572	$C_{41}H_{68}O_{14}$		−2.29	Astragaloside IV			◎
C54	42.560		825.4532	$C_{43}H_{70}O_{15}$	871,825	−13.33	Isoastragaloside II			◎
C55	44.190		941.5081	$C_{48}H_{78}O_{18}$	941,525,437	−3.61	Soyasaponin Bb		★	
C56	45.315	269.0791		$C_{16}H_{12}O_4$	269,254,237,118	−6.32	formononetin		★	
C57	45.935		329.2299	$C_{18}H_{34}O_5$		−10.33	--			◎
C58	46.158	301.1052		$C_{17}H_{16}O_5$		−6.31	Astraoptercarpan			◎
C59	46.708	303.1181		$C_{17}H_{18}O_5$		−15.18	Astraisoflavan			◎
C60	46.768	$[M+HCOO]^-$	871.4656	$C_{43}H_{70}O_{15}$	871,825,603	−4.70	Astragaloside II			◎
C61	47.172	$[M+HCOO]^-$	911.4668	$C_{45}H_{70}O_{16}$	955,911	2.41	--			◎
C62	49.432		285.0423	$C_{15}H_{10}O_6$	285,163	6.31	Kaempferol or isomer			◎
C63	50.670		311.2212	$C_{18}H_{32}O_4$		−5.14	--	●		
C64	50.713	$[M+HCOO]^-$	913.4777	$C_{45}H_{72}O_{16}$		−2.74	Astragaloside I			◎
C65	51.607	$[M+HCOO]^-$	953.4637	$C_{47}H_{72}O_{17}$	953,909	−12.06	--			◎
C66	52.363	437.3374	$[M+Na]^+$	$C_{28}H_{46}O_2$		−3.66	--			◎
C67	59.933	213.0871	$[M+Na]^+$	$C_{12}H_{14}O_2$	403$[2M+Na]^+$,213,191	−7.04	Z-ligustilide		★	
C68	61.708	403.1867	$[2M+Na]^+$	$C_{12}H_{14}O_2$	403,381,191	−3.22	E-ligustilide		★	
C69	69.377		283.0238	$C_{15}H_8O_6$	283,203,147	−3.53	--	●		

T(●)The same unidentified proposed compounds of two kinds DBT; IT(★) The same identified proposed compounds of two kinds DBT; S(◎) The special proposed compounds of two kinds DBT in respectively.

Table S3. The identified compounds from DBT2 boiled sample by HPLC-IT-TOF-MS^n.

NO.	T_R (min)	$[M+H]^+$	$[M-H]^-$	Predicted Formula	Fragment ions	Error ppm	Identification	T	IT	S
c1	2.395		173.1044	$C_6H_{14}N_4O_2$		0.00	Arginine	●		
c2	2.575		195.0502	$C_6H_{12}O_7$		−4.10	Gluconic acid	●		
c3	2.695		341.1074	$C_{12}H_{22}O_{11}$		−4.40	D(+)sucrose	●		
c4	3.643		191.0187	$C_6H_8O_7$	191,173	−5.24	Citric acid	●		
c5	4.477		328.0427	$C_{11}H_{11}N_3O_9$		1.22	--	●		
c6	6.172		344.0387	$C_{11}H_{11}N_3O_{10}$		4.36	--	●		
c7	9.120	443.1146	[M+Na]+	$C_{17}H_{24}O_{12}$		−3.16	--			◎
c8	9.345	267.1369		$C_{18}H_{18}O_2$	267,225	−4.12	Magnolol			◎
c9	10.842	188.0674		$C_9H_{11}NO_2$		−4.25	L-phenylalanine	●		
c10	12.765		382.1007	$C_{13}H_{21}NO_{12}$		4.19	--	●		
c11	13.840		461.1283	$C_{19}H_{26}O_{13}$	461,167	−3.90	--			◎
c12	21.207		433.1129	$C_{21}H_{22}O_{10}$	433,285	−2.54	--			◎
c13	21.322		433.1121	$C_{21}H_{22}O_{10}$	433,285,241	−4.39	--			◎
c14	22.020		417.1017	$C_{17}H_{22}O_{12}$	417,285,152	−5.03	--		★	
c15	23.018		401.1445	$C_{18}H_{26}O_{10}$	401,269,161	−1.99	--	●		
c16	23.080	389.2325		$C_{23}H_{32}O_5$		0.51	--	●		
c17	26.998		287.1595	$C_{13}H_{24}N_2O_5$		−5.92	--	●		
c18	34.517	431.1322		$C_{22}H_{22}O_9$	431,269,237,118	−3.48	Ononin		★	
c19	37.480		255.0657	$C_{15}H_{12}O_4$	255,135	−2.35	Isoliquiritigenin			◎
c20	38.072		283.0596	$C_{16}H_{12}O_5$	283,268,240	−5.65	Calycosin		★	
c21	39.917		269.0807	$C_{16}H_{14}O_4$	269,253,227	−4.46	Isomer of alpinetin			◎
c22	39.977		255.0657	$C_{15}H_{12}O_4$	255,237	−2.35	Liquiritigenin			◎
c23	39.745		957.5030	$C_{48}H_{78}O_{19}$	957,541,453	−3.66	Soyasaponin Ba		★	
c24	40.903		927.4915	$C_{47}H_{76}O_{18}$		−4.74	Akebia saponin D			◎
c25	41.273		285.0751	$C_{16}H_{14}O_5$	285,194,109	−5.96	Isomer of isosakuranetin			◎

Table S3. *Cont.*

NO.	T_R (min)	$[M+H]^+$	$[M-H]^-$	Predicted Formula	Fragment ions	Error ppm	Identification	T	IT	S
c26	41.772		269.0456	$C_{15}H_{10}O_5$	269,237	0.37	Genistein			◎
c27	42.243		283.0602	$C_{16}H_{12}O_5$	283,268,224	−3.53	Glycetein			◎
c28	42.965		329.2319	$C_{18}H_{34}O_5$		−4.25	--			◎
c29	43.197		955.4857	$C_{48}H_{76}O_{19}$		−5.34	--			◎
c30	44.690		287.0577	$C_{15}H_{12}O_6$		5.57	Dihydro-kaempferol			◎
c31	44.982		255.0649	$C_{15}H_{12}O_4$	256,135	−5.49	Isomer of Liquiritigenin			◎
c32	45.393	269.0784		$C_{16}H_{12}O_4$	269,253,237	−8.92	Formononetin		★	
c33	45.745	299.0911		$C_{17}H_{14}O_5$	299,284,166		Pterocarpin			◎
c34	46.080		283.0599	$C_{16}H_{12}O_5$	283,255,240	−4.59	The isomer of glycetein			◎
c35	47.592		941.5080	$C_{48}H_{78}O_{18}$	947,525,437	−3.72	Soyasaponin Bb		★	
c36	47.772		299.0552	$C_{16}H_{12}O_6$		−3.01	Kaempferide or isomer			◎
c37	47.943		909.4836	$C_{47}H_{74}O_{17}$		−1.87	Acetylastragaloside I			◎
c38	48.347	$[M+HCOO]^-$	911.5011	$C_{46}H_{74}O_{15}$		0.11	Castaraleside H			◎
c39	50.065		939.4925	$C_{48}H_{76}O_{18}$		−3.62	--			◎
c40	50.435	285.0748		$C_{16}H_{12}O_5$	285,253,152	−3.51	Isomer of calycosin			◎
c41	50.667	335.2180	$[M+Na]^+$	$C_{18}H_{32}O_4$		−3.88	--	●		
c42	51.560	193.1212		$C_{12}H_{16}O_2$		−5.70	Senkyunolide A			◎
c43	55.212	191.1043		$C_{12}H_{14}O_2$		−12.56	n-butyl-phthalide			◎
c44	59.250	213.0876	$[M+Na]^+$	$C_{12}H_{14}O_2$	403,213	−4.69	Z-ligustilide		★	
c45	61.765	213.0893	$[M+Na]^+$	$C_{12}H_{14}O_2$	381,191,213	3.29	E-ligustilide		★	
c46	69.093		283.0257	$C_{15}H_8O_6$		3.18	--	●		

T(●)The same unidentified proposed compounds of two kinds DBT; IT(★) The same identified proposed compounds of two kinds DBT; S(◎) The special proposed compounds of two kinds DBT in respectively.

Table S4. The identified proposed metabolites from the urine sample after administrated DBT1 to rats.

NO.	T_R (min)	$[M+H]^+$	$[M-H]^-$	Predicted Formula	Fragment ions	Error ppm	Identification	T	IT	S
M1	4.130		287.0066	$C_{10}H_8O_{10}$		7.32	--	●		
M2	28.110	233.0909		$C_{12}H_{12}N_2O_3$	233，130	−5.15	--	●		
M3	28.848		233.0116	$C_{12}H_2N_4O_2$	233,169	4.72	--	●		
M4	29.415		337.1407	$C_{16}H_{22}N_2O_6$	337.193	0.59	--	●		
M5	31.338		275.0209	$C_{13}H_8O_7$	275,195	4.36	--	●		
M6	32.678		273.0063	$C_{13}H_6O_7$	273,193	8.06	--	●		
M7	33.038	271.0585		$C_{15}H_{10}O_5$		−5.90	Hydroxyl-daidzein			◎
M8	36.155		303.0863	$C_{16}H_{16}O_6$	303,151	−3.63	hydroxyl-calycosin, di-reduction($C^2=C^3$; $C^4=O$)			◎
M9	36.705		253.0492	$C_{15}H_{10}O_4$		−5.53	Daidzein		★	
M10	36.808		477.1372	$C_{23}H_{26}O_{11}$	477,301	−6.29	Astraisoflavan, glucuronidation			◎
M11	36.868		255.0662	$C_{15}H_{12}O_4$	255,149	−0.39	Daidzein, reduction($C^2=C^3$)		★	
M12	37.255	385.1478		$C_{18}H_{24}O_9$		−3.89	Hydro-ligustilide, glucuronidation			◎
M13	37.435		285.0751	$C_{16}H_{14}O_5$	285,269,149	−5.96	Calycosin, reduction($C^2=C^3$)		★	
M14	37.195		257.0809	$C_{15}H_{14}O_4$		−3.89	Daidzein, di-reduction($C^2=C^3$; $C^4=O$)			◎
M15	38.070		283.0605	$C_{16}H_{12}O_5$	283,268,211	−2.47	Calycosin		★	
M16	38.362		285.0751	$C_{16}H_{14}O_5$	285,270	−5.96	Calycosin, reduction($C^4=O$)			◎
M17	39.367		233.0098	$C_{12}H_2N_4O_2$		−3.00	--	●		
M18	40.260	$[M+HCOO]^-$	363.0748	$C_{16}H_{14}O_7$		7.16	Dihydroxy-calycosin, reduction($C^2=C^3$)			◎
M19	41.755		269.0441	$C_{15}H_{10}O_5$		−5.20	Hydroxy-daidzein			◎
M20	42.365		299.0554	$C_{16}H_{12}O_6$	299,284	−2.34	Hydroxy-calycosin, or isomer			◎
M21	42.923		299.0556	$C_{16}H_{12}O_6$	299,284	−1.67	Hydroxy-calycosin			◎
M22	44.315		257.0819	$C_{15}H_{14}O_4$		0.00	Isoliquiritigenin, reduction(C=C)		★	
M23	45.328	269.0793		$C_{16}H_{12}O_4$	269,253,237	−5.57	Formononetin		★	
M24	45.732		269.0798	$C_{16}H_{14}O_4$	269,254,135	−7.80	Formononetin, reduction($C^2=C^3$)		★	
M25	46.290	335.0201		$C_{15}H_{10}O_7S$	335,255	−5.67	Daidzein, sulfation		★	

Table S4. *Cont.*

NO.	T_R (min)	$[M+H]^+$	$[M-H]^-$	Predicted Formula	Fragment ions	Error ppm	Identification	T	IT	S
M26	47.862		363.0174	$C_{16}H_{12}O_8S$	363,268	−1.65	Calycosin, sulfation			◎
M27	47.922		333.0059	$C_{15}H_{10}O_7S$	333,253,225	−4.50	Daidzein, sulfation			◎
M28	48.205		349.0053	$C_{15}H_{10}O_8S$	349,269	8.31	Hydroxy-daidzein, sulfation		★	
M29	48.747		365.0347	$C_{16}H_{14}O_8S$	365,285	2.74	Calycosin, reduction(C^2=C^3), sulfation			◎
M30	49.193		365.0360	$C_{16}H_{14}O_8$ S	365,285	6.30	Calycosin, reduction(C^2=C^3), sulfation			◎
M31	49.623		351.0187	$C_{15}H_{12}O_8S$	351,271,149	1.99	Hydroxy-daidzein, reduction(C^2=C^3), sulfation			◎
M32	50.467		283.0595	$C_{16}H_{12}O_5$	283,268	−6.01	Isomer of calycosin			◎
M33	50.973		367.0483	$C_{16}H_{16}O_8S$	367,272,150	−2.72	Calycosin, di-reduction(C^2=C^3; C^4=O), sulfation			◎
M34	51.515		363.0180	$C_{16}H_{12}O_8S$	363,283	0.00	Calycosin, sulfation		★	
M35	52.563		335.0250	$C_{15}H_{12}O_7S$	335,255,135	5.67	Daidzein, reduction(C^2=C^3), sulfation			◎
M36	53.637		343.0835	$C_{18}H_{16}O_7$		3.50	--			◎
M37	55.165		321.0417	$C_{15}H_{14}O_6S$	321,241	−6.54	Equol, sulfation	●		
M38	59.468		337.0388	$C_{15}H_{14}O_7S$	337,257,243	0.30	Daidzein, di-reduction(C^2=C^3; C^4=O), sulfation			◎
M39	61.838	683.4277		$C_{35}H_{64}O_{11}$		−9.57	Related to astragaloside			◎
M40	62.002	639.4061		$C_{35}H_{58}O_{10}$		−6.57	Related to astragaloside			◎
M41	62.113	595.3742		$C_{33}H_{54}O_9$		−16.63	Related to astragaloside	●		
M42	62.414	507.3296		$C_{29}H_{46}O_7$		−3.94	Related to astragaloside			◎
M43	71.557		353.0324	$C_{15}H_{14}O_8S$	353,273	−3.68	Hydroxy-daidzein,di-reduction(C^2=C^3;C^4=O) sulfation			◎
M44	71.557		397.0250	$C_{16}H_{14}O_{10}S$	397,317	3.78	Dihydroxy-calycosin, reduction(C^2=C^3), sulfation			◎

T(●)The same unidentified metabolites from the urine administrated two kinds DBT; IT(★) The same standard metabolites from the urine administrated two kinds DBT; S(◎) The special metabolites from the urine administrated two kinds DBT in respectively.

Table S5. The identified proposed metabolites from the urine sample after administrated by DBT2 to rats.

NO.	T_R (min)	$[M+H]^+$	$[M-H]^-$	Predicted Formula	Fragment ions	Error ppm	Identification	T	IT	S
m1	4.132		287.0065	$C_{10}H_8O_{10}$		6.97	--	●		
m2	28.200		231.0768	$C_{12}H_{12}N_2O_3$	463,231	−3.03	--	●		
m3	29.298		233.0115	$C_{12}H_2N_4O_2$	233,169	4.29	--	●		
m4	29.298		337.1408	$C_{16}H_{22}N_2O_6$	337,253	0.89	--	●		
m5	31.083	268.1164	266.1021	$C_{13}H_{17}NO_5$		−4.89	--			◎
m6	31.970		275.0211	$C_{13}H_8O_7$	275,195	5.09	--	●		
m7	32.927		273.0056	$C_{13}H_6O_7$	273,193	5.49	--	●		
m8	34.518	$[M+HCOO]^-$	475.1244	$C_{22}H_{22}O_9$	475,267	−0.42	Isomer of ononin			◎
m9	36.583	255.0637		$C_{15}H_{10}O_4$	255,199	−5.88	daidzein		★	
m10	36.918		255.0651	$C_{15}H_{12}O_4$	255,149	−4.70	Daidzein, reduction(C^2=C^3)		★	
m11	37.442		255.0655	$C_{15}H_{12}O_4$		−3.14	Daidzein, reduction(C^4=O)			◎
m12	37.502		285.0751	$C_{16}H_{14}O_5$	285,269,149	−5.96	Calycosin, reduction(C^2=C^3)		★	
m13	38.033		283.0608	$C_{16}H_{12}O_5$	283,268	−1.41	Calycosin		★	
m14	39.047		273.0761	$C_{15}H_{14}O_5$	273,240,109	−2.56	hydroxy-daidzein, di-reduction (C^2=C^3; C^4=O)			◎
m15	40.128		233.0115	$C_{12}H_2N_4O_2$		4.29	--	●		
m16	41.630		283.0609	$C_{16}H_{12}O_5$	283,268,224	−1.06	Isomer of calycosin			◎
m17	42.180		283.0603	$C_{16}H_{12}O_5$	283,268,224	−3.18	Isomer of calycosin			◎
m18	44.327		257.0804	$C_{15}H_{14}O_4$	257,240	−5.83	Isoliquiritigenin, reduction(C=C)		★	
m19	45.322	269.0796		$C_{16}H_{12}O_4$	269,253,237	−4.46	Formononetin		★	
m20	45.733		269.0804	$C_{16}H_{14}O_4$	269,254,135	−5.57	Formononetin, reduction(C^2=C^3)		★	
m21	47.175		333.0052	$C_{15}H_{10}O_7S$	333,253	−6.61	daidzein, sulfation		★	
m22	47.775		299.0556	$C_{16}H_{12}O_6$	299,256	−1.67	Hydroxy-calycosin			◎
m23	49.072		333.0056	$C_{15}H_{10}O_7S$	333,253,208	−5.41	daidzein, sulfation			◎
m24	49.543		333.0059	$C_{15}H_{10}O_7S$	333,253	−4.50	Isomer of daidzein, sulfation			◎
m25	49.810		349.0033	$C_{15}H_{10}O_8S$	349,269,225	2.58	Hydroxy-daidzein, sulfation		★	

Table S5. *Cont.*

NO.	T_R (min)	$[M+H]^+$	$[M-H]^-$	Predicted Formula	Fragment ions	Error ppm	Identification	T	IT	S
m26	49.930		363.0184	$C_{16}H_{12}O_8S$	363,283	1.10	Calycosin, sulfation		★	
m27	50.325		337.0395	$C_{15}H_{14}O_7S$	337,257	2.37	Daidzein,di-reduction(C^2=C^3; C^4=O), sulfation			◎
m28	55.693		351.0172	$C_{15}H_{12}O_8S$	351,271	−2.28	Hydroxy-daidzein, reduction(C^2=C^3), sufaltion			◎
m29	57.015		321.0414	$C_{15}H_{14}O_6S$	321,241	−7.48	Equol, sulfation	●		
m30	59.728	619.3669	$[M+Na]^+$	$C_{36}H_{52}O_7$		10.33	Related to astragaloside			◎
m31	62.218	595.3742		$C_{33}H_{54}O_9$		−16.63	Related to astragaloside	●		
m32	63.292		509.3599	$C_{33}H_{50}O_4$		−7.26	Related to astragaloside			◎
m33	72.797		363.0197	$C_{16}H_{12}O_8S$	363,283	4.68	Calycosin, sulfation			◎
m34	73.543		347.0213	$C_{16}H_{12}O_7S$	347,267	−5.19	Formononetin, sulfation			◎

T(●)The same unidentified metabolites from the urine administrated two kinds DBT; IT(★) The same standard metabolites from the urine administrated two kinds DBT; S(◎) The special metabolites from the urine administrated two kinds DBT in respectively.

Table S6. The number comparison of the identified compounds between DBT1 and DBT2.

N.	ID.	S-ID.	T.ID.	S%	H+S	S%
DBT1	69	21	115-21=94	22.34	56	59.57
DBT2	46					
DBT1-U	44	19	75-19=56	33.93	48	85.71
DBT2-U	31					

ID.: Identified proposed compounds; **S-ID.**: Identified the common proposed compounds between two DBT; **T.ID.**: Identified the unfamiliar proposed compounds between two DBT; **H+S**: The total isoflavones and the total saponins; **S%**: The ratio about the selective compounds in the total identified proposed compounds number.
